# Nickel‐Catalyzed Three‐Component Difluoroalkylation‐Amination for the Direct Access to Anti‐Inflammatory β‐Difluoroalkyl Amines

**DOI:** 10.1002/advs.76993

**Published:** 2026-08-03

**Authors:** Chang Xu, Fangting Ma, Fan Ding, Luyan Wang, Yunxi Li, Yunzhi He, Xingang Zhang, Siyu He, Dandan Liu

**Affiliations:** ^1^ School of Pharmaceutical Sciences and Yunnan Key Laboratory of Pharmacology for Natural Products Kunming Medical University Kunming Yunnan People's Republic of China; ^2^ Yunnan Institute for Advanced Studies Ministry of Education Kunming People's Republic of China; ^3^ State Key Laboratory of Fluorine and Nitrogen Chemistry and Advanced Materials Shanghai Institute of Organic Chemistry University of Chinese Academy of Sciences Chinese Academy of Sciences Shanghai People's Republic of China

**Keywords:** anti‐inflammation, carboamination, in vitro and in vivo study, nickel catalysis, S_H_2‐type reaction, β‐difluoroalkyl amines

## Abstract

Fluoroalkyl amines represent a promising structural motif in pharmaceutical sciences, but the development of an efficient and practical synthetic method is in urgent need, especially for β‐difluoroalkyl amines, in which the concurrent introduction of fluoroalkyl and aminyl groups remains challenging. Here, we report a nickel‐catalyzed three‐component reductive difluoroalkylation‐amination strategy of enamides for the direct access to the α‐amidyl‐β‐difluoroalkyl amines. This strategy utilizes carboamination of alkene with nitrogen electrophiles and fluoroalkyl chlorides to simultaneously forge C─C and C(sp^3^)‐N bond, being compatible with various chlorodifluoroacetamides and nitrogen electrophiles, including amino acid‐derived or drug‐derived substrates. The synthetic method features with broad substrate scope, mild conditions, high functional group tolerance, and easy handling. Control experiments and DFT calculations are performed to elucidate the mechanism of the reaction, and an S_H_2 pathway regarding C─N bond‐forming step is proposed. More remarkably, the products α‐amidyl‐β‐difluoroalkyl amines exhibit prominent anti‐inflammatory activity both in vitro and in vivo, indicating promising applications of this strategy in medicinal chemistry and drug development.

## Graphical abstract

A nickel‐catalyzed three‐component reductive fluoroalkylation‐amination strategy of *N*‐vinylamides for the direct access to the α‐amidyl‐β‐difluoroalkyl amines was reported here. The synthetic method features with broad substrate scope, mild conditions, high functional group tolerance, and convenience to handle. Mechanistic studies suggested the involvement of S_H_2 pathway. More remarkably, potent anti‐inflammatory activity was found in the products α‐amidyl‐β‐difluoroalkyl amines, showing promising applications of this strategy in medicinal chemistry and drug development.

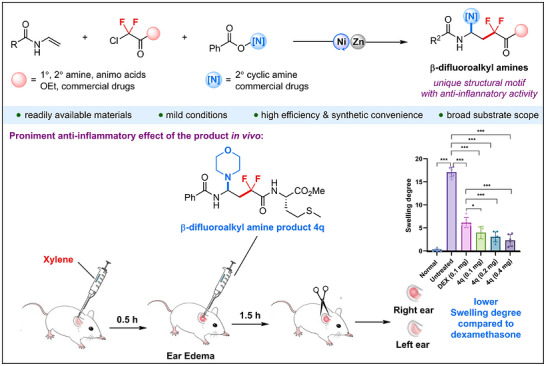

## 1 Introduction

**FIGURE 1:**
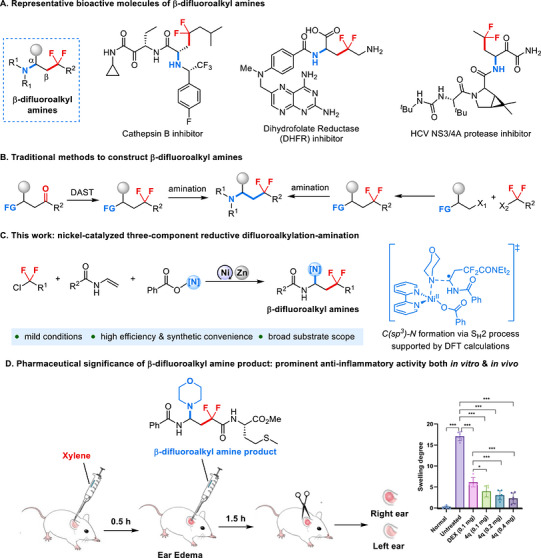
(A) Representative bioactive molecules of β‐difluoroalkyl amines. (B) Traditional methods to synthesize β‐difluoroalkyl amines. (C) This work: nickel‐catalyzed three‐component reductive difluoroalkylation‐amination. (D) Pharmaceutical significance of α‐amidyl‐β‐difluoroalkyl amine constructed by current method: prominent anti‐inflammatory activity both in vitro and in vivo.

Introducing fluorine‐containing groups into organic molecules often dramatically influences their physical, chemical, and biological properties. Hence, the development of synthetic methods for organofluorine compounds has gained increasing attention in medicinal chemistry and related fields [1, 2, 3, 4]. Among them, nitrogen‐based organofluorine compounds are a class of privileged compounds that play a crucial role in the pharmaceutical and agrochemical industry [5]. The incorporation of fluorinated groups into nitrogen compounds such as aliphatic amines and amides that are prevalent in the pharmaceutical industry would modify their physicochemical properties and enhance pharmacological effects, altering their solubility, lipophilicity, ionization ability, and target binding affinity [6, 7, 8]. The difluoromethylene group (CF_2_), one of the crucial fluorinated functionalities, serves as the bioisostere of an oxygen atom or a carbonyl group, providing additional effects on the modulation of bioactivity. Specifically, the installation of CF_2_ group at β‐position of aliphatic amines to form β‐difluoroalkyl amines represents a promising method in drug development due to the presence of this structural motif in a variety of bioactive molecules [9, 10, 11]. (Figure 1A). Given the aforementioned advantages, developing an efficient method to construct β‐difluoroalkyl amine scaffold is essential to facilitate its further research and development in medicinal chemistry.

Traditional method to construct β‐difluoroalkyl amine structure is usually indirect and requires multi‐step approaches including: (a) the fluorination of carbonyl group by DAST followed with introducing aminyl group into the molecule [10, 11, 12, 13] or (b) introduction of fluoroalkyl fragment into nitrogenous skeleton using nucleophilic addition [11] (e.g. Reformatsky reaction) or nucleophilic substitution [9] followed by functional group transformations (Figure 1B). Although those methods are able to afford the desired compounds, the redundancy and lack of efficiency partly hindered the in‐depth research and broad application of this structure in pharmaceutical sciences.

To overcome the traditional limitations, carboamination of alkenes has emerged as a highly reliable and efficient strategy for the direct construction of complex aliphatic amines. By simultaneously forging C─C and C─N bonds across carbon‐carbon double bonds, this approach provides straightforward access to diverse amine derivatives from simple synthons [14, 15, 16]. Furthermore, the recent application of electrophilic amination reagents via a nitrogen “umpolung” strategy has significantly advanced this field, successfully circumventing the intrinsic kinetic and thermodynamic barriers of conventional nucleophilic amination [17]. In particular, transition metal‐catalyzed three‐component carboamination utilizing electrophilic nitrogen sources has proven to be a highly modular and versatile toolkit for generating functionalized amines. Among the various transition metals explored for this umpolung alkene amination reaction, nickel has exhibited exceptional capability [18, 19, 20, 21, 22, 23, 24, 25, 26, 27, 28, 29, 30, 31, 32]. Owing to the cheapness, earth‐abundance, and versatile catalytic modes of nickel, nickel‐catalyzed alkene carboamination has attracted growing attention in recent years as a powerful strategy for the synthesis of functionalized aliphatic amines [33, 34]. However, despite a few examples [35, 36], the fluoroalkylative amination of alkenes via nickel catalysis remains relatively underdeveloped. Meanwhile, constructing C(sp^3^)‐N bonds continues to pose challenges, which often arise from sluggish C(sp^3^)‐N reductive elimination and the propensity for β‐hydride elimination [37]. These challenges to some extent hinder the development of this transformation. In order to address the above issues and develop a general method for building β‐difluoroalkyl amine structures efficiently, we envisioned that a nickel‐catalyzed three‐component alkene fluoroalkylation‐amination strategy would be feasible under mild conditions with a nickel catalyst using two electrophiles, enabling the concurrent implementation of fluoroalkyl and nitrogen functional groups into carbon–carbon double bond (Figure 1C).

Here, we report an efficient and straightforward method for the synthesis of α‐amidyl‐β‐difluoroalkyl amines by a nickel‐catalyzed reductive carboamination strategy of *N*‐vinylamides with difluoroalkyl chlorides and *O*‐benzoylhydroxylamine. This protocol integrates the umpolung characteristic of electrophilic aminating reagents with reductive tandem reaction [38, 39] to facilitate the formation of C(sp^3^)‐N bond. By fine‐tuning the nickel catalytic system, a broad array of α‐amidyl‐β‐difluoroalkyl amines can be prepared efficiently under mild reaction conditions with various chlorodifluoroacetamides and *O*‐benzoylhydroxylamines, including a number of amino acid‐derived or drug‐derived substrates, showing potential applications in medicinal chemistry. Moreover, mechanistic studies suggested an S_H_2 process was involved in C(sp^3^)‐N forming step, which provides a new understanding for the development of a novel mode for C(sp^3^)‐N construction (Figure 1C). In addition to the β‐difluoroalkyl amine motif, this carboamination method can also afford a geminal diamine structure that is a pivotal class of structural motif in pharmaceutical sciences [40, 41, 42]. We envisioned that the integration of β‐difluoroalkyl amine and geminal diamine structure via this nickel‐catalyzed reductive fluoroalkylation‐amination strategy would provide new insight for β‐difluoroalkyl amines in medicinal chemistry. More remarkably, one of these α‐amidyl‐β‐difluoroalkyl amine products exhibits prominent anti‐inflammatory activity in pharmacological tests both in vitro and in vivo, demonstrating this fluoroalkylative amination method of alkene would offer a new toolkit for the construction and modification of nitrogen‐containing organofluorine compounds of pharmaceutical significance (Figure 1D).

## 2 Results and Discussion

### 2.1 Development of the Reaction

To achieve the hypothetical reaction design, the catalytic system was investigated in which *N*‐vinylbenzamide **1a**, chlorodifluoroacetamide **2a** and nitrogen source **3a** were chosen as the model substrates under nickel‐catalyzed reductive condition. Inspired by our previous work [43, 44, 45], initially, a series of nitrogen ligands were tested with NiCl_2_•(DME) (5 mol%) in the presence of zinc powder (2.0 equiv.) and MgCl_2_ (1.5 equiv.), showing that most of the bipyridine‐ and phenanthroline‐type ligands can smoothly promote the reaction, but the ligands with steric hindrance led to a lower yield (Table 1, entry 1–6). Of all ligands, 2,2’‐ bipyridine **L1** was the optimal, with the delivery of **4a** in 71% yield (for details, see Table S1). Then the examination of [Ni]/ligand ratio suggested that decreasing the loading amount of nickel catalyst to 2.5 mol% led to a little higher yield of **4a** in 79% and 77% (entry 7, 8), and increasing the loading of ligand had limited effect (entry 9, for details, see Table S2). Considering the reaction efficiency and the cost of catalyst, 2.5 mol% of nickel with 5 mol% of **L1** would be preferable. Subsequent survey of nickel catalysts showed that NiCl_2_(dppp) gave the best result (87% yield) and a similar yield (83%) was observed with NiCl_2_(dppe) (entry 10, 11). Other catalysts were less effective (for details, see Table S3). A series of leaving groups of nitrogen electrophile **3** were tested, and the best was still benzoyl group. Either electron‐biased groups or those with steric hindrance would result in a reduced yield (for details, see Table S4). Next, the investigation of the molar ratio of substrates and Zn/MgCl_2_ suggested that a 1/1.4/1.1 ratio of **1a**/**2a**/**3a** and a 2.0/2.5 ratio of Zn/MgCl_2_ would increase the yield of **4a** to 94% (entry 12, for details, see Tables S5 and S6). Following screening of solvents indicated that DMA remained the best. DMF gave a comparable result but the reaction performed in DMF would produce more unknown defluorinated byproducts (for details, see Table S7). Control experiments showed that both the nickel catalyst and ligand as well as the presence of Zn and MgCl_2_ were indispensable for this reaction (entry 13–16, for details, see supporting information Table S8).

**TABLE 1:** Optimization of the reaction conditions^a^.

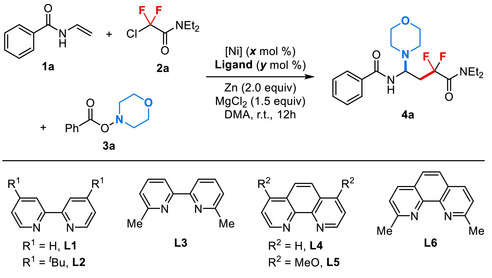				
entry	[Ni]	Ligand	***x***/***y***	**4a**, yield [%]^b^
1	NiCl_2_•(DME)	**L1**	5/5	71
2	NiCl_2_•(DME)	**L2**	5/5	64
3	NiCl_2_•(DME)	**L3**	5/5	48
4	NiCl_2_•(DME)	**L4**	5/5	55
5	NiCl_2_•(DME)	**L5**	5/5	7
6	NiCl_2_•(DME)	**L6**	5/5	35
7	NiCl_2_•(DME)	**L1**	2.5/5	79
8	NiCl_2_•(DME)	**L1**	2.5/2.5	77
9	NiCl_2_•(DME)	**L1**	5/10	75
10	NiCl_2_(dppp)	**L1**	2.5/5	87
11	NiCl_2_(dppe)	**L1**	2.5/5	83
12^c^	NiCl_2_(dppp)	**L1**	2.5/5	94 (87)
13	None	**L1**	0/5	ND
14	NiCl_2_(dppp)	None	2.5/0	6
15^c^, ^d^	NiCl_2_(dppp)	**L1**	2.5/5	ND
16^c^, ^e^	NiCl_2_(dppp)	**L1**	2.5/5	trace

^a^ Reaction conditions (unless otherwise specified): **1a** (0.4 mmol, 1.0 equiv.), **2a** (1.2 equiv.), **3a** (1.2 equiv.), Zn (2.0 equiv.), MgCl_2_ (1.5 equiv.) and DMA (3 mL) at room temperature.

^b^ Determined by ^19^F NMR using fluorobenzene (0.4 mmol) as an internal standard. Value in parenthesis is isolated yield. ND, not detected.

^c^ **1a** (0.4 mmol, 1.0 equiv.), **2a** (1.4 equiv.), **3a** (1.1 equiv.), Zn (2.0 equiv.), MgCl_2_ (2.5 equiv.) and DMA (3 mL).

^d^ Reaction was run without Zn.

^e^ Reaction was run without MgCl_2_.

### 2.2 Substrate Scope

**SCHEME 1:**
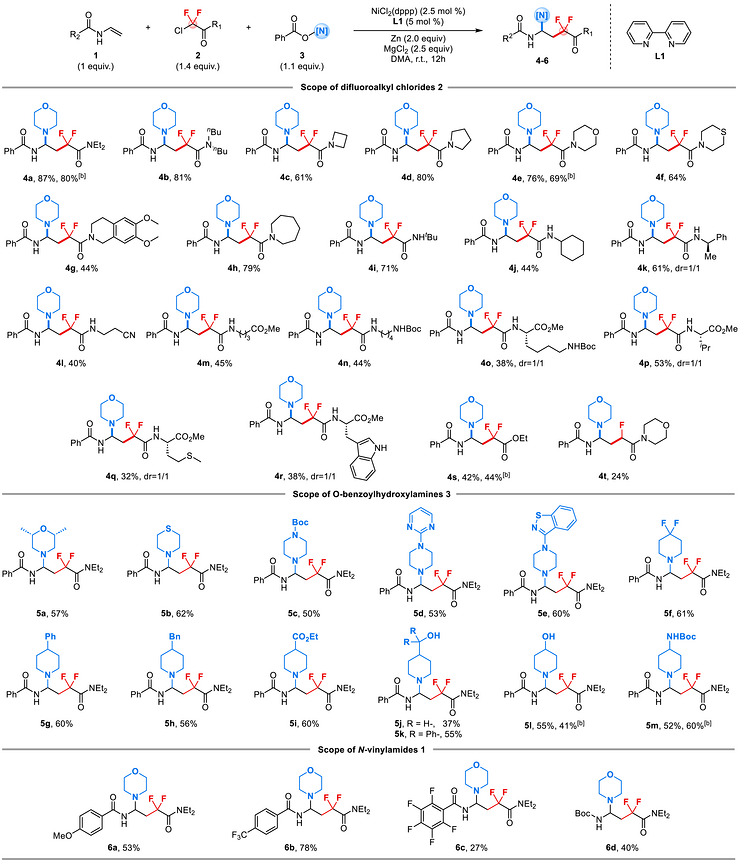
Nickel‐catalyzed three‐component fluoroalkylation‐amination of alkene **1** with difluoroalkyl chlorides **2** and nitrogen electrophiles **3**. ^[a]^Reaction conditions (unless otherwise specified): **1** (0.4 mmol, 1.0 equiv.), **2** (1.4 equiv.), **3** (1.1 equiv.), Zn (2.5 equiv.), MgCl_2_ (2.0 equiv.) and DMA (3 mL). Yields of isolated products are given. ^[b]^Gram‐scale synthesis. Reaction scale: **4a**, **4e** (4 mmol **1a** was used), **4s** (8 mmol **1a** was used), **5l** (5 mmol **1a** was used), **5m** (7 mmol **1a** was used).

With the optimized reaction conditions in hand, the substrate scope was investigated for the reaction of *N*‐vinylamide‐type alkene **1** with difluoroacetamide chlorides **2** and nitrogen electrophiles **3** (Scheme 1, for the preparation and structures of all starting materials, see supporting information Figures S1–S3). In general, good to excellent yields of α‐amidyl‐β‐difluoroalkyl amines were obtained with high functional group compatibility under mild conditions. A variety of difluoroacetamide chlorides were compatible with the catalytic system, such as substrates bearing secondary acyclic amides (**4a**, **4b**), cyclic amides of four‐ to six‐membered rings (**4c**‐**4 h**) and primary amides (**4i**‐**4k**), including dimethoxy tetrahydroisoquinoline (**4** **g**) which is prevalent in many natural products. The thiomorpholine‐containing substrate (**4f**) was also feasible in the reaction without poisoning the nickel catalyst. Important functional groups such as cyano, ester, and amino groups were well tolerated in the reaction (**4l**‐**4n**). Notably, a series of amino acid‐derived chlorodifluoroacetamides were applicable to the reaction, affording the corresponding fluorinated amino acid analogues effectively (**4o**‐**4r**), even the unprotected tryptophan‐derived substrate didn't impede the reaction (**4r**). In addition to chlorodifluoroacetamides, ethyl chlorodifluoroacetate was also viable with the reaction, giving the difluoroamino acid ester (**4s**) in moderate yield. Apart from difluoroalkyl chlorides, monofluoroacetamide chloride was also compatible with the reaction, although only 24% yield of **4t** was obtained, the result has demonstrated the broad substrate scope of this method.

Next, the scope of nitrogen electrophiles **3** was examined. Various aminating agents gave the products with good yields, including substrates possessing 2,6‐dimethylmorpholine (**5a**), thiomorpholine (**5b**), piperazine bearing Boc, pyrimidine and benzoisothiazole (**5c**–**5e**). These heterocycles are significant structural components in drug development [46, 47]. Difluoro, alkyl and aryl substituted piperidines were feasible in the reaction (**5f**–**5 h**), as well as the piperidines with important functional groups such as ester (**5i**), hydroxyl (**5j**–**5l**) and Boc‐protected amine (**5m**). For alkenes, *N*‐vinylbenzamide with electron‐donating methoxy (**6a**), electron‐withdrawing trifluoromethyl (**6b**) and perfluorophenyl (**6c**) delivered the product smoothly. The low yield of **6c** was presumably attributed to the strong electron‐withdrawing effect of the perfluorophenyl group, which reduced the reactivity of the alkene. Besides benzamide, *tert*‐butyl vinylcarbamate afforded **6d** in 40% yield, and the Boc‐protected amine provided a potential site for downstream derivatization of the product. The reaction can be readily scaled up, as illustrated by the gram‐scale synthesis of **4a**, **4e**, **4s**, **5l** and **5m** with comparable or even higher efficiency.

### 2.3 Synthetic Applications

**SCHEME 2:**
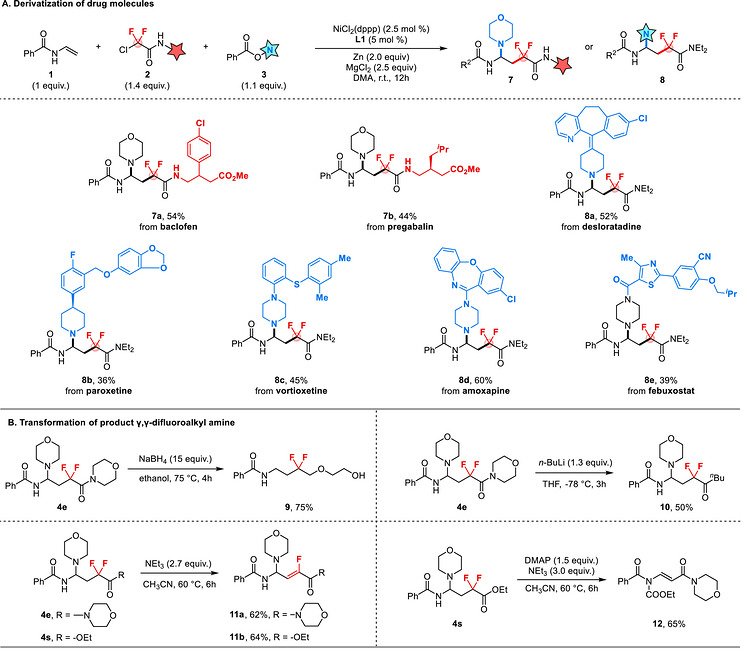
Synthetic applications. (A) Derivatization of drug molecules. (B) Transformation of the products α‐amidyl‐β‐difluoroalkyl amines.

The synthetic utilization of this method is further demonstrated by the derivatization of drug molecules and transformation of products α‐amidyl‐β‐difluoroalkyl amines (Scheme 2). Drug molecules bearing free primary or secondary amine groups could be directly incorporated into the products by this protocol (Scheme 2A). For instance, baclofen and pregabalin with primary amine groups can be introduced into β‐difluoroalkyl amine motif with difluoroacetamide as the linker, delivering **7a** in 54% yield and **7b** in 44% yield, respectively. Similarly, drugs such as paroxetine, vortioxetine, amoxapine, and febuxostat that bear secondary amine groups can be easily introduced into alkene **1a** to deliver the products in good yields (**8a**‐**8d**). Notably, the aryl chlorides in these biomolecules were compatible with the reductive conditions (**7a**, **8a** and **8d**) without interfering with the catalytic system. Drug molecule containing a carboxyl group such as febuxostat, is also a feasible conjugate partner as piperazine can serve as a linker to assemble the drug molecule with alkene and difluoroalkyl chloride, affording **8e** in 39% yield. These examples indicated that this method is capable of diversifying bioactive molecules through amine or carboxyl groups that are prevalent in pharmaceutical sciences, providing a useful toolkit of late‐stage modification for drugs in medicinal chemistry.

The chemical characteristics of the products have also been explored through several transformations (Scheme 2B). Subjecting **4e** to 15 equiv. of NaBH_4_ not only reduced difluoroalkyl amide motif but also made morpholine undergo deaminative hydrogenation to give amidyl difluoroalkyl compound **9** in 75% yield. The difluoroalkyl amide motif of **4e** was not only reduced to a hydroxyl group [48], but also was further attached with a hydroxyethyl group. Morpholine amides are known to serve as surrogates for Weinreb amides [49], thus treatment of **4e** with *n*‐BuLi afforded aminyl difluoroalkyl ketone **10** in 50% yield. In the presence of triethylamine as an organic base, both difluoroalkyl amide **4e** and difluoroalkyl ester **4s** were prone to undergo HF elimination reaction at 60°C to give monofluoroolefins **11a** in 62% yield and **11b** in 64% yield, respectively, which offering an alternative method to synthesize nitrogen‐containing functionalized monofluoroolefins. These results could be explained by the distinctive structure of α‐amidyl‐β‐difluoroalkyl amine structure, as β‐H is affected by the electron‐withdrawing effect of both difluoroalkyl amide and the geminal diamine group. Interestingly, dealing **4s** with DMAP and triethylamine at 60°C gave a particular defluorinative rearrangement product **12** in 65% yield, although the reaction mechanism is unclear at present; this result represents the unique characteristic deriving from the special structure of the products. These examples have implied the potential synthetic utility of the present reaction and the unique feature of α‐amidyl‐β‐difluoroalkyl amine.

### 2.4 Mechanistic Investigations

**SCHEME 3:**
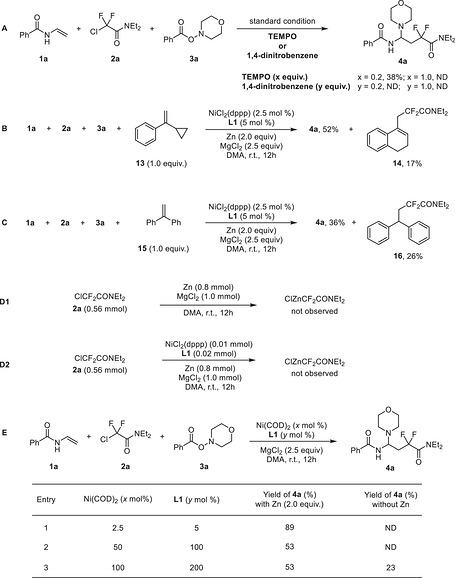
Preliminary mechanistic experiments. (A) Radical inhibition experiment. (B) Radical trapping experiment with α‐cyclopropylstyrene. (C) Radical trapping experiment with 1,1‐diphenylethylene. (D) Reaction of 2a with zinc powder. (E) Control experiments using Ni(COD)_2_.

To probe how the C‐N bond was forming under these reductive condition and shed light on the mechanism of this reaction, several preliminary experiments were conducted (Scheme 3). Radical inhibition experiments with TEMPO or electron‐transfer inhibitor 1,4‐dinitrobenzene [50] dramatically suppressed the reaction, indicating a SET process and radical reaction were involved (Scheme 3A). The addition of radical scavengers α‐cyclopropylstyrene **13** or 1,1‐diphenylethylene **15** in the standard reaction decreased the yield of **4a** to 52% and 36% respectively, with the simultaneous formation of radical‐trapping products **14** (17% yield with **13**) and **16** (26% yield with **15**) (Scheme 3B,C). This result suggested a difluoroacetamidyl radical was formed during the reaction. In order to probe whether a difluoroacetamide zinc reagent was formed [44], **2a** and zinc powder were mixed and stirred under argon for 12 h. Whether a nickel catalyst was present or not, the difluoroacetamide zinc reagent was not formed, thus the pathway involving fluoroalkyl zinc reagent can be ruled out (Scheme 3D1,D2). The use of a Ni(0) source, Ni(COD)_2_, instead of Ni(II) in the absence of zinc powder, failed to deliver **4a** with 2.5% and 50% catalyst loading and afforded only 23% yield of **4a** with 100% catalyst loading. Yet in the presence of zinc powder, 2.5% of Ni(COD)_2_ led to 89% yield of **4a**. Increasing the catalyst loading to 50% and even 100% delivered **4a** both in 53% yield (Scheme 3E & Table S9). The results of these control experiments with Ni(COD)_2_ plus the optimization result in Table 1 (entry 15) revealed that both Ni(0) and Ni(II) are not responsible for initiating the reaction and that zinc is critical in triggering and maintaining the reaction. It is reasonable to reckon that Ni(I) complex is probably the catalytically active species under these reductive conditions.

**FIGURE 2:**
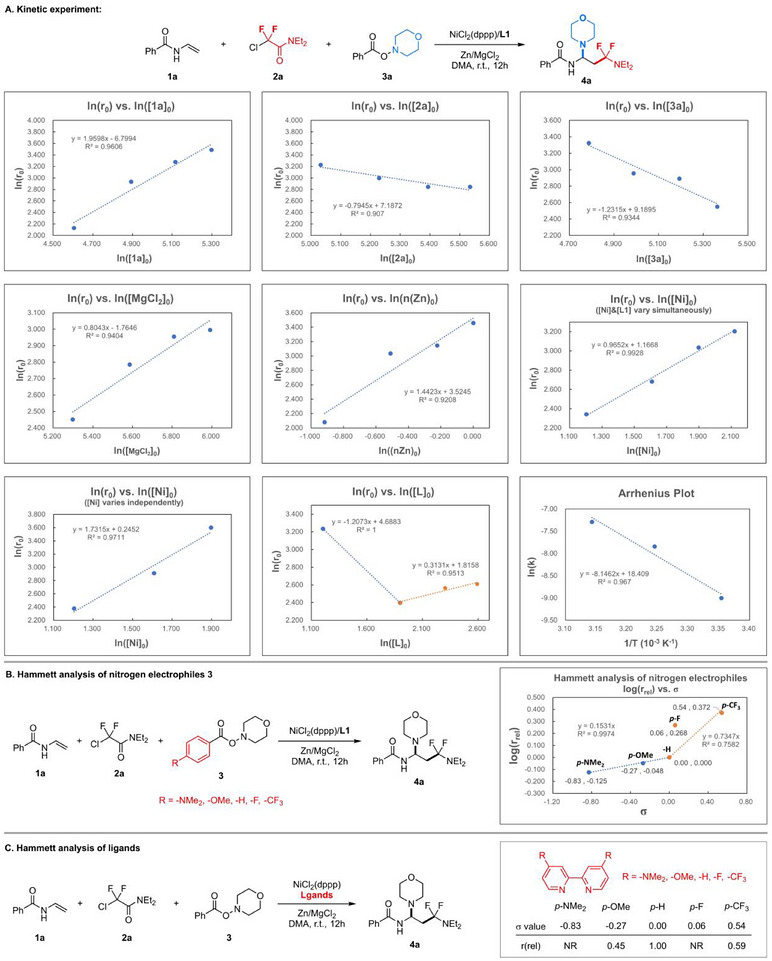
Kinetic experiments and Hammett analysis. (A) Kinetic experiment using initial rates method. (B) Hammett analysis on laving groups of nitrogen electrophiles 3. (C) Hammett analysis on ligands.

To gain more information on the reaction mechanism, a series of kinetic experiments were performed. Using the initial rates method, all components of the reaction were examined, and the relationship between ln(r_0_) (logarithm of initial rates) and ln(c_0_) (logarithm of initial concentration) was plotted (Figure 2A, for the details of kinetic data and plots of initial rates, see supporting information Figures S4–S12 and Tables S10–S18). According to the plot, the reaction orders for the components were as follows: 2 for **1a**, ‐1 for **2a**, ‐1 for **3a**, 1 for MgCl_2_, 1 for zinc (using n_0_ instead of c_0_) and nearly 1 for nickel/**L1** together (when independently varying, the order for nickel was 1.7; ‐1.2 for **L1** when the loading of **L1** was less than 5 mol%). The kinetic data suggested that the turnover‐limiting step for this reaction involved all the components, including zinc and MgCl_2_. Considering the result of control experiments (Scheme 3E), the turnover‐limiting step was likely the reduction process of Ni(II) pre‐catalyst by zinc to the catalytically active Ni(I) species under the facilitation of MgCl_2_ [44, 51]. It can be inferred from the result of kinetic experiments that: (1) the Ni(II) complex to be reduced (**CPA**) was probably formed by reaction of N(II) precatalyst, bpy and bimolecular **1a** and **1a** might play a role in promoting the reduction process through binding with Ni(II)/bpy; (2) the reduction of **CPA** could be hindered by **2a** and **3a**, possibly through the competitive binding with Ni(II) pre‐catalyst to impede the formation of **CPA**; (3) the initial rate decreased when the loading of **L1** was increasing over 2.5 mol% but the rate started to increase slowly after reaching the minimum at 5 mol% loading of ligand, suggesting that the formation of a 1/1 complex of nickel catalyst with **L1** was vital in the catalytic cycle because the rate was maximum when the amount of **L1** was equal to the Ni(II); (4) the Arrhenius plot showed a linear correlation with a negative slope of −8.146, which gave the apparent experimental activation energy of 16.2 kcal/mol, implying that the energy barrier of reduction process might be 16.2 kcal/mol (for the details of Arrhenius plot data, see Tables S21 and S22).

Hammett analysis on the leaving groups of nitrogen electrophiles **3** and substituents of ligands was also conducted using the initial rates method. Five nitrogen electrophiles with electronically varied leaving groups were examined, and the relationship between the substituent parameter σ and the logarithm of relative reaction rate [log(r_R_/r_H_)] was plotted (Figure 2B, for the details of kinetic data, see Figure S13 and Table S19). The plot showed linear correlation within electron‐donating groups or electron‐withdrawing groups, respectively, and the slope of EWGs was higher than EDGs, indicating the reaction was more sensitive to substituent effects with EWGs. The reaction rate was increased while increasing the electron‐withdrawing effect of **3**’s leaving groups. The positive slope (namely reaction constant ρ) indicated that negative charge was building near the carboxyl group when the N─O bond of **3** was broken. The Hammett analysis of ligands showed that ligands bearing dimethylamine and fluorine failed to initiate the reaction and ligands bearing methoxy and trifluoromethyl reduced the initial rates (Figure 2C, for the details of kinetic data, see Figure S14 and Table S20). Though a clear linear correlation was not revealed, the result indicated that the reduction process of Ni(II) complex did play a vital role in initiating and maintaining the reaction because the electronic properties of ligands would certainly affect the properties of Ni(II) complex, thereby had an influence on the reduction process.

**SCHEME 4:**
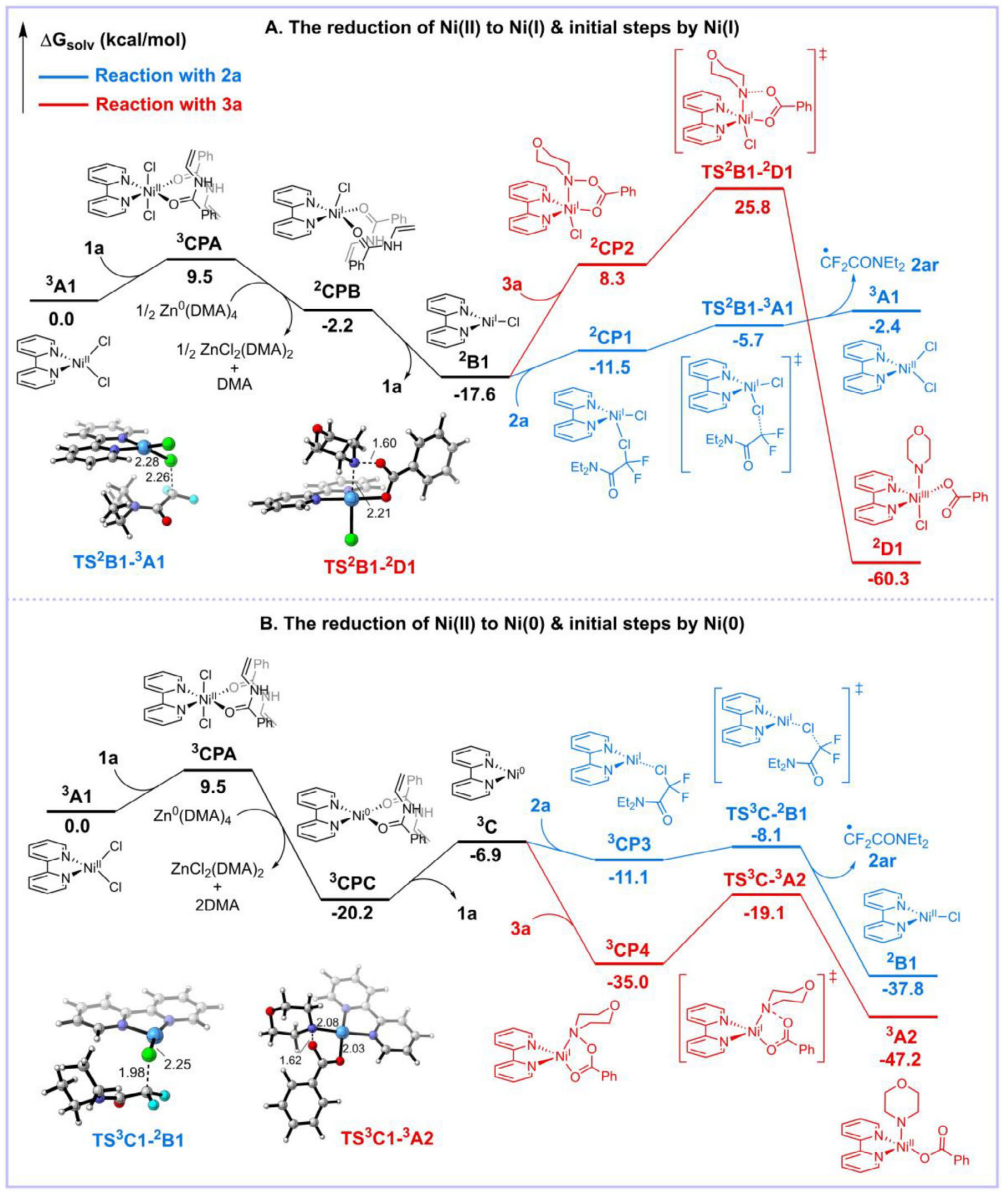
The Gibbs free energy profile for the process of reduction of Ni(II) and initial steps. (A) The reduction of Ni(II) to Ni(I) and initial steps by Ni(I). (B) The reduction of Ni(II) to Ni(0) & initial steps by Ni(0). Calculated at RI‐(U)PWPB95‐D3/def2‐QZVPP‐SDD(Ni)‐SMD‐(DMA)//(U)B3LYP‐D3(BJ)/def2‐SVP‐SDD(Ni)‐SMD‐(DMA) level of theory.

Further understanding on reaction mechanism was provided by DFT calculations. Based on the optimization results (Table 1 & Table S3), NiCl_2_(dppp) only serves as an effective precatalyst, and the ligand bpy, instead of dppp, plays a crucial role in promoting the reaction. Thus, the active nickel complexes calculated were considered as bpy‐ligated nickel generated by ligand exchange between NiCl_2_(dppp) and bpy. Firstly, free energy profile of the two possible pathways for the reduction process of Ni(II) complex as well as the initial steps by low‐valent nickel species were calculated (Scheme 4) [52]. In both pathway A and B, the formation of bimolecular **1a** ligated Ni(II) complex was uphill in free energy (**CPA**, ΔG = 9.5 kcal/mol), but the reduction process was both favorable for the generation of Ni(I) (**CPB**, ΔG = −11.7 kcal/mol) and Ni(0) (**CPC**, ΔG = −29.7 kcal/mol), suggesting that **1a** could facilitate the reduction process (Scheme 4A,B). In pathway A (Scheme 4A), subsequent dissociation of **1a** from **CPB** gave active species (bpy)Ni^I^Cl **B1,** which would bind with **2a** or **3a** to form complexes **CP1** or **CP2,** respectively. **CP1** underwent chlorine abstraction to give (bpy)Ni^II^Cl_2_ **A1** and difluoroacetamidyl radical **2ar** via transition‐state **TS^2^B1‐^3^A1** with an energy barrier of 5.8 kcal/mol. **CP2** underwent oxidative addition with **3a** to give Ni(III) complex **D1** via **TS^2^B1‐^2^D1** with a high energy barrier of 17.5 kcal/mol. The calculation suggested that the reaction of Ni(I) complex with **2a** was kinetically more favorable than that of Ni(I) with **3a**. In pathway B (Scheme 4B), dissociation of **1a** from **CPC** to release (bpy)Ni° complex **C** was thermodynamically unfavorable (ΔG = 13.3 kcal/mol) compared to the formation of **B1**. Upon generation, **C** would bind with **2a** or **3a** to form complexes **CP3** or **CP4,** respectively. **CP3** underwent chlorine abstraction to form (bpy)Ni^I^Cl **B1** and difluoroacetamidyl radical **2ar** via **TS^3^C‐^2^B1** with a low energy barrier of 3.0 kcal/mol. **CP4** underwent oxidative addition into N−O bond via **TS^3^C‐^3^A2** with an energy barrier of 15.9 kcal/mol. This result also suggested that the reaction of Ni(0) complex with **2a** was kinetically more favorable than the reaction of Ni(0) with **3a**. Based on the above results and from an energetic view, at the initial stage of the reaction, the reduction of Ni(II) complex to Ni(I) complex by zinc was more likely to occur, and Ni(I) complex **B1** preferred to react with **2a** to initiate the reaction by chlorine abstraction, affording difluoroacetamidyl radical **2ar** and Ni(II) complex **A1**. Although it seems that Ni(0)‐initiated pathway is also possible from the computational view, the results of control experiment (Scheme 3E) exclude this possibility since there were probably some off‐cycle reactions related with Ni(0) complex.

**SCHEME 5:**
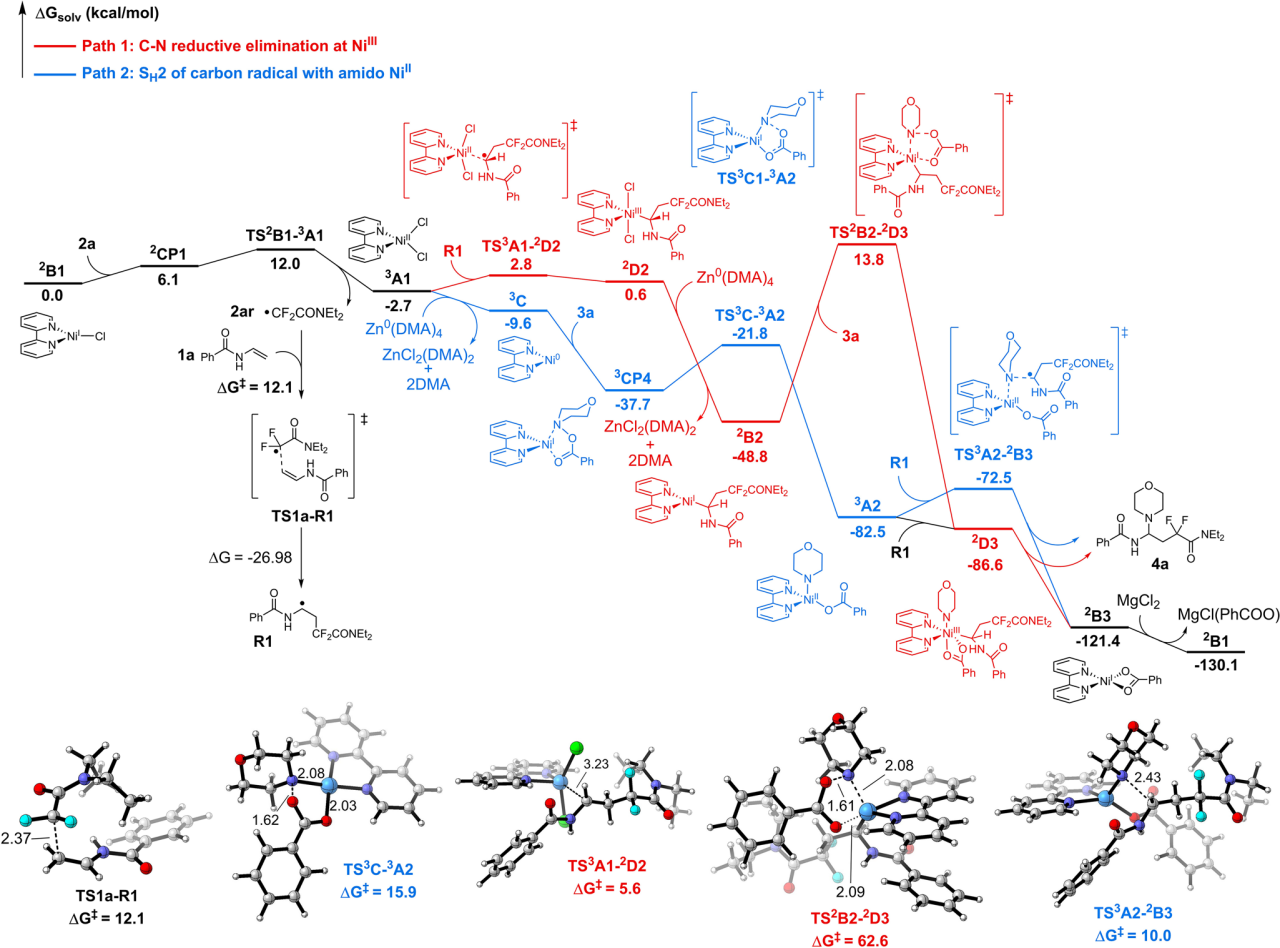
The Gibbs free energy profile for the catalytic cycle initiated by Ni(I) complex. Calculated at RI‐(U)PWPB95‐D3/def2‐QZVPP‐SDD(Ni)‐SMD‐(DMA)//(U)B3LYP‐D3(BJ)/def2‐SVP‐SDD(Ni)‐SMD‐(DMA) level of theory.

Then the free energy profile of the most feasible catalytic cycle starting from Ni(I) complex **B1** was calculated (Scheme 5, for energies of all intermediates and transition states, see Table S24). Following the reaction of **B1** (bpy)Ni^I^Cl with **2a** to generate **2ar** and **A1**, radical **2ar** reacted with alkene **1a** by radical addition to form carbon radical **R1** with an energy barrier of 12.1 kcal/mol via **TS1a‐R1**. After the initial steps, there were two possible paths leading to the product. In path 1, (bpy)Ni^II^Cl_2_ **A1** recombined with carbon radical **R1** to afford (bpy)Ni^III^(R)Cl_2_ **D2** [R = ‐CH(CH_2_CF_2_CONEt_2_)(NHCOPh)] via **TS^3^A1‐^2^D2** with a low energy barrier of 5.5 kcal/mol, then **D2** was reduced by zinc to (bpy)Ni^I^(R) **B2** with an energy release of 49.4 kcal/mol. Later, **B2** reacted with nitrogen electrophile **3a** by oxidative addition into N─O bond to give **D3** with a very high energy barrier of 62.6 kcal/mol via **TS^2^B2‐^2^D3**, which was kinetically unfavorable. The Ni(III) complex **D3** would undergo reductive elimination to afford product **4a,** and (bpy)Ni^I^(OOCPh) **B3**, yet the corresponding transition state could not be located. Finally, **B3** underwent ligand exchange with MgCl_2_ to regenerate **B1** with an energy release of 8.7 kcal/mol. In comparison, path 2 was more favorable energetically. In path 2, **A1** was first reduced by zinc to **C** with energy release of 6.9 kcal/mol, followed by the formation of complex **CP4** through binding between **C** and **3a**, which was energetically downhill by 28.1 kcal/mol. **CP4** underwent oxidative addition into N─O bond to form amido Ni(II) complex **A2** via **TS^3^C‐^3^A2** with an energy barrier of 15.9 kcal/mol. **A2** then reacted with carbon radical **R1** through a bimolecular homolytic substitution (S_H_2) process via **TS^3^A2‐^2^B3** with an energy barrier of 10.0 kcal/mol to produce **B3** and product **4a**, releasing 48.9 kcal/mol of free energy. The possibility where **A2** recombined with radical **R1** to form **D3** was also explored, though the process was downhill in thermodynamics with 4.1 kcal/mol, the corresponding transition state could not be located. Based on the computational and experimental results, path 2 was more reasonable because the highest energy barrier was less than the apparent experimental activation energy (16.2 kcal/mol).

**SCHEME 6:**
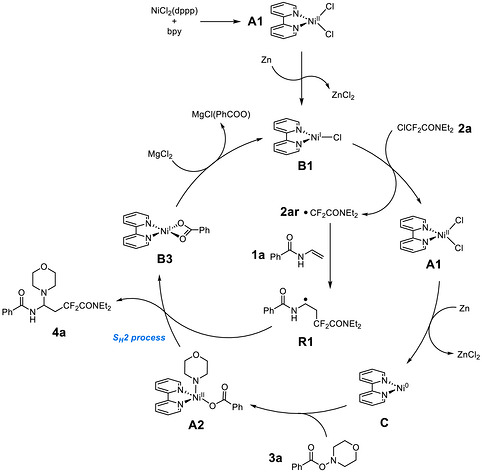
Proposed catalytic cycle.

In general, by integrating the experimental and computational results, the reaction mechanism was possibly involving a Ni(I)/Ni(0)/Ni(II) catalytic cycle (Scheme 6). In this cycle, Ni(II) precatalyst (bpy)Ni^II^Cl_2_ **A1** was first reduced by zinc to Ni(I) complex (bpy)Ni^I^Cl **B1,** which subsequently reacted with chlorodifluoroacetamide **2a** to generate (bpy)Ni^II^Cl_2_ **A1** and a difluoroacetamidyl radical **2ar**. Radical **2ar** then reacted with alkene **1a** by radical addition to give a carbon radical **R1**. Meanwhile, the (bpy)Ni^II^Cl_2_ **A1** was reduced to Ni(0) complex (bpy)Ni°**C** which reacted with nitrogen electrophiles **3a** by oxidative addition to give amido Ni(II) complex (bpy)Ni^II^(morpholino)(O_2_CPh) **A2**. Finally, the carbon radical **R1** would react with amido Ni(II) complex **A2** through an S_H_2 C─N bond‐forming process to afford products **4a** and Ni(I) complex (bpy)NiI(O_2_CPh) **B3**. **B3** underwent ligand exchange with MgCl_2_ to give (bpy)Ni^I^Cl **B1**, thus finishing the cycle. In this mechanism, a bimolecular homolytic substitution (S_H_2) mechanism for C(sp^3^)‐N bond forming process at the nickel center was revealed [53, 54, 55, 56, 57, 58, 59], which was energetically favorable from a computational view.

### 2.5 Pharmacological Tests

**FIGURE 3:**
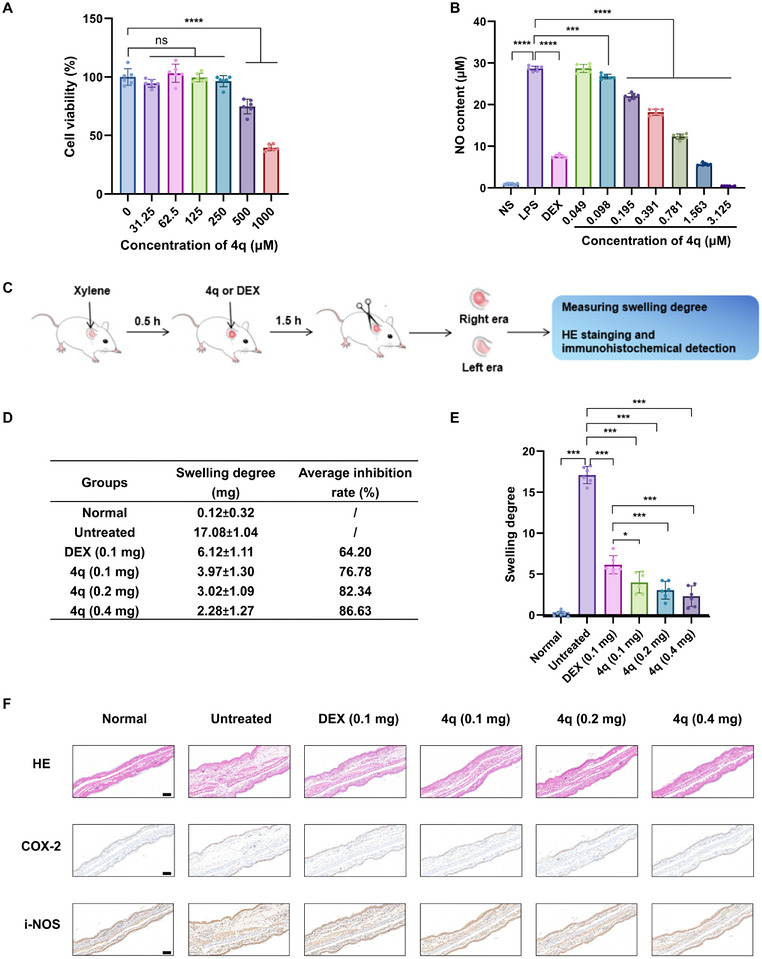
Pharmacological evaluation of compound **4q**. (A) Effects of compound **4q** on the viability of RAW264.7 cells. Data are expressed as mean ± SD (*n* = 6). (B) Concentration‐dependent inhibition of LPS‐induced NO production in RAW264.7 macrophages following treatment with **4q**. Data are presented as mean ± SD (*n* = 6). (C) Schematic representation of in vivo study of **4q** anti‐inflammatory drugs in mice. (D) Mean auricular swelling and inhibition rate following treatment with compound **4q** in the xylene‐induced inflammation model (*n* = 6). (E) Comparative analysis of ear swelling degree among normal control, untreated, and treatment groups. Data are presented as mean ± SD (*n* = 6). (F) Representative H&E staining and immunohistochemical detection of iNOS and COX‐2 expression of right ear tissue sections from indicated experimental groups. Scale bars: 100 µm. Images are representative of six independent biological replicates. ^*^ *p* < 0.05, ^***^ *p* < 0.001, ^****^ *p* < 0.0001, and ns, not significant.

Since the products α‐amidyl‐β‐difluoroalkyl amine possess the pharmacophore that is frequently occurring in anti‐inflammatory agents (such as benzamide group in gentisuric acid and balsalazide) [60, 61, 62], their anti‐inflammatory activities were explored and tested [63, 64]. The pharmacological test revealed that these compounds possess moderate to good anti‐inflammatory activity, in which difluorinated amino acid analogue **4q** showed the best result (Figure 3). Firstly, the effect of **4q** on cell viability was tested using RAW264.7 cells via CCK‐8 assays. The result showed the cell viability was not affected even when the concentration of **4q** reached 250 µM, and only the concentration reached as high as 500 µM the cell viability would decrease. The IC_50_ of **4q** of the inhibition rate on cell viability was 577.2 µM, demonstrating that **4q** has good safety (Figure 3A and Figure S15). Subsequently, the anti‐inflammatory activity was evaluated through nitric oxide (NO) levels with dexamethasone (DEX, 10 µM) as a positive control. The effects of different concentrations of **4q** on the production of NO in LPS‐induced RAW264.7 cells were tested. Compared with LPS group, the production of NO in RAW264.7 macrophages decreased in both the DEX and **4q** group. Specifically, compound **4q** could significantly reduce the production of NO with an IC_50_ = 0.54 µM, and its inhibitory ability increased with increasing concentration (Figure 3B and Figure S16). The inhibition rate of **4q** at 1.563 µM against NO production was better than that of DEX at a concentration of 10 µM. More remarkably, the selectivity index of **4q** is as high as 1069 (the ratio of IC_50_ of cytotoxicity and the IC_50_ of anti‐inflammatory activity). The above results indicated that **4q** could effectively inhibit LPS‐induced macrophage inflammatory response without cytotoxicity, showing good potential in the development of anti‐inflammatory drugs.

To further assess its pharmacological potential, we investigated the therapeutic efficacy of compound **4q** in vivo using a xylene‐induced murine model of auricular inflammation (Figure 3C). Xylene can provoke acute, non‐specific localized inflammatory responses and vascular alterations, rendering it a well‐established model for screening anti‐inflammatory candidates. Thirty minutes after topical application of xylene, compound **4q** was administered at three dosage levels (0.1, 0.2, and 0.4 mg per ear) and dexamethasone (0.1 mg per ear) was used as a positive control. After a 1.5‐h treatment interval, punch biopsies were collected from the ears and quantitatively assessed by gravimetric analysis. Our results showed that **4q** significantly attenuated xylene‐induced auricular inflammation and associated edematous changes (Figure 3D,E, Table S23 and Figure S17). Compared with the untreated control group, which showed a mean ear weight gain of 17.08 mg, treatment with **4q** at 0.1 mg reduced swelling to 3.97 mg, whereas DEX‐treated mice demonstrated swelling of 6.12 mg at an equivalent dosage. Notably, at identical concentrations, compound **4q** achieved a swelling inhibition rate of 76.78%, compared to 64.20% for DEX. Furthermore, **4q** elicited dose‐dependent anti‐inflammatory effects, with inhibition rates of 82.34% and 86.63% at 0.2 mg and 0.4 mg per ear, respectively (Figure 3D)

Histological examination via hematoxylin and eosin (H&E) staining corroborated these quantitative observations. Auricular tissue from normal control mice displayed well‐preserved epidermal and dermal architecture, devoid of inflammatory infiltrates. In contrast, specimens from the untreated group exhibited characteristic inflammatory pathology, including pronounced epidermal hyperplasia, dermal edema, vascular dilatation, and dense leukocytic infiltration predominantly comprising neutrophils and monocytes (Figure 3F and Figure S18). Treatment with either compound **4q** or DEX markedly ameliorated these histopathological alterations. Notably, **4q** demonstrated superior anti‐inflammatory activity, preserving tissue cytoarchitecture, attenuating inflammatory cell accumulation, and reducing interstitial edema. To elucidate the molecular mechanisms underlying the anti‐inflammatory actions of compound **4q**, we performed immunohistochemical analysis targeting two pivotal pro‐inflammatory enzymes: inducible nitric oxide synthase (iNOS) and cyclooxygenase‐2 (COX‐2) (Figure 3F). Basal expression of both markers was minimal in normal control tissues, consistent with a quiescent, noninflamed state. Following xylene challenge, however, untreated specimens displayed robust upregulation of iNOS and COX‐2, evidenced by intense brown‐yellow immunoreactivity localized predominantly within the dermal compartment. This xylene‐induced overexpression was substantially attenuated in tissues from animals treated with compound **4q**, indicating effective suppression of NO and prostaglandin‐mediated inflammatory cascades. Collectively, these findings establish **4q** as a promising anti‐inflammatory compound with potent in vivo activity, warranting further preclinical development and pharmacological characterization.

## 3 Conclusions

In summary, we have developed an efficient nickel‐catalyzed three‐component reductive fluoroalkylation‐amination strategy of alkenes for the direct synthesis of α‐amidyl‐β‐difluoroalkyl amines through construction of C─C and C(sp^3^)─N bond in a single step. By applying this reaction, a broad array of difluoroalkyl chlorides and electrophilic aminating reagents, including a number of amino acid‐derived and drug‐derived reagents, can be smoothly assembled into N‐vinylbenzamides with high efficiency, mild reaction conditions, good functional group tolerance, and synthetic convenience. Mechanistic experiments and DFT calculations suggested the involvement of an S_H_2‐type C‐N bond‐forming process, which provides an inspiration for the development of a new mode for C(sp^3^)‐N construction. The significance of this protocol is further underscored by the potent anti‐inflammatory activity and high selectivity index of the resulting α‐amidyl‐β‐difluoroalkyl amines. This result highlights that the synthetic method provides a novel avenue for drug discovery and development through the integration of β‐difluoroalkyl amine and geminal diamine structural motif that are both pharmacophoricly vital in medicinal chemistry. In‐depth and ongoing research in our laboratory intends to broaden the scope of this approach and increase the structural diversity of the products for further structural optimization and SAR studies in anti‐inflammatory drug development.

## Author Contributions

**Fan Ding**: methodology, validation, investigation, Writing – review and editing, data curation. **Luyan Wang**: writing – review and editing, validation. **Dandan Liu**: conceptualization, funding acquisition, project administration, supervision, resources, writing – original draft, writing – review and editing. **Chang Xu**: conceptualization, visualization, funding acquisition, writing – original draft, writing – review and editing, project administration, supervision. **Yunxi Li**: investigation, validation. **Yunzhi He**: investigation, validation. **Xingang Zhang**: writing – review and editing, conceptualization. **Siyu He**: methodology, data curation, investigation, validation, writing – original draft, writing – review and editing, funding acquisition. **Fangting Ma**: methodology, data curation, investigation, validation, formal analysis, writing – review and editing.

## Conflicts of Interest

The authors declare no conflicts of interest.

## Supporting information




**Supporting File**: advs76993‐sup‐0001‐SuppMat.docx.

## Data Availability

The data that support the findings of this study are available in the supporting information of this article.
