# Supplementary material for: Nickel‐Catalyzed Three‐Component Difluoroalkylation‐Amination for the Direct Access to Anti‐Inflammatory β‐Difluoroalkyl Amines
Source: Adv Sci (Weinh). 2026 Aug 3:e76993. Online ahead of print. doi: 10.1002/advs.76993 (PMC13430932; doi:10.1002/advs.76993)
Supplement: Supplementary file 1 — Supporting File: advs76993‐sup‐0001‐SuppMat.docx. [file ADVS-9999-e76993-s001.docx]

**Nickel-Catalyzed Three-Component Difluoroalkylation-Amination for the Direct Access to Anti-inflammatory β-Difluoroalkyl Amines**

Chang Xu^1^, Fangting Ma^1^, Fan Ding^1,2^, Luyan Wang^1^, Yunxi Li^1^, Yunzhi He^1^ Xingang Zhang^3^, Siyu He^*1^, and Dandan Liu^*1,2^

^1^School of Pharmaceutical Sciences and Yunnan Key Laboratory of Pharmacology for Natural Products, Kunming Medical University, Kunming 650500, China

^2^Yunnan Institute for Advanced Studies, Ministry of Education, Kunming, China

^3^State Key Laboratory of Fluorine and Nitrogen Chemistry and Advanced Materials, Shanghai Institute of Organic Chemistry, University of Chinese Academy of Sciences, Chinese Academy of Sciences, 345 Lingling Road, Shanghai 200032, China

*Corresponding author. Email: liudandan@kmmu.edu.cn; hesiyu@kmmu.edu.cn

**Supplementary Information**

**Table of Contents**

[1. Materials and methods 2](#_Toc222493079)

[2. General procedure for the preparation of α-chloro-α,α-difluoroacetamides **2** and α-chloro-α-monofluoroacetamides **2t** from ethyl chlorodi(mono)fluoroacetate 3](#_Toc222493080)

[3. General procedure for the preparation of *N*-benzoyloxyamines **3** 10](#_Toc222493081)

[4. Optimization of nickel catalyzed reductive fluoroalkylation-amidation of enamide **1a** with *N*, *N*-diethylchlorodifluoroacetamide **2a** and 4-benzoyloxymorpholine **3a**. 12](#_Toc222493082)

[5. General procedure for nickel catalyzed reductive fluoroalkylation-animation of enamides **1** with fluoroalkyl chlorides **2** and *N*-benzoyloxyamines **3**. 17](#_Toc222493083)

[6. Characterization Data for Compounds **4**-**8** 18](#_Toc222493084)

[7. Synthetic applications 44](#_Toc222493085)

[8. Mechanistic Studies 48](#_Toc222493086)

[9. Pharmacological tests 64](#_Toc222493087)

[10. DFT calculations 70](#_Toc222493088)

[11. Supplementary Reference 98](#_Toc222493089)

[12. Characterization Spectra for compounds **1**-**3**. 101](#_Toc222493090)

[13. Characterization Spectra for compounds **4**-**12**, **14** and **16** 128](#_Toc222493091)

# Materials and methods

**General Information:** ^1^H NMR and ^13^C NMR spectra were recorded on a Bruker Avance Neo 600M spectrometer and are calibrated using residual undeuterated solvent (CDCl_3_ at 7.26 ppm ^1^H NMR, 77.16 ppm ^13^C NMR; CD_3_OD at 3.31 ppm ^1^H NMR, 49.00 ppm ^13^C NMR; DMSO-*d*_6_ at 2.50 ppm ^1^H NMR, 39.52 ppm ^13^C NMR; acetone-*d_6_* at 2.05 ppm ^1^H NMR, 30.60 (CH_3_) ppm ^13^C NMR). ^19^F NMR was recorded on a Bruker Avance Neo 600M spectrometer (CFCl_3_ as an external standard and low field is positive). Chemical shifts (δ) are reported in ppm, and coupling constants (*J*) are in Hertz (Hz). The following abbreviations were used to explain the multiplicities: s = singlet, d = doublet, t = triplet, q = quartet, m = multiplet, br = broad. NMR yield was determined by ^19^F NMR using fluorobenzene as an internal standard before working up the reaction. Mass spectra were recorded on a Bruker ESI-Q-TOF MS/MS. Melting points were determined by SGW X-5 melting-point apparatus of Shanghai INESA Physico-Optical Instrument Co.,Ltd.

**Materials:** All reagents were used as received from commercial sources and used without further purification. Superdry solvents, DMF, DMA, NMP, THF, 1,4-dioxane and MeCN were purchased form Adamas chemicals. Ni(dppp)Cl_2_ and ligands were purchased from Adamas chemicals and used as received. The enamides **1a**-**1d** were prepared according to the literature [1]. Emamides **1e** was purchased from Adamas chemicals.

**Figure S1.** Enamides **1**

**2,3,4,5,6-pentafluoro-N-vinylbenzamide (1d).** The product (20 mmol scale, 2.6 g, 55% yield) was purified with silica gel chromatography (Petroleum ether/Ethyl Acetate = 10/1) as a white solid (m.p. 97.9 – 99.0 ℃) . ^1^H NMR (600 MHz, CD_3_OD) δ 7.06 – 7.00 (m, 1H), 4.89 (d, *J* = 15.9 Hz, 1H), 4.63 (d, *J* = 8.8 Hz, 1H). ^19^F NMR (565 MHz, CD_3_OD) δ -143.53 – -143.72 (m, 2F), -154.48 – -154.63 (m, 1F), -163.42 – -163.76 (m, 2F). ^13^C NMR (151 MHz, CD_3_OD) δ 156.9, 146.3 – 146.0 (m), 144.7 – 144.4 (m), 142.9 – 142.7 (m), 140.0 – 139.6 (m), 138.3 – 138.0 (m), 129.2, 113.1 – 112.7 (m), 99.4. MS (ESI): m/z (%) 497.0 (100, [2M+Na]^+^), 260.0 ([M+Na]^+^). HRMS (ESI): Calculated for C_9_H_4_F_5_NONa ([M+Na]^+^): 260.0105; Found: 260.0105.

# General procedure for the preparation of α-chloro-α,α-difluoroacetamides 2 and α-chloro-α-monofluoroacetamides 2t from ethyl chlorodi(mono)fluoroacetate

**Figure S2.** Fluoroalkyl substrates **2**

The general procedure for the preparation of α-chloro-α,α-difluoroacetamides and α-chloro-α-monofluoroacetamides **2** is according to the literature. [2] Typical procedure for the preparation of **2q** is as follows: to a dry 100 mL Schlenk flask is added L-methionine methyl ester hydrochloride (24 mmol, 1.2 equiv.), and the flask was evacuated and backfilled with argon for 3 times. Then DCM (20 mL), ethyl chlorodifluoroacetate (20 mmol, 1 equiv.) and trimethylamine (35 mmol, 1.5 equiv.) were added under argon. The reaction was stirred at room temperature overnight. The reaction mixture was diluted with DCM, washed with water, 1M HCl (25 mL) and brine. The organic layer was dried over Na_2_SO_4_, filtered and concentrated. The residue was purified with silica gel chromatography (Petroleum ether /Ethyl Acetate = 5/1) to give the corresponding 1-chloro-1,1-difluoroacetamide **2q** as a colorless oil. **2a**, **2d**, **2e**, **2k**, **2s**, **2t** are known compounds [2–4].

***N*, *N*-dibutyl-2-chloro-2,2-difluoroacetamide (2b).** The product (20 mmol scale, 3.6 g, 75% yield) was purified with silica gel chromatography (Petroleum ether/Ethyl Acetate = 10/1) as a colorless oil. ^1^H NMR (600 MHz, CDCl_3_) δ 3.37 (dt, *J* = 15.6, 7.8 Hz, 4H), 1.63 – 1.52 (m, 4H), 1.38 – 1.24 (m, 4H), 1.00 – 0.87 (m, 6H). ^19^F NMR (565 MHz, CDCl_3_) δ -57.4 (2F). ^13^C NMR (151 MHz, CDCl_3_) δ 158.7 (t, *J* = 28.8 Hz), 119.1 (t, *J* = 301.5 Hz), 48.1 (t, *J* = 3.5 Hz), 47.3, 30.9, 29.0, 20.2, 20.0, 13.9, 13.8. MS (ESI): m/z (%) 505.2 (100, [2M+ Na]^+^), 242.1 ([M+ H]^+^). HRMS (ESI): Calculated for C_10_H_19_ClF_2_NO ([M+ H]^+^): 242.1118; Found: 242.1118.

**1-(Azetidin-1-yl)-2-chloro-2,2-difluoroethan-1-one (2c).** The product (20 mmol scale, 2.0 g, 59% yield) was purified with silica gel chromatography (Petroleum ether/Ethyl Acetate = 10/1) as a yellow oil. ^1^H NMR (600 MHz, CDCl_3_) δ 4.43 (t, *J* = 7.7 Hz, 2H), 4.17 (s, 2H), 2.41 (p, *J* = 7.9 Hz, 2H). ^19^F NMR (565 MHz, CDCl_3_) δ -61.5 (t, *J* = 9.7 Hz, 1F), -64.5 (td, *J* = 7.6, 4.6 Hz, 1F). ^13^C NMR (151 MHz, CDCl_3_) δ 158.2 (t, *J* = 30.7 Hz), 118.8 (t, *J* = 302.5 Hz), 52.7 (t, *J* = 3.1 Hz), 49.4, 16.2. MS (ESI): m/z (%) 361.0 (100, [2M+ Na]^+^), 170.0 ([M+H]^+^). HRMS (ESI): Calculated for C_5_H_7_ClF_2_NO ([M+H]^+^): 170.0179; Found: 170.0179.

**2-Chloro-2,2-difluoro-1-thiomorpholinoethan-1-one** **(2f).** The product (20 mmol scale, 3.9 g, 91% yield) was purified with silica gel chromatography (Petroleum ether/Ethyl Acetate = 5/1) as a colorless oil. ^1^H NMR (600 MHz, CDCl_3_) δ 3.96 – 3.87 (m, 4H), 2.69 (q, *J* = 4.7 Hz, 4H). ^19^F NMR (565 MHz, CDCl_3_) δ -57.4 (2F). ^13^C NMR (151 MHz, CDCl_3_) δ 157.6 (t, *J* = 29.2 Hz), 118.6 (t, *J* = 301.0 Hz), 49.7 – 48.6 (m), 46.7, 27.8, 27.3. MS (ESI): m/z (%) 453.0 (100, [2M+ Na]^+^), 238.0 ([M+Na]^+^). HRMS (ESI): Calculated for C_6_H_8_ClF_2_NOSNa ([M+Na]^+^): 237.9875; Found: 237.9875.

**2-chloro-1-(6,7-dimethoxy-3,4-dihydroisoquinolin-2(1H)-yl)-2,2-difluoroethan-1-one (2g).** The product (20 mmol scale, 2.8 g, 46% yield) was purified with silica gel chromatography (Petroleum ether/Ethyl Acetate = 5/1) as a white solid (m.p. 104.6 – 105.2 ℃) . ^1^H NMR (600 MHz, CDCl_3_) δ 6.65 – 6.57 (m, 2H), 4.72 (d, *J* = 17.0 Hz, 2H), 3.92 – 3.82 (m, 8H), 2.87 (dt, *J* = 13.9, 5.7 Hz, 2H). ^19^F NMR (565 MHz, CDCl_3_) δ -57.91 (d, *J* = 93.2 Hz, 2F). ^13^C NMR (151 MHz, CDCl_3_) δ 158.2 – 157.6 (m), 148.7 – 147.8 (m), 125.6 (d, *J* = 121.7 Hz), 123.4 (d, *J* = 31.7 Hz), 118.8 (t, *J* = 300.9 Hz), 111.5 (d, *J* = 42.6 Hz), 109.0 (d, *J* = 62.1 Hz), 56.9 – 55.4 (m), 47.6 (t, *J* = 4.4 Hz), 45.8, 44.1 (t, *J* = 3.9 Hz), 42.4, 28.1 (d, *J* = 174.2 Hz). MS (ESI): m/z (%) 633.1 (100, [2M+Na]^+^), 328.1 ([M+ Na]^+^). HRMS (ESI): Calculated for C_13_H_14_ClF_2_NO_3_Na ([M+ Na]^+^): 328.0522; Found: 328.0522.

**1-(Azepan-1-yl)-2-chloro-2,2-difluoroethan-1-one (2h).** The product (20 mmol scale, 4.0 g, 95% yield) was purified with silica gel chromatography (Petroleum ether/Ethyl Acetate = 10/1) as a colorless oil. ^1^H NMR (600 MHz, CDCl_3_) δ 3.66 – 3.60 (m, 2H), 3.58 – 3.52 (m, 2H), 1.81 – 1.77 (m, 2H), 1.77 – 1.72 (m, 2H), 1.59 (dt, *J* = 5.9, 2.9 Hz, 4H). ^19^F NMR (565 MHz, CDCl_3_) δ -57.2 (2F). ^13^C NMR (151 MHz, CDCl_3_) δ 158.8 (t, *J* = 28.9 Hz), 119.1 (t, *J* = 301.4 Hz), 48.6, 48.4 (t, *J* = 3.7 Hz), 29.3, 27.6, 26.4, 25.9. MS (ESI): m/z (%) 445.1 (100, [2M+Na]^+^), 234.1 ([M+ Na]^+^). HRMS (ESI): Calculated for C_8_H_12_ClF_2_NONa ([M+Na]^+^): 234.0468; Found: 234.0468.

***N*-(Tert-butyl)-2-chloro-2,2-difluoroacetamide (2i).** The product (20 mmol scale, 2.9 g, 78% yield) was purified with silica gel chromatography (Petroleum ether/Ethyl Acetate = 5/1) as a white solid (m.p. 56.0 – 56.9 ℃) . ^1^H NMR (600 MHz, CDCl_3_) δ 6.02 (s, 1H), 1.41 (d, *J* = 1.0 Hz, 9H). ^19^F NMR (565 MHz, CDCl_3_) δ -64.1 (2F). ^13^C NMR (151 MHz, CDCl_3_) δ 158.3 (t, *J* = 29.1 Hz), 119.2 (t, *J* = 303.9 Hz), 52.9, 28.3. MS (ESI): m/z (%) 393.1 (100, [2M+ Na]^+^), 208.0 ([M+ Na]^+^). HRMS (ESI): Calculated for C_6_H_10_ClF_2_NONa ([M+ Na]^+^): 208.0311; Found: 208.0311.

**2-Chloro-*N*-cyclohexyl-2,2-difluoroacetamide (2j).** The product (20 mmol scale, 3.1 g, 73% yield) was purified with silica gel chromatography (Petroleum ether/Ethyl Acetate = 8/1) as a white solid (m.p. 85.2 – 85.7 ℃) . ^1^H NMR (600 MHz, CDCl_3_) δ 6.12 (s, 1H), 3.95 – 3.65 (m, 1H), 2.00 – 1.93 (m, 2H), 1.75 (dt, *J* = 13.6, 3.6 Hz, 2H), 1.64 (dt, *J* = 13.1, 3.8 Hz, 1H), 1.43 – 1.33 (m, 2H), 1.32 – 1.13 (m, 3H). ^19^F NMR (565 MHz, CDCl_3_) δ -64.8 (2F). ^13^C NMR (151 MHz, CDCl_3_) δ 158.5 (t, *J* = 29.7 Hz), 119.3 (t, *J* = 302.7 Hz), 49.6, 32.5, 25.4, 24.7. MS (ESI): m/z (%) 445.1 (100, [2M+ Na]^+^), 234.1 ([M+ Na]^+^). HRMS (ESI): Calculated for C_8_H_12_ClF_2_NONa ([M+ Na]^+^): 234.0468; Found: 234.0468.

**2-Chloro-*N*-(2-cyanoethyl)-2,2-difluoroacetamide (2l).** The product (20 mmol scale, 2.56 g, 70% yield) was purified with silica gel chromatography (Petroleum ether/Ethyl Acetate = 10/1) as a white solid (m.p. 51.3 – 52.3 ℃) . ^1^H NMR (600 MHz, CDCl_3_) δ 7.19 (s, 1H), 3.63 (q, *J* = 6.3 Hz, 2H), 2.72 (t, *J* = 6.3 Hz, 2H). ^19^F NMR (565 MHz, CDCl_3_) δ -64.5 (2F). ^13^C NMR (151 MHz, CDCl_3_) δ 160.2 (t, *J* = 31.1 Hz), 118.8 (t, *J* = 301.9 Hz), 117.4, 36.2, 17.9. MS (ESI): m/z (%) 205.0 ([M+ Na]^+^). HRMS (ESI): Calculated for C_5_H_5_ClF_2_N_2_ONa ([M+ Na]^+^): 204.9951; Found: 204.9951.

**Methyl 4-(2-chloro-2,2-difluoroacetamido) butanoate (2m).** The product (20 mmol scale, 4.2 g, 91% yield) was purified with silica gel chromatography (Petroleum ether/Ethyl Acetate = 5/1) as a yellow oil. ^1^H NMR (600 MHz, CDCl_3_) δ 6.93 (s, 1H), 3.69 (d, *J* = 1.5 Hz, 3H), 3.44 – 3.39 (m, 2H), 2.43 (t, *J* = 6.8 Hz, 2H), 1.96 – 1.90 (m, 2H). ^19^F NMR (565 MHz, CDCl_3_) δ -64.2 (2F). ^13^C NMR (151 MHz, CDCl_3_) δ 174.1, 159.7 (t, *J* = 29.9 Hz), 119.2 (t, *J* = 302.4 Hz), 52.1, 40.0, 31.6, 23.8. MS (ESI): m/z (%) 481.1 (100, [2M+ Na]^+^), 252.0 ([M+ Na]^+^). HRMS (ESI): Calculated for C_7_H_10_ClF_2_NO_3_Na ([M+ Na]^+^): 252.0209; Found: 252.0209.

**Tert-butyl (4-(2-chloro-2,2-difluoroacetamido)butyl)carbamate (2n).** The product (20 mmol scale, 2.8 g, 47% yield) was purified with silica gel chromatography (Petroleum ether/Ethyl Acetate = 8/1) as a white solid (m.p. 81.5 – 82.4 ℃) . ^1^H NMR (600 MHz, CDCl_3_) δ 6.98 (s, 1H), 4.66 (s, 1H), 3.42 – 3.36 (m, 2H), 3.13 (s, 2H), 1.65 – 1.58 (m, 2H), 1.57 – 1.51 (m, 2H), 1.43 (t, *J* = 3.7 Hz, 9H). ^19^F NMR (565 MHz, CDCl_3_) δ -64.1 (2F). ^13^C NMR (151 MHz, CDCl_3_) δ 159.7 (t, *J* = 29.8 Hz), 156.4, 119.3 (t, *J* = 302.5 Hz), 79.7, 39.9, 28.5, 27.7, 26.0. MS (ESI): m/z (%) 623.2 (100, [2M+Na]^+^), 323.1 ([M+Na]^+^). HRMS (ESI): Calculated for C_11_H_19_ClF_2_N_2_O_3_Na ([M+ Na]^+^): 323.0944; Found: 323.0944.

**Methyl *N*^6^-(tert-butoxycarbonyl)-*N*^2^-(2-chloro-2,2-difluoroacetyl)-L-lysinate (2o).** The product (20 mmol scale, 4.5 g, 60% yield) was purified with silica gel chromatography (Petroleum ether/Ethyl Acetate = 10/1) as a white solid (m.p. 61.2 – 62.2 ℃) . ^1^H NMR (600 MHz, CDCl_3_) δ 4.55 – 4.50 (m, 1H), 3.73 (s, 3H), 3.08 – 3.01 (m, 2H), 1.95 – 1.86 (m, 1H), 1.81 – 1.71 (m, 1H), 1.51 – 1.42 (m, 2H), 1.38 (s, 9H), 1.35 – 1.26 (m, 2H). ^19^F NMR (565 MHz, CDCl_3_) δ -64.3 (d, *J* = 3.3 Hz, 2F). ^13^C NMR (151 MHz, CDCl_3_) δ 171.5, 159.2 (t, *J* = 30.8 Hz), 156.2, 118.9 (t, *J* = 302.3 Hz), 79.3, 52.9, 40.0, 31.4, 29.6, 28.4, 22.3. MS (ESI): m/z (%) 767.3 (100, [2M+ Na]^+^), 395.1 ([M+ Na]^+^). HRMS (ESI): Calculated for C_14_H_23_ClF_2_N_2_O_5_Na ([M+ Na]^+^): 395.1156; Found: 395.1156.

**Methyl (2-chloro-2,2-difluoroacetyl)-L-valinate (2p).** The product (20 mmol scale, 2.0 g, 41% yield) was purified with silica gel chromatography (Petroleum ether/Ethyl Acetate = 8/1) as a yellow oil. ^1^H NMR (600 MHz, CDCl_3_) δ 6.80 (d, *J* = 8.6 Hz, 1H), 4.55 (dd, *J* = 8.7, 4.8 Hz, 1H), 3.78 (s, 3H), 2.32 – 2.18 (m, 1H), 0.95 (dd, *J* = 18.5, 6.9 Hz, 6H). ^19^F NMR (565 MHz, CDCl_3_) δ -64.3 (d, *J* = 28.0 Hz, 2F). ^13^C NMR (151 MHz, CDCl_3_) δ 171.2, 159.3 (t, *J* = 30.6 Hz), 119.0 (t, *J* = 302.4 Hz), 57.8, 52.8, 31.7, 18.9, 17.7. MS (ESI): m/z (%) 509.1 (100, [2M+ Na]^+^), 266.0 ([M+ Na]^+^). HRMS (ESI): Calculated for C_8_H_12_ClF_2_NO_3_Na ([M+ Na]^+^): 266.0366; Found: 266.0366.

**Methyl (2-chloro-2,2-difluoroacetyl)-L-methioninate (2q).** The product (20 mmol scale, 2.4 g, 44% yield) was purified with silica gel chromatography (Petroleum ether/Ethyl Acetate = 5/1) as a colorless oil. ^1^H NMR (600 MHz, CDCl_3_) δ 7.20 (s, 1H), 4.75 – 4.69 (m, 1H), 3.79 (s, 3H), 2.52 (t, *J* = 7.2 Hz, 2H), 2.28 – 2.19 (m, 1H), 2.09 (s, 4H). ^19^F NMR (565 MHz, CDCl_3_) δ -64.4 (d, *J* = 15.9 Hz, 2F). ^13^C NMR (151 MHz, CDCl_3_) δ 171.0, 159.1 (t, *J* = 30.8 Hz), 118.8 (t, *J* = 302.3 Hz), 53.1, 52.3, 30.8, 29.8, 15.5. MS (ESI): m/z (%) 573.0 (100, [2M+ Na]^+^), 298.1 ([M+ Na]^+^). HRMS (ESI): Calculated for C_8_H_12_ClF_2_NO_3_SNa ([M+ Na]^+^): 298.0087; Found: 298.0087.

**Methyl (2-chloro-2,2-difluoroacetyl)-L-tryptophanate (2r).** The product (20 mmol scale, 3.2 g, 48% yield) was purified with silica gel chromatography (Petroleum ether/Ethyl Acetate = 3/1) as a white solid (m.p. 119.1 – 119.6 ℃) . ^1^H NMR (600 MHz, CDCl_3_) δ 8.11 (s, 1H), 7.51 (dd, *J* = 7.9, 1.2 Hz, 1H), 7.37 (dt, *J* = 8.2, 0.9 Hz, 1H), 7.23 – 7.19 (m, 1H), 7.15 – 7.11 (m, 1H), 7.00 (d, *J* = 2.4 Hz, 1H), 6.82 (d, *J* = 7.5 Hz, 1H), 4.92 (dt, *J* = 7.8, 5.1 Hz, 1H), 3.73 (s, 3H), 3.43 (dd, *J* = 5.2, 2.1 Hz, 2H). ^19^F NMR (565 MHz, CDCl_3_) δ -64.2 (d, *J* = 5.7 Hz, 2F). ^13^C NMR (151 MHz, CDCl_3_) δ 170.8, 158.7 (t, *J* = 30.9 Hz), 136.1, 127.3, 122.9, 122.6, 120.0, 118.4, 111.4, 109.0, 53.6, 52.9, 27.3. MS (ESI): m/z (%) 683.1 (100, [2M+Na]^+^), 353.1 ([M+Na]^+^). HRMS (ESI): Calculated for C_14_H_13_ClF_2_N_2_O_3_Na ([M+ Na]^+^): 353.0475; Found: 353.0475.

**Methyl 4-(2-chloro-2,2-difluoroacetamido)-3-(4-chlorophenyl) butanoate (2u).** The product (16 mmol scale, 3.5 g, 65% yield) was purified with silica gel chromatography (Petroleum ether/Ethyl Acetate = 10/1) as a white solid (m.p. 66.7 – 67.6 ℃) . ^1^H NMR (600 MHz, CDCl_3_) δ 7.33 – 7.29 (m, 2H), 7.14 (d, *J* = 8.4 Hz, 2H), 6.54 (d, *J* = 32.1 Hz, 1H), 3.67 (dd, *J* = 13.4, 6.6 Hz, 1H), 3.63 (d, *J* = 4.5 Hz, 3H), 3.50 (dt, *J* = 13.4, 6.6 Hz, 1H), 3.42 (p, *J* = 7.2 Hz, 1H), 2.73 – 2.64 (m, 2H). ^19^F NMR (565 MHz, CDCl_3_) δ -64.3 (2F). ^13^C NMR (151 MHz, CDCl_3_) δ 172.2, 159.8 – 159.4 (m), 138.8 (d, *J* = 2.0 Hz), 133.6 (d, *J* = 4.7 Hz), 129.4 (d, *J* = 3.3 Hz), 128.9, 119.0 (t, *J* = 302.3 Hz), 52.2 (d, *J* = 1.4 Hz), 45.0, 40.8, 38.2. MS (ESI): m/z (%) 703.0 (100, [2M+Na]^+^), 362.0 ([M+Na]^+^). HRMS (ESI): Calculated for C_13_H_13_Cl_2_F_2_NO_3_Na ([M+ Na]^+^): 362.0133; Found: 362.0133.

**Methyl (S)-3-((2-chloro-2,2-difluoroacetamido) methyl)-5-methylhexanoate (2v).** The product (20 mmol scale, 5.6 g, 98% yield) was purified with silica gel chromatography (Petroleum ether/Ethyl Acetate = 10/1) as a yellow oil. ^1^H NMR (600 MHz, CDCl_3_) δ 7.16 (s, 1H), 3.69 – 3.65 (m, 3H), 3.40 (dt, *J* = 13.3, 4.9 Hz, 1H), 3.31 – 3.18 (m, 1H), 2.48 – 2.34 (m, 1H), 2.28 (dd, *J* = 15.9, 7.9 Hz, 1H), 2.23 – 2.13 (m, 1H), 1.70 – 1.56 (m, 1H), 1.21 – 1.11 (m, 2H), 0.89 (dd, *J* = 6.5, 1.9 Hz, 3H), 0.87 (dd, *J* = 6.7, 1.7 Hz, 3H). ^19^F NMR (565 MHz, CDCl_3_) δ -64.1 (2F). ^13^C NMR (151 MHz, CDCl_3_) δ 174.0, 159.7 (t, *J* = 30.0 Hz), 119.3 (t, *J* = 302.4 Hz), 52.0, 44.4, 41.7, 37.6, 32.7, 25.3, 22.7, 22.6. MS (ESI): m/z (%) 593.2 (100, [2M+Na]^+^), 308.1 ([M+Na]^+^). HRMS (ESI): Calculated for C_11_H_18_ClF_2_NO_3_Na ([M+ Na]^+^): 308.0835; Found: 308.0835.

# General procedure for the preparation of *N*-benzoyloxyamines 3

**Figure S3.** *N*-Benzoyloxyamine substrates **3**

The general procedure for the preparation of *N*-benzoyloxyamines **3** is according to the literature [5]. To a dry 250 mL two-neck flask was added benzoyl peroxide (20 mmol, 1 equiv.) and potassium phosphate dibasic (40 mmol, 2 equiv.), and the flask was evacuated and backfilled with argon for 3 times. Dry DMF (50 mL) was added into the flask and the mixture was cooled to 0 ℃. The amine (30 mmol 1.5 equiv.) was added dropwise and reaction was stirred at room temperature for 2 hours. The reaction was monitored by TLC. After consumption of benzoyl peroxide, the reaction mixture was quenched with water and extracted with DCM, and the organic phase was washed with water, saturated NaHCO_3_ solution, NH_4_Cl solution and brine. The organic layer was dried over Na_2_SO_4_, filtered and concentrated. The residue was purified with silica gel chromatography to give the corresponding *N*-benzoyloxyamine. Known compounds are **3a** [5], **3b** [6], **3c** [7], **3d** [8], **3e** [9], **3f** [10], **3g** [11], **3h** [12], **3i** [13], **3j** [8], **3k** [14], **3l** [15], **3m** [16], **3n** [17], **3p** [18], **3q** [18], **3r** [19].

**4-(2-((2,4-dimethylphenyl)thio)phenyl)piperazin-1-yl benzoate (3o).** The product (6 mmol scale, 1.3 g, 52% yield) was purified with silica gel chromatography (Petroleum ether/Ethyl Acetate = 10/1) as a white solid (m.p. 133.2 – 133.8 ℃) . ^1^H NMR (600 MHz, CDCl_3_) δ 7.16 (s, 2H), 3.69 – 3.65 (m, 1H), 3.40 (dt, *J* = 13.3, 4.9 Hz, 2H), 3.31 – 3.18 (m, 1H), 2.48 – 2.34 (m, 4H), 2.28 (dd, *J* = 15.9, 7.9 Hz, 1H), 2.23 – 2.13 (m, 1H), 1.70 – 1.56 (m, 2H), 1.21 – 1.11 (m, 2H), 0.89 (dd, *J* = 6.5, 1.9 Hz, 4H), 0.87 (dd, *J* = 6.7, 1.7 Hz, 6H). ^13^C NMR (151 MHz, CDCl_3_) δ 164.9, 142.5, 139.5, 136.3, 134.7, 133.3, 131.9, 129.6 (d, *J* = 14.9 Hz), 128.6, 127.9 (d, *J* = 26.4 Hz), 126.7 – 124.9 (m), 120.3, 56.6, 50.0, 21.3, 20.8. MS (ESI): m/z (%) 859.3 (100, [2M+Na]^+^), 441.2 ([M+Na]^+^). HRMS (ESI): Calculated for C_25_H_26_N_2_O_2_SNa ([M+ Na]^+^): 441.1607; Found: 441.1607.

**4-(2-(3-cyano-4-isobutoxyphenyl)-4-methylthiazole-5-carbonyl)piperazin-1-yl benzoate (3s).** The product (2 mmol scale, 0.6 g, 60% yield) was purified with silica gel chromatography (Petroleum ether/Ethyl Acetate = 10/1) as a white solid (m.p. 159.3 – 160.3 ℃) . ^1^H NMR (600 MHz, CDCl_3_) δ 8.12 (s, 1H), 8.05 (d, *J* = 8.8 Hz, 1H), 8.01 (d, *J* = 7.7 Hz, 2H), 7.59 (t, *J* = 7.4 Hz, 1H), 7.46 (t, *J* = 7.5 Hz, 2H), 7.01 (d, *J* = 8.8 Hz, 1H), 3.89 (d, *J* = 6.4 Hz, 2H), 3.55 (s, 4H), 3.01 (s, 2H), 2.52 (s, 3H), 2.27 – 2.16 (m, 1H), 1.78 (s, 2H), 1.09 (d, *J* = 6.7 Hz, 6H). ^13^C NMR (151 MHz, CDCl_3_) δ 165.6, 164.6, 162.5 (d, *J* = 9.7 Hz), 153.5, 133.6, 132.6, 132.0, 129.6, 128.9, 128.7, 125.9, 123.9, 115.6, 112.8, 103.1, 75.8, 56.1, 28.3, 19.2, 16.8. MS (ESI): m/z (%) 235.2 (100). HRMS (ESI): Calculated for C_27_H_28_N_4_O_4_SNa ([M+ Na]^+^): 527.1723; Found: 527.1723.

# Optimization of nickel catalyzed reductive fluoroalkylation-amidation of enamide 1a with *N*, *N*-diethylchlorodifluoroacetamide 2a and 4-benzoyloxymorpholine 3a.

**Table S1.** Ligand effect on Ni-catalyzed reductive fluoroalkylation-amidation of enamide **1a** with chlorodifluoroacetamide **2a** and 4-benzoyloxymorpholine **3a**.*^a^*

| Entry | Ligand | **2a**, remained (%) | **4a,** yield (%)*^b^* | **HCF_2_CONEt_2_,** yield (%)*^b^* |
| --- | --- | --- | --- | --- |
| 1 | **L1** | 1 | 71 | 7 |
| 2 | **L2** | 5 | 64 | 20 |
| 3 | **L3** | 13 | 48 | 5 |
| 4 | **L4** | 17 | 55 | 14 |
| 5 | **L5** | 101 | 7 | 18 |
| 6 | **L6** | 23 | 35 | 10 |
| 7 | **L7** | 24 | 23 | 7 |
| 8 | **L8** | 13 | 26 | 23 |
| 9 | **L9** | 96 | ND | 18 |
| 10 | **L10** | 9 | 71 | 14 |
| 11 | **L11** | 82 | 9 | 19 |
| 12 | **L12** | 36 | 48 | 12 |

*^a^*Reaction conditions (unless otherwise specified): **1a** (0.4 mmol, 1.0 equiv.), **2a** (1.2 equiv.), **3a** (1.2 equiv.) and DMA (3 mL). *^b^*Determined by ^19^F NMR using fluorobenzene (0.4 mmol) as an internal standard. The yield of the products and recovery yield of **2a** are calculated using **1a** as reference substance. ND, not detected.

**Table S2.** Screening of ratio of [Ni]/ligand for Ni-catalyzed reductive fluoroalkylation-amidation of enamide **1a** with chlorodifluoroacetamide **2a** and 4-benzoyloxymorpholine **3a**.*^a^*

| Entry | ***x*** (mol %) | ***y*** (mol %) | **2a**  remained (%) | **4a,** yield (%)*^b^* | **HCF_2_CONEt_2_,** yield (%)*^b^* |
| --- | --- | --- | --- | --- | --- |
| 1 | 5 | 5 | 1 | 71 | 7 |
| 2 | 5 | 7.5 | ND | 79 | 6 |
| 3 | 5 | 10 | 1 | 75 | 7 |
| 4 | 2.5 | 2.5 | 12 | 77 | 8 |
| 5 | 2.5 | 5 | 16 | 79 | 8 |
| 6 | 2.5 | 7.5 | 8 | 78 | 8 |

*^a^*Reaction conditions (unless otherwise specified): **1a** (0.4 mmol, 1.0 equiv.), **2a** (1.2 equiv.), **3a** (1.2 equiv.) and DMA (3 mL). *^b^*Determined by ^19^F NMR using fluorobenzene (0.4 mmol) as an internal standard. The yield of the products and recovery yield of **2a** are calculated using **1a** as reference substance. ND, not detected.

**Table S3.** Screening of nickel catalysts for Ni-catalyzed reductive fluoroalkylation-amidation of enamide **1a** with chlorodifluoroacetamide **2a** and 4-benzoyloxymorpholine **3a**.*^a^*

| Entry | [Ni] | **2a,** remained (%) | **4a,** yield (%)*^b^* | **HCF_2_CONEt_2_,** yield (%)*^b^* |
| --- | --- | --- | --- | --- |
| 1 | NiCl_2_∙DME | 16 | 79 | 8 |
| 2 | NiBr_2_∙DME | 13 | 79 | 8 |
| 3 | NiCl_2_(PPh_3_)_2_ | 17 | 75 | 7 |
| 4 | NiBr_2_(PPh_3_)_2_ | 15 | 78 | 7 |
| 5 | NiCl_2_ | 30 | 66 | 8 |
| 6 | NiBr_2_ | 17 | 67 | 18 |
| 7 | NiI_2_ | 19 | 79 | 8 |
| 8 | Ni(acac)_2_ | 21 | 76 | 8 |
| 9 | NiCl_2_∙6H_2_O | 20 | 72 | 8 |
| 10 | NiBr_2_∙3H_2_O | 46 | 45 | 13 |
| 11 | NiCl_2_(dppe) | 13 | 83 | 8 |
| 12 | NiCl_2_(dppp) | 6 | 87 | 11 |
| 13 | NiCl_2_(dppf) | 20 | 55 | 27 |
| 14 | NiF_2_ | 75 | trace | 45 |
| 15 | NiBr_2_(bpy)_3_ | 14 | 82 | 5 |

*^a^*Reaction conditions (unless otherwise specified): **1a** (0.4 mmol, 1.0 equiv.), **2a** (1.2 equiv.), **3a** (1.2 equiv.) and DMA (3 mL). *^b^*Determined by ^19^F NMR using fluorobenzene (0.4 mmol) as an internal standard. The yield of the products and recovery yield of **2a** are calculated using **1a** as reference substance. ND, not detected.

**Table S4.** Effect of the leaving group of **3a** on Ni-catalyzed reductive fluoroalkylation-amidation of enamide **1a** with chlorodifluoroacetamide **2a** and 4-acyloxymorpholine **3**.*^a^*

| Entry | **3** | **2a**, remained (%) | **4a,** yield (%)*^b^* | **HCF_2_CONEt_2_,** yield (%)*^b^* |
| --- | --- | --- | --- | --- |
| 1 | **3a** | 6 | 87 | 11 |
| 2 | **3aa** | 18 | 69 | 12 |
| 3 | **3ab** | 28 | 62 | 6 |
| 4 | **3ac** | 9 | 77 | 6 |
| 5 | **3ad** | 24 | 18 | 27 |
| 6 | **3ae** | 42 | 48 | 6 |
| 7 | **3af** | 18 | 79 | 5 |
| 8 | **3ag** | 16 | 73 | 6 |

*^a^*Reaction conditions (unless otherwise specified): **1a** (0.4 mmol, 1.0 equiv.), **2a** (1.2 equiv.), **3** (1.2 equiv.) and DMA (3 mL). *^b^*Determined by ^19^F NMR using fluorobenzene (0.4 mmol) as an internal standard. The yield of the products and recovery yield of **2a** are calculated using **1a** as reference substance. ND, not detected.

**Table S5.** Screening of the ratio of **2a**/**3a** for Ni-catalyzed reductive fluoroalkylation-amidation of enamide **1a** with chlorodifluoroacetamide **2a** and 4-benzoyloxymorpholine **3a**.*^a^*

| Entry | equiv. ratio  **1a / 2a / 3a** | **2a**, remained (%) | **4a,** yield (%)*^b^* | **HCF_2_CONEt_2_,** yield (%)*^b^* |
| --- | --- | --- | --- | --- |
| 1 | 1 / 1.2 / 1.2 | 6 | 87 | 11 |
| 2 | 1 / 1.2 / 1.0 | 1 | 78 | 13 |
| 3 | 1 / 1.4 / 1.0 | 15 | 79 | 14 |
| 4 | 1 / 1.5 / 1.0 | 25 | 85 | 14 |
| 5 | 1 / 1.2 / 1.1 | ND | 84 | 13 |
| 6 | 1 / 1.4 / 1.1 | 11 | 91 | 14 |
| 7 | 1 / 1.5 / 1.1 | 25 | 82 | 16 |

*^a^*Reaction conditions (unless otherwise specified): **1a** (0.4 mmol, 1.0 equiv.), **2a** (1.2 – 1.5 equiv.), **3** (1.0 – 1.2 equiv.) and DMA (3 mL). *^b^*Determined by ^19^F NMR using fluorobenzene (0.4 mmol) as an internal standard. The yield of the products and recovery yield of **2a** are calculated using **1a** as reference substance. ND, not detected.

**Table S6.** Screening of the loading amount of Zn and MgCl_2_ for Ni-catalyzed reductive fluoroalkylation-amidation of enamide **1a** with chlorodifluoroacetamide **2a** and 4-benzoyloxymorpholine **3a**.*^a^*

| Entry | Zn (***x*** equiv.) | MgCl_2_ (***y*** equiv.) | **2a**, remained (%) | **4a,** yield (%)*^b^* | **HCF_2_CONEt_2_,** yield (%)*^b^* |
| --- | --- | --- | --- | --- | --- |
| 1 | 2.0 | 1.5 | 11 | 91 | 14 |
| 2 | 1.0 | 1.5 | 105 | 12 | 6 |
| 3 | 1.5 | 1.5 | 29 | 85 | 12 |
| 4 | 1.0 | 1.0 | 96 | 23 | 7 |
| 5 | 1.5 | 1.0 | 63 | 47 | 10 |
| 6 | 2.0 | 1.0 | 57 | 50 | 12 |
| 7 | 2.0 | 2.0 | 7 | 93 | 17 |
| 8 | 2.5 | 2.0 | 2 | 94 | 18 |

*^a^*Reaction conditions (unless otherwise specified): **1a** (0.4 mmol, 1.0 equiv.), **2a** (1.4 equiv.), **3** (1.1 equiv.) and DMA (3 mL). *^b^*Determined by ^19^F NMR using fluorobenzene (0.4 mmol) as an internal standard. The yield of the products and recovery yield of **2a** are calculated using **1a** as reference substance. ND, not detected.

**Table S7.** Screening of solvents for Ni-catalyzed reductive fluoroalkylation-amidation of enamide **1a** with chlorodifluoroacetamide **2a** and 4-benzoyloxymorpholine **3a**.*^a^*

| Entry | Solvent | **2a**, remained (%) | **4a,** yield (%)*^b^* | **HCF_2_CONEt_2_,** yield (%)*^b^* |
| --- | --- | --- | --- | --- |
| 1 | DMA | 2 | 94 (87) | 18 |
| 2 | DMF | ND | 97*^c^* | 21 |
| 3 | 1,4-dioxane | 146 | ND | ND |
| 4 | THF | 121 | ND | 5 |
| 5 | DMSO | 8 | 5 | 29 |
| 6 | MeCN | 132 | ND | ND |

*^a^*Reaction conditions (unless otherwise specified): **1a** (0.4 mmol, 1.0 equiv.), **2a** (1.4 equiv.), **3a** (1.1 equiv.) and DMA (3 mL). *^b^*Determined by ^19^F NMR using fluorobenzene (0.4 mmol) as an internal standard. The yield of the products and recovery yield of **2a** are calculated using **1a** as reference substance, value in parenthesis is isolated yield. ND, not detected. *^c^*More unknown defluorinated byproducts would be produced in DMF than in DMA.

**Table S8.** Control experiments.*^a^*

| Entry | [Ni] | ligand | Zn | MgCl_2_ | **2a**,  remained (%) | **4a,**  yield (%)*^b^* | **HCF_2_CONEt_2_,** yield (%)*^b^* |
| --- | --- | --- | --- | --- | --- | --- | --- |
| 1 | NiCl_2_(dppp) | **L1** | Zn | MgCl_2_ | 2 | 94 | 18 |
| 2 | none | **L1** | Zn | MgCl_2_ | ND | ND | 100 |
| 3 | NiCl_2_(dppp) | none | Zn | MgCl_2_ | 88 | 6 | 26 |
| 4 | none | none | Zn | MgCl_2_ | ND | ND | 97 |
| 5 | NiCl_2_(dppp) | **L1** | none | MgCl_2_ | 133 | ND | ND |
| 6 | NiCl_2_(dppp) | **L1** | Zn | none | 120 | 1 | 3 |

*^a^*Reaction conditions (unless otherwise specified): **1a** (0.4 mmol, 1.0 equiv.), **2a** (1.4 equiv.), **3** (1.1 equiv.) and DMA (3 mL). *^b^*Determined by ^19^F NMR using fluorobenzene (0.4 mmol) as an internal standard. The yield of the products and recovery yield of **2a** are calculated using **1a** as reference substance. ND, not detected. *^c^*More unknown defluorinated byproducts would be produced in DMF than in DMA.

# General procedure for nickel catalyzed reductive fluoroalkylation-animation of enamides 1 with fluoroalkyl chlorides 2 and *N*-benzoyloxyamines 3.

To a 25 mL Schlenck tube were added enamide **1** (0.4 mmol, 1.0 equiv.), zinc powder (0.8 mmol, 2.0 equiv.), *N*-benzoyloxyamine **3** (0.44 mmol, 1.1 equiv.), **L1** (5 mol%), Ni(dppp)Cl_2_ (2.5 mol%) and MgCl_2_ (1.0 mmol, 2.5 equiv.) under air. The tube was evacuated and backfilled with argon for 3 times, then fluoroalkyl chloride **2** (0.56 mmol, 1.4 equiv.) and dry DMA (3 mL) were added via syringe under argon, and the tube was sealed with Teflon cap. The resulting mixture was stirred for 12 hours at room temperature. After stirring for 12 h, the reaction mixture was quenched with water and diluted with EtOAc. The reaction mixture was filtered through a pad of Celite, and the filtrate was washed with brine. The organic layer was dried over Na_2_SO_4_, filtered and concentrated. The residue was purified with silica gel chromatography to give the corresponding products **4** - **8**.

# Characterization Data for Compounds 4-8

1. **(4-(diethylamino)-3,3-difluoro-1-morpholino-4-oxobutyl)benzamide (4a).** The product (133.4 mg, 87% yield) as a yellow oil was purified with silica gel chromatography (Petroleum ether/Ethyl acetate = 1/1). ^1^H NMR (600 MHz, Acetone-*d*_6_) δ 7.96 – 7.85 (m, 3H), 7.52 (t, *J* = 7.4 Hz, 1H), 7.45 (t, *J* = 7.6 Hz, 2H), 5.44 – 5.36 (m, 1H), 3.64 – 3.54 (m, 5H), 3.50 (dt, *J* = 14.7, 7.2 Hz, 1H), 3.44 – 3.38 (m, 1H), 3.33 – 3.26 (m, 1H), 2.86 – 2.71 (m, 2H), 2.71 – 2.67 (m, 2H), 2.62 – 2.57 (m, 2H), 1.17 (t, *J* = 7.0 Hz, 3H), 1.08 (t, *J* = 7.1 Hz, 3H). ^19^F NMR (565 MHz, Acetone-*d*_6_) δ -98.47 (dt, *J* = 276.7, 16.2 Hz, 1F), -99.91 (dt, *J* = 276.7, 17.1 Hz, 1F). ^13^C NMR (151 MHz, Acetone-*d*_6_) δ 168.4, 163.9 (t, *J* = 28.4 Hz), 136.4, 132.9, 129.9, 128.9, 122.7 – 118.5 (m), 68.2, 65.7 (t, *J* = 5.9 Hz), 50.1, 43.5 (t, *J* = 6.6 Hz), 43.4, 39.0 (t, *J* = 23.0 Hz), 15.6, 13.4. MS (ESI): m/z (%) 789.4 (100, [2M+Na]^+^), 384.2 ([M+H]^+^). HRMS (ESI): Calculated for C_19_H_28_F_2_N_3_O_3_ ([M+H]^+^): 384.2093; Found: 384.2093.

**Gram-scale synthesis of compound 4a**

To a 100 mL of Schlenk tube were added enamide **1** (4 mmol, 1.0 equiv.), Zn (8 mmol, 2 equiv.), *N*-benzoyloxyamine **3** (4.4 mmol, 1.1 equiv.), 2,2'-bpy (5 mol%), Ni(dppp)Cl_2_ (2.5 mol%) and MgCl_2_ (10 mmol, 2.5 equiv.). The tube was evacuated and backfilled with argon for 3 times, then fluotoalkyl chloride **2** (5.6 mmol, 1.4 equiv.) and dry DMA (30 mL) were added via syringe under Ar, and the tube was sealed with Teflon cap. The resulting mixture was stirred for 12 hours at room temperature. After stirring for 12 h, the reaction mixture was quenched with water and diluted with EtOAc. The reaction mixture was filtered through a pad of Celite, and the filtrate was washed with brine. The organic layer was dried over Na_2_SO_4_, filtered and concentrated. The residue was purified with silica gel chromatography to give the corresponding products **4a** as a yellow oil (1.2 g, 80% yield).

***N*-(4-(dibutylamino)-3,3-difluoro-1-morpholino-4-oxobutyl)benzamide (4b).** The product (142.5 mg, 81% yield) as a yellow oil was purified with silica gel chromatography (Petroleum ether/Ethyl acetate = 2/3). ^1^H NMR (600 MHz, CDCl_3_) δ 7.90 (d, *J* = 7.4 Hz, 2H), 7.85 (d, *J* = 8.9 Hz, 1H), 7.52 (t, *J* = 7.4 Hz, 1H), 7.45 (t, *J* = 7.6 Hz, 2H), 5.43 – 5.36 (m, 1H), 3.63 – 3.54 (m, 4H), 3.50 (dt, *J* = 15.0, 6.7 Hz, 1H), 3.45 – 3.36 (m, 2H), 3.23 (dt, *J* = 13.2, 7.7 Hz, 1H), 2.84 – 2.73 (m, 2H), 2.71 – 2.66 (m, 2H), 2.62 – 2.57 (m, 2H), 1.63 – 1.56 (m, 2H), 1.53 – 1.48 (m, 2H), 1.28 (hept, *J* = 7.4 Hz, 4H), 0.92 – 0.87 (m, 6H). ^19^F NMR (565 MHz, CDCl_3_) δ -98.05 (dt, *J* = 276.7, 16.1 Hz, 1F), -98.98 (dt, *J* = 276.7, 16.7 Hz, 1F). ^13^C NMR (151 MHz, CDCl_3_) δ 168.4, 164.2 (t, *J* = 28.2 Hz), 136.4, 132.9, 129.9, 128.9, 120.8 (t, *J* = 255.1 Hz), 68.2, 65.7 (t, *J* = 6.0 Hz), 50.1, 49.1 (t, *J* = 6.2 Hz), 48.8, 39.1 (t, *J* = 23.1 Hz), 32.9, 30.7, 21.5, 21.3, 14.9, 14.8. MS (ESI): m/z (%) 901.5 (100, [2M+Na]^+^), 440.3 ([M+H]^+^). HRMS (ESI): Calculated for C_23_H_36_F_2_N_3_O_3_ ([M+H]^+^): 440.2719; Found: 440.2719.

***N*-(4-(azetidin-1-yl)-3,3-difluoro-1-morpholino-4-oxobutyl)benzamide (4c).** The product (90.0 mg, 61% yield) as a white solid (m.p. 129.9 – 130.6 ℃) was purified with silica gel chromatography (Petroleum ether/Ethyl acetate = 1/3). ^1^H NMR (600 MHz, CDCl_3_) δ 7.85 (d, *J* = 7.4 Hz, 2H), 7.47 (t, *J* = 7.4 Hz, 1H), 7.35 (t, *J* = 7.7 Hz, 2H), 7.05 (d, *J* = 9.0 Hz, 1H), 5.35 – 5.24 (m, 1H), 4.47 – 4.32 (m, 2H), 3.91 (q, *J* = 9.8 Hz, 1H), 3.70 – 3.59 (m, 4H), 3.59 – 3.51 (m, 1H), 2.86 – 2.76 (m, 1H), 2.66 – 2.49 (m, 5H), 2.26 – 2.07 (m, 2H). ^19^F NMR (565 MHz, CDCl_3_) δ -97.99 – -98.68 (m, 1F), -105.30 – -106.21 (m, 1F). ^13^C NMR (151 MHz, CDCl_3_) δ 167.4 (d, *J* = 3.1 Hz), 162.9 (t, *J* = 29.7 Hz), 133.6, 132.1, 128.9 – 128.5 (m), 127.6 – 126.9 (m), 120.5 – 116.3 (m), 67.0, 63.9, 53.2, 49.1, 48.4, 37.5 – 36.5 (m), 16.4. MS (ESI): m/z (%) 757.3 (100, [2M+Na]^+^), 368.2 ([M+H]^+^). HRMS (ESI): Calculated for C_18_H_24_F_2_N_3_O_3_ ([M+H]^+^): 368.1780; Found: 368.1780.

***N*-(3,3-difluoro-1-morpholino-4-oxo-4-(pyrrolidin-1-yl)butyl)benzamide (4d).** The product (121.5 mg, 80% yield) as a white solid (m.p. 131.3 – 132.7 ℃) was purified with silica gel chromatography (Petroleum ether/Ethyl acetate = 1/2). ^1^H NMR (600 MHz, Acetone-*d*_6_) δ 7.90 (d, *J* = 7.3 Hz, 2H), 7.85 (d, *J* = 8.6 Hz, 1H), 7.53 (t, *J* = 7.4 Hz, 1H), 7.46 (t, *J* = 7.6 Hz, 2H), 5.33 (q, *J* = 7.4 Hz, 1H), 3.71 – 3.67 (m, 2H), 3.60 – 3.52 (m, 4H), 3.44 (dt, *J* = 13.8, 7.1 Hz, 1H), 3.35 (dt, *J* = 12.6, 6.9 Hz, 1H), 2.93 – 2.85 (m, 1H), 2.70 – 2.65 (m, 2H), 2.61 – 2.54 (m, 3H), 1.95 (m, 2H), 1.79 (m, 2H). ^19^F NMR (565 MHz, Acetone-*d*_6_) δ -98.59 (dt, *J* = 268.7, 14.2 Hz, 1F), 102.56 – 103.35 (m, 1F). ^13^C NMR (151 MHz, Acetone-*d*_6_) δ 168.4, 163.4 (t, *J* = 28.8 Hz), 136.3, 133.0, 129.9, 129.0, 120.2 (dd, *J* = 254.6, 250.3 Hz), 68.2, 65.8 – 65.6 (m), 50.1, 49.4, 48.3 (dd, *J* = 9.0, 5.7 Hz), 39.0 – 38.6 (m), 28.0, 24.5. MS (ESI): m/z (%) 785.4 (100, [2M+Na]^+^), 382.2 ([M+H]^+^). HRMS (ESI): Calculated for C_19_H_26_F_2_N_3_O_3_ ([M+H]^+^): 382.1937; Found: 382.1937.

***N*-(3,3-difluoro-1,4-dimorpholino-4-oxobutyl)benzamide (4e).** The product (122.0 mg, 76% yield) as a white solid (m.p. 69.1 – 71.7 ℃) was purified with silica gel chromatography (Petroleum ether/Ethyl acetate = 1/1). ^1^H NMR (600 MHz, Acetone-*d*_6_) δ 7.89 (d, *J* = 7.9 Hz, 2H), 7.53 (t, *J* = 7.3 Hz, 1H), 7.45 (t, *J* = 7.5 Hz, 2H), 5.39 (q, *J* = 7.1 Hz, 1H), 3.76 – 3.69 (m, 2H), 3.67 – 3.59 (m, 6H), 3.56 (s, 4H), 2.91 (s, 1H), 2.80 – 2.72 (m, 2H), 2.70 – 2.65 (m, 2H), 2.62 – 2.57 (m, 2H). ^19^F NMR (565 MHz, Acetone-*d*_6_) δ -98.13 (dt, *J* = 276.9, 16.3 Hz, 1F), -99.35 (dt, *J* = 276.9, 17.9 Hz, 1F). ^13^C NMR (151 MHz, Acetone-*d*_6_) δ 168.5, 163.2 (t, *J* = 28.5 Hz), 136.3, 132.9, 129.9, 128.9, 120.5 (t, *J* = 254.1 Hz), 68.2, 68.0, 67.9, 65.5 (t, *J* = 5.5 Hz), 50.1, 48.1 (t, *J* = 6.5 Hz), 45.0, 39.1 (t, *J* = 22.5 Hz). MS (ESI): m/z (%) 817.4 (100, [2M+Na]^+^), 398.2 ([M+H]^+^). HRMS (ESI): Calculated for C_19_H_26_F_2_N_3_O_4_ ([M+H]^+^): 398.1886; Found: 398.1886.

**Gram-scale synthesis of compound 4e**

To a 100 mL of Schlenk tube were added enamide **1** (4 mmol, 1.0 equiv.), Zn (8 mmol, 2 equiv.), *N*-benzoyloxyamine **3** (4.4 mmol, 1.1 equiv.), 2,2'-bpy (5 mol%), Ni(dppp)Cl_2_ (2.5 mol%) and MgCl_2_ (10 mmol, 2.5 equiv.). The tube was evacuated and backfilled with argon for 3 times, then fluotoalkyl chloride **2e** (5.6 mmol, 1.4 equiv.) and dry DMA (30 mL) were added via syringe under Ar, and the tube was sealed with Teflon cap. The resulting mixture was stirred for 12 hours at room temperature. After stirring for 12 h, the reaction mixture was quenched with water and diluted with EtOAc. The reaction mixture was filtered through a pad of Celite, and the filtrate was washed with brine. The organic layer was dried over Na_2_SO_4_, filtered and concentrated. The residue was purified with silica gel chromatography to give the corresponding products **4e** as a white solid (1.1 g, 69% yield).

***N*-(3,3-difluoro-1-morpholino-4-oxo-4-thiomorpholinobutyl)benzamide (4f).** The product (106.6 mg, 64% yield) as a white solid (m.p. 66.0 – 67.1 ℃) was purified with silica gel chromatography (Petroleum ether/Ethyl acetate = 3/2). ^1^H NMR (600 MHz, Acetone-*d*_6_) δ 7.92 – 7.86 (m, 3H), 7.53 (t, *J* = 7.4 Hz, 1H), 7.46 (t, *J* = 7.7 Hz, 2H), 5.39 (dt, *J* = 8.7, 6.8 Hz, 1H), 3.96 – 3.93 (m, 2H), 3.83 (t, *J* = 4.9 Hz, 2H), 3.64 – 3.54 (m, 4H), 2.80 – 2.73 (m, 2H), 2.71 – 2.56 (m, 8H). ^19^F NMR (565 MHz, Acetone-*d*_6_) δ -98.15 (dt, *J* = 277.7, 16.2 Hz, 1F), -99.09 (dt, *J* = 277.5, 17.7 Hz, 1F). ^13^C NMR (151 MHz, Acetone-*d*_6_) δ 168.4, 163.2 (t, *J* = 28.5 Hz), 136.4, 132.9, 129.9, 128.9, 120.6 (t, *J* = 254.5 Hz), 68.2, 65.5 (t, *J* = 5.5 Hz), 50.4 (t, *J* = 6.6 Hz), 50.1, 47.7, 39.2 (t, *J* = 22.6 Hz), 29.3, 28.4. MS (ESI): m/z (%) 849.3 (100, [2M+Na]^+^), 414.2 ([M+H]^+^). HRMS (ESI): Calculated for C_19_H_26_F_2_N_3_O_3_S ([M+H]^+^): 414.1657; Found: 414.1657.

***N*-(4-(6,7-dimethoxy-3,4-dihydroisoquinolin-2(1H)-yl)-3,3-difluoro-1-morpholino-4-oxobutyl) benzamide (4g).** The product (88.0 mg, 44% yield) as a white solid (m.p. 89.8 – 90.3 ℃) was purified with silica gel chromatography (Petroleum ether/Ethyl acetate = 1/5). ^1^H NMR (600 MHz, Acetone-*d*_6_) δ 7.88 (t, *J* = 8.0 Hz, 3H), 7.51 (q, *J* = 7.6 Hz, 1H), 7.44 (dt, *J* = 15.1, 7.6 Hz, 2H), 6.83 – 6.71 (m, 2H), 5.40 (p, *J* = 7.2 Hz, 1H), 4.80 (s, 1H), 4.61 (d, *J* = 3.4 Hz, 1H), 3.91 (q, *J* = 6.2 Hz, 1H), 3.77 (d, *J* = 8.4 Hz, 7H), 3.58 – 3.50 (m, 4H), 2.85 – 2.72 (m, 4H), 2.71 – 2.54 (m, 4H). ^19^F NMR (565 MHz, Acetone-*d*_6_) δ -97.69 – -98.63 (m, 1F), -99.58 – -100.47 (m, 1F). ^13^C NMR (151 MHz, Acetone-*d*_6_) δ 168.4 (d, *J* = 2.8 Hz), 163.9 – 163.2 (m), 150.05 (t, *J* = 14.6 Hz), 150.0, 136.4 (d, *J* = 3.1 Hz), 132.9 (d, *J* = 5.2 Hz), 130.0 (d, *J* = 2.5 Hz), 128.9 (d, *J* = 5.3 Hz), 127.7 (d, *J* = 33.1 Hz), 126.2 (d, *J* = 47.0 Hz), 120.7 (t, *J* = 253.9 Hz), 113.7 (d, *J* = 15.7 Hz), 111.5 (d, *J* = 44.1 Hz), 68.2 (d, *J* = 5.4 Hz), 65.6(t, *J* = 5.7 Hz), 56.9 (d, *J* = 5.5 Hz), 50.1, 48.2 (t, *J* = 7.8 Hz), 46.8, 45.2 (t, *J* = 6.9 Hz), 43.4, 39.2 (t, *J* = 22.7 Hz), 28.8. MS (ESI): m/z (%) 1029.4 (100, [2M+Na]^+^), 504.2 ([M+H]^+^). HRMS (ESI): Calculated for C_26_H_32_F_2_N_3_O_5_ ([M+H]^+^): 504.2305; Found: 504.2305.

***N*-(4-(azepan-1-yl)-3,3-difluoro-1-morpholino-4-oxobutyl)benzamide (4h).** The product (128.5 mg, 79% yield) as a yellow oil was purified with silica gel chromatography (Petroleum ether/Ethyl acetate = 1/1). ^1^H NMR (600 MHz, Acetone-*d*_6_) δ 7.91 – 7.89 (m, 2H), 7.85 (d, *J* = 8.8 Hz, 1H), 7.52 (t, *J* = 7.4 Hz, 1H), 7.45 (t, *J* = 7.6 Hz, 2H), 5.42 – 5.35 (m, 1H), 3.69 (dt, *J* = 12.2, 6.3 Hz, 1H), 3.64 – 3.58 (m, 3H), 3.58 – 3.51 (m, 3H), 3.47 – 3.42 (m, 1H), 2.82 – 2.72 (m, 2H), 2.70 – 2.66 (m, 2H), 2.62 – 2.56 (m, 2H), 1.73 (p, *J* = 5.5 Hz, 2H), 1.69 – 1.60 (m, 2H), 1.58 – 1.46 (m, 4H). ^19^F NMR (565 MHz, Acetone-*d*_6_) δ -97.81 (dt, *J* = 277.4, 15.7 Hz, 1F), 98.87 (dt, *J* = 277.0, 16.9 Hz, 1F). ^13^C NMR (151 MHz, Acetone-*d*_6_) δ 168.4, 164.3 (t, *J* = 28.3 Hz), 136.4, 132.9, 129.9, 128.9, 122.8 – 118.8 (m), 68.2, 65.6 (t, *J* = 6.0 Hz), 50.1, 49.7, 49.3 (t, *J* = 6.4 Hz), 39.1 (t, *J* = 23.0 Hz), 31.2, 28.5, 27.9, 27.4. MS (ESI): m/z (%) 841.4 (100, [2M+Na]^+^), 410.2 ([M+H]^+^). HRMS (ESI): Calculated for C_21_H_30_F_2_N_3_O_3_ ([M+H]^+^): 410.2250; Found: 410.2250.

***N*-(4-(tert-butylamino)-3,3-difluoro-1-morpholino-4-oxobutyl)benzamide (4i).** The product (109.6 mg, 71% yield) as a white solid (m.p. 132.0 – 133.1 ℃) was purified with silica gel chromatography (Petroleum ether/Ethyl acetate = 1/1). ^1^H NMR (600 MHz, Acetone-*d*_6_) δ 7.89 (dd, *J* = 18.9, 8.2 Hz, 3H), 7.53 (t, *J* = 7.4 Hz, 1H), 7.46 (t, *J* = 7.6 Hz, 2H), 7.09 (s, 1H), 5.30 (q, *J* = 7.5 Hz, 1H), 3.65 – 3.56 (m, 4H), 2.79 – 2.71 (m, 1H), 2.70 – 2.64 (m, 2H), 2.61 – 2.55 (m, 2H), 2.54 – 2.48 (m, 1H), 1.38 (s, 9H). ^19^F NMR (565 MHz, Acetone-*d*_6_) δ -101.36 – -102.15 (m, 1F), -104.76 – -105.49 (m, 1F). ^13^C NMR (151 MHz, Acetone-*d*_6_) δ 168.6, 164.3 (t, *J* = 27.4 Hz), 136.4, 132.9, 129.9, 129.0, 120.9 – 116.9 (m), 68.1, 65.6 (t, *J* = 6.4 Hz), 53.0, 50.0, 38.0 (t, *J* = 23.4 Hz), 29.4. MS (ESI): m/z (%) 789.4 (100, [2M+Na]^+^), 384.2 ([M+H]^+^). HRMS (ESI): Calculated for C_19_H_28_F_2_N_3_O_3_ ([M+H]^+^): 384.2093; Found: 384.2093.

***N*-(4-(cyclohexylamino)-3,3-difluoro-1-morpholino-4-oxobutyl)benzamide (4j).** The product (71.5 mg, 44% yield) as a white solid (m.p. 143.4 – 145.4 ℃) was purified with silica gel chromatography (Petroleum ether/Ethyl acetate = 1/1). ^1^H NMR (600 MHz, CD_3_OD) δ 7.85 (d, *J* = 7.4 Hz, 2H), 7.56 (t, *J* = 7.3 Hz, 1H), 7.48 (t, *J* = 7.6 Hz, 2H), 5.22 (t, *J* = 6.9 Hz, 1H), 3.70 – 3.55 (m, 5H), 2.81 – 2.72 (m, 1H), 2.68 – 2.47 (m, 5H), 1.90 – 1.59 (m, 6H), 1.38 – 1.13 (m, 6H). ^19^F NMR (565 MHz, CD_3_OD) δ -103.22 – -104.09 (m, 1F), -106.88 – -107.67 (m, 1F). ^13^C NMR (151 MHz, CD_3_OD) δ 170.5, 165.0 (t, *J* = 28.7 Hz), 135.4, 132.9, 129.6, 128.6, 119.1 (d, *J* = 251.3 Hz), 67.9, 65.4 (t, *J* = 6.9 Hz), 50.6, 37.4 (t, *J* = 24.1 Hz), 33.2, 26.4, 26.3, 26.2. MS (ESI): m/z (%) 841.4 (100, [2M+Na]^+^), 410.2 ([M+H]^+^). HRMS (ESI): Calculated for C_21_H_30_F_2_N_3_O_3_ ([M+H]^+^): 410.2250; Found: 410.2250.

***N*-(3,3-difluoro-1-morpholino-4-oxo-4-(((R)-1-phenylethyl)amino)butyl)benzamide (4k).** The product (104.5 mg, 61% yield) as a yellow solid (m.p. 135.9 – 136.4 ℃) was purified with silica gel chromatography (Petroleum ether/Ethyl acetate = 2/3). ^1^H NMR (600 MHz, CD_3_OD) δ 7.86 – 7.81 (m, 2H), 7.55 (dt, *J* = 9.1, 7.5 Hz, 1H), 7.50 – 7.44 (m, 2H), 7.37 – 7.28 (m, 4H), 7.23 (dt, *J* = 9.2, 7.4 Hz, 1H), 5.24 (dt, *J* = 26.0, 7.0 Hz, 1H), 5.07 – 4.94 (m, 1H), 3.66 – 3.57 (m, 2H), 3.42 (t, *J* = 4.5 Hz, 2H), 2.96 – 2.75 (m, 1H), 2.68 – 2.63 (m, 1H), 2.59 (dt, *J* = 9.7, 4.6 Hz, 1H), 2.54 – 2.32 (m, 3H), 1.47 (dd, *J* = 74.5, 7.1 Hz, 3H). ^19^F NMR (565 MHz, CD_3_OD) δ -100.77 – -103.14 (m, 1F), -107.73 – -110.01 (m, 1F). ^13^C NMR (151 MHz, CD_3_OD) δ 170.6 (d, *J* = 10.4 Hz), 165.4 – 164.7 (m), 144.5 (d, *J* = 10.6 Hz), 135.4 (d, *J* = 10.8 Hz), 132.9 (d, *J* = 11.9 Hz), 129.6 (d, *J* = 10.4 Hz), 128.5 (d, *J* = 7.6 Hz), 128.3 (d, *J* = 18.3 Hz), 127.3 (d, *J* = 37.1 Hz), 120.2 – 116.4 (m), 67.9, 67.6, 65.5 (dd, *J* = 8.5, 4.7 Hz), 65.3 (t, *J* = 6.9 Hz), 50.7 (d, *J* = 22.7 Hz), 37.4 (t, *J* = 23.9 Hz), 37.0 (t, *J* = 23.7 Hz), 22.1 (d, *J* = 42.5 Hz). MS (ESI): m/z (%) 885.4 (100, [2M+Na]^+^), 432.2 ([M+H]^+^). HRMS (ESI): Calculated for C_23_H_28_F_2_N_3_O_3_ ([M+H]^+^): 432.2093; Found: 432.2093.

***N*-(4-((2-cyanoethyl)amino)-3,3-difluoro-1-morpholino-4-oxobutyl)benzamide (4l).** The product (60.5 mg, 40% yield) as a white solid (m.p. 125.2 – 125.9 ℃) was purified with silica gel chromatography (Petroleum ether/Ethyl acetate = 1/1). ^1^H NMR (600 MHz, Acetone-*d*_6_) δ 8.31 (s, 1H), 7.90 (d, *J* = 7.4 Hz, 3H), 7.54 (t, *J* = 7.3 Hz, 1H), 7.46 (t, *J* = 7.6 Hz, 2H), 5.29 (q, *J* = 8.7 Hz, 1H), 3.70 – 3.41 (m, 6H), 3.00 – 2.89 (m, 1H), 2.81 – 2.72 (m, 2H), 2.70 – 2.64 (m, 2H), 2.57 – 2.52 (m, 2H), 2.51 – 2.41 (m, 1H). ^19^F NMR (565 MHz, Acetone-*d*_6_) δ -100.45 – -101.12 (m, 1F), -108.75 – -109.41 (m, 1F). ^13^C NMR (151 MHz, Acetone-*d*_6_) δ 168.6, 165.8 (t, *J* = 28.5 Hz), 136.3, 133.0, 129.9, 129.0, 119.5, 118.9 (dd, *J* = 254.0, 248.6 Hz), 68.1, 65.7 (dd, *J* = 8.7, 5.2 Hz), 50.0, 37.8 (t, *J* = 23.7 Hz), 37.3, 18.8. MS (ESI): m/z (%) 783.3 (100, [2M+Na]^+^), 403.2 ([M+Na]^+^). HRMS (ESI): Calculated for C_18_H_22_F_2_N_4_O_3_Na ([M+Na]^+^): 403.1552; Found: 403.1522.

**Methyl 4-(4-benzamido-2,2-difluoro-4-morpholinobutanamido)butanoate (4m).** The product (77.15 mg, 45% yield) as a white solid (m.p. 126.5 – 127.1 ℃) was purified with silica gel chromatography (Petroleum ether/Ethyl acetate = 1/3). ^1^H NMR (600 MHz, Acetone-*d*_6_) δ 7.88 (dd, *J* = 13.8, 8.7 Hz, 3H), 7.53 (t, *J* = 7.3 Hz, 1H), 7.46 (t, *J* = 7.6 Hz, 2H), 5.27 (q, *J* = 8.0 Hz, 1H), 3.62 (s, 3H), 3.59 – 3.51 (m, 4H), 3.34 (dt, *J* = 12.6, 6.6 Hz, 1H), 3.24 (dt, *J* = 13.3, 6.7 Hz, 1H), 2.96 – 2.87 (m, 1H), 2.81 (s, 1H), 2.69 – 2.64 (m, 2H), 2.57 – 2.50 (m, 2H), 2.49 – 2.41 (m, 1H), 2.37 (t, *J* = 7.4 Hz, 2H), 1.88 – 1.78 (m, 2H). ^19^F NMR (565 MHz, Acetone-*d*_6_) -100.49 – -101.28 (m, 1F), -108.05 – -109.14 (m, 1F). ^13^C NMR (151 MHz, Acetone-*d*_6_) δ 174.6, 168.5, 165.3 (t, *J* = 27.6 Hz), 136.3, 133.0, 129.9, 129.0, 119.0 (dd, *J* = 253.7, 248.6 Hz), 68.1, 65.7 (dd, *J* = 8.4, 5.6 Hz), 52.4, 50.0, 40.3 (d, *J* = 18.8 Hz), 37.9 (t, *J* = 23.7 Hz), 32.4, 26.1 – 25.7 (m). MS (ESI): m/z (%) 877.4 (100, [2M+Na]^+^), 428.2 ([M+H]^+^). HRMS (ESI): Calculated for C_20_H_28_F_2_N_3_O_5_ ([M+H]^+^): 428.1992; Found: 428.1992.

**Tert-butyl (4-(4-benzamido-2,2-difluoro-4-morpholinobutanamido)butyl)carbamate (4n).**The product (88.5. mg, 44% yield) as a yellow solid (m.p. 137.6 – 138.3 ℃) was purified with silica gel chromatography (Petroleum ether/Ethyl acetate = 1/3). ^1^H NMR (600 MHz, CD_3_OD) δ 7.86 – 7.83 (m, 2H), 7.55 (t, *J* = 7.4 Hz, 1H), 7.48 (t, *J* = 7.7 Hz, 2H), 5.22 (t, *J* = 7.1 Hz, 1H), 3.71 – 3.57 (m, 4H), 3.26 (dt, *J* = 13.8, 7.0 Hz, 1H), 3.14 (dt, *J* = 13.5, 6.9 Hz, 1H), 3.02 (t, *J* = 6.8 Hz, 2H), 2.89 – 2.78 (m, 1H), 2.70 – 2.62 (m, 2H), 2.57 – 2.53 (m, 2H), 2.49 – 2.39 (m, 1H), 1.57 – 1.38 (m, 16H). ^19^F NMR (565 MHz, CD_3_OD) δ -102.09 – -102.88 (m, 1F), -108.60 – -109.37 (m, 1F). ^13^C NMR (151 MHz, CD_3_OD) δ 170.6, 166.0 (t, *J* = 28.5 Hz), 158.6, 135.4, 132.92, 129.6, 128.6, 118.3 (dd, *J* = 253.5, 248.7 Hz), 79.9, 67.9, 65.5 – 65.3 (m), 40.9, 40.4, 37.3 (t, *J* = 24.0 Hz), 28.8, 28.3, 27.4. MS (ESI): m/z (%) 1019.5 (100, [2M+Na]^+^), 499.3 ([M+H]^+^). HRMS (ESI): Calculated for C_24_H_37_F_2_N_4_O_5_ ([M+H]^+^): 499.2727; Found: 499.2727.

**Methyl *N*^2^-(4-benzamido-2,2-difluoro-4-morpholinobutanoyl)-*N*^6^-(tert-butoxycarbonyl)-L-lysinate (4o).** The product (86.0 mg, 38% yield) as a white solid (m.p. 143.3 – 143.5 ℃) was purified with silica gel chromatography (Petroleum ether/Ethyl acetate = 1/2). ^1^H NMR (600 MHz, Acetone-*d*_6_) δ 7.93 – 7.88 (m, 2H), 7.57 – 7.51 (m, 1H), 7.47 (q, *J* = 7.4 Hz, 2H), 5.99 (d, *J* = 16.3 Hz, 1H), 5.36 – 5.28 (m, 1H), 4.44 – 4.38 (m, 1H), 3.74 – 3.49 (m, 8H), 3.10 – 3.01 (m, 2H), 2.70 – 2.52 (m, 5H), 1.94 – 1.73 (m, 2H), 1.54 – 1.28 (m, 15H). ^19^F NMR (565 MHz, Acetone-*d*_6_) δ -100.15 – -102.46 (m, 1F), -106.83 – -108.73 (m, 1F). ^13^C NMR (151 MHz, Acetone-*d*_6_) δ 173.3 (d, *J* = 18.3 Hz), 168.6 (d, *J* = 7.6 Hz), 165.3 (q, *J* = 28.3 Hz), 157.5 (d, *J* = 7.2 Hz), 136.3 (d, *J* = 11.5 Hz), 133.0 (d, *J* = 10.9 Hz), 129.9 (d, *J* = 4.7 Hz), 129.0 (d, *J* = 4.7 Hz), 120.9 – 116.9 (m), 79.1, 68.1 (d, *J* = 22.9 Hz), 65.6 (t, *J* = 6.6 Hz), 54.3 (d, *J* = 47.1 Hz), 53.3 (d, *J* = 13.5 Hz), 50.0, 41.5 (d, *J* = 6.0 Hz), 38.3 – 37.6 (m), 32.6 (d, *J* = 48.5 Hz), 31.2 (d, *J* = 16.9 Hz), 29.4, 24.5 (d, *J* = 29.4 Hz). MS (ESI): m/z (%) 1163.6 (100, [2M+Na]^+^), 593.3 ([M+Na]^+^). HRMS (ESI): Calculated for C_27_H_40_F_2_N_4_O_7_Na ([M+Na]^+^): 593.2757; Found: 593.2757.

**Methyl (4-benzamido-2,2-difluoro-4-morpholinobutanoyl)-L-valinate (4p).** The product (92.65 mg, 53% yield) as a white solid (m.p. 50.1 – 50.9 ℃) was purified with silica gel chromatography (Petroleum ether/Ethyl acetate = 1/1). ^1^H NMR (600 MHz, CDCl_3_) δ 7.76 (d, *J* = 7.5 Hz, 2H), 7.51 (t, *J* = 7.4 Hz, 1H), 7.43 (t, *J* = 7.6 Hz, 2H), 6.99 (d, *J* = 8.0 Hz, 1H), 6.54 (d, *J* = 8.5 Hz, 1H), 5.31 (q, *J* = 7.8 Hz, 1H), 4.41 (dd, *J* = 8.6, 5.0 Hz, 1H), 3.75 (s, 3H), 3.63 – 3.52 (m, 4H), 2.88 – 2.77 (m, 1H), 2.70 – 2.65 (m, 2H), 2.58 – 2.53 (m, 2H), 2.53 – 2.45 (m, 1H), 2.14 – 2.00 (m, 1H), 0.90 (dd, *J* = 18.6, 7.0 Hz, 1H), 0.85 (t, *J* = 7.4 Hz, 5H). ^19^F NMR (565 MHz, CDCl_3_) δ -99.63 – -100.94 (m, 1F), -106.74 – -107.92 (m, 1F). ^13^C NMR (151 MHz, CDCl_3_) δ 171.4, 167.6, 163.9 (t, *J* = 28.6 Hz), 133.8, 132.1, 128.8, 127.2, 116.8 (dd, *J* = 255.3, 250.9 Hz), 66.8, 64.2 (dd, *J* = 8.0, 5.6 Hz), 57.5, 52.5, 48.3, 36.0 (t, *J* = 23.8 Hz), 31.6, 18.7, 18.0. MS (ESI): m/z (%) 905.4 (100, [2M+Na]^+^), 442.2 ([M+H]^+^). HRMS (ESI): Calculated for C_21_H_30_F_2_N_3_O_5_ ([M+H]^+^): 442.2148; Found: 442.2148.

**Methyl (4-benzamido-2,2-difluoro-4-morpholinobutanoyl)-L-methioninate (4q).** The product (60.6 mg, 32% yield) as a yellow solid (m.p. 116.8 – 117.5 ℃) was purified with silica gel chromatography (Petroleum ether/Ethyl acetate = 1/1).^1^H NMR (600 MHz, Acetone-*d_6_*) δ 8.16 (s, 1H), 7.94 – 7.87 (m, 3H), 7.57 – 7.50 (m, 1H), 7.50 – 7.42 (m, 2H), 5.39 – 5.27 (m, 1H), 4.71 – 4.55 (m, 1H), 3.72 (d, *J* = 24.1 Hz, 3H), 3.64 – 3.49 (m, 4H), 2.97 – 2.86 (m, 1H), 2.73 – 2.61 (m, 3H), 2.59 – 2.45 (m, 4H), 2.23 – 2.07 (m, 5H). ^19^F NMR (565 MHz, Acetone-*d_6_*) δ -100.21 – -101.77 (m, 1F), -107.66 – -108.75 (m, 1F). ^13^C NMR (151 MHz, Acetone-*d_6_*) δ 172.9 (dd, *J* = 5.1, 2.5 Hz), 168.6 (d, *J* = 9.2 Hz), 165.4 (td, *J* = 28.8, 25.6 Hz), 136.3 (d, *J* = 9.8 Hz), 133.0 (d, *J* = 8.5 Hz), 129.9 (d, *J* = 4.2 Hz), 129.0 (d, *J* = 4.5 Hz), 118.9 (ddd, *J* = 254.1, 249.7, 4.2 Hz), 68.0 (d, *J* = 22.1 Hz), 65.6 (dt, *J* = 9.1, 4.8 Hz), 53.5 – 53.3 (m), 53.3 – 53.1 (m), 50.0, 37.9 (td, *J* = 23.1, 14.6 Hz), 32.6 (d, *J* = 4.1 Hz), 32.2 (d, *J* = 5.0 Hz), 31.4 (d, *J* = 23.9 Hz), 15.8 (d, *J* = 5.4 Hz). MS (ESI): m/z (%) 969.3 (100, [2M+Na]^+^), 474.2 ([M+H]^+^). HRMS (ESI): Calculated for C_21_H_30_F_2_N_3_O_5_S ([M+H]^+^): 474.1869; Found: 474.1869.

**Methyl (4-benzamido-2,2-difluoro-4-morpholinobutanoyl)-L-tryptophanate (4r).**The product (80.3 mg, 38% yield) as a yellow oil was purified with silica gel chromatography (Petroleum ether/Ethyl acetate = 1/1). ^1^H NMR (600 MHz, CD_3_OD) δ 7.88 – 7.78 (m, 2H), 7.54 (t, *J* = 8.1 Hz, 1H), 7.50 – 7.43 (m, 3H), 7.33 (dd, *J* = 11.7, 8.1 Hz, 1H), 7.13 – 6.97 (m, 3H), 5.21 (dt, *J* = 42.4, 7.1 Hz, 1H), 4.85 (s, 3H), 4.77 – 4.66 (m, 1H), 3.65 (d, *J* = 4.8 Hz, 3H), 3.60 – 3.55 (m, 1H), 3.54 – 3.49 (m, 1H), 3.39 – 3.34 (m, 2H), 3.31 – 3.19 (m, 2H), 2.80 – 2.65 (m, 1H), 2.63 – 2.57 (m, 1H), 2.55 – 2.45 (m, 2H), 2.45 – 2.26 (m, 2H). ^19^F NMR (565 MHz, CD_3_OD) δ -102.61 – -103.60 (m, 1F), -108.55 – -109.23 (m, 1F). ^13^C NMR (151 MHz, CD_3_OD) δ 173.0, 170.6 (d, *J* = 9.3 Hz), 165.7 (q, *J* = 29.3 Hz), 138.0 (d, *J* = 11.9 Hz), 135.4, 132.9 (d, *J* = 8.3 Hz), 129.6 – 129.5 (m), 128.7 – 128.4 (m), 124.6 (d, *J* = 28.8 Hz), 122.6 (d, *J* = 21.1 Hz), 120.0 (d, *J* = 22.9 Hz), 119.1 (d, *J* = 12.7 Hz), 119.8 – 116.2 (m), 112.4 (d, *J* = 17.7 Hz), 110.2 (d, *J* = 16.0 Hz), 67.7, 65.2 (dt, *J* = 12.1, 6.1 Hz), 55.1 (d, *J* = 56.9 Hz), 52.9, 37.2 (t, *J* = 23.5 Hz), 37.0 (t, *J* = 23.6 Hz),31.9 (d, *J* = 17.4 Hz), 30.8 (d, *J* = 9.5 Hz), 28.2 (d, *J* = 36.1 Hz). MS (ESI): m/z (%) 1079.4 (100, [2M+Na]^+^), 529.2 ([M+H]^+^). HRMS (ESI): Calculated for C_27_H_31_F_2_N_4_O_5_ ([M+H]^+^): 529.2257; Found: 529.2257.

**Ethyl 4-benzamido-2,2-difluoro-4-morpholinobutanoate (4s).** The product (60.0 mg, 42% yield) as a yellow solid (m.p. 81.7 – 82.2 ℃) was purified with silica gel chromatography (Petroleum ether/Ethyl acetate = 1/1). ^1^H NMR (600 MHz, Acetone-*d*_6_) δ 8.02 (dd, *J* = 36.5, 8.0 Hz, 1H), 7.90 (d, *J* = 7.3 Hz, 2H), 7.54 (t, *J* = 7.4 Hz, 1H), 7.46 (t, *J* = 7.7 Hz, 2H), 5.34 – 5.26 (m, 1H), 4.32 (q, *J* = 7.1 Hz, 2H), 3.59 – 3.50 (m, 4H), 3.04 – 2.93 (m, 1H), 2.70 – 2.65 (m, 2H), 2.57 – 2.52 (m, 2H), 2.48 – 2.40 (m, 1H), 1.33 (t, *J* = 7.1 Hz, 3H). ^19^F NMR (565 MHz, Acetone-*d*_6_) δ -100.29 – -100.87 (m, 1F), -108.47 – -109.12 (m, 1F). ^13^C NMR (151 MHz, Acetone-*d*_6_) δ 168.6, 165.4 – 164.8 (m), 136.2, 133.0, 129.9, 129.0, 117.2 (dd, *J* = 253.2, 244.8 Hz), 67.9, 65.7 (dd, *J* = 9.9, 4.8 Hz), 64.3, 50.1, 38.6 (d, *J* = 47.7 Hz), 14.9. MS (ESI): m/z (%) 735.3 (100, [2M+Na]^+^), 357.2 ([M+H]^+^). HRMS (ESI): Calculated for C_17_H_23_F_2_N_2_O_4_ ([M+H]^+^): 357.1620; Found: 357.1620.

**Gram-scale synthesis of compound 4s**

To a 100 mL of Schlenk tube were added enamide **1** (8 mmol, 1.0 equiv.), Zn (16 mmol, 2 equiv.), *N*-benzoyloxyamine **3** (8.8 mmol, 1.1 equiv.), 2,2'-bpy (5 mol%), Ni(dppp)Cl_2_ (2.5 mol%) and MgCl_2_ (20 mmol, 2.5 equiv.). The tube was evacuated and backfilled with argon for 3 times, then fluotoalkyl chloride **2s** (16 mmol, 2.0 equiv.) and dry DMA (30 mL) were added via syringe under Ar, and the tube was sealed with Teflon cap. The resulting mixture was stirred for 12 hours at room temperature. After stirring for 12 h, the reaction mixture was quenched with water and diluted with EtOAc. The reaction mixture was filtered through a pad of Celite, and the filtrate was washed with brine. The organic layer was dried over Na_2_SO_4_, filtered and concentrated. The residue was purified with silica gel chromatography to give the corresponding products **4s** as a white solid (1.3 g, 44% yield).

***N*-(3-fluoro-1,4-dimorpholino-4-oxobutyl)benzamide (4t).** The product (36.5 mg, 24% yield) as a yellow solid (m.p. 50.2 – 51.2 ℃) was purified with silica gel chromatography (DCM). ^1^H NMR (600 MHz, Acetone-*d*_6_) δ 7.94 – 7.81 (m, 3H), 7.55 – 7.51 (m, 1H), 7.46 (t, *J* = 7.6 Hz, 2H), 5.63 – 5.34 (m, 1H), 5.25 – 5.18 (m, 1H), 3.67 – 3.50 (m, 11H), 2.90 (s, 1H), 2.73 – 2.65 (m, 2H), 2.62 – 2.42 (m, 3H), 2.39 – 2.08 (m, 1H). ^19^F NMR (565 MHz, Acetone-*d*_6_) δ -185.99 – -186.23 (m, 1F), -187.08 – -187.27 (m, 1F). ^13^C NMR (151 MHz, Acetone-*d*_6_) δ 168.9, 168.6, 168.0 (dd, *J* = 19.9, 16.6 Hz), 136.4 (d, *J* = 11.2 Hz), 132.9 (d, *J* = 4.3 Hz), 129.9, 129.0 (d, *J* = 3.2 Hz), 88.7 (d, *J* = 54.0 Hz), 87.5 (d, *J* = 54.6 Hz), 68.3 (d, *J* = 1.9 Hz), 68.2 – 67.9 (m), 67.2 (dd, *J* = 55.9, 5.7 Hz), 50.1, 47.5, 43.9, 36.0 (dd, *J* = 85.0, 21.7 Hz). MS (ESI): m/z (%) 781.4 (100, [2M+Na]^+^), 380.2 ([M+H]^+^). HRMS (ESI): Calculated for C_19_H_27_FN_3_O_4_ ([M+H]^+^): 380.1980; Found: 380.1980.

**Methyl 4-(4-benzamido-2,2-difluoro-4-morpholinobutanamido)-3-(4-chlorophenyl)butanoate (7a).** The product (115.5 mg, 54% yield) as a white solid (m.p. 134.8 – 135.0 ℃) was purified with silica gel chromatography (Petroleum ether/Ethyl acetate = 1/1). ^1^H NMR (600 MHz, Acetone-*d*_6_) δ 7.95 (s, 1H), 7.90 – 7.88 (m, 2H), 7.53 (t, *J* = 7.2 Hz, 1H), 7.45 (t, *J* = 7.3 Hz, 2H), 7.36 – 7.27 (m, 4H), 5.24 (dt, *J* = 17.1, 8.1 Hz, 1H), 3.64 – 3.38 (m, 10H), 2.92 – 2.78 (m, 3H), 2.71 – 2.60 (m, 3H), 2.55 – 2.47 (m, 2H), 2.46 – 2.34 (m, 1H). ^19^F NMR (565 MHz, Acetone-*d*_6_) δ -100.31 – -101.13 (m, 1F), -108.77 – -109.93 (m, 1F). ^13^C NMR (151 MHz, Acetone-*d*_6_) δ 173.2, 168.6, 165.8 – 165.3 (m), 142.3 (d, *J* = 7.9 Hz), 136.3 (d, *J* = 2.9 Hz), 133.7 (d, *J* = 4.0 Hz), 133.0, 131.2 (d, *J* = 6.0 Hz), 130.0 (d, *J* = 5.3 Hz), 129.9, 129.0, 120.8 – 117.2 (m), 68.1, 65.7 (dd, *J* = 8.6, 4.7 Hz), 52.5, 50.0, 45.9 (d, *J* = 24.5 Hz), 42.7 (d, *J* = 9.8 Hz), 39.2 (d, *J* = 11.0 Hz), 37.8 (t, *J* = 23.6 Hz). MS (ESI): m/z (%) 1097.4 (100, [2M+Na]^+^), 538.2 ([M+H]^+^). HRMS (ESI): Calculated for C_26_H_31_ClF_2_N_3_O_5_ ([M+H]^+^): 538.1915; Found: 538.1915.

**Methyl (3S)-3-((4-benzamido-2,2-difluoro-4-morpholinobutanamido)methyl)-5-methylhexanoate (7b).** The product (84.1 mg, 44% yield) as a yellow solid (m.p. 120.1 – 121.1 ℃) was purified with silica gel chromatography (Petroleum ether/Ethyl acetate = 1/1). ^1^H NMR (600 MHz, Acetone-*d*_6_) δ 7.88 (dd, *J* = 20.8, 7.9 Hz, 4H), 7.56 – 7.51 (m, 1H), 7.46 (t, *J* = 7.6 Hz, 2H), 5.30 – 5.24 (m, 1H), 3.63 – 3.51 (m, 7H), 3.43 – 3.38 (m, 0.5H), 3.26 – 3.21 (m, 1H), 3.13 – 3.07 (m, 0.5H), 2.95 – 2.86 (m, 1H), 2.66 (td, *J* = 7.6, 6.1, 2.9 Hz, 2H), 2.57 – 2.51 (m, 2H), 2.47 – 2.36 (m, 2H), 2.29 – 2.18 (m, 2H), 1.72 – 1.65 (m, 1H), 1.26 – 1.20 (m, 1H), 1.14 (dt, *J* = 13.8, 6.9 Hz, 1H), 0.91 – 0.85 (m, 6H). ^19^F NMR (565 MHz, Acetone-*d*_6_) δ -100.35 – -101.13 (m, 1F), -109.40 – -109.26 (m, 1F). ^13^C NMR (151 MHz, Acetone-*d*_6_) δ 174.4 (d, *J* = 4.0 Hz), 168.5 (d, *J* = 3.0 Hz), 165.6 (t, *J* = 27.8 Hz), 136.3, 133.0, 129.9, 129.01, 119.9 (d, *J* = 254.5 Hz), 68.1, 65.7 (d, *J* = 7.8 Hz), 52.4, 44.6 (d, *J* = 6.5 Hz), 43.1 (d, *J* = 10.2 Hz), 38.5 (d, *J* = 13.0 Hz), 37.9 (t, *J* = 23.8 Hz), 34.8, 26.7, 23.9 (d, *J* = 14.2 Hz), 23.6 (d, *J* = 16.9 Hz). MS (ESI): m/z (%) 989.5 (100, [2M+Na]^+^), 484.3 ([M+H]^+^). HRMS (ESI): Calculated for C_24_H_36_F_2_N_3_O_5_ ([M+H]^+^): 484.2618; Found: 484.2618.

***N*-(4-(diethylamino)-1-((2R,6S)-2,6-dimethylmorpholino)-3,3-difluoro-4-oxobutyl)benzamide (5a).** The product (94.0 mg, 57% yield) as a yellow oil was purified with silica gel chromatography (Petroleum ether/Ethyl acetate = 2/1). ^1^H NMR (600 MHz, Acetone-*d*_6_) δ 7.90 (d, *J* = 7.6 Hz, 2H), 7.84 (d, *J* = 8.5 Hz, 1H), 7.52 (t, *J* = 7.4 Hz, 1H), 7.45 (t, *J* = 7.6 Hz, 2H), 5.44 – 5.40 (m, 1H), 3.59 – 3.47 (m, 4H), 3.43 – 3.37 (m, 1H), 3.32 – 3.25 (m, 1H), 2.93 (s, 1H), 2.84 – 2.74 (m, 3H), 2.11 (t, *J* = 10.8 Hz, 1H), 1.99 (t, *J* = 10.4 Hz, 1H), 1.16 (t, *J* = 7.0 Hz, 3H), 1.08 (t, *J* = 6.8 Hz, 6H), 1.03 (d, *J* = 6.3 Hz, 3H). ^19^F NMR (565 MHz, Acetone-*d*_6_) δ -98.57 (dt, *J* = 276.7, 16.3 Hz, 1F), -99.87 (dt, *J* = 276.4, 16.7 Hz, 1F). ^13^C NMR (151 MHz, Acetone-*d*_6_) δ 168.3 (d, *J* = 4.0 Hz), 163.9 (t, *J* = 28.3 Hz), 136.3, 132.9, 129.9, 128.9, 122.8 – 118.7 (m), 73.0 (d, *J* = 23.3 Hz), 65.3 (t, *J* = 5.8 Hz), 59.4, 52.3, 43.5 (t, *J* = 6.6 Hz), 43.4, 39.1 (t, *J* = 22.9 Hz), 20.3 (d, *J* = 18.6 Hz), 15.6, 13.4. MS (ESI): m/z (%) 845.5 (100, [2M+Na]^+^), 412.2 ([M+H]^+^). HRMS (ESI): Calculated for C_21_H_32_F_2_N_3_O_3_ ([M+H]^+^): 412.2406; Found: 412.2406.

***N*-(4-(diethylamino)-3,3-difluoro-4-oxo-1-thiomorpholinobutyl)benzamide (5b).** The product (99.0 mg, 62% yield) as a yellow oil was purified with silica gel chromatography (Petroleum ether/Ethyl acetate = 3/1). ^1^H NMR (600 MHz, Acetone-*d*_6_) δ 7.88 (dd, *J* = 16.1, 8.2 Hz, 3H), 7.52 (t, *J* = 7.4 Hz, 1H), 7.45 (t, *J* = 7.7 Hz, 2H), 5.40 – 5.35 (m, 1H), 3.58 – 3.47 (m, 2H), 3.57 – 3.47 (m, 1H), 3.44 – 3.38 (m, 1H), 3.02 – 2.97 (m, 2H), 2.91 – 2.85 (m, 2H), 2.85 – 2.77 (m, 1H), 2.74 – 2.66 (m, 1H), 2.65 – 2.55 (m, 4H), 1.18 (t, *J* = 7.0 Hz, 3H), 1.09 (t, *J* = 7.1 Hz, 3H). ^19^F NMR (565 MHz, Acetone-*d*_6_) δ -98.54 (dt, *J* = 277.1, 16.4 Hz, 1F), -99.84 (dt, *J* = 277.1, 17.0 Hz, 1F). ^13^C NMR (151 MHz, Acetone-*d*_6_) δ 168.3, 163.9 (t, *J* = 28.4 Hz), 136.4, 132.9, 129.9, 128.9, 122.8 – 118.2 (m), 67.1 (t, *J* = 5.9 Hz), 52.7, 43.5 (t, *J* = 6.6 Hz), 43.3, 39.2 (t, *J* = 22.9 Hz), 29.3, 15.6, 13.4. MS (ESI): m/z (%) 821.4 (100, [2M+Na]^+^), 400.2 ([M+H]^+^). HRMS (ESI): Calculated for C_19_H_28_F_2_N_3_O_2_S ([M+H]^+^): 400.1865; Found: 400.1865.

***Tert*-butyl 4-(1-benzamido-4-(diethylamino)-3,3-difluoro-4-oxobutyl)piperazine-1-carboxylate (5c).** The product (97.0 mg, 50% yield) as a white solid (m.p. 135.4 – 136.2 ℃) was purified with silica gel chromatography (Petroleum ether/Ethyl acetate = 1/1). ^1^H NMR (600 MHz, Acetone-*d*_6_) δ 7.88 (d, *J* = 7.3 Hz, 2H), 7.81 (d, *J* = 8.9 Hz, 1H), 7.52 (t, *J* = 7.4 Hz, 1H), 7.45 (t, *J* = 7.6 Hz, 2H), 5.47 – 5.42 (m, 1H), 3.58 – 3.48 (m, 2H), 3.44 – 3.34 (m, 4H), 3.33 – 3.26 (m, 2H), 2.82 (dd, *J* = 15.7, 6.0 Hz, 1H), 2.78 – 2.72 (m, 1H), 2.71 – 2.63 (m, 2H), 2.58 – 2.55 (m, 2H), 1.40 (s, 9H), 1.18 (t, *J* = 7.0 Hz, 3H), 1.09 (t, *J* = 7.1 Hz, 3H). ^19^F NMR (565 MHz, Acetone-*d*_6_) δ -98.55 (dt, *J* = 277.1, 16.1 Hz, 1F), -99.93 (dt, *J* = 277.2, 17.0 Hz, 1F). ^13^C NMR (151 MHz, Acetone-*d*_6_) δ 168.3, 163.9 (t, *J* = 28.3 Hz), 155.7, 136.4, 133.0, 129.9, 128.9, 122.6 – 118.6 (m), 80.2, 65.7 (t, *J* = 6.1 Hz), 43.5 (t, *J* = 6.7 Hz), 43.4, 39.3 (t, *J* = 23.0 Hz), 29.3, 15.6, 13.4. MS (ESI): m/z (%) 987.5 (100, [2M+Na]^+^), 483.3 ([M+H]^+^). HRMS (ESI): Calculated for C_24_H_37_F_2_N_4_O_4_ ([M+H]^+^): 483.2777; Found: 483.2777.

***N*-(4-(diethylamino)-3,3-difluoro-4-oxo-1-(4-(pyrimidin-2-yl)piperazin-1-yl)butyl)benzamide (5d).** The product (98.0 mg, 53% yield) as a yellow solid (m.p. 131.0 – 131.6 ℃) was purified with silica gel chromatography (Petroleum ether/Ethyl acetate = 1/1). ^1^H NMR (600 MHz, Acetone-*d*_6_) δ 8.28 (d, *J* = 4.7 Hz, 2H), 7.87 (d, *J* = 7.5 Hz, 3H), 7.49 (t, *J* = 7.3 Hz, 1H), 7.42 (t, *J* = 7.6 Hz, 2H), 6.51 (t, *J* = 4.7 Hz, 1H), 5.54 – 5.48 (m, 1H), 3.83 – 3.74 (m, 4H), 3.60 – 3.55 (m, 1H), 3.51 (dt, *J* = 14.2, 7.0 Hz, 1H), 3.43 (dt, *J* = 13.5, 7.0 Hz, 1H), 3.32 – 3.27 (m, 1H), 2.90 – 2.84 (m, 1H), 2.81 – 2.75 (m, 3H), 2.70 – 2.66 (m, 2H), 1.18 (t, *J* = 7.0 Hz, 3H), 1.09 (t, *J* = 7.1 Hz, 3H). ^19^F NMR (565 MHz, Acetone-*d*_6_) δ -98.43 (dt, *J* = 276.4, 16.2 Hz, 1F), -99.88 (dt, *J* = 276.4, 17.1 Hz, 1F). ^13^C NMR (151 MHz, Acetone-*d*_6_) δ 168.4, 164.0 (t, *J* = 28.4 Hz), 163.4, 159.3, 136.4, 132.9, 129.9, 129.1, 128.9, 123.0 – 118.4 (m), 111.4, 65.7 (t, *J* = 6.0 Hz), 49.6, 45.2, 43.6 (t, *J* = 6.6 Hz), 43.4, 39.30 (t, *J* = 22.9 Hz), 15.6, 13.4. MS (ESI): m/z (%) 943.5 (100, [2M+Na]^+^), 461.3 ([M+H]^+^). HRMS (ESI): Calculated for C_23_H_31_F_2_N_6_O_2_ ([M+H]^+^): 461.2471; Found: 461.2471.

***N*-(1-(4-(benzo[d]isothiazol-3-yl)piperazin-1-yl)-4-(diethylamino)-3,3-difluoro-4-oxobutyl)benzamide (5e).** The product (123.5 mg, 60% yield) as a white solid (m.p. 66.3 – 66.9 ℃) was purified with silica gel chromatography (Petroleum ether/Ethyl acetate = 2/1). ^1^H NMR (600 MHz, Acetone-*d_6_*) δ 8.05 (d, *J* = 8.2 Hz, 1H), 7.95 (dd, *J* = 22.9, 7.6 Hz, 4H), 7.55 – 7.50 (m, 2H), 7.47 (t, *J* = 6.8 Hz, 2H), 7.43 – 7.38 (m, 1H), 5.53 (q, *J* = 7.8 Hz, 1H), 3.58 (dt, *J* = 12.9, 7.0 Hz, 1H), 3.51 (s, 4H), 3.44 (dd, *J* = 13.4, 6.9 Hz, 1H), 3.31 (dt, *J* = 13.1, 6.8 Hz, 1H), 3.01 – 2.95 (m, 2H), 2.90 – 2.84 (m, 3H), 2.82 – 2.74 (m, 2H), 1.19 (t, *J* = 6.8 Hz, 3H), 1.10 (t, *J* = 6.9 Hz, 3H). ^19^F NMR (565 MHz, Acetone-*d_6_*) δ -98.49 (dt, *J* = 276.6, 16.0 Hz, 1F), -99.85 (dt, *J* = 276.7, 16.9 Hz, 1F). ^13^C NMR (151 MHz, Acetone-*d_6_*) δ 168.4, 165.6, 164.0 (t, *J* = 28.6 Hz), 154.3, 136.4, 132.9, 129.9, 129.6, 129.3, 129.0, 125.8 (d, *J* = 10.8 Hz), 122.2, 120.8 (t, *J* = 254.7 Hz), 65.6 (t, *J* = 6.0 Hz), 51.8, 49.5, 43.6 (t, *J* = 6.4 Hz), 43.4, 39.3 (t, *J* = 22.9 Hz), 15.6, 13.5. MS (ESI): m/z (%) 1031.5 (100, [2M+H]^+^), 516.2 ([M+H]^+^). HRMS (ESI): Calculated for C_26_H_32_F_2_N_5_O_2_S ([M+H]^+^): 516.2239; Found: 516.2239.

***N*-(4-(diethylamino)-1-(4,4-difluoropiperidin-1-yl)-3,3-difluoro-4-oxobutyl)benzamide (5f).** The product (101.0 mg, 61% yield) as a yellow oil was purified with silica gel chromatography (Petroleum ether/Ethyl acetate = 3/1). ^1^H NMR (600 MHz, Acetone-*d*_6_) δ 7.90 – 7.86 (m, 2H), 7.54 – 7.50 (m, 1H), 7.45 (t, *J* = 7.6 Hz, 2H), 5.50 (q, *J* = 8.1 Hz, 1H), 3.60 – 3.47 (m, 2H), 3.45 – 3.38 (m, 1H), 3.34 – 3.28 (m, 1H), 2.87 (s, 1H), 2.86 – 2.81 (m, 3H), 2.79 – 2.68 (m, 3H), 2.00 – 1.89 (m, 4H), 1.18 (t, *J* = 7.0 Hz, 3H), 1.09 (t, *J* = 7.1 Hz, 3H). ^19^F NMR (565 MHz, Acetone-*d*_6_) δ -98.50 (dt, *J* = 277.8, 16.4 Hz, 1F), -100.09 (dt, *J* = 277.7, 17.2 Hz, 1F). ^13^C NMR (151 MHz, Acetone-*d*_6_) δ 168.4, 163.9 (t, *J* = 28.3 Hz), 136.3, 132.9, 129.9, 128.9, 123.8, 125.9 – 118.5 (m), 120.7, 65.3 (t, *J* = 6.1 Hz), 46.8, 43.5 (t, *J* = 6.6 Hz), 43.3, 39.5 (t, *J* = 22.9 Hz), 35.7 (t, *J* = 22.6 Hz), 15.5, 13.4. MS (ESI): m/z (%) 857.4 (100, [2M+Na]^+^), 418.2 ([M+H]^+^). HRMS (ESI): Calculated for C_20_H_28_F_4_N_3_O_2_ ([M+H]^+^): 418.2112; Found: 418.2112.

***N*-(4-(diethylamino)-3,3-difluoro-4-oxo-1-(4-phenylpiperidin-1-yl)butyl)benzamide (5g).** The product (110.0 mg, 60% yield) as a yellow oil was purified with silica gel chromatography (Petroleum ether/Ethyl acetate = 4/1). ^1^H NMR (600 MHz, Acetone-*d*_6_) δ 7.92 (d, *J* = 7.2 Hz, 2H), 7.83 (d, *J* = 8.9 Hz, 1H), 7.53 (t, *J* = 7.4 Hz, 1H), 7.46 (t, *J* = 7.8 Hz, 2H), 7.27 (t, *J* = 7.6 Hz, 2H), 7.22 (d, *J* = 7.1 Hz, 2H), 7.16 (t, *J* = 7.2 Hz, 1H), 5.49 (q, *J* = 8.3 Hz, 1H), 3.60 – 3.51 (m, 2H), 3.48 – 3.42 (m, 1H), 3.35 – 3.28 (m, 1H), 3.15 (d, *J* = 11.1 Hz, 1H), 3.08 (d, *J* = 11.6 Hz, 1H), 2.88 – 2.85 (m, 1H), 2.80 – 2.74 (m, 1H), 2.56 – 2.51 (m, 1H), 2.49 – 2.43 (m, 1H), 2.42 – 2.43 (m, 1H), 1.83 – 1.75 (m, 2H), 1.72 – 1.64 (m, 2H), 1.20 (t, *J* = 7.0 Hz, 3H), 1.10 (t, *J* = 7.1 Hz, 3H). ^19^F NMR (565 MHz, Acetone-*d*_6_) δ -93.29 (dt, *J* = 275.4, 16.3 Hz, 1F), -94.57 (dt, *J* = 275.7, 17.2 Hz, 1F). ^13^C NMR (151 MHz, Acetone-*d*_6_) δ 168.3, 164.0 (t, *J* = 28.3 Hz), 148.2, 136.5, 132.8, 129.9 (d, *J* = 7.7 Hz), 128.9, 128.3, 127.6, 120.8 (t, *J* = 254.5 Hz), 65.8 (t, *J* = 5.9 Hz), 54.5, 46.7, 44.2, 43.6 (t, *J* = 6.6 Hz), 43.4, 39.5 (t, *J* = 22.9 Hz), 35.4, 34.9, 15.6, 13.5. MS (ESI): m/z (%) 937.5 (100, [2M+Na]^+^), 458.3 ([M+H]^+^). HRMS (ESI): Calculated for C_26_H_34_F_2_N_3_O_2_ ([M+H]^+^): 458.2614; Found: 458.2614.

***N*-(1-(4-benzylpiperidin-1-yl)-4-(diethylamino)-3,3-difluoro-4-oxobutyl)benzamide (5h).** The product (106.6 mg, 56% yield) as a yellow oil was purified with silica gel chromatography (Petroleum ether/Ethyl acetate = 2/1). ^1^H NMR (600 MHz, Acetone-*d*_6_) δ 7.89 – 7.85 (m, 2H), 7.76 (d, *J* = 9.0 Hz, 1H), 7.52 – 7.48 (m, 1H), 7.43 (t, *J* = 7.6 Hz, 2H), 7.27 – 7.23 (m, 2H), 7.15 (t, *J* = 7.5 Hz, 3H), 5.44 – 5.38 (m, 1H), 3.56 – 3.47 (m, 2H), 3.43 – 3.37 (m, 1H), 3.27 – 3.21 (m, 1H), 3.01 (d, *J* = 9.4 Hz, 1H), 2.93 (d, *J* = 11.0 Hz, 1H), 2.83 – 2.73 (m, 1H), 2.72 – 2.61 (m, 1H), 2.55 – 2.47 (m, 2H), 2.40 – 2.32 (m, 1H), 2.24 – 2.16 (m, 1H), 1.63 – 1.56 (m, 2H), 1.53 – 1.44 (m, 1H), 1.29 – 1.19 (m, 1H), 1.17 (q, *J* = 7.1, 6.5 Hz, 4H), 1.08 (t, *J* = 7.1 Hz, 3H). ^19^F NMR (565 MHz, Acetone-*d*_6_) δ -93.27 (dt, *J* = 275.4, 16.3 Hz, 1F), -94.69 (dt, *J* = 274.4, 17.2 Hz, 1F). ^13^C NMR (151 MHz, Acetone-*d*_6_) δ 168.2, 164.0 (t, *J* = 28.3 Hz), 142.3, 136.6, 132.8, 130.7, 129.8 (d, *J* = 24.4 Hz), 128.9, 127.3, 122.7 – 118.6 (m), 65.8 (t, *J* = 6.0 Hz), 53.9, 46.4, 44.5, 43.6 (t, *J* = 6.7 Hz), 43.4, 39.6, 39.5 (t, *J* = 22.9 Hz), 34.0, 33.5, 15.6, 13.5. MS (ESI): m/z (%) 943.5 (100, [2M+Na]^+^), 472.3 ([M+H]^+^). HRMS (ESI): Calculated for C_27_H_36_F_2_N_3_O_2_ ([M+H]^+^): 472.2770; Found: 472.2770.

**Ethyl 1-(1-benzamido-4-(diethylamino)-3,3-difluoro-4-oxobutyl)piperidine-4-carboxylate (5i).** The product (108.5 mg, 60% yield) as a yellow solid (m.p. 83.7 – 84.5 ℃) was purified with silica gel chromatography (Petroleum ether/Ethyl acetate = 2/1). ^1^H NMR (600 MHz, Acetone-*d*_6_) δ 7.89 (d, *J* = 7.5 Hz, 2H), 7.80 (d, *J* = 8.4 Hz, 1H), 7.51 (t, *J* = 7.4 Hz, 1H), 7.44 (t, *J* = 7.5 Hz, 2H), 5.45 – 5.40 (m, 1H), 4.06 (q, *J* = 7.1 Hz, 2H), 3.58 – 3.48 (m, 2H), 3.44 – 3.39 (m, 1H), 3.32 – 3.25 (m, 1H), 3.03 (d, *J* = 11.2 Hz, 1H), 2.95 (d, *J* = 11.8 Hz, 1H), 2.86 – 2.64 (m, 2H), 2.49 – 2.40 (m, 1H), 2.34 – 2.27 (m, 1H), 2.26 – 2.16 (m, 1H), 1.88 – 1.79 (m, 2H), 1.69 – 1.56 (m, 2H), 1.22 – 1.16 (m, 6H), 1.09 (t, *J* = 7.1 Hz, 3H). ^19^F NMR (565 MHz, Acetone-*d*_6_) δ -98.43 (dt, *J* = 275.4, 16.2 Hz, 1F), -99.99 (dt, *J* = 276.1, 17.9 Hz, 1F). ^13^C NMR (151 MHz, Acetone-*d*_6_) δ 176.0, 168.3 (d, *J* = 4.3 Hz), 164.0 (t, *J* = 28.2 Hz), 136.5, 132.8, 129.9, 128.9, 122.8 – 118.5 (m), 65.8 (dt, *J* = 13.1, 5.9 Hz), 61.3, 52.6, 46.2, 43.6 (t, *J* = 6.6 Hz), 43.4, 42.6, 39.4 (td, *J* = 23.0, 5.6 Hz), 15.6, 15.3, 13.5. MS (ESI): m/z (%) 929.5 (100, [2M+Na]^+^), 454.3 ([M+H]^+^). HRMS (ESI): Calculated for C_23_H_34_F_2_N_3_O_4_ ([M+H]^+^): 454.2512; Found: 454.2512.

***N*-(4-(diethylamino)-3,3-difluoro-1-(4-(hydroxymethyl)piperidin-1-yl)-4-oxobutyl)benzamide (5j).** The product (60.9 mg, 37% yield) as a yellow oil was purified with silica gel chromatography (Petroleum ether/Ethyl acetate = 1/3). ^1^H NMR (600 MHz, Acetone-*d*_6_) δ 7.99 – 7.84 (m, 2H), 7.73 (d, *J* = 8.3 Hz, 1H), 7.51 (t, *J* = 7.2 Hz, 1H), 7.45 (t, *J* = 7.3 Hz, 2H), 5.42 (q, *J* = 7.6 Hz, 1H), 3.53 (dd, *J* = 13.6, 6.8 Hz, 1H), 3.49 (dd, *J* = 14.1, 6.5 Hz, 1H), 3.44 – 3.38 (m, 1H), 3.35 (d, *J* = 6.0 Hz, 2H), 3.31 – 3.25 (m, 1H), 3.03 (d, *J* = 10.9 Hz, 1H), 2.96 (d, *J* = 11.5 Hz, 1H), 2.79 – 2.64 (m, 2H), 2.38 (t, *J* = 11.7 Hz, 1H), 2.23 (t, *J* = 11.4 Hz, 1H), 1.70 (dd, *J* = 30.8, 12.5 Hz, 2H), 1.41 – 1.27 (m, 2H), 1.17 (t, *J* = 6.9 Hz, 4H), 1.08 (t, *J* = 7.0 Hz, 3H). ^19^F NMR (565 MHz, Acetone-*d*_6_) δ -98.67 (dt, *J* = 275.4, 16.3 Hz, 1F), -99.77 (dt, *J* = 275.4, 17.1 Hz, 1F). ^13^C NMR (151 MHz, Acetone-*d*_6_) δ 168.2 (d, *J* = 3.0 Hz), 164.0 (t, *J* = 28.3 Hz), 136.7, 132.8, 129.9, 128.9, 120.8 (t, *J* = 254.6 Hz), 68.5, 65.9 (t, *J* = 5.9 Hz), 53.7, 46.3, 43.6 (t, *J* = 6.6 Hz), 43.4, 40.6, 39.6 (t, *J* = 22.9 Hz), 15.6, 13.5. MS (ESI): m/z (%) 412.2 ([M+H]^+^). HRMS (ESI): Calculated for C_21_H_32_F_2_N_3_O_3_ ([M+H]^+^): 412.2406; Found: 412.2406.

***N*-(4-(diethylamino)-3,3-difluoro-1-(4-(hydroxydiphenylmethyl)piperidin-1-yl)-4-oxobutyl) benzamide (5k).** The product (124.0 mg, 55% yield) as a white solid (m.p. 90.0 – 91.0 ℃) was purified with silica gel chromatography (Petroleum ether/Ethyl acetate = 2/1). ^1^H NMR (600 MHz, Acetone-*d*_6_) δ 7.91 (d, *J* = 7.4 Hz, 2H), 7.73 (d, *J* = 8.6 Hz, 1H), 7.55 (dd, *J* = 22.8, 7.0 Hz, 4H), 7.50 – 7.44 (m, 2H), 7.27 – 7.21 (m, 4H), 7.12 (s, 2H), 5.43 – 5.36 (m, 1H), 4.22 (s, 1H), 3.56 – 3.45 (m, 2H), 3.42 – 3.35 (m, 1H), 3.27 – 3.18 (m, 1H), 3.00 (dd, *J* = 38.8, 10.8 Hz, 2H), 2.77 – 2.58 (m, 3H), 2.50 (dt, *J* = 32.4, 11.6 Hz, 2H), 2.31 (t, *J* = 11.3 Hz, 1H), 1.58 – 1.41 (m, 4H), 1.15 (t, *J* = 6.8 Hz, 3H), 1.07 (t, *J* = 6.9 Hz, 3H). ^19^F NMR (565 MHz, Acetone-*d*_6_) δ -98.62 (dt, *J* = 275.1, 16.0 Hz, 1F), -99.78 (dt, *J* = 275.0, 17.0 Hz, 1F). ^13^C NMR (151 MHz, Acetone-*d*_6_) δ 168.2 (d, *J* = 18.9 Hz), 164.1 (t, *J* = 28.2 Hz), 148.9 (d, *J* = 15.1 Hz), 136.6, 132.9, 129.9, 129.4 (d, *J* = 3.0 Hz), 129.0, 127.7, 127.6 (d, *J* = 6.9 Hz), 120.8 (t, *J* = 254.5 Hz), 80.7, 65.7 (d, *J* = 5.9 Hz), 54.4, 46.4, 45.9, 43.7 (d, *J* = 6.1 Hz), 43.6, 39.6 (t, *J* = 22.8 Hz), 28.3, 27.8, 15.7, 13.5. MS (ESI): m/z (%) 1127.6 (100, [2M+Na]^+^), 564.3 ([M+H]^+^). HRMS (ESI): Calculated for C_33_H_40_F_2_N_3_O_3_ ([M+H]^+^): 564.3032; Found: 564.3032.

***N*-(4-(diethylamino)-3,3-difluoro-1-(4-hydroxypiperidin-1-yl)-4-oxobutyl)benzamide (5l).** The product (87.5 mg, 55% yield) as a yellow oil was purified with silica gel chromatography (Ethyl acetate). ^1^H NMR (600 MHz, Acetone-*d*_6_) δ 7.88 (d, *J* = 7.6 Hz, 2H), 7.77 (d, *J* = 8.9 Hz, 1H), 7.51 (t, *J* = 7.3 Hz, 1H), 7.44 (t, *J* = 7.6 Hz, 2H), 5.43 (q, *J* = 8.2 Hz, 1H), 3.65 (s, 1H), 3.58 – 3.47 (m, 3H), 3.45 – 3.38 (m, 1H), 3.31 – 3.25 (m, 1H), 2.96 (dd, *J* = 10.6, 4.9 Hz, 1H), 2.90 – 2.85 (m, 1H), 2.80 – 2.65 (m, 2H), 2.49 – 2.44 (m, 1H), 2.33 (t, *J* = 9.7 Hz, 1H), 1.84 – 1.75 (m, 2H), 1.52 – 1.40 (m, 2H), 1.17 (t, *J* = 7.0 Hz, 3H), 1.08 (t, *J* = 7.1 Hz, 3H). ^19^F NMR (565 MHz, Acetone-*d*_6_) δ -97.06 (dt, *J* = 275.3, 16.3 Hz, 1F), -98.13 (dt, *J* = 275.3, 17.1 Hz, 1F). ^13^C NMR (151 MHz, Acetone-*d*_6_) δ 168.2, 164.0 (t, *J* = 28.3 Hz), 136.5, 132.8, 129.9, 128.9, 120.8 (t, *J* = 254.5 Hz), 68.8, 65.6 (t, *J* = 5.9 Hz), 49.76, 45.5, 43.6 (t, *J* = 6.6 Hz), 43.4, 39.6 (t, *J* = 22.8 Hz), 36.5, 36.2, 15.6, 13.4. MS (ESI): m/z (%) 817.4 (100, [2M+Na]^+^), 398.2 ([M+H]^+^). HRMS (ESI): Calculated for C_20_H_30_F_2_N_3_O_3_ ([M+H]^+^): 398.2250; Found: 398.2250.

**Gram-scale synthesis of compound 5l**

To a 100 mL of Schlenk tube were added enamide **1** (6 mmol, 1.0 equiv.), Zn (12 mmol, 2 equiv.), *N*-benzoyloxyamine **3l** (6.6 mmol, 1.1 equiv.), 2,2'-bpy (5 mol%), Ni(dppp)Cl_2_ (2.5 mol%) and MgCl_2_ (15 mmol, 2.5 equiv.). The tube was evacuated and backfilled with argon for 3 times, then fluotoalkyl chloride **2** (8.4 mmol, 1.4 equiv.) and dry DMA (30 mL) were added via syringe under Ar, and the tube was sealed with Teflon cap. The resulting mixture was stirred for 12 hours at room temperature. After stirring for 12 h, the reaction mixture was quenched with water and diluted with EtOAc. The reaction mixture was filtered through a pad of Celite, and the filtrate was washed with brine. The organic layer was dried over Na_2_SO_4_, filtered and concentrated. The residue was purified with silica gel chromatography to give the corresponding products **5l** as a yellow oil (1.0 g, 41% yield).

***Tert*-butyl (1-(1-benzamido-4-(diethylamino)-3,3-difluoro-4-oxobutyl)piperidin-4-yl)carbamate (5m).** The product (103.0 mg, 52% yield) as a white solid (m.p. 85.5 – 86.8 ℃) was purified with silica gel chromatography (Petroleum ether/Ethyl acetate = 3/2). ^1^H NMR (600 MHz, Acetone-*d*_6_) δ 7.88 (d, *J* = 7.6 Hz, 2H), 7.82 – 7.75 (m, 1H), 7.51 (t, *J* = 7.3 Hz, 1H), 7.45 (t, *J* = 7.3 Hz, 2H), 5.89 (s, 1H), 5.43 (q, *J* = 7.5 Hz, 1H), 3.56 (dt, *J* = 14.4, 6.9 Hz, 1H), 3.49 (dt, *J* = 14.3, 7.0 Hz, 1H), 3.43 (dt, *J* = 13.9, 6.9 Hz, 1H), 3.35 – 3.23 (m, 2H), 3.00 (d, *J* = 11.3 Hz, 1H), 2.93 (d, *J* = 11.8 Hz, 1H), 2.85 (d, *J* = 34.7 Hz, 1H), 2.80 – 2.66 (m, 2H), 2.46 (t, *J* = 11.5 Hz, 1H), 2.33 (t, *J* = 11.2 Hz, 1H), 1.89 – 1.76 (m, 2H), 1.48 (q, *J* = 16.0, 13.1 Hz, 1H), 1.37 (s, 9H), 1.17 (t, *J* = 6.9 Hz, 3H), 1.08 (t, *J* = 7.0 Hz, 3H). ^19^F NMR (565 MHz, Acetone-*d*_6_) δ -98.60 (dt, *J* = 275.3, 16.6 Hz, 1F), -99.87 (dt, *J* = 275.7, 16.4 Hz, 1F). ^13^C NMR (151 MHz, Acetone-*d*_6_) δ 168.3, 164.0 (t, *J* = 28.3 Hz), 156.7, 136.6, 132.8, 129.9, 128.9, 120.8 (t, *J* = 254.6 Hz), 79.1, 65.6 (t, *J* = 5.7 Hz), 52.1, 49.6, 45.7, 43.6 (t, *J* = 6.6 Hz), 43.5, 39.6 (t, *J* = 22.7 Hz), 34.1, 33.9, 29.4, 15.6, 13.5. MS (ESI): m/z (%) 1015.6 (100, [2M+Na]^+^), 497.3 ([M+H]^+^). HRMS (ESI): Calculated for C_25_H_39_F_2_N_4_O_4_ ([M+H]^+^): 497.2934; Found: 497.2934.

**Gram-scale synthesis of compound 5m**

To a 100 mL of Schlenk tube were added enamide **1** (7 mmol, 1.0 equiv.), Zn (14 mmol, 2 equiv.), *N*-benzoyloxyamine **3m** (7.7 mmol, 1.1 equiv.), 2,2'-bpy (5 mol%), Ni(dppp)Cl_2_ (2.5 mol%) and MgCl_2_ (17.5 mmol, 2.5 equiv.). The tube was evacuated and backfilled with argon for 3 times, then fluotoalkyl chloride **2** (9.8 mmol, 1.4 equiv.) and dry DMA (30 mL) were added via syringe under Ar, and the tube was sealed with Teflon cap. The resulting mixture was stirred for 12 hours at room temperature. After stirring for 12 h, the reaction mixture was quenched with water and diluted with EtOAc. The reaction mixture was filtered through a pad of Celite, and the filtrate was washed with brine. The organic layer was dried over Na_2_SO_4_, filtered and concentrated. The residue was purified with silica gel chromatography to give the corresponding products **5m** as a white solid (2.1 g, 60% yield).

***N*-(1-(4-(8-chloro-5,6-dihydro-11H-benzo[5,6]cyclohepta[1,2-b]pyridin-11-ylidene)piperidin-1-yl)-4-(diethylamino)-3,3-difluoro-4-oxobutyl)benzamide (8a).** The product (125.5 mg, 52% yield) as a yellow solid (m.p. 100.7 – 101.5 ℃) was purified with silica gel chromatography (Petroleum ether/Ethyl acetate = 1/4). ^1^H NMR (600 MHz, Acetone-*d_6_*) δ 8.34 (d, *J* = 4.6 Hz, 1H), 7.88 (d, *J* = 7.7 Hz, 2H), 7.75 (t, *J* = 8.8 Hz, 1H), 7.51 (q, *J* = 7.7, 7.0 Hz, 2H), 7.44 (t, *J* = 7.4 Hz, 2H), 7.24 – 7.07 (m, 4H), 5.46 (q, *J* = 8.0 Hz, 1H), 3.60 – 3.54 (m, 1H), 3.53 – 3.47 (dt, *J* = 14.0, 6.9 Hz, 1H), 3.46 – 3.40 (dt, *J* = 13.0, 6.5 Hz, 1H), 3.39 – 3.26 (m, 3H), 2.98 – 2.90 (m, 1H), 2.87 – 2.77 (m, 4H), 2.75 – 2.66 (m, 1H), 2.65 – 2.56 (m, 1H), 2.53 – 2.34 (m, 3H), 2.34 – 2.20 (m, 2H), 1.18 (t, *J* = 6.8 Hz, 3H), 1.09 (t, *J* = 7.0 Hz, 3H). ^19^F NMR (565 MHz, Acetone-*d_6_*) δ -98.15 – -98.96 (m, 1F), -99.24 – -100.35 (m, 1F). ^13^C NMR (151 MHz, Acetone-*d_6_*) δ 168.19 (d, *J* = 4.8 Hz), 163.98 (t, *J* = 28.3 Hz), 159.36, 148.10 (d, *J* = 4.6 Hz), 141.94 (d, *J* = 2.6 Hz), 140.08 (d, *J* = 3.3 Hz), 139.94 (d, *J* = 2.0 Hz), 138.66, 136.47, 135.05 (d, *J* = 1.9 Hz), 134.38 (d, *J* = 2.1 Hz), 132.84, 132.57 (d, *J* = 13.1 Hz), 130.50, 129.89, 128.91, 127.25 (d, *J* = 10.3 Hz), 123.69, 120.81 (t, *J* = 254.7 Hz), 65.64 (t, *J* = 5.9 Hz), 52.47 (d, *J* = 10.2 Hz), 50.24 (d, *J* = 16.8 Hz), 43.57 (t, *J* = 6.4 Hz), 43.43 (d, *J* = 3.2 Hz), 39.8 – 39.4 (m), 33.10 (d, *J* = 5.5 Hz), 32.60 (t, *J* = 108.0 Hz), 32.66 (d, *J* = 6.3 Hz), 32.59, 15.61, 13.45. MS (ESI): m/z (%) 1213.5 (100, [2M+H]^+^), 607.3 ([M+H]^+^). HRMS (ESI): Calculated for C_34_H_38_ClF_2_N_4_O_2_ ([M+H]^+^): 607.2646; Found: 607.2646.

***N*-(1-(4-(3-((benzo[d][1,3]dioxol-5-yloxy)methyl)-4-fluorophenyl)piperidin-1-yl)-4-(diethylamino)-3,3-difluoro-4-oxobutyl)benzamide (8b).** The product (89.1 mg, 36% yield) as a yellow solid (m.p. 56.6 – 58.1 ℃) was purified with silica gel chromatography (Petroleum ether/Ethyl acetate = 5/2). ^1^H NMR (600 MHz, Acetone-*d*_6_) δ 7.92 (dd, *J* = 20.3, 8.2 Hz, 3H), 7.53 (t, *J* = 7.3 Hz, 1H), 7.46 (t, *J* = 7.3 Hz, 2H), 7.30 – 7.25 (m, 2H), 7.04 (t, *J* = 8.2 Hz, 2H), 6.66 (d, *J* = 8.4 Hz, 1H), 6.40 (s, 1H), 6.23 – 6.12 (m, 1H), 5.90 (s, 2H), 5.54 (q, *J* = 7.5 Hz, 1H), 3.63 (d, *J* = 9.5 Hz, 1H), 3.59 – 3.50 (m, 3H), 3.47 – 3.41 (m, 1H), 3.40 – 3.30 (m, 2H), 3.09 (d, *J* = 11.5 Hz, 1H), 2.83 (dd, *J* = 33.4, 7.0 Hz, 2H), 2.56 – 2.47 (m, 2H), 2.31 (t, *J* = 10.9 Hz, 1H), 2.16 – 2.10 (m, 1H), 1.86 – 1.73 (m, 2H), 1.20 (t, *J* = 6.9 Hz, 3H), 1.10 (t, *J* = 6.9 Hz, 3H). ^19^F NMR (565 MHz, Acetone-*d*_6_) δ -98.82 (dt, *J* = 276.0, 16.6 Hz, 1F), -99.64 (dt, *J* = 276.1, 17.2 Hz, 1F), -118.33 (p, *J* = 7.5 Hz, 1F). ^13^C NMR (151 MHz, Acetone-*d*_6_) δ 168.2, 164.3 – 163.7 (m), 162.3, 156.3, 149.9, 143.3, 142.1 (d, *J* = 3.0 Hz), 136.5, 132.9, 130.8 (d, *J* = 7.8 Hz), 129.9, 129.0, 120.8 (t, *J* = 254.6 Hz), 116.7 (d, *J* = 21.2 Hz), 109.4, 107.2, 102.8, 99.4, 71.4, 65.9 (t, *J* = 5.8 Hz), 54.5, 50.3, 45.7, 43.7, 43.6 (t, *J* = 6.5 Hz), 43.4, 39.7 (t, *J* = 22.8 Hz), 36.2, 15.6, 13.5. MS (ESI): m/z (%) 1251.6 (100, [2M+Na]^+^), 626.3 ([M+H]^+^). HRMS (ESI): Calculated for C_34_H_39_F_3_N_3_O_5_ ([M+H]^+^): 626.2836; Found: 626.2836.

***N*-(4-(diethylamino)-1-(4-(2-((2,4-dimethylphenyl)thio)phenyl)piperazin-1-yl)-3,3-difluoro-4-oxobutyl)benzamide (8c).** The product (107.5 mg, 45% yield) as a white solid (m.p. 135.7 – 136.7 ℃) was purified with silica gel chromatography (Petroleum ether/Ethyl acetate = 3/1). ^1^H NMR (600 MHz, Acetone-*d*_6_) δ 7.94 (d, *J* = 7.5 Hz, 2H), 7.85 (d, *J* = 8.9 Hz, 1H), 7.53 (t, *J* = 7.3 Hz, 1H), 7.47 (t, *J* = 7.5 Hz, 2H), 7.26 (d, *J* = 7.8 Hz, 1H), 7.11 (d, *J* = 6.5 Hz, 3H), 7.03 (d, *J* = 7.7 Hz, 1H), 6.88 (t, *J* = 6.7 Hz, 1H), 6.51 (d, *J* = 7.7 Hz, 1H), 5.51 (q, *J* = 8.2 Hz, 1H), 2.61 – 2.50 (m, 2H), 3.48 – 3.41 (m, 1H), 3.36 – 3.28 (m, 1H), 3.04 (s, 4H), 2.93 – 2.84 (m, 3H), 2.84 – 2.71 (m, 3H), 2.28 (s, 3H), 2.24 (s, 3H), 1.20 (t, *J* = 7.0 Hz, 3H), 1.11 (t, *J* = 7.0 Hz, 3H). ^19^F NMR (565 MHz, Acetone-*d*_6_) δ -98.53 (dt, *J* = 276.2, 16.3 Hz, 1F), -99.90 (dt, *J* = 276.2, 17.1 Hz, 1F). ^13^C NMR (151 MHz, Acetone-*d*_6_) δ 168.2, 164.0, 151.5, 143.3, 140.6, 137.1, 136.4, 135.5, 133.2, 132.9, 130.0 (d, *J* = 10.9 Hz), 129.4, 129.0, 128.2, 127.6, 125.8, 122.6 – 120.7 (m), 65.5 (t, *J* = 6.0 Hz), 53.3, 43.6 (t, *J* = 6.6 Hz), 43.5, 39.4 (t, *J* = 22.9 Hz), 21.9, 21.4, 15.7, 13.5. MS (ESI): m/z (%) 1211.6 (100, [2M+Na]^+^), 595.3 ([M+H]^+^). HRMS (ESI): Calculated for C_33_H_41_F_2_N_4_O_2_S ([M+H]^+^): 595.2913; Found: 595.2913.

***N*-(1-(4-(8-chlorodibenzo[b,f][1,4]oxazepin-11-yl)piperazin-1-yl)-4-(diethylamino)-3,3-difluoro-4-oxobutyl)benzamide (8d).** The product (147 mg, 60% yield) as a white solid (m.p. 101.4 – 102.2 ℃) was purified with silica gel chromatography (Petroleum ether/Ethyl acetate = 3/1). ^1^H NMR (600 MHz, Acetone-*d*_6_) δ 7.90 (t, *J* = 9.6 Hz, 3H), 7.52 (t, *J* = 7.3 Hz, 2H), 7.45 (t, *J* = 7.6 Hz, 2H), 7.42 – 7.31 (m, 2H), 7.14 – 7.04 (m, 3H), 7.00 – 6.96 (m, 1H), 5.52 (q, *J* = 6.4 Hz, 1H), 3.63 – 3.40 (m, 7H), 3.33 – 3.27 (m, 1H), 2.94 – 2.84 (m, 3H), 2.78 (t, *J* = 11.2 Hz, 3H), 1.18 (t, *J* = 7.0 Hz, 3H), 1.09 (t, *J* = 7.1 Hz, 3H). ^19^F NMR (565 MHz, Acetone-*d*_6_) -98.49 (dt, *J* = 277.1, 15.6 Hz, 1F), -99.89 (dt, *J* = 277.1, 16.8 Hz, 1F). ^13^C NMR (151 MHz, Acetone-*d*_6_) δ 168.4 (d, *J* = 4.5 Hz), 164.0 (t, *J* = 28.2 Hz), 161.0, 160.3, 153.4, 142.2, 136.4, 134.3, 132.9, 131.5, 130.6, 129.9, 129.0, 128.5, 127.3, 126.8, 125.9, 124.5, 121.7, 122.7 – 118.8 (m), 65.6 (t, *J* = 5.9 Hz), 43.6 (t, *J* = 6.5 Hz), 43.4, 39.3 (t, *J* = 22.9 Hz), 15.6, 13.5. MS (ESI): m/z (%) 1219.5 (100, [2M+H]^+^), 610.2 ([M+H]^+^). HRMS (ESI): Calculated for C_32_H_35_ClF_2_N_5_O_3_ ([M+H]^+^): 610.2391; Found: 610.2391.

***N*-(1-(4-(2-(3-cyano-4-isobutoxyphenyl)-4-methylthiazole-5-carbonyl)piperazin-1-yl)-4-(diethylamino)-3,3-difluoro-4-oxobutyl)benzamide (8e).** The product (80.0 mg, 39% yield) as a yellow solid (m.p. 70.4 – 71.3 ℃) was purified with silica gel chromatography (Petroleum ether/Ethyl acetate = 1/3). ^1^H NMR (600 MHz, Acetone-*d*_6_) δ 8.19 (d, *J* = 2.2 Hz, 1H), 8.15 (dd, *J* = 8.8, 2.3 Hz, 1H), 7.92 – 7.88 (m, 3H), 7.55 – 7.51 (m, 1H), 7.48 – 7.42 (m, 2H), 7.32 (d, *J* = 8.9 Hz, 1H), 5.52 – 5.45 (m, 1H), 4.03 (d, *J* = 6.5 Hz, 2H), 3.65 (s, 2H), 3.56 (dt, *J* = 16.0, 7.3 Hz, 3H), 3.50 (dt, *J* = 13.9, 6.9 Hz, 1H), 3.45 – 3.39 (m, 1H), 3.34 – 3.28 (m, 1H), 2.84 – 2.76 (m, 4H), 2.73 – 2.67 (m, 2H), 2.38 (s, 3H), 2.20 – 2.13 (m, 1H), 1.18 (t, *J* = 7.0 Hz, 3H), 1.10 – 1.07 (m, 9H). ^19^F NMR (565 MHz, Acetone-*d*_6_) δ -98.62 (dt, *J* = 277.7, 16.2 Hz, 1F), -99.93 (dt, *J* = 277.8, 17.0 Hz, 1F). ^13^C NMR (151 MHz, Acetone-*d*_6_) δ 168.4, 165.9, 163.9 (t, *J* = 114.0 Hz), 163.7, 163.0, 153.9, 136.3, 134.1, 132.9 (d, *J* = 16.3 Hz), 129.9, 128.9, 127.8, 126.8, 120.7 (t, *J* = 255.0 Hz), 116.8, 115.1, 104.0, 77.0, 65.6, 50.0, 43.5 (t, *J* = 6.4 Hz), 43.4, 39.2 (t, *J* = 22.9 Hz), 29.7, 19.9, 17.3, 15.6, 13.4. MS (ESI): m/z (%) 1361.6 (100, [2M+H]^+^), 681.3 ([M+H]^+^). HRMS (ESI): Calculated for C_35_H_43_F_2_N_6_O_4_S ([M+H]^+^): 681.3029; Found: 681.3029.

***N*-(4-(diethylamino)-3,3-difluoro-1-morpholino-4-oxobutyl)-4-methoxybenzamide (6a).** The product (87.5 mg, 53% yield) as a yellow oil was purified with silica gel chromatography (Petroleum ether/Ethyl acetate = 2/3). ^1^H NMR (600 MHz, Acetone-*d*_6_) δ 7.88 (d, *J* = 7.9 Hz, 2H), 7.68 (d, *J* = 8.6 Hz, 1H), 6.99 (d, *J* = 8.1 Hz, 2H), 5.37 (q, *J* = 8.0 Hz, 1H), 3.85 (s, 3H), 3.63 – 3.47 (m, 6H), 3.45 – 3.38 (m, 1H), 3.33 – 3.26 (m, 1H), 2.78 (dd, *J* = 16.5, 5.4 Hz, 1H), 2.75 – 2.64 (m, 3H), 2.60 – 2.56 (m, 2H), 1.17 (t, *J* = 7.0 Hz, 3H), 1.09 (t, *J* = 7.0 Hz, 3H). ^19^F NMR (565 MHz, Acetone-*d*_6_) δ -98.55 (dt, *J* = 276.5, 16.2 Hz, 1F), -99.85 (dt, *J* = 276.6, 17.0 Hz, 1F). ^13^C NMR (151 MHz, Acetone-*d*_6_) δ 167.8, 164.0 (t, *J* = 28.5 Hz), 163.9, 130.7, 128.6, 122.8 – 118.7 (m), 115.1, 68.2, 65.5 (t, *J* = 6.0 Hz), 56.6, 50.2, 43.6 (t, *J* = 6.6 Hz), 43.4, 39.1 (t, *J* = 23.0 Hz), 15.6, 13.4. MS (ESI): m/z (%) 849.4 (100, [2M+ Na]^+^), 414.2 ([M+H]^+^). HRMS (ESI): Calculated for C_20_H_30_F_2_N_3_O_4_ ([M+H]^+^): 414.2199; Found: 414.2199.

***N*-(4-(diethylamino)-3,3-difluoro-1-morpholino-4-oxobutyl)-4-(trifluoromethyl)benzamide (6b).**

The product (140.5 mg, 78% yield) as a white solid (m.p. 126.1 – 126.8 ℃) was purified with silica gel chromatography (Petroleum ether/Ethyl acetate = 1/1). ^1^H NMR (600 MHz, Acetone-*d*_6_) δ 8.15 – 8.00 (m, 3H), 7.83 (d, *J* = 7.0 Hz, 2H), 5.40 (q, *J* = 8.0 Hz, 1H), 3.63 – 3.48 (m, 6H), 3.45 – 3.39 (m, 1H), 3.35 – 3.23 (m, 1H), 2.76 (dt, *J* = 23.3, 7.2 Hz, 2H), 2.72 – 2.67 (m, 2H), 2.62 – 2.59 (m, 2H), 1.18 (t, *J* = 7.0 Hz, 3H), 1.08 (t, *J* = 7.0 Hz, 3H). ^19^F NMR (565 MHz, Acetone-*d*_6_) δ -63.38, -98.52 (dt, *J* = 277.3, 14.2 Hz, 4F), -100.01 (dt, *J* = 277.3, 17.1 Hz, 1F). ^13^C NMR (151 MHz, Acetone-*d*_6_) δ 167.4, 163.9 (t, *J* = 28.4 Hz), 140.2, 133.9 (q, *J* = 32.2 Hz), 129.8, 127.0 (q, *J* = 3.7 Hz), 125.8 (q, *J* = 271.8 Hz), 122.5 – 118.9 (m), 68.2, 66.0 (t, *J* = 5.7 Hz), 50.1, 43.5 (t, *J* = 6.5 Hz), 43.4, 39.0 (t, *J* = 23.0 Hz), 15.6, 13.4. MS (ESI): m/z (%) 925.4 (100, [2M+ Na]^+^), 452.2 ([M+H]^+^). HRMS (ESI): Calculated for C_20_H_27_F_5_N_3_O_3_ ([M+H]^+^): 452.1967; Found: 452.1967.

***N*-(4-(diethylamino)-3,3-difluoro-1-morpholino-4-oxobutyl)-2,3,4,5,6-pentafluorobenzamide (6c).** The product (50.5 mg, 27% yield) as a yellow oil was purified with silica gel chromatography (Petroleum ether/Ethyl acetate = 3/2). ^1^H NMR (600 MHz, Acetone-*d*_6_) δ 8.28 (d, *J* = 8.7 Hz, 1H), 5.34 (q, *J* = 8.1 Hz, 1H), 3.64 – 3.51 (m, 6H), 3.49 – 3.43 (m, 1H), 3.38 – 3.31 (m, 1H), 2.82 – 2.67 (m, 3H), 2.63 – 2.53 (m, 3H), 1.23 (t, *J* = 7.0 Hz, 3H), 1.13 (t, *J* = 7.0 Hz, 3H). ^19^F NMR (565 MHz, Acetone-*d*_6_) δ -98.18 (dt, *J* = 278.8, 15.5 Hz, 1F), -100.94 (dt, *J* = 279.1, 17.6 Hz, 1F), -142.68 (dd, *J* = 22.1, 6.5 Hz, 2F), -155.13 – -155.30 (m, 1F), -163.22 – -163.34 (m, 2F). ^13^C NMR (151 MHz, Acetone-*d*_6_) δ 163.7 (t, *J* = 28.2 Hz), 158.6, 146.2 – 146.0 (m), 144.6 – 144.1 (m), 142.7 – 142.5 (m), 140.1 – 139.8 (m), 138.5 – 138.2 (m), 120.5 (dd, *J* = 256.3, 254.4 Hz), 114.4 (t, *J* = 21.8 Hz), 68.1, 66.2 (t, *J* = 5.6 Hz), 50.0, 43.6 – 43.4 (m), 43.3, 39.3 – 38.9 (m), 15.5, 13.4. MS (ESI): m/z (%) 969.3 (100, [2M+ Na]^+^), 474.2 ([M+H]^+^). HRMS (ESI): Calculated for C_19_H_23_F_7_N_3_O_3_ ([M+H]^+^): 474.1622; Found: 474.1622.

***Tert*-butyl (4-(diethylamino)-3,3-difluoro-1-morpholino-4-oxobutyl)carbamate (6d).** The product (60.9 mg, 40% yield) as a yellow oil was purified with silica gel chromatography (Petroleum ether/Ethyl acetate = 1/1). ^1^H NMR (600 MHz, Acetone-*d*_6_) δ 6.26 (d, *J* = 7.8 Hz, 1H), 4.80 (q, *J* = 7.4 Hz, 1H), 3.60 – 3.52 (m, 5H), 3.47 (dt, *J* = 13.9, 6.9 Hz, 1H), 3.36 – 3.30 (m, 1H), 2.84 (s, 1H), 2.73 – 2.65 (m, 1H), 2.64 – 2.57 (m, 2H), 2.56 – 2.49 (m, 1H), 2.46 (s, 2H), 1.41 (s, 9H), 1.21 (t, *J* = 7.0 Hz, 3H), 1.14 (t, *J* = 7.0 Hz, 3H). ^19^F NMR (565 MHz, Acetone-*d*_6_) δ -98.34 (dt, *J* = 275.8, 15.6 Hz, 1F), -100.09 (dt, *J* = 275.7, 16.9 Hz, 1F). ^13^C NMR (151 MHz, Acetone-*d*_6_) δ 164.2 – 163.7 (m), 157.1, 122.6 – 118.4 (m), 79.6, 68.2, 67.3, 49.8, 43.7 – 43.6 (m), 43.5, 39.3 (t, *J* = 23.0 Hz), 29.3, 15.7, 13.5. MS (ESI): m/z (%) 380.2 ([M+H]^+^). HRMS (ESI): Calculated for C_17_H_32_F_2_N_3_O_4_ ([M+H]^+^): 380.2355; Found: 380.2308.

# Synthetic applications

**Reduction of compound 4a by sodium borohydride**

In a 50 mL round-bottom flask, **4e** (0.4 mmol, 1.0 equiv.) and ethaol (5 mL) was added. The reaction suspension was stirred and NaBH_4_ (6 mmol, 15 equiv.) was then added in small portions. After the cessation of bubble generation, the reaction mixture is further stirred under reflux at 75℃ for 4 hours, then allowed to warm to room temperature.The mixture was quenched with Saturated ammonium chloride and extracted with ethyl acetate. The organic extracts were washed with brine, dried over anhydrous Na_2_SO_4_ and concentrated, the residue was purified with silica gel chromatography (Petroleum ether/Ethyl acetate = 1/2) to give **9** as a white solid (82 mg, 75% yield). ***N*-(3,3-difluoro-4-(2-hydroxyethoxy)butyl)benzamide (9).** The product (82.0 mg, 75% yield) as a white solid (m.p. 76.9 – 78.9 ℃) was purified with silica gel chromatography (Petroleum ether/Ethyl acetate = 1/2). ^1^H NMR (600 MHz, CDCl_3_) δ 7.75 (d, *J* = 7.4 Hz, 2H), 7.49 (t, *J* = 7.3 Hz, 1H), 7.41 (t, *J* = 7.6 Hz, 2H), 6.75 (s, 1H), 3.97 (d, *J* = 10.7 Hz, 1H), 3.85 – 3.77 (m, 2H), 3.69 (q, *J* = 6.2 Hz, 2H), 3.61 – 3.54 (m, 1H), 3.19 (s, 1H), 3.10 (d, *J* = 13.7 Hz, 1H), 2.87 – 2.78 (m, 1H), 2.34 – 2.22 (m, 2H). ^19^F NMR (565 MHz, CDCl_3_) δ -106.90 – -107.39 (m, 2F). ^13^C NMR (151 MHz, CDCl_3_) δ 168.0, 134.3, 131.8, 128.8, 127.0, 123.3 (t, *J* = 242.6 Hz), 66.0, 64.2 (t, *J* = 32.6 Hz), 52.3, 33.9 (t, *J* = 5.3 Hz), 33.4 (t, *J* = 23.6 Hz). MS (ESI): m/z (%) 274.1 ([M+H]^+^). HRMS (ESI): Calculated for C_13_H_18_F_2_NO_3_ ([M+H]^+^): 274.1249; Found: 274.1249.

**Synthesis of difluoroalkyl ketone 10**

In a 25 mL round-bottom flask, **4e** (0.3 mmol, 1.0 equiv.) was added, and the round-bottom flask was evacuated and backfilled with argon for 3 times. then THF (2 mL) was added under argon, *^n^*BuLi (0.39 mmol, 1.3 equiv.)was added dropwise at -78 ℃ under argon. After addition, the reaction mixture was stirred for 30 min at -78 ℃, then allowed to warm to room temperature.The mixture was quenched with Saturated ammonium chloride and concentrated under reduced pressure, then extracted with ethyl acetate. The organic extracts were washed with brine, dried over anhydrous Na_2_SO_4_ and concentrated, the residue was purified with silica gel chromatography (Petroleum ether/Ethyl acetate = 5/1) to give **10** as a yellow solid (55 mg, 50% yield). ***N*-(3,3-difluoro-1-morpholino-4-oxooctyl)benzamide (10).** The product (55.0 mg, 50% yield) as a yellow solid (m.p. 112.7 – 113.4 ℃) was purified with silica gel chromatography (Petroleum ether/Ethyl acetate = 5/1). ^1^H NMR (600 MHz, Acetone-*d*_6_) δ 7.98 (d, *J* = 8.9 Hz, 1H), 7.89 (dt, *J* = 8.5, 1.6 Hz, 2H), 7.55 – 7.51 (m, 1H), 7.48 – 7.43 (m, 2H), 5.30 – 5.24 (m, 1H), 3.56 – 3.51 (m, 2H), 3.49 – 3.45 (m, 2H), 3.12 – 3.06 (m, 1H), 2.85 – 2.80 (m, 2H), 2.62 – 2.57 (m, 2H), 2.55 – 2.50 (m, 2H), 2.33 – 2.24 (m, 1H), 1.65 – 1.56 (m, 2H), 1.43 – 1.35 (m, 2H), 0.93 (t, *J* = 7.4 Hz, 3H). ^19^F NMR (565 MHz, Acetone-*d*_6_) δ -99.37 (m, *J* = 268.5, 15.1, 5.4 Hz, 1F), -112.94 (m, *J* = 268.6, 28.0, 8.3 Hz, 1F). ^13^C NMR (151 MHz, Acetone-*d*_6_) δ 201.7 (dd, *J* = 34.2, 25.1 Hz), 168.7, 136.1, 133.0, 129.9, 129.0, 119.2 (dd, *J* = 255.1, 247.5 Hz), 67.7, 65.7 (dd, *J* = 10.9, 4.3 Hz), 49.8, 38.4, 37.4 (t, *J* = 23.9 Hz), 26.2, 23.5, 14.9. MS (ESI): m/z (%) 759.4 (100, [2M+ Na]^+^), 369.2 ([M+H]^+^). HRMS (ESI): Calculated for C_19_H_27_F_2_N_2_O_3_ ([M+H]^+^): 369.1984; Found: 369.1984.

**Synthesis of monofluoroolefins 11**

In a 50 mL round-bottom flask, **4e** (0.2 mmol, 1.0 equiv.) was added, and the round-bottom flask was evacuated and backfilled with argon for 3 times. then CH_3_CN (3 mL) and TEA (0.54 mmol, 2.7 equiv.) was added under argon, the reaction mixture was stirred for 6 hours at 60 ℃, then allowed to warm to room temperature.The mixture was quenched with water and extracted with ethyl acetate. The organic extracts were washed with brine, dried over anhydrous Na_2_SO_4_ and concentrated, the residue was purified with silica gel chromatography (Petroleum ether/Ethyl acetate = 5/1) to give **11a** as a yellow solid (47 mg, 62% yield). **(Z)-*N*-(3-fluoro-1,4-dimorpholino-4-oxobut-2-en-1-yl)benzamide (11a).** The product (47.0 mg, 62% yield) as a yellow solid (m.p. 58.1 – 58.9 ℃) was purified with silica gel chromatography (Petroleum ether/Ethyl acetate = 1/1). ^1^H NMR (600 MHz, Acetone-*d*_6_) δ 8.15 (d, *J* = 8.6 Hz, 1H), 7.94 – 7.91 (m, 2H), 7.55 – 7.52 (m, 1H), 7.46 (t, *J* = 7.6 Hz, 2H), 6.00 – 5.90 (m, 1H), 5.84 – 5.80 (m, 1H), 3.65 – 3.62 (m, 5H), 3.62 – 3.59 (m, 7H), 2.68 – 2.61 (m, 4H). ^19^F NMR (565 MHz, Acetone-*d*_6_) δ -113.78 (d, *J* = 35.7 Hz, 1F). ^13^C NMR (151 MHz, Acetone-*d*_6_) δ 168.1 (d, *J* = 9.7 Hz), 162.0 (d, *J* = 29.5 Hz), 153.7 (d, *J* = 272.3 Hz), 136.1 (d, *J* = 4.5 Hz), 133.1, 129.9, 129.0, 114.3 (dd, *J* = 9.1, 4.9 Hz), 68.1, 68.0, 65.2 (d, *J* = 5.0 Hz), 65.1 (d, *J* = 5.1 Hz), 50.5. MS (ESI): m/z (%) 777.4 (100, [2M+ Na]^+^), 378.2 ([M+H]^+^). HRMS (ESI): Calculated for C_19_H_25_FN_3_O_4_ ([M+H]^+^): 378.1824; Found: 378.1824.

In a 50 mL round-bottom flask, **4s** (0.2 mmol, 1.0 equiv.) was added, and the round-bottom flask was evacuated and backfilled with argon for 3 times. then CH_3_CN (3 mL) and TEA (0.54 mmol, 2.7 equiv.) was added under argon, the reaction mixture was stirred for 6 hours at 60 ℃, then allowed to warm to room temperature.The mixture was quenched with water and extracted with ethyl acetate. The organic extracts were washed with brine, dried over anhydrous Na_2_SO_4_ and concentrated, the residue was purified with silica gel chromatography (Petroleum ether/Ethyl acetate = 5/1) to give **11b** as a yellow oil (43 mg, 64% yield). **Ethyl (Z)-4-benzamido-2-fluoro-4-morpholinobut-2-enoate (11b).** The product (43.0 mg, 64% yield) as a yellow oil was purified with silica gel chromatography (Petroleum ether/Ethyl acetate = 1/1). ^1^H NMR (600 MHz, Acetone-*d*_6_) δ 7.93 (d, *J* = 7.2 Hz, 2H), 7.57 – 7.52 (m, 1H), 7.47 (t, *J* = 7.7 Hz, 2H), 6.51 – 6.39 (m, 1H), 5.91 – 5.82 (m, 1H), 4.28 (q, *J* = 7.1 Hz, 2H), 3.65 – 3.58 (m, 4H), 2.88 (s, 1H), 2.70 – 2.61 (m, 4H), 1.29 (t, *J* = 7.1 Hz, 3H). ^19^F NMR (565 MHz, Acetone-*d*_6_) δ -124.85 (d, *J* = 32.5 Hz, 1F). ^13^C NMR (151 MHz, Acetone-*d*_6_) δ 168.2, 161.6 (d, *J* = 35.6 Hz), 149.8 (d, *J* = 262.4 Hz), 136.0, 133.1, 130.0, 129.0, 117.8 (d, *J* = 9.0 Hz), 68.1, 65.3 (d, *J* = 3.4 Hz), 63.3, 50.5, 15.1. MS (ESI): m/z (%) 695.3 (100, [2M+ Na]^+^), 337.2 ([M+H]^+^). HRMS (ESI): Calculated for C_17_H_22_FN_2_O_4_ ([M+H]^+^): 337.1558; Found: 337.1558.

**Synthesis of compound 12**

In a 50 mL round-bottom flask, **4s** (0.3 mmol, 1.0 equiv.) and DMAP (0.45 mmol, 1.5 equiv.)was added, and the round-bottom flask was evacuated and backfilled with argon for 3 times. then CH_3_CN (4 mL) and TEA (0.9 mmol, 3 equiv.) was added under argon, the reaction mixture was stirred for 6 hours at 60 ℃, then allowed to warm to room temperature.The mixture was quenched with water and extracted with ethyl acetate. The organic extracts were washed with brine, dried over anhydrous Na_2_SO_4_ and concentrated, the residue was purified with silica gel chromatography (Petroleum ether/Ethyl acetate = 2/1) to give **12** as a yellow oil (65 mg, 65% yield). **Ethyl (E)-benzoyl(3-morpholino-3-oxoprop-1-en-1-yl)carbamate (12).** The product (65.0 mg, 65% yield) as a yellow oil was purified with silica gel chromatography (Petroleum ether/Ethyl acetate = 2/1). ^1^H NMR (600 MHz, Acetone-*d*_6_) δ 8.08 – 8.05 (m, 2H), 7.55 – 7.49 (m, 2H), 7.44 – 7.40 (m, 2H), 6.05 (d, *J* = 16.3 Hz, 1H), 4.16 (q, *J* = 7.1 Hz, 2H), 3.79 – 3.73 (m, 8H), 1.23 (t, *J* = 7.1 Hz, 3H). ^13^C NMR (151 MHz, Acetone-*d*_6_) δ 176.9, 166.0, 162.4, 139.7, 138.8, 133.2, 131.0, 129.6, 127.3, 67.7, 62.2, 48.0, 15.2. MS (ESI): m/z (%) 317.2 (100). HRMS (ESI): Calculated for C_17_H_20_N_2_O_5_ (M^+^): 332.1367; Found: 332.1367.

# Mechanistic Studies

**Radical inhibition experiment**

**Procedure**: To a 25 mL Schlenck tube were added enamide **1a** (0.4 mmol, 1.0 equiv.), zinc powder (0.8 mmol, 2.0 equiv.), *N*-benzoyloxyamine **3a** (0.44 mmol, 1.1 equiv.), **L1** (5 mol%), Ni(dppp)Cl_2_ (2.5 mol%) and MgCl_2_ (1.0 mmol, 2.5 equiv.) and TEMPO (0.2 equiv or 1.0 equiv.) in the air. The tube was evacuated and backfilled with argon for 3 times, then fluotoalkyl chloride **2a** (0.56 mmol, 1.4 equiv.) and dry DMA (3 mL) were added via syringe under Argon, and the tube was sealed with Teflon cap. The resulting mixture was stirred for 12 hours at room temperature. After stirring for 12 h, the reaction mixture was diluted with ethyl acetate. The yield was determined by ^19^F-NMR with fluorobenzene as the internal standard. When 0.2 equiv of TEMPO was added, the yield of product **4a** was 38%. When 1.0 equiv of TEMPO was added, **4a** was not detected.

**Procedure**: To a 25 mL Schlenck tube were added enamide **1a** (0.4 mmol, 1.0 equiv.), zinc powder (0.8 mmol, 2.0 equiv.), *N*-benzoyloxyamine **3a** (0.44 mmol, 1.1 equiv.), **L1** (5 mol%), Ni(dppp)Cl_2_ (2.5 mol%) and MgCl_2_ (1.0 mmol, 2.5 equiv.) and 1,4-dinitrobenzene (0.2 equiv. or 1.0 equiv.) in the air. The tube was evacuated and backfilled with argon for 3 times, then fluotoalkyl chloride **2a** (0.56 mmol, 1.4 equiv.) and dry DMA (3 mL) were added via syringe under Argon, and the tube was sealed with Teflon cap. The resulting mixture was stirred for 12 hours at room temperature. After stirring for 12 h, the reaction mixture was diluted with ethyl acetate. The yield was determined by ^19^F-NMR with fluorobenzene as the internal standard. When 0.2 equiv of 1,4-dinitrobenzene was added, the product **4a** was not detected. When 1.0 equiv of 1,4-dinitrobenzene was added, **4a** was not detected.

**Radical trapping experiment**

**Procedure**: To a 25 mL Schlenck tube were added enamide **1a** (0.4 mmol, 1.0 equiv.), zinc powder (0.8 mmol, 2.0 equiv.), *N*-benzoyloxyamine **3a** (0.44 mmol, 1.1 equiv.), **L1** (5 mol%), Ni(dppp)Cl_2_ (2.5 mol%) and MgCl_2_ (1.0 mmol, 2.5 equiv.) in the air. The tube was evacuated and backfilled with argon for 3 times, then fluotoalkyl chloride **2a** (0.56 mmol, 1.4 equiv.), compound **13** (0.4 mmol, 1.0 equiv.) and dry DMA (3 mL) were added via syringe under Argon, and the tube was sealed with Teflon cap. The resulting mixture was stirred for 12 hours at room temperature. After stirring for 12 h, the reaction mixture was quenched with water and diluted with EtOAc. The reaction mixture was filtered through a pad of Celite, and the filtrate was washed with brine. The organic layer was dried over Na_2_SO_4_, filtered and concentrated. The residue was purified with silica gel chromatography to give the corresponding product **14** as a colorless oil (20 mg, 17% yield). **3-(3,4-dihydronaphthalen-1-yl)-*N*,*N*-diethyl-2,2-difluoropropanamide (14).** The product (19.7 mg, 17% yield) as a colorless oil was purified with silica gel chromatography (Petroleum ether/Ethyl acetate = 10/1). ^1^H NMR (600 MHz, Acetone-*d*_6_) δ 7.31 (d, *J* = 7.7 Hz, 1H), 7.21 – 7.17 (m, 1H), 7.14 (q, *J* = 3.6, 2.9 Hz, 2H), 6.11 (t, *J* = 4.5 Hz, 1H), 3.44 (q, *J* = 7.0 Hz, 2H), 3.38 – 3.31 (m, 4H), 2.74 (t, *J* = 8.0 Hz, 2H), 2.29 – 2.27 (m, 2H), 1.13 (t, *J* = 7.0 Hz, 3H), 1.05 (t, *J* = 7.1 Hz, 3H). ^19^F NMR (565 MHz, Acetone-*d*_6_) δ -98.47 (t, *J* = 18.0 Hz, 2F). ^13^C NMR (151 MHz, Acetone-*d*_6_) δ 162.5 (t, *J* = 28.5 Hz), 136.2, 134.6, 131.5, 127.7 – 126.0 (m), 123.3, 118.6 (t, *J* = 254.5 Hz), 65.0, 41.7 (t, *J* = 6.4 Hz), 41.4, 37.1 (t, *J* = 24.1 Hz), 27.8, 23.0, 13.9, 11.6.

**Procedure**: To a 25 mL Schlenck tube were added enamide **1a** (0.4 mmol, 1.0 equiv.), zinc powder (0.8 mmol, 2.0 equiv.), *N*-benzoyloxyamine **3a** (0.44 mmol, 1.1 equiv.), **L1** (5 mol%), Ni(dppp)Cl_2_ (2.5 mol%) and MgCl_2_ (1.0 mmol, 2.5 equiv.) in the air. The tube was evacuated and backfilled with argon for 3 times, then fluotoalkyl chloride **2a** (0.56 mmol, 1.4 equiv.), compound **15** (0.4 mmol, 1.0 equiv.) and dry DMA (3 mL) were added via syringe under Argon, and the tube was sealed with Teflon cap. The resulting mixture was stirred for 12 hours at room temperature. After stirring for 12 h, the reaction mixture was quenched with water and diluted with EtOAc. The reaction mixture was filtered through a pad of Celite, and the filtrate was washed with brine. The organic layer was dried over Na_2_SO_4_, filtered and concentrated. The residue was purified with silica gel chromatography to give the corresponding product **16** as a white solid (34 mg, 26% yield). ***N*,*N*-diethyl-2,2-difluoro-4,4-diphenylbutanamide (16).** The product (34.2 mg, 26% yield) as a white solid (m.p. 70.5 – 70.8 ℃) was purified with silica gel chromatography (Petroleum ether/Ethyl acetate = 10/1). ^1^H NMR (600 MHz, Acetone-*d*_6_) δ 7.40 (d, *J* = 7.3 Hz, 4H), 7.28 (t, *J* = 7.7 Hz, 4H), 7.17 (t, *J* = 7.4 Hz, 2H), 4.45 (t, *J* = 7.0 Hz, 1H), 3.44 (q, *J* = 7.0 Hz, 2H), 3.32 (q, *J* = 7.1 Hz, 2H), 3.12 – 3.00 (m, 2H), 1.10 (t, *J* = 7.0 Hz, 3H), 1.06 (t, *J* = 7.1 Hz, 3H). ^19^F NMR (565 MHz, Acetone-*d*_6_) δ -98.40 (t, *J* = 17.7 Hz, 2F). ^13^C NMR (151 MHz, Acetone-*d*_6_) δ 163.8 (t, *J* = 28.6 Hz), 146.3, 130.0, 129.3, 127.9, 120.9 (t, *J* = 254.9 Hz), 46.7 (t, *J* = 3.8 Hz), 43.3 (t, *J* = 6.1 Hz), 42.9, 41.9 (t, *J* = 22.4 Hz), 15.4, 13.4. MS (ESI): m/z (%) 685.3 (100, [2M+Na]^+^), 354.2 ([M+Na]^+^). HRMS (ESI): Calculated for C_20_H_23_F_2_NONa ([M+Na]^+^): 354.1640; Found: 354.1640.

**Reaction of difluoroacetamide chloride 2a with zinc powder**

**Procedure**: To a 25 mL of Schlenk tube were added zinc powder (0.8 mmol), MgCl_2_ (1 mmol). The mixture was evacuated and backfilled with argon for three times, then fluotoalkyl chloride **2a** (0.56 mmol) and dry DMA (2 mL) were added via syringe under Argon, and the tube was sealed with Teflon cap. The resulting mixture was stirred for 1 hour at room temperature. After stirring for 1 h, the ^19^F NMR of reaction mixture showed no difluoromethylzinc species were formed and only HCF_2_CONEt_2_ were observed.

**Procedure**: To a 25 mL of Schlenk tube were added Ni(dppp)Cl_2_ (0.025 mmol), **L1** (0.5 mmol), zinc powder (0.8 mmol) and MgCl_2_ (1 mmol). The mixture was evacuated and backfilled with argon for three times, then fluotoalkyl chloride **2a** (0.56 mmol) and dry DMA (2 mL) were added via syringe under Argon, and the tube was sealed with Teflon cap. The resulting mixture was stirred for 1 hour at room temperature. After stirring for 1 h, the ^19^F NMR of reaction mixture showed no difluoromethylzinc species were formed and only HCF_2_CONEt_2_ were observed.

**Table S9.** Control experiment: reaction performed using Ni(COD)_2_ as the catalyst. *^a^*

| Entry | Ni(COD)_2_(x mol%) | **L1** (y mol%) | Yield of **4a,** (%)*^b^* with Zn (2.0 equiv.) | Yield of **4a,** (%)*^b^* without Zn (2.0 equiv.) |
| --- | --- | --- | --- | --- |
| 1 | 2.5 | 5 | 89 | ND |
| 2 | 50 | 100 | 53 | ND |
| 3 | 100 | 200 | 53 | 23 |

*^a^*Reaction conditions (unless otherwise specified): **1a** (0.4 mmol, 1.0 equiv.), **2a** (1.4 equiv.), **3a** (1.1 equiv.) and DMA (3 mL). *^b^*Determined by ^19^F NMR using fluorobenzene (0.4 mmol) as an internal standard. The yield of the products and recovery yield of **2a** are calculated using **1a** as reference substance. ND, not detected.

**Procedure**: Experiments were conducted inside of a glovebox filled with argon atmosphere. To a 25 mL Schlenck tube were added enamide **1a** (0.4 mmol, 1.0 equiv.), zinc powder (0.8 mmol, 2.0 equiv.), *N*-benzoyloxyamine **3a**, **L1** (5 /100 /200 mol%), Ni(COD)_2_ (2.5 /50 /100 mol%) and MgCl_2_ (1.0 mmol, 2.5 equiv.), then fluotoalkyl chloride **2a** (0.56 mmol, 1.4 equiv.), and dry DMA (3 mL) were added via syringe , and the tube was sealed with Teflon cap. The resulting mixture was stirred for 12 hours at room temperature. After stirring for 12 h, the reaction mixture was diluted with ethyl acetate. The yield was determined by ^19^F-NMR with fluorobenzene as the internal standard.

**Kinetic studies using initial-rate method**

**General Procedure for Kinetic Experiments:**

**Procedure**: To a 25 mL Schlenck tube were added enamide **1a**, zinc powder, *N*-benzoyloxyamine **3a**, **L1**, Ni(dppp)Cl_2_ and MgCl_2_ in the air. The tube was evacuated and backfilled with argon for 3 times, then fluotoalkyl chloride **2a** and dry DMA (3 mL) were added via syringe under argon, and the tube was sealed with Teflon cap. Aliquots (100 μL) were removed at predetermined time points with a Luer microliter syringe, the aliquots were diluted with ethyl acetate. The yield was determined by ^19^F-NMR with fluorobenzene as the internal standard.

(1) Full time course for standard reaction

**Figure S4.** Full Time Kinetics

**Table S10. Full time kinetic data**

| **Time** | **Product (mM)** |
| --- | --- |
| 35 min | 120 |
| 40 min | 240 |
| 50 min | 440 |
| 60 min | 640 |
| 70 min | 800 |
| 80 min | 920 |
| 90 min | 1140 |
| 100 min | 1360 |
| 110 min | 1600 |
| 120 min | 1760 |
| 130 min | 1960 |

(2) Kinetic data for different [**1a**]_0_

**Table S11.** Initial [**1a**] (mmol)

|  | **Initial [1a] (mmol)** | | | |
| --- | --- | --- | --- | --- |
|  | 0.3 mmol | 0.4 mmol | 0.5 mmol | 0.6 mmol |
| **Time** | **Product (mM)** | **Product (mM)** | **Product (mM)** | **Product (mM)** |
| 40 min | 180 | 440 | 0 | 60 |
| 50 min | 285 | 600 | 200 | 300 |
| 60 min | 360 | 760 | 500 | 600 |
| 70 min | 435 | 960 | 750 | 900 |
| 80 min | 525 | 1200 | 1050 | 1290 |

**Figure S5.** Kinetics (different [1a]_0_)

(3) Kinetic data for different [**2a**]_0_

**Table S12.** Initial [**2a**] (mmol)

|  | **Initial [2a] (mmol)** | | | |
| --- | --- | --- | --- | --- |
|  | 0.46 mmol | 0.56 mmol | 0.66 mmol | 0.76 mmol |
| **Time** | **Product (mM)** | **Product (mM)** | **Product (mM)** | **Product (mM)** |
| 40 min | 280 | 0 | 340 | 0 |
| 50 min | 520 | 120 | 560 | 0 |
| 60 min | 720 | 400 | 720 | 40 |
| 70 min | 1000 | 600 | 880 | 260 |
| 80 min | 1300 | 760 | 1040 | 500 |

**Figure S6.** Kinetics (different [2a]_0_)

(4) Kinetic data for different [**3a**]_0_

**Table S13.** Initial [**3a**] (mmol)

|  | **Initial [3a] (mmol)** | | | |
| --- | --- | --- | --- | --- |
|  | 0.36 mmol | 0.44 mmol | 0.54 mmol | 0.64 mmol |
| **Time** | **Product (mM)** | **Product (mM)** | **Product (mM)** | **Product (mM)** |
| 40 min | 0 | 240 | 0 | 160 |
| 50 min | 260 | 400 | 240 | 360 |
| 60 min | 520 | 640 | 440 | 480 |
| 70 min | 800 | 840 | 600 | 600 |
| 80 min | 1120 | 980 | 720 | 680 |

**Figure S7.** Kinetics (different [3a]_0_)

(5) Kinetic data for different [MgCl_2_]_0_

**Table S14.** Initial [MgCl_2_] (mmol)

|  | **Initial [MgCl_2_] (mmol)** | | | |
| --- | --- | --- | --- | --- |
|  | 0.6 mmol | 0.8 mmol | 1.0 mmol | 1.2 mmol |
| **Time** | **Product (mM)** | **Product (mM)** | **Product (mM)** | **Product (mM)** |
| 40 min | 0 | 0 | 0 | 0 |
| 50 min | 160 | 0 | 180 | 0 |
| 60 min | 280 | 160 | 400 | 200 |
| 70 min | 400 | 340 | 580 | 400 |
| 80 min | 460 | 480 | 760 | 600 |

**Figure S8.** Kinetics (different [MgCl_2_]_0_)

(6) Kinetic data for different n_0_(**Zn)**

**Table S15.** Initial n(**Zn)** (mmol)

|  | **Initial** n(**Zn) (mmol)** | | | |
| --- | --- | --- | --- | --- |
|  | 0.4 mmol | 0.6 mmol | 0.8 mmol | 1.0 mmol |
| **Time** | **Product (mM)** | **Product (mM)** | **Product (mM)** | **Product (mM)** |
| 40 min | 0 | 160 | 0 | 280 |
| 50 min | 0 | 320 | 120 | 520 |
| 60 min | 0 | 520 | 340 | 780 |
| 70 min | 100 | 720 | 600 | 1180 |
| 80 min | 160 | 1000 | 920 | 1540 |

**Figure S9.** Kinetics (different [Zn]_0_)

(7) Kinetic data for different [Ni/**L1**]_0_

**Table S16.** Initial [Ni/**L1**] (mmol)

|  | **Initial [Ni]/L (mmol)** | | | |
| --- | --- | --- | --- | --- |
|  | 0.01/0.02 mmol | 0.015/0.03 mmol | 0.02/0.04 mmol | 0.025/0.05 mmol |
| **Time** | **Product (mM)** | **Product (mM)** | **Product (mM)** | **Product (mM)** |
| 40 min | 0 | 240 | 40 | 680 |
| 50 min | 60 | 440 | 160 | 900 |
| 60 min | 180 | 560 | 440 | 1160 |
| 70 min | 300 | 700 | 640 | 1440 |
| 80 min | 400 | 840 | 840 | 1640 |

**Figure S10.** Kinetics (different [Ni/L]_0_)

(8) Kinetic data for different [Ni]_0_

**Table S17.** Initial [Ni] (mmol)

|  | **Initial [Ni] (mmol)** | | | |
| --- | --- | --- | --- | --- |
|  | 0.01 mmol | 0.015 mmol | 0.02 mmol | 0.025 mmol |
| **Time** | **Product (mM)** | **Product (mM)** | **Product (mM)** | **Product (mM)** |
| 40 min | 120 | 400 | 880 | 840 |
| 50 min | 280 | 560 | 1560 | 1400 |
| 60 min | 360 | 740 | 2040 | 1800 |
| 70 min | 440 | 800 | 2220 | 2020 |
| 80 min | 580 | 1200 | 2380 | 2180 |

**Figure S11.** Kinetics (different [Ni]_0_)

(9) Kinetic data for different [**L1**]_0_

**Table S18.** Initial [**L1**] (mmol)

|  | **Initial [L1] (mmol)** | | | |
| --- | --- | --- | --- | --- |
|  | 0.01 mmol | 0.02 mmol | 0.03 mmol | 0.04 mmol |
| **Time** | **Product (mM)** | **Product (mM)** | **Product (mM)** | **Product (mM)** |
| 40 min | 0 | 0 | 40 | 80 |
| 50 min | 140 | 160 | 200 | 240 |
| 60 min | 300 | 260 | 320 | 360 |
| 70 min | 680 | 400 | 460 | 520 |
| 80 min | 860 | 480 | 560 | 620 |

**Figure S12.** Kinetics (different [L]_0_)

**Hammett analysis of nitrogen electrophiles 3**

**Table S19.** Kinetic data for different **3**.

|  | **Kinetic data for different 3** | | | | |
| --- | --- | --- | --- | --- | --- |
|  | R=-NMe_2_ | R=-OMe | R=-H | R=-F | R=-CF_3_ |
| **Time** | **Product (mM)** | **Product (mM)** | **Product (mM)** | **Product (mM)** | **Product (mM)** |
| 40 min | 0 | 120 | 240 | 520 | 120 |
| 50 min | 120 | 360 | 440 | 1000 | 440 |
| 60 min | 360 | 520 | 640 | 1360 | 1060 |
| 70 min | 440 | 680 | 800 | 1720 | 1520 |
| 80 min | 560 | 820 | 920 | 1940 | 1840 |

**Figure S13.** Initial rates for different **3**

**Hammett analysis of ligands**

**Table S20.** Kinetic data for different **L**.

|  | **Kinetic data for different Ligand** | | | | |
| --- | --- | --- | --- | --- | --- |
|  | R=-NMe_2_ | R=-OMe | R=-H | R=-F | R=-CF_3_ |
| **Time** | **Product (mM)** | **Product (mM)** | **Product (mM)** | **Product (mM)** | **Product (mM)** |
| 40 min | 0 | 0 | 240 | 0 | 40 |
| 50 min | 0 | 0 | 440 | 0 | 120 |
| 60 min | 0 | 40 | 640 | 0 | 240 |
| 70 min | 0 | 120 | 800 | 0 | 380 |
| 80 min | 0 | 260 | 920 | 0 | 480 |

**Figure S14.** Initial rates for different **L**

***General Procedure for Arrhenius plot experiments:***

**Procedure**: To a 25 mL Schlenck tube were added enamide **1a** (0.4 mmol, 1.0 equiv.), zinc powder (0.8 mmol, 2.0 equiv.), *N*-benzoyloxyamine **3a** (0.44 mmol, 1.1 equiv.), **L1** (5 mol%), Ni(dppp)Cl_2_ (2.5 mol%) and MgCl_2_ (1.0 mmol, 2.5 equiv.) in the air. The tube was evacuated and backfilled with argon for 3 times, then fluotoalkyl chloride **2a** (0.56 mmol, 1.4 equiv.) and dry DMA (3 mL) were added via syringe under Argon, and the tube was sealed with Teflon cap on a thermo magnetic stirrer. Aliquots (100 μL) were removed at predetermined time points with a Luer microliter syringe, the reaction mixture was diluted with ethyl acetate. The yield was determined by ^19^F-NMR with fluorobenzene as the internal standard.

**Table S21.** Data for Arrhenius plot experiments.

|  | **1a** | **2a** | **3a** | Zn | MgCl_2_ | Ni(dppp)Cl_2_ | Ligand |
| --- | --- | --- | --- | --- | --- | --- | --- |
| **reaction order** | 1.9598 | -0.7945 | -1.2315 | 1.4423 | 0.8043 | 1.7315 | -1.2073 |
| **Volume (mL)** | 0.4 | 0.56 | 0.44 | 1 | 0.8 | 0.01 | 0.02 |
| **Substrate**  **conc. (mmol**  **/L)** | 133.3333 | 186.6667 | 146.6667 | 333.3333 | 266.6667 | 3.3333 | 6.6667 |
| **c^a** | 14603.4321 | 0.0157 | 0.0021 | 4352.5925 | 89.3731 | 8.0420 | 0.1012 |

**Table S22.** Data for Arrhenius plot experiments.

| **r_0_-initial rate mmol/(L·min)** | **Rate constant mmol/(L·min)** | **T (K)** |
| --- | --- | --- |
| 19.2 | 1.23×10^-4^ | 298.15 |
| 61.14 | 3.92×10^-4^ | 308.15 |
| 106.33 | 6.82×10^-4^ | 318.15 |

# Pharmacological tests

**Cell culture:** The RAW264.7 mouse macrophage cell line was obtained from the BeNa Culture Collection. The cells were cultured in a humidified atmosphere with 5% CO_2_ at 37 ℃ using DMEM culture medium supplemented with 10% FBS. Additionally, the culture medium contained penicilin (100 U/mL) and streptomycin (100 μg/mL).

**(1) Cell viability assessment via CCK-8 assay**

**Method:** To evaluate the effect of **4q** on the cell viability of RAW264.7 cells, we utilized the Cell Counting Kit-8 (CCK-8) assay. The cells were seeded in a 96-well plate at 1×10^5^ cells/mL. After incubating for 24 h, different concentrations of **4q** were added to the cell cultures. Subsequently, after an additional 24 h incubation, 10 μL of the CCK-8 reagent was added to each well. The plated samples were then incubated in a 37 ℃ environment for 2 h. Following this incubation period, the absorbance at 450 nm was quantified using a Scientific Multiskan GO microplate reader.

**Result:** In order to determine the effect of **4q** on cell survival rate, the CCK-8 assay was used. As shown in Figure S15, different concentrations of **4q** showed different effects on the cell viability of RAW264.7 cells. Compared with control group (0 μM), the cell viability was not affected when the concentration of **4q** was at 250 μM (P>0.05). When the concentration of **4q** reached 500 μM, there was a statistically significant decrease on cell viability (P<0.0001), and the cell viability decreased with the concentration increase. Meanwhile, the inhibition rate on cell viability was calculated by SPSS software, and the IC_50_ of **4q** was 577.2 μM. Therefore, **4q** was selected for preliminary exploration of anti-inflammatory activity.


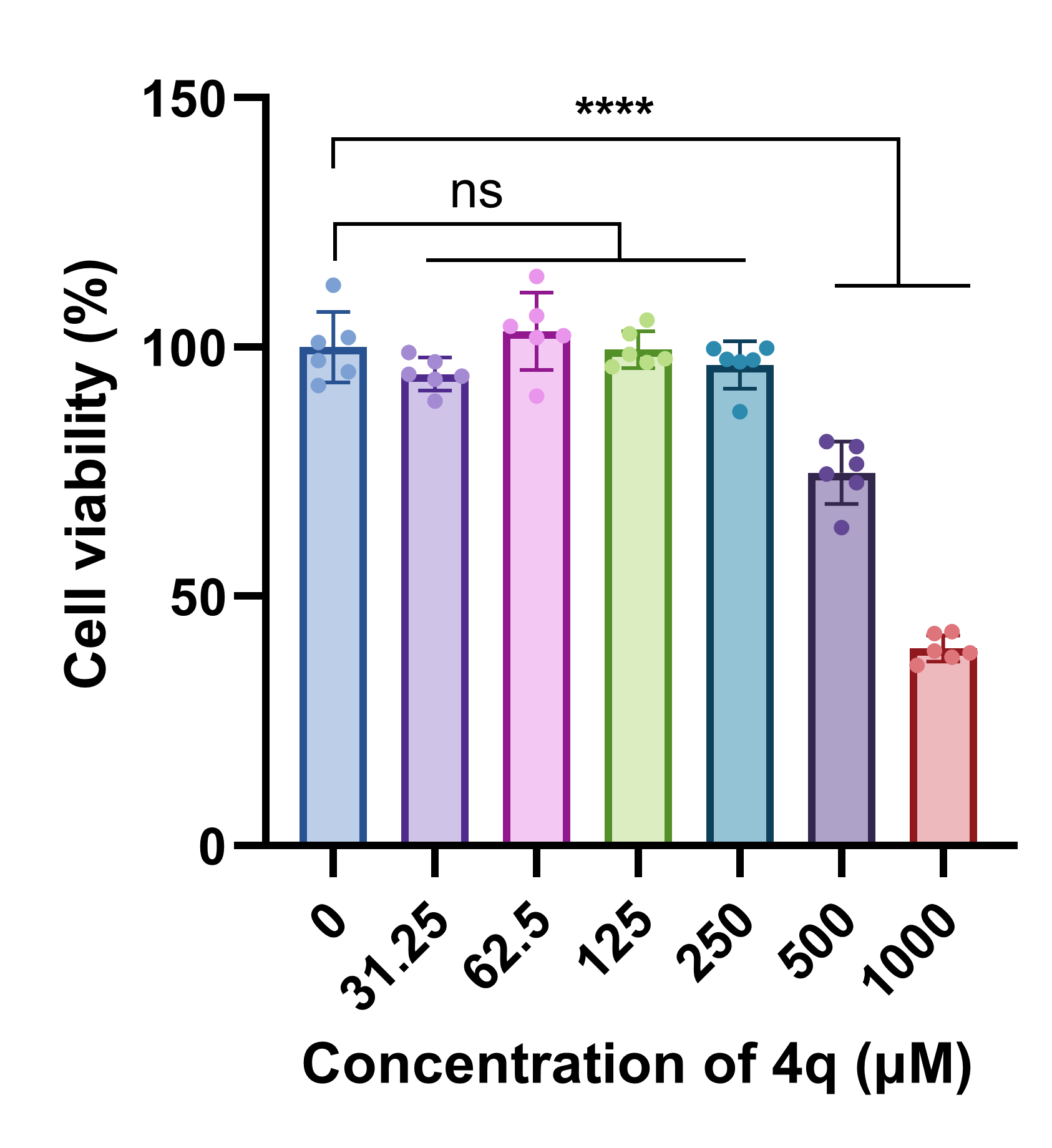


**Figure S15.** Effect of **4q** on the cell viability of RAW264.7 cells. Data are expressed as mean ± SD (n = 6). ****P<0.0001, ns=no significant.

**(2) Detection of nitric oxide (NO)**

**Method:** NO levels were assessed by the NO assay Kit (SOO21, Beyotime Biotechnology Co, Ltd.). Firstly, RAW264.7 cells were cultured in 96-well plates for 24 h. Subsequently, the cells were exposed to varying concentrations of **4q** (0.049 μM to 3.125 μM) with LPS at 1 μg/mL. Dexamethasone (DEX) at 10 μM was selected as a positive control. After 24 h treatment, the supernatants were collected from the cell cultures and introduced Griess Reagent Ⅰ and Griess Reagent Ⅱ sequentially. Following an appropriate incubation time for the chemical reaction, the NO levels were quantified by measuring the absorbance at 540 nm using a microplate reader.

**Result:** The excessive and uncontrolled release of inflammatory cytokines can lead to various inflammatory diseases. Therefore, the effects of different concentrations of **4q** on the production of NO in LPS-induced RAW264.7 cells were further tested. Compared with the LPS group, the production of NO in RAW264.7 macrophages decreased in DEX group and **4q** group (Figure S16). **4q** significantly reduced the production of NO with an IC_50_=0.54 μM, and its inhibitory ability increased with increasing concentration. Results showed that the inhibition rate of **4q** at 1.563 μM against NO production was better than that of DEX at a concentration of 10 μM. Importantly, the selectivity index of **4q** is 1069, which was equal to the IC_50_ of cytotoxicity divided by the IC_50_ of anti-inflammatory activity. The above results indicated that **4q** could effectively inhibit LPS-induced macrophage inflammatory response without cytotoxicity.


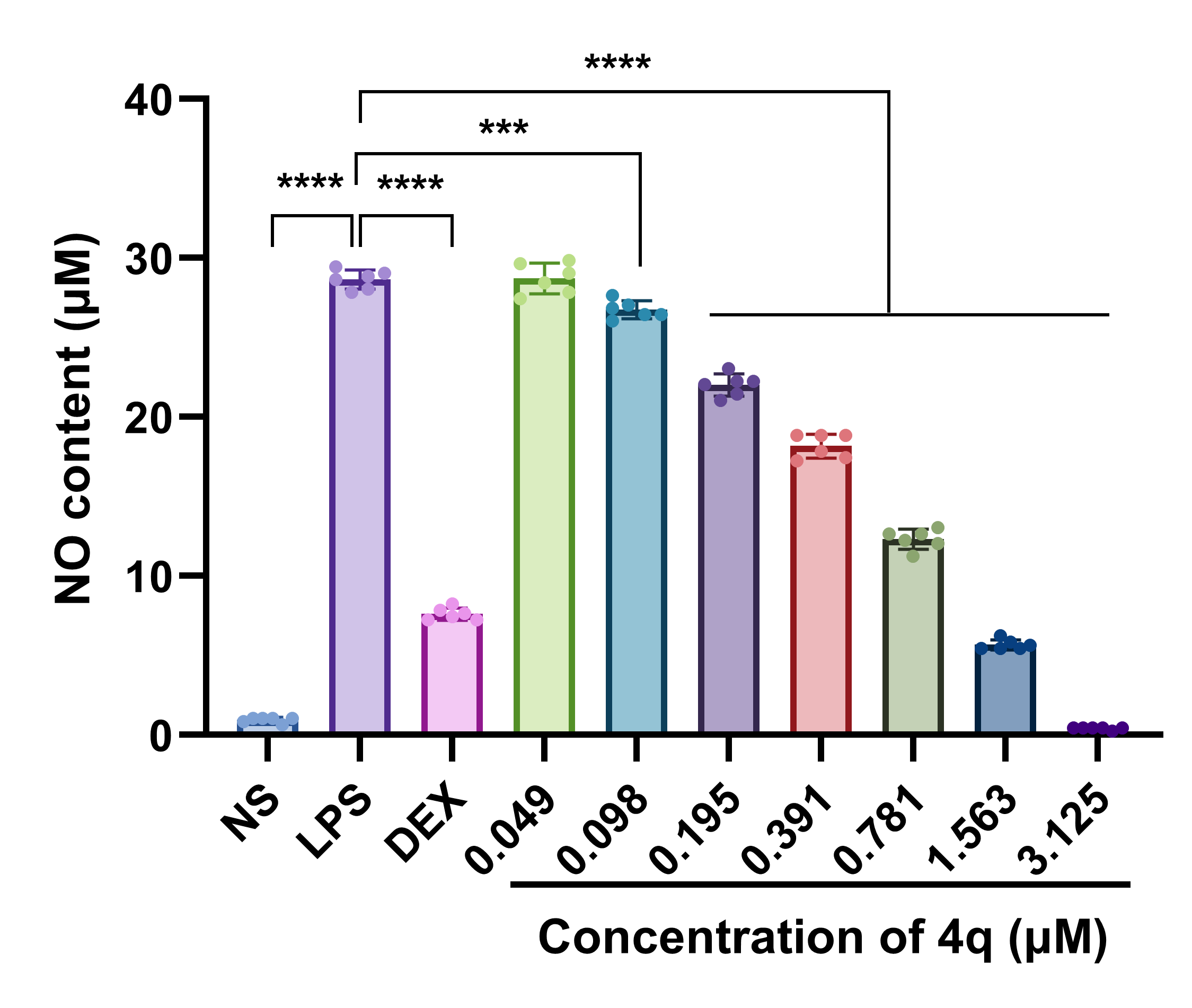


**Figure S16.** Effect of different concentrations of **4q** on LPS-induced NO production in RAW264.7 cells. Values are expressed as mean±SD (n=6). ^***^P<0.001, ^****^P<0.0001.

**(3) In Vivo Anti-Inflammatory Activity of Compound 4q**

**Method**: To evaluate the anti-inflammatory properties of compound **4q** in vivo, a murine model of xylene-induced ear edema was employed. Male Kunming mice (weighing 20–22 g) were obtained from the Kunming Medical Laboratory Animal Center. All experimental procedures were conducted in accordance with the guidelines and regulations approved by the Institutional Animal Care and Use Committee of Kunming Medical University (KMMUD2026036). Following a one-week acclimatization period, the animals were allocated randomly into six experimental cohorts (n=6 per group): a normal (control) group, an untreated (xylene only) group, a positive control group receiving DEX (0.1 mg), and three treatment groups administered compound **4q** at doses of 0.1 mg, 0.2 mg, and 0.4 mg, respectively. Edema was induced by uniformly applying xylene to both the ventral and dorsal surfaces of each mouse's right ear (20 μL per side). After 30 minutes, the right ears of the animals in the treatment groups received topical applications of either compound **4q** or DEX solution (20 μL per side). The left ears remained untreated and served as internal controls. At 1.5 hours post-administration, the mice were humanely euthanized. Both ears were then excised at the base of the pinna. Using a standard biopsy punch, circular tissue sections (8 mm in diameter) were collected from identical anatomical locations on each ear and subsequently weighed. The swelling degree of ears was quantified for each animal as the weight difference between its right (treated) and left (control) ear punches. The anti-edematous effect was further expressed as the swelling inhibition rate, calculated using the following formula:

$$Inhibition Rate \left( \% \right)=\frac{\mathrm{Mean}s\mathrm{welling}d\mathrm{egree}\left( u\mathrm{ntreated}g\mathrm{roup} \right)-Mean s\mathrm{welling}degree(t\mathrm{reatment}group)}{\mathrm{Mean}s\mathrm{welling}degree(u\mathrm{ntreated}group)}\times100\%$$

To obtain deeper histological insight into the anti-inflammatory action of compound **4q**, the ear tissue samples were immediately fixed by immersion in 4% paraformaldehyde for 24 hours. Following fixation, the tissues were embedded in paraffin and sectioned. These sections were then subjected to hematoxylin and eosin (H&E) staining as well as immunohistochemical staining. The stained specimens were examined microscopically to assess the histological inflammation and the modulatory mechanism of the treatment.

**Statistical Analysis:** All experimental values are reported as means accompanied by their standard deviations (SD). Graphical representations were generated utilizing GraphPad Prism (version 10.0.0). To determine statistical significance, a one-way analysis of variance (ANOVA) was employed, with subsequent application of Tukey's multiple comparisons test for more than two groups or unpaired Student’s t-test for two groups. Threshold for statistical significance was established at P ≤ 0.05.

**Result:** **4q** significantly attenuated xylene-induced auricular inflammation and associated edematous changes (Table S23 & Figure S17). Relative to the untreated control cohort, where mean ear swelling measured 17.08 mg, animals receiving **4q** at the 0.1 mg dose exhibited a marked reduction to 3.97 mg, whereas DEX-treated mice demonstrated swelling of 6.12 mg at an equivalent dosage. Notably, at identical concentrations, compound **4q** achieved a swelling inhibition rate of 76.78%, compared to 64.20% for DEX. Furthermore, **4q** elicited dose-dependent anti-inflammatory effects, with inhibition rates escalating to 82.34% and 86.63% in the medium-dose (0.2 mg) and high-dose (0.4 mg) cohorts, respectively (Table S23). Histological examination via H&E staining corroborated these quantitative observations. Auricular tissue from normal control mice displayed well-preserved epidermal and dermal architecture, devoid of inflammatory infiltrates. In contrast, specimens from the untreated group exhibited characteristic inflammatory pathology, including pronounced epidermal hyperplasia, dermal edema, vascular dilatation, and dense leukocytic infiltration predominantly comprising neutrophils and monocytes (Figure S18). Treatment with either compound **4q** or DEX markedly ameliorated these histopathological alterations. Notably, **4q** demonstrated superior anti-inflammatory activity, preserving tissue cytoarchitecture, attenuating inflammatory cell accumulation, and reducing interstitial edema. To elucidate the molecular mechanisms underlying the anti-inflammatory actions of compound **4q**, we performed immunohistochemical analysis targeting two pivotal pro-inflammatory enzymes: inducible nitric oxide synthase (iNOS) and cyclooxygenase-2 (COX-2) (Figure S18). Basal expression of both markers was minimal in normal control tissues, consistent with a quiescent, non-inflamed state. Following xylene challenge, however, untreated specimens displayed robust upregulation of iNOS and COX-2, evidenced by intense brown-yellow immunoreactivity localized predominantly within the dermal compartment. This xylene-induced overexpression was substantially attenuated in tissues from animals treated with compound **4q**, indicating effective suppression of nitric oxide and prostaglandin-mediated inflammatory cascades.

**Table S23. Quantification of swelling degree and swelling inhibition rate (n = 6)**

| Groups | Swelling degree (mg) | Average inhibition rate (%) |
| --- | --- | --- |
| Normal | 0.12±0.32 | / |
| Untreated | 17.08±1.04 | / |
| DEX (0.1 mg) | 6.12±1.11 | 64.20 |
| **4q** (0.1 mg) | 3.97±1.30 | 76.78 |
| **4q** (0.2 mg) | 3.02±1.09 | 82.34 |
| **4q** (0.4 mg) | 2.28±1.27 | 86.63 |


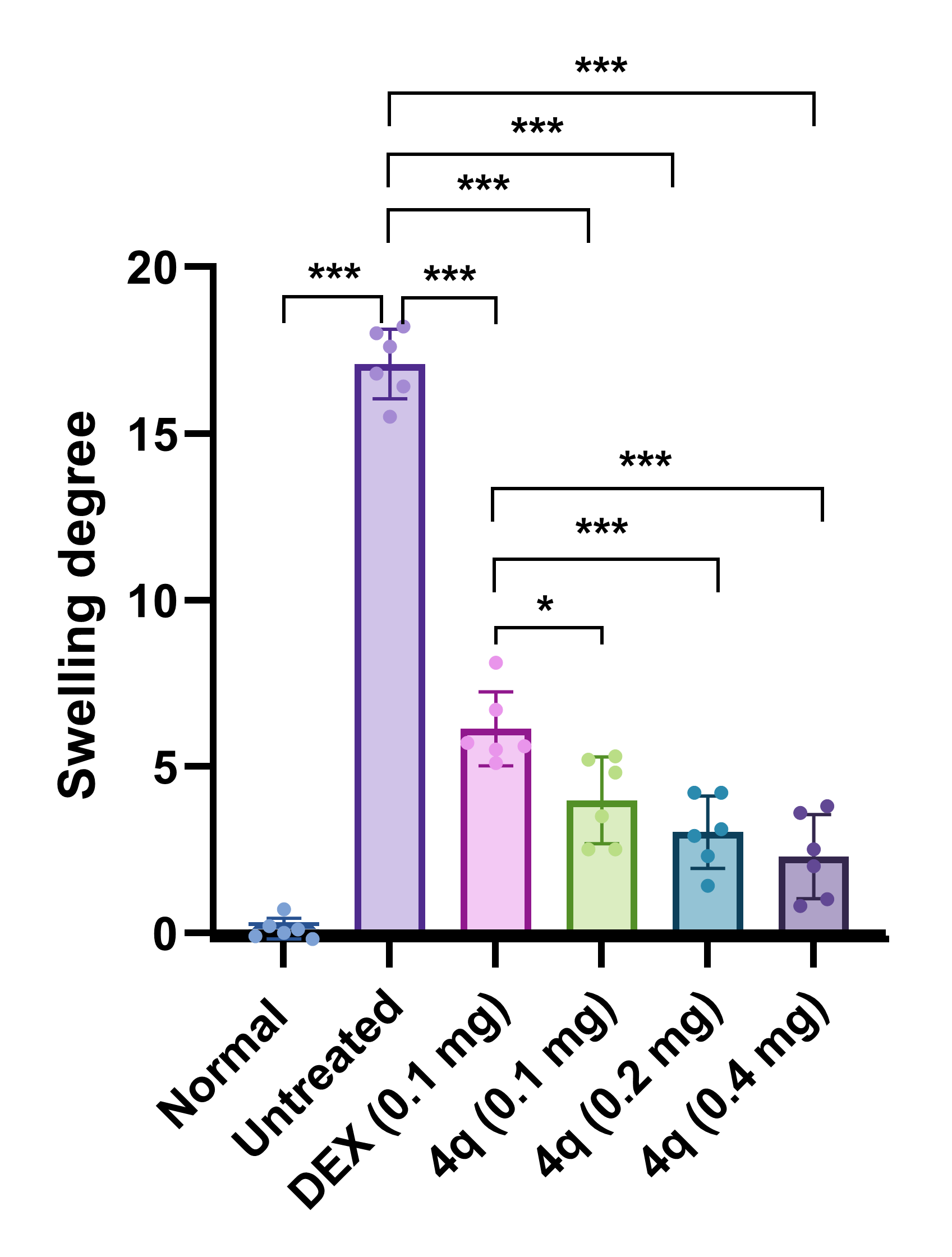


**Figure S17.** Comparative analysis of ear swelling degree. Data are presented as mean ± SD (n=6). ^*^P < 0.05, ^***^P < 0.001.


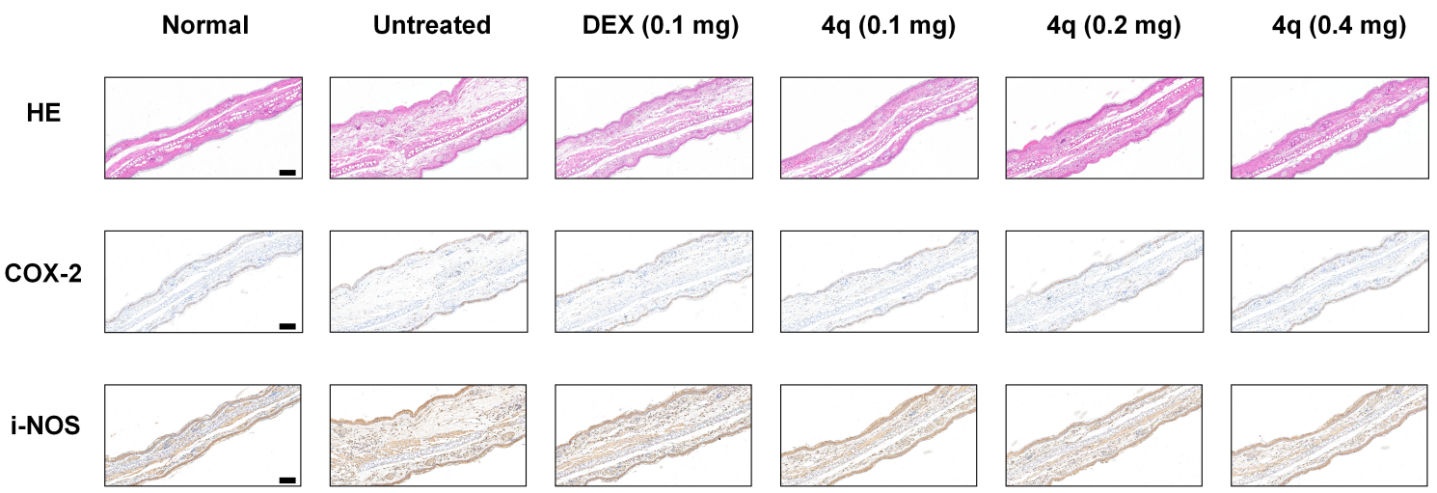


**Figure S18.** Representative H&E staining and immunohistochemical detection of iNOS and COX-2 expression in right ear specimens from respective groups. Scale bars: 100 μm. Images are representative of six independent biological replicates.

# DFT calculations

Computational details:

Geometry optimizations and frequency analyses were performed at the (U)B3LYP-D3(BJ)/def2-SVP-SDD(Ni)-SMD-(DMA) level of theory as well as the thermal corrections to free energy (G_corr_) at the temperature of 298.15 K using Gaussian 16 program[20]. Single-point energies (SP) were calculated in gas phase at the RI-(U)PWPB95-D3/def2-QZVPP-SMD-(DMA)[21,22] level of theory with def2/J[23] and def2-QZVPP/C[24] as auxiliary basis sets using ORCA 5.0.2 program[25]. The Gibbs free energies of solvation (ΔG_solv_) were calculated at the M052X/6-31G(d) level of theory using SMD model of solvation. CYLview20 was utilized to generate 3D structures.[26] Imaginary frequencies were inspected to determine the stationary points (no imaginary frequencies) or transition states (only one imaginary frequency). All transition states were confirmed by IRC calculations using the same theory level with geometry optimization. The thermal-corrected, solvated Gibbs free energies were calculated as G = SP + G_corr_ + ΔG_solv_. Zinc atom complexed with DMA molecules was used as the model of the Zn powder for simplicity[27]. ***Note***: Superscript in the top left corner of the structure name denotes spin multiplicity. For instance, **^1^A** and **^3^A** mean **A** in the singlet state and triplet state respectively. All energies in free energy diagram are in kcal/mol.

**Table S24.** Energies of intermediates and transition states

| Structure | SP (Hartree) | G_corr_ (Hartree) | ΔG_solv_ (Hartree) | Imaginary frequency  (cm^-1^) |
| --- | --- | --- | --- | --- |
| **1a** | -478.238216314 | 0.124617 | -0.018356775 | none |
| **2a** | -1024.450972866 | 0.124283 | -0.010867660 | none |
| **3a** | -707.229971341 | 0.188729 | -0.019165392 | none |
| **4a** | -1329.676767233 | 0.390535 | -0.039085440 | none |
| **DMA** | -287.76333652 | 0.097049 | -0.011099065 | none |
| **2ar** | -564.200278298 | 0.12057 | -0.009863709 | none |
| **R1** | -1042.480824742 | 0.270688 | -0.032055580 | none |
| **Zn^0^(DMA)_4_** | -2930.565663441 | 0.447123 | -0.059471410 | none |
| **ZnCl_2_(DMA)_2_** | -3275.558678445 | 0.211371 | -0.042600450 | none |
| **MgCl_2_** | -1120.580161027 | -0.024209 | -0.050151250 | none |
| **MgCl(O_2_CPh)** | -1080.55648165783 | 0.069157 | -0.036238500 | none |
| **^3^CPA** | -3880.684863829 | 0.413419 | -0.067352890 | none |
| **^2^CPB** | -3420.421921766 | 0.414288 | -0.069535350 | none |
| **^3^CPC** | -2960.183157603 | 0.413665 | -0.055998630 | none |
| **^2^CP1** | -3488.397933964 | 0.265329 | -0.035921460 | none |
| **^2^CP2** | -3171.14854170018 | 0.334973 | -0.049315750 | none |
| **^3^CP3** | -3028.12465287 | 0.263471 | -0.026520440 | none |
| **^3^CP4** | -2710.914594792 | 0.332624 | -0.066700740 | none |
| **^3^A1** | -2924.163819725 | 0.119682 | -0.051960710 | none |
| **^3^A2** | -2711.000619656 | 0.328239 | -0.047647290 | none |
| **^2^B1** | -2463.937018527 | 0.120202 | -0.026926240 | none |
| **^2^B2** | -3046.213551215 | 0.414686 | -0.049139630 | none |
| **^2^B3** | -2290.819692373 | 0.235437 | -0.023436440 | none |
| **^3^C** | -2003.640413027 | 0.119559 | -0.025642160 | none |
| **^2^D1** | -3171.184610655 | 0.32901 | -0.116647760 | none |
| **^2^D2** | -3966.687944765 | 0.418388 | -0.060328730 | none |
| **^2^D3** | -3753.537708121 | 0.630471 | -0.058486640 | none |
| **TS1a-R1** | -1042.441294363 | 0.268685 | -0.026581900 | -291.21 |
| **TS^2^B1-^3^A1** | -3488.363287788 | 0.265144 | -0.061093590 | -590.90 |
| **TS^2^B1-^2^D1** | -3171.1398691 | 0.331745 | -0.026954590 | -633.29 |
| **TS^3^C-^2^B1** | -3028.1161262 | 0.263312 | -0.030182510 | -535.48 |
| **TS^3^C-^3^A2** | -2710.912534092 | 0.330568 | -0.041335910 | -746.18 |
| **TS^2^B2-^2^D3** | -3753.446904815 | 0.632638 | 0.008612080 | -706.87 |
| **TS^3^A1-^2^D2** | -3966.676210415 | 0.414495 | -0.064695430 | -95.98 |
| **TS^3^A2-^2^B3** | -3753.524119861 | 0.632975 | -0.052168910 | -277.66 |

**Cartesian coordinates**

**1a**

C -0.80398000 0.67015900 0.09998300

O -1.07771600 1.85025600 0.28105400

C 0.61549700 0.18607200 0.03867100

C 1.61623400 1.15057200 -0.16293100

C 0.98506200 -1.15956800 0.20152900

C 2.95846700 0.77698300 -0.22291600

H 1.31993700 2.19493900 -0.27370200

C 2.33031000 -1.53155700 0.14801000

H 0.23431700 -1.92948400 0.39384700

C 3.31862900 -0.56637400 -0.06847100

H 3.72786100 1.53508600 -0.38863200

H 2.60702800 -2.57999200 0.28212300

H 4.37026100 -0.86052000 -0.11228200

N -1.77546900 -0.28671800 -0.07146400

H -1.49793800 -1.23894500 -0.29059900

C -3.13539000 -0.00819300 -0.01049000

C -4.10593000 -0.91463600 -0.19434800

H -3.34754500 1.04099100 0.20749900

H -5.15330200 -0.61368900 -0.12717600

H -3.88400200 -1.96415000 -0.41347300

**2a**

C 0.04646900 -0.89524900 0.17438600

O -0.03262800 -2.07145200 -0.14548300

C 1.47838900 -0.27556300 0.19789900

N -1.00656400 -0.11634700 0.49617300

C -0.96933000 1.29166400 0.90994600

H -1.48397800 1.36028600 1.88275900

H 0.06436700 1.59702100 1.08904900

C -1.62179700 2.23680000 -0.09221300

H -2.69496900 2.02769000 -0.21589600

H -1.52158200 3.27336300 0.26698900

H -1.13585000 2.16397500 -1.07704200

C -2.32783400 -0.76163200 0.44469500

H -3.00761400 -0.14786000 1.05227500

H -2.24416800 -1.74563900 0.92943500

C -2.87266100 -0.92861500 -0.96996100

H -3.86184100 -1.41281100 -0.93221300

H -2.98506600 0.04090000 -1.47774100

H -2.20633700 -1.56407600 -1.57163300

C 1.68112200 0.96924600 -1.09067600

F 2.37220600 -1.23910700 -0.02445700

F 1.78396200 0.27372800 1.39086300

**3a**

C -0.61130200 -0.68429700 -0.27069800

O -0.31294400 -1.80768300 -0.59748000

C -2.00902100 -0.19480200 -0.08357000

C -3.05388200 -1.11259300 -0.27662900

C -2.30413000 1.13284500 0.26843500

C -4.37946200 -0.70886400 -0.11989200

H -2.80974900 -2.14065100 -0.54963000

C -3.63239400 1.53264500 0.42376000

H -1.49487400 1.84747500 0.41999100

C -4.67020800 0.61446400 0.23064000

H -5.18923600 -1.42675500 -0.27053300

H -3.85927500 2.56590800 0.69707700

H -5.70897800 0.93114700 0.35379700

O 0.28626000 0.29506600 -0.00306200

C 2.32527900 1.16682400 -0.64855200

C 2.22459400 -0.59360400 0.99095800

C 3.80774900 0.85718200 -0.83577000

H 2.20291700 1.96040100 0.11570800

H 1.89231400 1.51950400 -1.59664600

C 3.71068000 -0.84729500 0.75446200

H 2.09158500 0.12100100 1.82739300

H 1.72229600 -1.53869100 1.24302300

H 4.35026700 1.78111200 -1.08409500

H 3.93106300 0.14366900 -1.67470900

**4a**

O 2.36759700 0.08023700 -1.97928500

C 2.42838700 -0.23686600 -0.79550200

N 1.43210700 0.03894500 0.09015100

H 1.47223900 -0.35517700 1.02265400

C 0.24943000 0.77806700 -0.29564800

H 0.10148100 0.60416400 -1.36985700

C -0.98103100 0.25259200 0.46499300

H -0.83240300 0.30286800 1.55407500

H -1.83623300 0.90090300 0.23045800

C -1.35447500 -1.18397700 0.14476400

C -2.74678600 -1.57809800 0.71035900

F -0.44511500 -2.03755100 0.70299700

F -1.27703100 -1.42096600 -1.21031800

O -2.76928600 -2.13029700 1.80342300

C 3.61538900 -0.97763800 -0.24228000

C 3.94038800 -0.99621900 1.12356300

C 4.44549000 -1.64984300 -1.15261100

C 5.06878900 -1.68870800 1.56983300

H 3.32969400 -0.45281100 1.84805100

C 5.56737100 -2.34820800 -0.70579900

H 4.19217600 -1.61441700 -2.21359300

C 5.88117800 -2.36962600 0.65789100

H 5.31690200 -1.69109700 2.63399400

H 6.20171800 -2.87615300 -1.42223000

H 6.76213100 -2.91283500 1.00915100

**DMA**

C 0.72639900 -0.29534300 0.00536200

O 1.06854500 -1.47598700 -0.00275100

N -0.59026600 0.07871600 0.02605100

C -1.62580000 -0.93615800 -0.00675000

H -1.15674900 -1.92594900 0.01363600

H -2.29947700 -0.83267500 0.86172300

H -2.23723500 -0.84322000 -0.92233000

C -1.06780000 1.44721300 -0.00447000

H -1.55702300 1.67639800 -0.96916200

H -1.81411200 1.60423800 0.79318200

H -0.25571100 2.16486400 0.14803100

C 1.76632000 0.81146700 -0.00486500

H 1.65593000 1.46636400 -0.88341200

H 1.69674500 1.44403500 0.89426400

H 2.75641300 0.33975300 -0.03193400

**2ar**

C -0.39069200 -0.74959700 -0.06916100

O -0.35185000 -1.95731900 0.16402400

C -1.68824700 -0.05408400 0.14528800

N 0.68023700 -0.00912300 -0.46755000

C 0.71204400 1.45999400 -0.51492100

H 1.65915200 1.72755200 -1.00300400

H -0.08782200 1.83056300 -1.17007100

C 0.63178600 2.13019700 0.85449400

H 1.45610400 1.80381000 1.50643700

H 0.69580500 3.22404700 0.73788200

H -0.31713500 1.90349600 1.36562800

C 1.94095900 -0.72315100 -0.68320000

H 2.43895700 -0.26048900 -1.54959300

H 1.69204700 -1.75784300 -0.95029100

C 2.86761800 -0.70914000 0.52914600

H 3.78188400 -1.28411600 0.30941100

H 3.17010100 0.31509600 0.79741600

H 2.37669100 -1.16644300 1.40251900

F -2.70829700 -0.81027800 0.50226200

F -2.09764200 0.95043700 -0.61954600

**R1**

C -2.71002200 -1.23682600 0.36294400

O -3.04955500 -2.41689900 0.29304500

C -3.67133100 -0.10764000 0.17618000

C -4.92300300 -0.41425500 -0.38597100

C -3.40293600 1.22095800 0.55324700

C -5.87620900 0.58361500 -0.58612800

H -5.12844300 -1.44948500 -0.66315100

C -4.36027700 2.21790400 0.35582200

H -2.45587400 1.49253500 1.02536700

C -5.59736700 1.90434700 -0.21692400

H -6.84207400 0.33160700 -1.03118000

H -4.14032300 3.24466100 0.65810900

H -6.34430600 2.68720400 -0.37025600

N -1.40008700 -0.88463500 0.60827000

H -1.15152700 0.10059700 0.57707500

C -0.39315000 -1.78235300 0.83488800

C 1.00756100 -1.30565200 0.95119000

H -0.66650400 -2.83466700 0.78473900

H 1.06910300 -0.37242600 1.53802300

H 1.63140600 -2.05869900 1.45105200

C 1.67031600 -1.02416600 -0.40223400

C 3.15845400 -0.62868700 -0.22557100

F 1.65424700 -2.15924000 -1.16622200

F 0.90792000 -0.11938300 -1.09671100

O 3.86481400 -1.53503700 0.20852400

N 3.62114400 0.61768100 -0.48813400

C 2.83479000 1.78291200 -0.92828600

C 5.04693200 0.84718000 -0.20957700

H 3.56555700 2.50869200 -1.31081200

H 2.20174900 1.50120100 -1.77690100

C 2.00172500 2.43607800 0.17066500

H 5.41455000 1.56834200 -0.95414700

H 5.57298900 -0.10222200 -0.36900300

C 5.31636600 1.35519700 1.20325500

H 2.63229100 2.77041800 1.00814700

H 1.47934100 3.31698200 -0.23659100

H 1.23990300 1.74871800 0.56642600

H 6.40146100 1.46601500 1.36165200

H 4.84732200 2.33521200 1.38027800

H 4.93330900 0.64542800 1.95307700

**Zn^0^(DMA)_4_**

Zn 0.22407600 0.37478600 -0.97251600

O -3.11843100 0.20353000 -2.68043200

C -3.22885200 0.64336900 -1.53718000

N -3.72462400 -0.11475200 -0.50847300

C -2.83269800 2.06277400 -1.19140700

C -4.29127300 -1.41748300 -0.80430200

C -4.01934700 0.43998000 0.80443400

H -3.71646600 2.65100000 -0.89132600

H -2.11963300 2.08832900 -0.35703600

H -2.38106500 2.52176300 -2.07909500

H -4.02092500 -2.13707700 -0.01707900

H -5.39547300 -1.37317800 -0.86255500

H -3.90488300 -1.76581300 -1.76852300

H -4.96779000 1.00983300 0.81041200

H -4.12187800 -0.38643000 1.52126300

H -3.20930400 1.08907100 1.16113900

O -1.32310400 2.23961500 1.92627100

C -0.09429200 2.29161000 1.82148000

N 0.52226100 3.20037300 1.01407100

C 0.79895300 1.35469400 2.60805100

C -0.26165100 4.19578000 0.30820800

C 1.96173300 3.29036800 0.84006900

H 1.38998200 0.70647700 1.94369300

H 1.51291000 1.91476700 3.23285900

H 0.16510300 0.73464900 3.25197400

H 0.15039200 5.20263800 0.49143100

H -0.24497400 4.00992400 -0.78022500

H -1.29797800 4.15860100 0.66050100

H 2.45881900 2.33045200 1.01923800

H 2.17747500 3.58006400 -0.19980900

H 2.40307700 4.05973800 1.50123400

O 3.57196700 0.37064000 0.78926200

C 3.36143500 -0.43803900 -0.11891500

N 3.53720900 -0.11474500 -1.43440300

C 2.90404700 -1.84810900 0.18450700

C 3.79726400 1.26677800 -1.79543200

C 3.13348000 -0.98728900 -2.52321700

H 1.91706700 -2.02701700 -0.26460500

H 3.60333700 -2.60232600 -0.20911900

H 2.81583300 -1.96377400 1.27047900

H 4.24294100 1.78952000 -0.94204700

H 4.48680600 1.30681500 -2.65281600

H 2.85790500 1.78041000 -2.07470500

H 2.05504100 -0.87122700 -2.74602000

H 3.70575500 -0.72586800 -3.42537100

H 3.33246400 -2.04041400 -2.29180100

O 0.53578600 -2.82573200 2.63886000

C -0.21965600 -2.62558500 1.68840600

N -0.15339200 -3.35955900 0.53411800

C -1.27546500 -1.54208100 1.73365800

C 0.72020300 -4.51517700 0.46360000

C -1.05551600 -3.15550200 -0.58666600

H -1.05867600 -0.75854000 0.98737600

H -2.27475200 -1.94496600 1.51394300

H -1.27386500 -1.09501900 2.73387300

H 0.13287100 -5.44837600 0.38900300

H 1.37852400 -4.45477900 -0.41859500

H 1.33364000 -4.55070300 1.37046000

H -0.58330400 -3.55029600 -1.49803000

H -2.02111600 -3.67537700 -0.45042500

H -1.24023700 -2.08662100 -0.75484200

**ZnCl_2_(DMA)_2_**

C -2.75207600 -0.34889200 0.04792900

O -1.52972700 -0.64729000 0.12143500

N -3.67933800 -1.31232400 0.01519100

C -5.11171700 -1.04016200 -0.09581700

H -5.52619100 -0.65917800 0.85141900

H -5.32260700 -0.32201000 -0.89911200

H -5.62196100 -1.97972800 -0.34177600

C -3.29493700 -2.71520400 0.12219600

H -2.21474500 -2.78985600 0.27939300

H -3.82244800 -3.18106700 0.96970600

H -3.56821200 -3.25456400 -0.79915800

C -3.15247400 1.09846000 -0.00728100

H -2.77336500 1.51859900 -0.95313400

H -4.22647300 1.28748700 0.07668200

H -2.62188200 1.61905000 0.80497200

O 1.52830500 -0.64922500 0.07931800

C 2.75156200 -0.35255400 0.01409000

N 3.67986500 -1.31529800 0.02048400

C 3.15209600 1.09262500 -0.07636800

C 5.11446800 -1.03720700 -0.04776500

C 3.29104200 -2.71998100 0.07435400

H 2.72444600 1.61480800 0.79426300

H 4.22877800 1.27699100 -0.12618800

H 2.66267600 1.51536600 -0.96853200

H 5.39315900 -0.58607100 -1.01251300

H 5.43475100 -0.37367700 0.76820500

H 5.65358300 -1.98652900 0.05527100

H 2.19992300 -2.79826300 0.09369300

H 3.68052800 -3.25019200 -0.80942100

H 3.70797400 -3.19248800 0.97813500

Zn -0.00049800 0.65750600 0.01820000

Cl -0.02949100 1.63551200 -2.02643200

Cl 0.03225100 1.93620100 1.88809700

**MgCl_2_**

Cl 0.00000000 2.24774100 0.05561900

Cl 0.00000000 -2.24774100 0.05561900

Mg 0.00000000 0.00000000 -0.15758700

**MgCl(O_2_Ph)**

Cl 4.66279000 0.00104600 -0.13264700

Mg 2.41582000 0.00023800 0.10671700

O 0.68025300 1.09443600 0.04872100

C 0.03115100 -0.00207200 0.09205100

O 0.67906500 -1.09704600 0.17265100

C -1.45223200 -0.00183500 0.03662600

C -2.15371000 1.21448100 0.02668200

C -2.15475500 -1.21620000 -0.01889300

C -3.54718500 1.21455200 -0.03710100

H -1.59513500 2.15064300 0.06972000

C -3.54807400 -1.21196900 -0.08910400

H -1.59739200 -2.15410200 -0.01206500

C -4.24441400 0.00228200 -0.09724500

H -4.09313900 2.16094200 -0.04192100

H -4.09479700 -2.15662300 -0.13864200

H -5.33604200 0.00395500 -0.15177700

**^3^CPA**

C -4.24828600 -2.12000600 -1.64275300

C -4.14160700 -3.49427300 -1.42286200

C -2.97426300 -4.00625600 -0.85697700

C -1.93713000 -3.12513700 -0.52579800

C -3.17264600 -1.30595700 -1.28816300

C -0.65807500 -3.55789300 0.09223600

C -0.36530600 -4.89295500 0.39733300

C 0.86200800 -5.20265900 0.98186600

H 1.10609100 -6.23853600 1.22685800

C 1.76759100 -4.17487100 1.24711900

C 1.40475700 -2.87025400 0.91462000

H -5.14611700 -1.68016500 -2.08000500

H -4.96113400 -4.16593600 -1.68743200

H -3.19215200 -0.22293600 -1.42692200

H 2.73890300 -4.37173500 1.70356900

H 2.08069500 -2.03509300 1.10280300

N -2.05774100 -1.80354300 -0.75207100

N 0.22907600 -2.57764100 0.35668300

Ni -0.42274100 -0.68436300 -0.18454500

Cl 0.53704400 -1.00600500 -2.39072000

H -2.87683500 -5.07642700 -0.67732700

H -1.08495100 -5.68218700 0.18467100

O -1.33833900 1.14441000 -0.77684700

C -1.22793300 2.37198800 -0.64363000

C -2.33806600 3.17064500 -0.04575500

N -0.13043400 3.06214900 -1.03826000

C -3.15721900 2.54375100 0.90803800

C -2.60209400 4.49650200 -0.42913900

H -0.09160300 4.05378500 -0.81432200

C 0.94625600 2.52216700 -1.74431500

C -4.21656900 3.24586900 1.48396900

H -2.92238300 1.51979000 1.21066000

C -3.67211500 5.18860000 0.14146500

H -1.99297400 4.98571500 -1.19316100

C 2.01470500 3.24771200 -2.09965500

H 0.83920900 1.46022400 -1.98357300

C -4.47813000 4.56619300 1.10046100

H -4.84265300 2.76152700 2.23759200

H -3.88087000 6.21523100 -0.16871000

H 2.82852100 2.78077600 -2.65650400

H 2.10810400 4.30766500 -1.84129200

H -5.31318700 5.11105200 1.54809500

Cl -1.46770800 -0.44245500 2.00451500

O 1.33113300 0.37763500 0.46153200

C 2.45153200 0.58780000 0.94068000

C 3.64299400 0.81469400 0.07153500

N 2.67114800 0.61827400 2.28120600

C 3.64569500 0.23706500 -1.20792800

C 4.72774700 1.60840800 0.48052400

H 3.62894100 0.73342200 2.60388300

C 1.67760600 0.45067400 3.24579500

C 4.73637300 0.43040200 -2.05606300

H 2.77853200 -0.34314400 -1.53484500

C 5.80817700 1.81261400 -0.37986300

H 4.72305800 2.09676000 1.45793200

**^2^CPB**

C 2.76933600 -3.16346400 -2.41499000

C 4.10919100 -2.71345700 -2.46355600

C 4.49310500 -1.65691100 -1.65550800

C 3.54793100 -1.04514100 -0.80318100

C 1.89220400 -2.53305800 -1.55081800

C 3.82605900 0.08412600 0.06439400

C 5.08098100 0.72289000 0.17550500

C 5.22794600 1.80838500 1.02210300

H 6.19377500 2.31018600 1.11382500

C 4.11103800 2.25443300 1.76638500

C 2.91001400 1.58292000 1.62241900

H 2.42405500 -3.99024300 -3.03827900

H 4.83248800 -3.19079200 -3.12841600

H 0.84783400 -2.84432700 -1.47030100

H 4.18283500 3.10734200 2.44368300

H 2.01783400 1.88072200 2.17766500

N 2.25402600 -1.50072100 -0.76093400

N 2.75298400 0.52784100 0.79609000

Ni 1.12902500 -0.64417000 0.66843500

H 5.52140100 -1.29499100 -1.67746400

H 5.93008100 0.36159100 -0.40567600

O -0.70146200 -1.34598500 -0.29340000

C -1.63570100 -0.87387200 -0.95867200

C -3.02571300 -1.38033600 -0.76677600

N -1.46062300 0.10759300 -1.87883200

C -3.18516100 -2.64632200 -0.18221300

C -4.16259800 -0.61983700 -1.08639400

H -2.27743200 0.43202900 -2.38997700

C -0.23796000 0.69534300 -2.20603400

C -4.46103600 -3.15446900 0.05954000

H -2.29652800 -3.22106500 0.08157800

C -5.43821000 -1.12294800 -0.82629500

H -4.06549600 0.38022300 -1.51340600

C -0.11605400 1.70596800 -3.07484000

H 0.62164700 0.26478900 -1.68850200

C -5.59006400 -2.39217800 -0.25794600

H -4.57599000 -4.14460600 0.50664500

H -6.31672900 -0.51925000 -1.06531500

H 0.87011000 2.12235600 -3.28748800

H -0.98244900 2.14177000 -3.58150000

H -6.59009900 -2.78515900 -0.05876200

O -0.09512000 0.98850200 1.43091600

C -1.24475500 1.39683200 1.20395000

C -1.46531200 2.60240600 0.35369500

N -2.33480400 0.76370300 1.70177900

C -0.36790900 3.45142400 0.13734700

C -2.68368700 2.87415700 -0.29134700

H -3.25235600 1.17205100 1.54397500

C -2.27505000 -0.40401700 2.46505300

C -0.48977200 4.56359400 -0.69463300

H 0.58111000 3.22263200 0.62338800

C -2.79784600 3.97839200 -1.13685200

H -3.54447600 2.21435900 -0.16871400

C -3.36488900 -1.01469600 2.94626100

H -1.26302600 -0.79219900 2.61111200

C -1.70369200 4.82705200 -1.33734700

H 0.36818800 5.22148100 -0.85146000

H -3.74456600 4.17373800 -1.64577200

H -3.26127300 -1.93422500 3.52538000

H -4.37370200 -0.63047500 2.76555900

H -1.79679600 5.69131400 -1.99957100

Cl 1.05259000 -2.04146100 2.53525200

**^3^CPC**

C -2.94200600 -3.59638400 1.52796900

C -4.30933800 -3.23047100 1.37982900

C -4.63369100 -2.10795400 0.64798700

C -3.60954500 -1.32036800 0.04537100

C -1.98832800 -2.79416100 0.91882700

C -3.83489200 -0.13982500 -0.73260300

C -5.11596000 0.41880000 -1.01484500

C -5.22542800 1.55920900 -1.78192000

H -6.20904600 1.98551000 -1.99583700

C -4.04707200 2.17324500 -2.29143200

C -2.82745500 1.58870200 -1.98227700

H -2.64191800 -4.47720000 2.09815000

H -5.09677800 -3.83235700 1.84100400

H -0.92196600 -3.02796900 1.00236900

H -4.08832800 3.07533400 -2.90422300

H -1.88884800 2.02159400 -2.34261900

N -2.28039800 -1.69622000 0.20210800

N -2.69598900 0.48154200 -1.23270600

Ni -1.01977500 -0.41653700 -0.66396800

H -5.67863300 -1.81768700 0.52706200

H -6.01246500 -0.06111200 -0.61876000

O 0.75029700 -1.39400700 -0.13611100

C 1.69516300 -1.17366000 0.63849800

C 3.09618800 -1.45797500 0.21516800

N 1.51758600 -0.66270800 1.88266900

C 3.30208500 -2.41308400 -0.79271900

C 4.19432300 -0.75076300 0.73197900

H 2.33579000 -0.56533400 2.47814900

C 0.29057900 -0.26780700 2.41633200

C 4.58959300 -2.67695900 -1.25867600

H 2.44246200 -2.94520500 -1.20283400

C 5.47925200 -1.00292000 0.24880400

H 4.05198200 0.02373400 1.48845700

C 0.14417100 0.20217000 3.66090500

H -0.54825100 -0.35482000 1.71941000

C 5.68015000 -1.96994600 -0.74179400

H 4.74262300 -3.42955700 -2.03565900

H 6.32633300 -0.43739000 0.64382000

H -0.84205900 0.51148800 4.01128800

H 0.99051600 0.29799700 4.34806800

H 6.68771300 -2.16691200 -1.11609400

O 0.30388100 1.00545700 -1.54029500

C 1.22874900 1.57053100 -0.93245200

C 0.99508100 2.33111700 0.32866800

N 2.50695700 1.50798400 -1.38399800

C -0.30825400 2.78041200 0.59792900

C 2.01364700 2.56834000 1.26723900

H 3.21819200 2.05297800 -0.90389000

C 2.91755500 0.73454700 -2.46881000

C -0.58445200 3.47354500 1.77607600

H -1.09802200 2.58774900 -0.12725400

C 1.73101900 3.25143100 2.45061700

H 3.02550800 2.19258800 1.10214500

C 4.18587800 0.67323700 -2.89256700

H 2.10976600 0.16684100 -2.93307100

C 0.43375000 3.70896200 2.70539800

H -1.59971100 3.82601900 1.97230500

H 2.52553100 3.41863400 3.18139800

H 4.44837000 0.03356000 -3.73689400

H 4.98441300 1.24394900 -2.40911800

H 0.21544600 4.24299300 3.63345700

**^2^CP1**

C 0.42221800 -0.53283200 -3.69309800

C 0.93979200 0.76326600 -3.75323700

C 1.60728900 1.27964200 -2.64329800

C 1.73403100 0.48773500 -1.49747800

C 0.59765200 -1.26680000 -2.52198900

C 2.36372100 0.94457200 -0.23837300

C 2.94415100 2.20495100 -0.06374200

C 3.47759800 2.53943700 1.18066100

H 3.93280600 3.51958400 1.33704200

C 3.41734500 1.60719400 2.21843300

C 2.82723600 0.36970300 1.96955000

H -0.10975300 -0.97406700 -4.53742200

H 0.82199100 1.36945300 -4.65374200

H 0.21251300 -2.28484600 -2.42831400

H 3.81822300 1.82931000 3.20863700

H 2.75634200 -0.39193000 2.75004900

N 1.23403100 -0.76909100 -1.45214900

N 2.31424700 0.04615000 0.77359100

Ni 1.47676800 -1.68761400 0.29441000

Cl -1.84096000 -1.70044100 0.17671200

C -3.21215800 -0.52798700 0.14930700

C -2.86535000 0.68280600 1.06424700

F -4.29799000 -1.14133400 0.62458600

F -3.47783100 -0.19478600 -1.12814600

O -3.36319400 0.68122600 2.17983600

Cl 1.13678000 -3.66103500 1.18918400

H 2.97856700 2.91780900 -0.88707800

H 2.00882000 2.29227800 -2.66455200

N -2.01082000 1.61561800 0.59615000

C -1.37952200 1.64606500 -0.72863900

H -1.26685200 0.62862900 -1.11645500

H -0.36045600 2.02467200 -0.57557600

C -2.11688600 2.52706600 -1.72900800

H -2.19769400 3.56364100 -1.36516900

H -1.56845000 2.54358900 -2.68412000

H -3.13122200 2.14595100 -1.91841900

C -1.61281100 2.68186400 1.52416300

H -2.44470200 2.85819900 2.21741200

H -1.46565000 3.59883600 0.93469800

C -0.34206900 2.32312300 2.28637600

H -0.05479800 3.14459300 2.96167800

H -0.48908100 1.41433900 2.89051500

H 0.49325800 2.14591300 1.59404100

**^2^CP2**

C 2.66291100 1.22949900 -0.29682200

C 3.95051700 1.59221900 -0.71165500

C 5.04140700 0.85292600 -0.25749200

C 4.82550100 -0.22180200 0.60736800

C 3.51512900 -0.52540700 0.97108700

H 4.10194900 2.44415000 -1.37337000

H 6.05257400 1.12063900 -0.57122300

H 5.65220000 -0.82019600 0.99310100

H 3.29293700 -1.35904600 1.64090100

C 1.44008100 1.96968700 -0.69569200

C 1.44655200 3.05452100 -1.58051600

C 0.24427800 3.69551900 -1.87629100

H 2.37601700 3.39580600 -2.03416300

C -0.86603300 2.15778300 -0.41282000

C -0.93549500 3.24599300 -1.28078200

H 0.23204500 4.54221000 -2.56595100

H -1.75954900 1.76410800 0.07605800

H -1.89549000 3.72483900 -1.47898600

N 2.46935600 0.17123200 0.51689100

N 0.28743000 1.54123200 -0.13807400

Ni 0.46813200 -0.12995300 1.05576600

Cl 0.86877700 0.55242600 3.24816900

C -0.03660100 -2.96826100 -0.22196500

C 0.47790100 -1.39330900 -1.99210900

C 1.40775900 -3.44066800 -0.29021100

H -0.66472100 -3.58175700 -0.89070500

H -0.42189800 -3.04442400 0.80391300

C 1.88932000 -1.97011600 -2.03283500

H -0.15610000 -1.92182100 -2.72459600

H 0.49308400 -0.32437300 -2.24395400

H 1.45169700 -4.50370400 -0.01278700

H 2.02685800 -2.87441800 0.43027700

H 2.24484500 -1.94570100 -3.07316800

H 2.57556800 -1.35714500 -1.42755100

N -0.11880300 -1.55734900 -0.65423200

O 1.92284100 -3.31736500 -1.60159900

O -1.50279500 -1.24988400 -0.84698700

C -2.12462900 -0.76438900 0.30140100

O -1.44231600 -0.65594500 1.36738700

C -3.48593500 -0.39514200 0.12355400

C -4.20802100 0.18951900 1.21175900

C -4.17991700 -0.57617900 -1.11488600

C -5.53335700 0.57025200 1.05975700

H -3.69341500 0.33588900 2.16328100

C -5.50773800 -0.18957600 -1.24272400

H -3.65629400 -1.02270700 -1.96135900

C -6.20506600 0.38775200 -0.16517400

H -6.06197600 1.01920300 1.90604600

H -6.01687400 -0.33767200 -2.19989000

H -7.24946700 0.68837100 -0.27639900

**^3^CP3**

C 2.64205300 -0.75982100 -0.07212700

C 3.70127900 -1.58623800 0.39841700

C 3.56349400 -2.95791700 0.40548400

C 2.35285400 -3.54101200 -0.06106400

C 1.35284200 -2.69178600 -0.50301300

H 4.62575500 -1.12721900 0.75132100

H 4.37897300 -3.59015700 0.76539100

H 2.20339900 -4.62166500 -0.07324300

H 0.40064800 -3.09551400 -0.86194000

C 2.68971400 0.67169600 -0.13734900

C 3.78683400 1.46997000 0.29166300

C 3.73154200 2.84375400 0.19077300

H 4.67324500 0.98736500 0.70568700

C 1.52874100 2.63994200 -0.75509700

C 2.56783400 3.45896400 -0.34928800

H 4.57514000 3.45335700 0.52380600

H 0.61198900 3.06522000 -1.17462500

H 2.48462300 4.54235700 -0.44700500

N 1.46392100 -1.35030300 -0.51388700

N 1.56256800 1.29545700 -0.66310000

Ni 0.16939900 0.03061700 -1.06153800

Cl -1.83114300 -1.03958400 -1.51724100

C -3.13894700 0.11130100 -0.82293200

C -2.57964100 0.89732100 0.40616500

F -3.43433600 0.95460300 -1.79743800

F -4.21858600 -0.62708300 -0.57095400

O -2.43100100 2.09987400 0.24324000

N -2.29794300 0.22718200 1.53644700

C -2.50299300 -1.20529900 1.79143000

C -1.76639000 1.03176900 2.64979100

H -3.21527500 -1.29118400 2.62906400

H -2.98687100 -1.66975500 0.92889300

C -1.21356400 -1.94825000 2.11740200

H -1.94652800 0.45924900 3.57001400

H -2.35928800 1.95581300 2.71125800

C -0.28731400 1.37275400 2.50283400

H -0.76394100 -1.60004600 3.05903700

H -1.43410400 -3.02136200 2.22893400

H -0.47252300 -1.82750400 1.31489000

H 0.04134100 1.96161000 3.37413000

H 0.33383200 0.46748700 2.44600800

H -0.10968300 1.97169800 1.59938900

**^3^CP4**

C 3.23231300 -1.44313600 0.07537200

C 4.18871100 -2.45509900 0.23154100

C 3.76104700 -3.75934900 0.47372500

C 2.38991100 -4.02343500 0.55040100

C 1.49830400 -2.96610800 0.38491000

H 5.25277300 -2.22968800 0.16545600

H 4.49143700 -4.56136900 0.60035900

H 2.01389600 -5.03123200 0.73517100

H 0.41579000 -3.10520300 0.43193600

C 3.55926700 -0.02532500 -0.19020900

C 4.87068200 0.44140100 -0.34313300

C 5.09128400 1.78993700 -0.61334800

H 5.71227900 -0.24504100 -0.25480800

C 2.71452900 2.10581000 -0.55928000

C 3.98908400 2.64022900 -0.72858600

H 6.10802000 2.16906300 -0.73551900

H 1.83618500 2.74678900 -0.64561200

H 4.10670400 3.70316900 -0.94605200

N 1.91034800 -1.71117700 0.15559700

N 2.49184600 0.80910200 -0.28961500

Ni 0.69771000 -0.11438700 -0.02109100

C -1.09634200 2.14231700 -0.99330200

C -0.44271800 2.34898800 1.34212400

C -2.11927200 3.24528400 -0.75602200

H -0.15605400 2.57237400 -1.36770500

H -1.45847800 1.41419800 -1.73353700

C -1.49401500 3.44244300 1.49111200

H 0.52692800 2.80110800 1.09166500

H -0.32749900 1.77123900 2.26931500

H -2.24053000 3.83820900 -1.67503600

H -3.10205900 2.80566300 -0.50098100

H -1.15241200 4.18118300 2.23173400

H -2.44793500 3.01240700 1.85076600

O -1.96612300 0.72742400 0.67772200

C -2.06595700 -0.55000700 0.13075600

O -0.99439500 -1.19673700 -0.09546900

C -3.39054500 -1.01027200 -0.06917700

C -3.62086900 -2.35916100 -0.50709500

C -4.54702700 -0.18992800 0.16350200

C -4.90799400 -2.83552800 -0.70204400

H -2.75826600 -3.00398800 -0.68678500

C -5.82469700 -0.68918000 -0.04066500

H -4.40928000 0.83785600 0.50299100

C -6.03325300 -2.01524700 -0.47486600

H -5.05082400 -3.86744100 -1.03849400

H -6.68606900 -0.03848700 0.14074000

H -7.04423200 -2.39902200 -0.63047300

N -0.75518200 1.40773600 0.24858600

O -1.69072900 4.12976200 0.26639300

**^3^A1**

C -0.74118000 1.43994000 0.02031300

C -1.52469300 2.59765300 0.00570500

C -2.91364700 2.47362600 0.04597400

C -3.48829400 1.20288200 0.10073700

C -2.64572600 0.09263200 0.10820900

H -1.06077300 3.58207800 -0.04323000

H -3.54166000 3.36682000 0.03285100

H -4.57000500 1.06566300 0.13332200

H -3.03940100 -0.92614200 0.14458500

C 0.74087100 1.44007800 -0.02027700

C 1.52413600 2.59796200 -0.00563000

C 2.91311700 2.47424300 -0.04578600

H 1.05997000 3.58226700 0.04326800

C 2.64572100 0.09318300 -0.10799100

C 3.48804200 1.20362100 -0.10047100

H 3.54093100 3.36757700 -0.03262200

H 3.03963300 -0.92550200 -0.14432200

H 4.56978700 1.06664400 -0.13296300

N -1.31605300 0.21855700 0.06805900

N 1.31601200 0.21880400 -0.06796700

Ni 0.00017200 -1.30833400 -0.00002500

Cl 0.18496700 -2.18563900 2.07236300

Cl -0.18456000 -2.18509600 -2.07268800

**^3^A2**

C -2.31041000 1.28667400 1.11173400

C -2.93892100 1.76456900 2.26597700

C -2.26199900 1.69139700 3.48380100

C -0.97878000 1.14530900 3.51888500

C -0.41360400 0.68592700 2.32881400

H -3.94397600 2.18222900 2.21963600

H -2.73782200 2.05789400 4.39593800

H -0.41721800 1.07010500 4.45149800

H 0.58571400 0.24124000 2.27894000

C -2.93801100 1.30844400 -0.23276100

C -4.19150300 1.87066600 -0.49822300

C -4.68409100 1.83716600 -1.80273600

H -4.77487300 2.33208300 0.29766800

C -2.68077800 0.70316100 -2.46734000

C -3.91875800 1.24505300 -2.80908300

H -5.65963500 2.27248600 -2.02969600

H -2.04356500 0.21969500 -3.21231900

H -4.26908900 1.19965000 -3.84127100

N -1.06790000 0.75825300 1.16271800

N -2.21158700 0.73715200 -1.21565500

Ni -0.39400200 -0.02919100 -0.61120200

C 0.48314500 -2.77506300 -0.65188300

C -1.84647000 -2.52346900 -0.97119600

C 0.22712600 -3.31434500 0.75552500

H 0.52612700 -3.64475800 -1.34495200

H 1.46548700 -2.28168000 -0.67966000

C -2.08903800 -3.06582600 0.43961000

H -1.89011800 -3.38554900 -1.67355300

H -2.66750700 -1.84128400 -1.24260100

H 0.97348100 -4.07635400 1.03538200

H 0.30133100 -2.47566500 1.47566300

H -3.02753700 -3.64279100 0.48995800

H -2.17019000 -2.21114100 1.14471300

O 2.25586900 -0.53678200 1.29613200

C 2.44485700 0.06597000 0.23040900

O 1.54062300 0.37779800 -0.62684800

C 3.85337800 0.49048900 -0.13482500

C 4.12838100 1.14742700 -1.34390000

C 4.90741100 0.22060300 0.75119600

C 5.43478500 1.52850100 -1.66075300

H 3.30564800 1.35354400 -2.03014800

C 6.21360100 0.60113600 0.43686300

H 4.67969200 -0.29198900 1.68746400

C 6.48003200 1.25625500 -0.77103900

H 5.64033100 2.03910400 -2.60533100

H 7.02808700 0.38694800 1.13393200

H 7.50224600 1.55417600 -1.01890900

N -0.56199300 -1.85158300 -1.06310300

O -1.04727800 -3.93696800 0.84560800

**^2^B1**

C -1.31967800 -0.74014200 0.00099400

C -2.47724100 -1.52461400 0.01651500

C -2.35477000 -2.91403500 0.01355500

C -1.08010500 -3.48412600 -0.00371500

C 0.02784400 -2.63903800 -0.01425400

H -3.46238600 -1.05923200 0.03338200

H -3.24709200 -3.54339900 0.02499000

H -0.93884900 -4.56612100 -0.00745600

H 1.04525100 -3.03798700 -0.02517700

C -1.31961000 0.74025500 -0.00099500

C -2.47710300 1.52482500 -0.01654700

C -2.35451900 2.91423600 -0.01358100

H -3.46228100 1.05951900 -0.03344300

C 0.02807100 2.63903600 0.01429300

C -1.07980700 3.48421800 0.00372600

H -3.24679000 3.54367200 -0.02503900

H 1.04551000 3.03789900 0.02524600

H -0.93846100 4.56620100 0.00747400

N -0.08815500 -1.30341900 -0.01121200

N -0.08804100 1.30342600 0.01124400

Ni 1.41438300 -0.00007200 0.00001100

Cl 3.60454400 -0.00013400 -0.00002700

**^2^B2**

C 4.45630300 -0.45863200 -1.91168700

C 5.41531400 0.49477900 -1.56623600

C 5.02947600 1.60402100 -0.81326700

C 3.69129300 1.72564500 -0.42295900

C 3.13981000 -0.26961300 -1.48876800

C 3.16259400 2.85716800 0.37378000

C 3.94129900 3.93054500 0.81882300

C 3.33940900 4.94811700 1.55845800

H 3.93224100 5.79383400 1.91310900

C 1.97296000 4.86747900 1.83592800

C 1.26088700 3.76772000 1.36262300

H 4.71599700 -1.34028100 -2.50039700

H 6.45533900 0.38022900 -1.87946300

H 2.35210800 -0.98982700 -1.73437700

H 1.46073400 5.64105000 2.41035200

H 0.19086200 3.66044700 1.55738700

N 2.77169200 0.79363600 -0.76134100

N 1.83744200 2.78658900 0.65043100

Ni 0.89701200 1.18148400 -0.02659800

C -0.47882200 -0.22724400 -0.36283700

C -1.86288900 0.15352800 0.17424800

H -1.86541700 0.26655600 1.27220300

H -2.13472600 1.13791300 -0.22941500

C -2.99909700 -0.81866700 -0.13379600

C -4.41052800 -0.28677200 0.23869900

F -2.81859100 -1.97659800 0.56998700

F -2.97684700 -1.20879700 -1.45992400

O -4.95923300 -0.75987300 1.22721700

C -4.40639700 1.31968200 -1.71384800

H -3.51141600 0.77394200 -2.02023700

H -5.14297300 1.18158700 -2.52438600

C -4.08515400 2.80307600 -1.56228100

H -3.36278400 2.97445600 -0.74838400

H -3.64328200 3.17946400 -2.49875200

H -4.98507100 3.40132100 -1.35421200

H 5.76172100 2.36177900 -0.53539700

H 5.00561900 3.97339400 0.58974300

N -4.96071400 0.71110400 -0.50038800

C -6.28019500 1.18930700 -0.06711400

H -6.74499100 1.68704100 -0.93054100

H -6.89611100 0.31246100 0.18245900

C -6.22862100 2.13249200 1.13104800

H -7.24792200 2.45428800 1.40035700

H -5.79077500 1.62443800 2.00277100

H -5.63248700 3.03165900 0.91396000

N -0.04385300 -1.51078000 0.19148700

H -0.17444000 -1.64776200 1.18731000

C 0.54341900 -2.48990500 -0.51131900

O 0.72609100 -2.42452700 -1.74036100

C 1.00218000 -3.69538000 0.26588200

C 1.29591100 -4.86143800 -0.45939000

C 1.17516000 -3.70595700 1.66089000

C 1.72756600 -6.01782600 0.19162700

H 1.17755000 -4.83661300 -1.54391300

C 1.61269700 -4.86151500 2.31279700

H 0.98770800 -2.80723400 2.25335900

C 1.88628500 -6.02232300 1.58183500

H 1.94429700 -6.91974800 -0.38681100

H 1.74733100 -4.85222700 3.39738500

H 2.22786900 -6.92550600 2.09388900

H -0.55637400 -0.38801300 -1.44878300

**^2^B3**

C 3.01353200 -0.74113200 -0.00264200

C 4.17283500 -1.52365300 -0.02204300

C 4.05380400 -2.91360000 -0.02265600

C 2.78023700 -3.48695500 -0.00501200

C 1.67050900 -2.64399300 0.00967600

H 5.15737700 -1.05688700 -0.03940300

H 4.94772100 -3.54068700 -0.03725300

H 2.64224000 -4.56949400 -0.00405900

H 0.65281900 -3.04197800 0.02116500

C 3.01352500 0.74113700 0.00282800

C 4.17281700 1.52366500 0.02259400

C 4.05377800 2.91361200 0.02312700

H 5.15735600 1.05690500 0.04028000

C 1.67049500 2.64399000 -0.00997800

C 2.78021300 3.48695800 0.00503900

H 4.94768600 3.54070500 0.03799200

H 0.65280700 3.04196900 -0.02180400

H 2.64221000 4.56949600 0.00399900

N 1.78356900 -1.30845400 0.01015800

N 1.78356200 1.30845100 -0.01037600

Ni 0.27889600 -0.00000600 -0.00023900

O -1.53614000 1.09962600 -0.01756400

C -2.16506800 0.00001200 -0.00016100

O -1.53613300 -1.09959700 0.01714700

C -3.66416400 0.00000200 -0.00002000

C -4.37229500 1.21190200 -0.02007400

C -4.37227400 -1.21190800 0.02019000

C -5.76845700 1.21173900 -0.01993800

H -3.81247500 2.14840900 -0.03569400

C -5.76843600 -1.21176400 0.02037800

H -3.81243600 -2.14840600 0.03569000

C -6.46863100 -0.00001700 0.00030000

H -6.31445100 2.15846600 -0.03568800

H -6.31441200 -2.15849900 0.03625300

H -7.56177300 -0.00002500 0.00042700

**^3^C**

C -0.00000100 -0.72815200 0.71736100

C -0.00000100 -1.90024600 1.52625500

C 0.00000100 -1.80095700 2.90119400

C 0.00000200 -0.51549800 3.51028500

C 0.00000100 0.59438900 2.68272300

H -0.00000100 -2.88207300 1.05109000

H 0.00000100 -2.70418800 3.51638800

H 0.00000300 -0.39390700 4.59449600

H 0.00000100 1.60510900 3.10309800

C -0.00000100 -0.72815200 -0.71736100

C -0.00000100 -1.90024600 -1.52625500

C 0.00000100 -1.80095700 -2.90119400

H -0.00000100 -2.88207300 -1.05109000

C 0.00000100 0.59438900 -2.68272300

C 0.00000200 -0.51549800 -3.51028500

H 0.00000100 -2.70418800 -3.51638800

H 0.00000100 1.60510900 -3.10309800

H 0.00000300 -0.39390700 -4.59449600

N 0.00000000 0.51936600 1.33746500

N 0.00000000 0.51936600 -1.33746500

Ni -0.00000100 1.91730600 0.00000000

**^2^D1**

C -3.05145400 -0.01018000 -0.52312300

C -4.43500500 -0.21517600 -0.45615200

C -4.92516600 -1.51006300 -0.29700100

C -4.02584300 -2.57426500 -0.21506500

C -2.66286000 -2.29564900 -0.28761700

H -5.12293700 0.62589000 -0.53086400

H -6.00168400 -1.68522000 -0.24271300

H -4.36600200 -3.60402800 -0.09675200

H -1.91697600 -3.09172600 -0.23165900

C -2.43271700 1.32725400 -0.70736200

C -3.16330100 2.52132500 -0.75694800

C -2.47530900 3.72279400 -0.92951600

H -4.24831100 2.52078600 -0.65907300

C -0.43112600 2.47318600 -0.99535300

C -1.08497300 3.70507900 -1.05404400

H -3.02497000 4.66576500 -0.96665200

H 0.65533600 2.39083700 -1.08370000

H -0.51352600 4.62430000 -1.19320200

N -2.19691400 -1.05075500 -0.42747100

N -1.09301200 1.32929100 -0.82334600

Ni -0.22427500 -0.62580500 -0.54737600

Cl -0.22607500 -1.45087000 -2.71286000

C -0.81427800 -1.27070600 2.38208200

C 0.49190800 0.68629300 2.03429200

C -1.90224200 -0.40345800 3.05741900

H -0.11662800 -1.60999800 3.17007400

H -1.26687600 -2.15146400 1.91022600

C -0.61665100 1.51996600 2.71602400

H 1.20826300 0.37047000 2.81538900

H 1.02462400 1.28227400 1.28417800

H -2.39383900 -0.98445000 3.85106600

H -2.66180100 -0.12009800 2.30516400

H -0.16007100 2.35971800 3.25947100

H -1.29586000 1.92763300 1.94455500

N -0.09541100 -0.47697500 1.42433700

O -1.32716900 0.73784900 3.64925200

O 2.25590400 -2.00896200 0.20384200

C 2.50295100 -0.86515300 -0.19747800

O 1.63457200 -0.03523800 -0.65543400

C 3.92531400 -0.34644800 -0.15210700

C 4.23892600 0.96336800 -0.54601000

C 4.95106400 -1.18700700 0.30545700

C 5.55636600 1.42459300 -0.48170500

H 3.43778900 1.61276000 -0.90184800

C 6.26852500 -0.72862700 0.36890500

H 4.69197700 -2.20263400 0.60985700

C 6.57380000 0.57959600 -0.02429600

H 5.79210600 2.44687800 -0.78913300

H 7.06119800 -1.39122500 0.72643900

H 7.60450500 0.94042000 0.02598300

**^2^D2**

C -4.20652200 -0.16612400 -1.76140400

C -4.96068800 -1.27722600 -1.38630900

C -4.33601700 -2.32302900 -0.70821100

C -2.97403900 -2.22434100 -0.40388100

C -2.85540400 -0.13371600 -1.42066200

C -2.20828400 -3.30541800 0.26175200

C -2.76519100 -4.52223200 0.67380600

C -1.93646100 -5.47796600 1.26069200

H -2.35194700 -6.43307200 1.58883900

C -0.57696900 -5.20242300 1.41962600

C -0.09733300 -3.96634100 0.98879200

H -4.64599100 0.66773300 -2.31059400

H -6.02368700 -1.33901600 -1.62842700

H -2.23025900 0.70654800 -1.71882000

H 0.10365700 -5.92661900 1.86982400

H 0.95612800 -3.69390400 1.09199400

N -2.26381700 -1.12332800 -0.74361200

N -0.89716800 -3.05533900 0.43235900

Ni -0.32452700 -1.14961300 -0.09048300

C 0.41379400 0.84800600 -0.50050500

C 1.80217800 0.70599300 0.08151300

H 1.80206700 0.92004900 1.15860900

H 2.17154900 -0.31512100 -0.05156100

Cl 0.80516300 -1.84693000 -1.97240600

C 2.79057900 1.66019400 -0.57786500

C 4.23238500 1.55803400 -0.00376000

F 2.36525200 2.93998100 -0.39797900

F 2.81309300 1.47765300 -1.94410500

O 4.65488200 2.50359600 0.64878500

C 4.53639900 -0.73470900 -1.02474900

H 3.62278600 -0.50117100 -1.57645300

H 5.32045000 -0.89139800 -1.78484300

C 4.33932400 -2.01493400 -0.21907000

H 3.58174700 -1.88047300 0.56938900

H 3.99361100 -2.81721800 -0.88902500

H 5.27341100 -2.34886000 0.25718200

Cl -0.50531200 -0.51751400 2.14062600

H -4.90340000 -3.20880400 -0.42536000

H -3.82674500 -4.72515900 0.53808200

N 4.93734400 0.41866500 -0.21000300

C 6.27053600 0.35950400 0.40607900

H 6.83920700 -0.41538500 -0.12725300

H 6.77237900 1.32247400 0.22918700

C 6.23761100 0.06611600 1.90268800

H 7.26485300 0.02913300 2.30037800

H 5.69319800 0.85630900 2.44034600

H 5.75347600 -0.89911100 2.11491400

N -0.40594600 1.79571900 0.08819000

H -0.43599900 1.79378600 1.10573700

C -1.22783000 2.65574500 -0.60638700

O -1.17080200 2.75352900 -1.82728800

C -2.18904600 3.44293700 0.22207300

C -2.74979600 4.59854500 -0.34585500

C -2.58558200 3.04322400 1.51008900

C -3.67470500 5.35708800 0.37007700

H -2.44649100 4.89004600 -1.35286100

C -3.51958700 3.80006200 2.22040100

H -2.19376600 2.12761900 1.95902500

C -4.06093200 4.95904000 1.65532300

H -4.09944300 6.26024800 -0.07462000

H -3.82987200 3.47882300 3.21743500

H -4.78955500 5.55070800 2.21512700

H 0.35677400 0.87463700 -1.58972700

**^2^D3**

C 4.13287800 0.89534500 -2.45254800

C 4.99955300 1.72686400 -1.74426300

C 4.54421100 2.33812400 -0.57762100

C 3.23644400 2.09022200 -0.14187000

C 2.84568000 0.69614800 -1.95443400

C 2.65833000 2.72373700 1.06672400

C 3.37833600 3.59865500 1.89038900

C 2.75234700 4.14999500 3.00661000

H 3.29877200 4.83361900 3.65987100

C 1.42536100 3.81332100 3.27523200

C 0.77399600 2.93631300 2.40967500

H 4.43728200 0.40352100 -3.37778800

H 6.01727200 1.90685700 -2.09709000

H 2.14055500 0.06699800 -2.49609800

H 0.89646500 4.21759700 4.13971100

H -0.25628200 2.62438000 2.58044200

N 2.41599900 1.26360500 -0.82325100

N 1.37478700 2.41342200 1.33552100

Ni 0.51241900 1.00296900 0.07578700

C -0.24320600 -0.44149100 -1.10308800

C -1.67739100 -0.67038100 -0.65301200

H -1.72212000 -1.39483800 0.17261300

H -2.10009000 0.26879800 -0.29250800

C -2.57245900 -1.19255400 -1.76178700

C -4.00427000 -1.57145700 -1.28846400

F -2.02836500 -2.31085400 -2.31816900

F -2.64463800 -0.27737800 -2.79802400

O -4.31422700 -2.75651700 -1.29696300

C -4.57560700 0.84918400 -0.83425800

H -3.67704700 1.06618200 -1.41715300

H -5.41093200 1.33154900 -1.36963400

C -4.44962200 1.44552800 0.56433000

H -3.61651300 0.98856600 1.12045000

H -4.25914400 2.52692500 0.48604500

H -5.37184800 1.31154600 1.14944900

H 5.19886900 3.00308900 -0.01630100

H 4.41503400 3.84569400 1.66616700

N -4.82943100 -0.59551400 -0.83451400

C -6.13664200 -1.03484600 -0.32737700

H -6.79786200 -0.15670300 -0.33311300

H -6.55050500 -1.76853700 -1.03521500

C -6.07441500 -1.64916000 1.06775900

H -7.08488200 -1.94558000 1.39334200

H -5.44003500 -2.54757300 1.06654400

H -5.67167000 -0.93793300 1.80470300

N 0.57833200 -1.59594200 -1.00129100

H 0.70556100 -1.96380400 -0.06371900

C 1.27512300 -2.16454500 -2.02097100

O 1.16267700 -1.80412400 -3.19326600

C 2.21558900 -3.26387100 -1.62198100

C 2.60261700 -4.19078500 -2.60199600

C 2.75487400 -3.36600600 -0.32835800

C 3.49109300 -5.21994000 -2.28940700

H 2.19380300 -4.09040600 -3.60901700

C 3.65158500 -4.39145200 -0.01945500

H 2.49970400 -2.63240000 0.43984300

C 4.01683500 -5.32319500 -0.99631900

H 3.77824500 -5.94341800 -3.05648700

H 4.07098000 -4.45813300 0.98737600

H 4.71615000 -6.12675700 -0.75197500

H -0.18244300 -0.03723200 -2.11524200

C -1.12231700 3.30558600 -0.24495500

C 0.03458100 2.69392600 -2.20877300

C -2.14492200 4.06221500 -1.09711800

H -0.34944000 4.04703000 0.06701800

H -1.61968000 2.94370300 0.66916000

C -0.98656300 3.47516500 -3.03963700

H 0.89874800 3.37326200 -2.00984100

H 0.41523900 1.86365200 -2.82487000

H -2.52941400 4.94156100 -0.55501800

H -2.99703400 3.39369800 -1.33153500

H -0.50898400 3.92206900 -3.92677100

H -1.77894900 2.77733900 -3.38202500

N -0.58091700 2.19457600 -0.99989800

O -1.55790700 4.53679200 -2.29641100

O 1.07722100 -0.43868800 1.43827700

C 0.04362600 -0.34324500 2.18568300

O -0.86398800 0.48093700 1.92479500

C -0.06743800 -1.25478300 3.36897600

C -1.18854000 -1.17481100 4.20933300

C 0.93399400 -2.20079600 3.63894600

C -1.30655600 -2.02941500 5.30666100

H -1.96201100 -0.43729500 3.98869100

C 0.81430300 -3.05592100 4.73597700

H 1.80216100 -2.25883300 2.98056800

C -0.30564200 -2.97136200 5.57092700

H -2.18158700 -1.96371000 5.95810300

H 1.59566000 -3.79192500 4.94115300

H -0.39891900 -3.64170800 6.42924300

**TS1a-R1**

C 1.46635900 -1.62375400 0.08191900

O 1.43594300 -2.49135900 0.94578400

C 2.64860800 -0.72664500 -0.11149200

C 3.50638100 -0.53823400 0.98407800

C 2.93465900 -0.08410700 -1.32725500

C 4.61556300 0.30059700 0.87589800

H 3.28192600 -1.05228500 1.92026300

C 4.05161200 0.74651800 -1.43695100

H 2.29978900 -0.23826700 -2.20220100

C 4.88962700 0.94612000 -0.33517000

H 5.26990900 0.45219100 1.73782200

H 4.26962200 1.23770200 -2.38825700

H 5.75958300 1.60190000 -0.42160600

N 0.39855000 -1.39307900 -0.75749500

H 0.43183400 -0.59588100 -1.38688000

C -0.76627400 -2.13516900 -0.71956800

C -1.87958000 -1.82558300 -1.43303700

H -0.73488600 -2.96688200 -0.01366400

H -1.86602800 -1.04983300 -2.20287400

H -2.72368300 -2.51656400 -1.42398900

C -3.21041700 -0.34822800 -0.14493000

C -2.55195900 0.96270000 -0.36667100

F -4.35041300 -0.52982700 -0.80631800

F -3.28037000 -0.94750100 1.04401600

O -2.68691400 1.45216000 -1.49260000

N -1.73686700 1.50633500 0.58240700

C -1.67426000 1.11466500 1.99526300

C -0.91603600 2.64569800 0.15393200

H -1.51309900 2.04039100 2.56804200

H -2.65032100 0.72742600 2.30846000

C -0.57144400 0.11250200 2.31872400

H -0.64558400 3.20993000 1.05731800

H -1.53533400 3.29956900 -0.47701200

C 0.33991900 2.23449500 -0.60676500

H 0.41594000 0.48928400 2.01122100

H -0.54040300 -0.07047400 3.40524300

H -0.74408800 -0.85028300 1.81891400

H 0.91381200 3.12684200 -0.90544500

H 0.99182000 1.59996100 0.00921800

H 0.07566600 1.68257700 -1.52157500

**TS^2^B1-^3^A1**

C 0.55287300 3.39341200 -1.88082100

C 1.59461200 3.81583400 -1.05410700

C 2.30511900 2.86689800 -0.32045100

C 1.95336700 1.51810400 -0.43125100

C 0.25354500 2.03388400 -1.93502800

C 2.63807800 0.42598900 0.29384100

C 3.66327400 0.62296000 1.22399000

C 4.21743200 -0.48623500 1.86301700

H 5.01354100 -0.35303200 2.59852600

C 3.74245900 -1.76061200 1.55017600

C 2.72173100 -1.87999100 0.60840100

H -0.02751100 4.10034300 -2.47552200

H 1.85508400 4.87370500 -0.98044100

H -0.56709400 1.65822900 -2.54740300

H 4.15053400 -2.65383100 2.02576200

H 2.31043800 -2.84694700 0.31137200

N 0.93219300 1.12224300 -1.22599500

N 2.18376100 -0.81366500 0.00643000

Ni 0.57491000 -0.85040500 -1.19146200

Cl -1.65764500 -0.64913900 -1.60924900

C -3.32428400 -0.23826400 -0.13815300

C -2.69284700 -0.61041700 1.18276800

F -4.28041500 -1.05890600 -0.52138900

F -3.70202600 1.01470800 -0.35974600

O -2.95581900 -1.72225900 1.63178200

Cl 0.69985800 -3.03046100 -1.79267400

H 4.01954400 1.62659000 1.45378800

H 3.12573100 3.17276700 0.32736900

N -1.82541500 0.26026400 1.75379800

C -1.42900500 1.56508500 1.21702000

H -1.51428000 1.55162700 0.12678500

H -0.35749100 1.68567500 1.43494100

C -2.21985900 2.72764900 1.80487100

H -2.11468300 2.77290300 2.90047000

H -1.85049900 3.67891800 1.38912700

H -3.28973900 2.63707700 1.56349100

C -1.19995400 -0.15172100 3.01387500

H -1.90389600 -0.80725600 3.54243800

H -1.05765100 0.75144700 3.62593800

C 0.12981800 -0.86626900 2.79924700

H 0.55304900 -1.18137800 3.76649900

H -0.00326000 -1.76046500 2.17218900

H 0.85967600 -0.20570800 2.31026600

**TS^2^B1-^2^D1**

C 2.79066700 0.80287300 -0.43783800

C 4.11347500 0.89324900 -0.88719900

C 5.08583800 0.09086800 -0.29159400

C 4.71890900 -0.77648100 0.73891700

C 3.38083300 -0.81497600 1.12708300

H 4.38383800 1.58280600 -1.68573800

H 6.12311200 0.14793800 -0.62814700

H 5.45011100 -1.41720300 1.23398300

H 3.03989800 -1.48132600 1.92296900

C 1.68083200 1.62638300 -0.98048700

C 1.82685000 2.53783300 -2.03214900

C 0.71441100 3.26432600 -2.45661400

H 2.79243300 2.68015300 -2.51593000

C -0.58523000 2.14484200 -0.78319300

C -0.51452300 3.06939200 -1.82390000

H 0.81005500 3.97896500 -3.27682200

H -1.51691400 1.94642300 -0.24931200

H -1.40665600 3.62031000 -2.12523300

N 2.45002800 -0.05446900 0.54521300

N 0.48357200 1.44828600 -0.38460100

Ni 0.46831100 0.08705300 1.12933100

Cl 1.16275400 1.29506700 3.02140800

C 0.39339500 -2.93165900 0.17476600

C -0.60212500 -1.46839600 -1.46530000

C 1.57347300 -3.11806600 -0.78237200

H -0.34079100 -3.73609300 -0.00316900

H 0.73485600 -2.98910300 1.21868200

C 0.63751600 -1.65868100 -2.33372400

H -1.34667100 -2.23015800 -1.75014700

H -1.03892300 -0.47251400 -1.61849200

H 1.95287100 -4.14481300 -0.67318100

H 2.39278500 -2.42570700 -0.53026900

H 0.34769400 -1.58163400 -3.39199400

H 1.38937100 -0.87585300 -2.12890700

N -0.26285900 -1.64050900 -0.04735300

O 1.18774500 -2.94543800 -2.13122400

O -1.67547600 -1.84131000 0.67205600

C -2.18509100 -0.63747600 0.98613600

O -1.45456300 0.16333500 1.67202800

C -3.48146200 -0.29129400 0.46196300

C -4.08538500 0.94469300 0.82912300

C -4.21355100 -1.16005100 -0.39525500

C -5.34308500 1.29303600 0.34696300

H -3.53893800 1.61545600 1.49445200

C -5.46792100 -0.79636800 -0.87140500

H -3.77340600 -2.11708400 -0.67936100

C -6.04838300 0.43186000 -0.50903000

H -5.78441300 2.25074400 0.63817300

H -6.00776300 -1.47678000 -1.53640400

H -7.03557600 0.71056400 -0.88540300

**TS^3^C-^2^B1**

C 2.36644000 1.44491100 0.08253200

C 2.65853000 2.82522200 -0.07042800

C 1.66532600 3.76684200 0.11701800

C 0.35763000 3.34070700 0.46404100

C 0.13063100 1.98107900 0.60213400

H 3.66796300 3.13919700 -0.33892900

H 1.88721600 4.83003800 -0.00262500

H -0.45526700 4.05213000 0.61589600

H -0.86053400 1.60141500 0.86473400

C 3.31490900 0.37537400 -0.09208000

C 4.68004200 0.55181300 -0.43322500

C 5.50789500 -0.54363700 -0.57921000

H 5.07312900 1.55890500 -0.57797800

C 3.63971400 -1.96352200 -0.04926700

C 4.97699400 -1.84579400 -0.38333900

H 6.55967700 -0.40777500 -0.84173200

H 3.18613500 -2.94600100 0.11278000

H 5.59613900 -2.73787600 -0.48945000

N 1.08552400 1.05132700 0.42159700

N 2.81525100 -0.90462300 0.09669800

Ni 0.94527600 -0.90898500 0.56685300

Cl -1.11983000 -1.50715200 1.22798000

C -2.11850200 -1.86506300 -0.44974100

C -2.53561400 -0.52563800 -1.08758300

F -1.25459200 -2.50651500 -1.21390600

F -3.10038300 -2.69851500 -0.12362600

O -1.93314700 -0.20120200 -2.10298400

N -3.50934800 0.20224600 -0.50396600

C -4.19309800 -0.10035000 0.75940700

C -3.93541100 1.41469400 -1.22239200

H -5.27589900 -0.11980100 0.55308700

H -3.92700300 -1.10582300 1.09421200

C -3.88952300 0.90291800 1.86646800

H -4.94229600 1.66006400 -0.85606000

H -4.02330400 1.15697800 -2.28837000

C -2.99946200 2.60704300 -1.06092300

H -4.24104600 1.91424800 1.61418700

H -4.40389700 0.58977800 2.78876100

H -2.80950900 0.94952600 2.07385400

H -3.39450100 3.46086800 -1.63479300

H -2.90981300 2.91587200 -0.00966900

H -1.99685500 2.37407200 -1.44456000

**TS^3^C-^3^A2**

C 3.29436700 -1.31649200 0.05057500

C 4.30854900 -2.27465500 0.17533500

C 3.96841100 -3.57788800 0.53464400

C 2.62464000 -3.89457900 0.75564800

C 1.67141800 -2.88892100 0.61265400

H 5.35041400 -2.00997500 -0.00323900

H 4.74532300 -4.33827800 0.63956100

H 2.31640200 -4.90366900 1.03489700

H 0.60502700 -3.07046800 0.76528900

C 3.52672000 0.09444600 -0.32968300

C 4.79393600 0.61203000 -0.62475900

C 4.91761200 1.94989000 -0.99385800

H 5.67596600 -0.02527300 -0.57103400

C 2.54082400 2.15512700 -0.74880000

C 3.76640000 2.73827300 -1.06059800

H 5.89842200 2.36879100 -1.22842200

H 1.62340100 2.74368600 -0.79333500

H 3.80949400 3.78967800 -1.34980200

N 1.99973000 -1.63428700 0.27297800

N 2.41322700 0.86857000 -0.38763500

Ni 0.69209200 -0.11533700 0.09421200

C -1.27304500 1.99260400 -0.85212400

C -0.46086100 2.36891500 1.41356100

C -2.35718700 3.02906200 -0.58248400

H -0.42117100 2.46812400 -1.36645400

H -1.64754200 1.19100400 -1.50742500

C -1.59130200 3.37509000 1.59538500

H 0.45637900 2.90043100 1.11293200

H -0.24965700 1.83156700 2.34884300

H -2.59393000 3.56424200 -1.51449900

H -3.27992700 2.53183100 -0.23038700

H -1.27104500 4.16985700 2.28603800

H -2.47914300 2.87825900 2.02965500

O -1.94148200 0.51730900 0.99833000

C -2.01902400 -0.66436500 0.35756700

O -0.94186600 -1.31677900 0.13383600

C -3.32445200 -1.09277500 -0.06478600

C -3.49535600 -2.38750900 -0.64126000

C -4.49140300 -0.28887300 0.10676200

C -4.75230400 -2.83466900 -1.03212500

H -2.61488500 -3.01903600 -0.77330000

C -5.73857200 -0.74760600 -0.29776400

H -4.39113400 0.69739600 0.56226700

C -5.89014100 -2.02444100 -0.87159700

H -4.85420500 -3.83040100 -1.47462000

H -6.61475300 -0.10610600 -0.16317600

H -6.87525100 -2.38096400 -1.18200800

N -0.72988500 1.38023600 0.36662100

O -1.92460800 3.99211300 0.36318100

**TS^2^B2-^2^D3**

C 3.14567100 0.88319400 -3.73699800

C 4.46064100 0.87068200 -3.26957900

C 4.69540300 0.86378900 -1.89520100

C 3.60234700 0.87562300 -1.02038100

C 2.11176400 0.86884200 -2.80239900

C 3.73586700 0.89672000 0.45865400

C 4.96208200 0.81321100 1.12897400

C 4.97183100 0.84458000 2.52320000

H 5.91759400 0.77297000 3.06447200

C 3.76406600 0.96199400 3.21320200

C 2.58308200 1.02732400 2.47433600

H 2.91894100 0.89152200 -4.80429900

H 5.30033400 0.86699500 -3.96803100

H 1.06532800 0.84806700 -3.11502000

H 3.73083700 0.98818700 4.30356500

H 1.59876900 1.08871200 2.94490400

N 2.33763600 0.87041000 -1.48740000

N 2.57706900 0.99386200 1.13954100

Ni 0.83786100 0.66488500 -0.03712100

C -0.11020400 -0.90741200 -0.90732400

C -1.53831600 -1.23872000 -0.53365300

H -1.64697500 -1.34721100 0.55503700

H -2.19287400 -0.40532000 -0.81347900

C -2.08803400 -2.50267500 -1.17419600

C -3.53221800 -2.89277000 -0.75611000

F -1.28481900 -3.55958200 -0.85294400

F -2.01766700 -2.41454500 -2.55632700

O -3.67752400 -3.87938900 -0.04361200

C -4.50002100 -0.91291600 -1.99136100

H -3.51201400 -0.84775100 -2.45326200

H -5.21624600 -1.03684700 -2.82087700

C -4.81253100 0.36886500 -1.22415600

H -4.14388100 0.48694300 -0.35915900

H -4.67900300 1.23989200 -1.88564600

H -5.84943100 0.38353300 -0.85660500

H 5.71549700 0.85566500 -1.51279300

H 5.89522300 0.71653300 0.57582800

N -4.57230300 -2.12070100 -1.16346400

C -5.91011200 -2.55157000 -0.73571500

H -6.63295100 -2.08644200 -1.42168200

H -5.97409600 -3.64145000 -0.87288900

C -6.24528300 -2.20115600 0.71029300

H -7.25320900 -2.57130500 0.96016200

H -5.52851800 -2.67341200 1.39716900

H -6.22685800 -1.11572000 0.88143600

N 0.87878700 -1.66840900 -0.15266200

H 0.68390600 -1.80559700 0.83410600

C 2.04204000 -2.16217700 -0.66123000

O 2.26499000 -2.20182200 -1.87515000

C 3.09603300 -2.55382300 0.33696500

C 4.31040200 -3.04166000 -0.17446500

C 2.96879900 -2.38907700 1.72784800

C 5.36979200 -3.35774500 0.67627900

H 4.40456900 -3.15154000 -1.25572000

C 4.02835400 -2.70665500 2.57955500

H 2.05460400 -1.98985600 2.16969300

C 5.23319800 -3.18963100 2.05847200

H 6.30888900 -3.73164700 0.26024600

H 3.91495900 -2.56201000 3.65658000

H 6.06368200 -3.42937800 2.72718800

H 0.08766100 -1.04965400 -1.97481700

C 0.80379200 3.64334600 -0.43908600

C -1.15176200 2.50751800 -1.33784700

C 0.06625500 4.96158100 -0.63598200

H 1.43835800 3.44158500 -1.31694100

H 1.45042100 3.66970300 0.44876300

C -1.79889400 3.87434500 -1.52301300

H -0.67030200 2.18546500 -2.27666300

H -1.90029700 1.74783900 -1.07255800

H 0.78990200 5.76103700 -0.85491900

H -0.48074800 5.23479800 0.28616600

H -2.45301500 3.85387100 -2.40759700

H -2.42228800 4.12224800 -0.64397800

N -0.09856200 2.49606400 -0.31786900

O -0.82612500 4.88291300 -1.73456800

O -0.34280700 0.60256100 1.68148700

C -1.21445700 1.53672000 1.59528400

O -0.83665900 2.73132100 1.09459600

C -2.60285200 1.38075300 1.94433100

C -3.56647000 2.40853600 1.73131700

C -3.04768700 0.17901500 2.56859700

C -4.89461900 2.22824500 2.09845900

H -3.24797400 3.34303500 1.26803700

C -4.37821400 0.01663700 2.93769400

H -2.32124400 -0.61481000 2.74946300

C -5.32023500 1.03305500 2.70568300

H -5.61607400 3.02918600 1.91187300

H -4.69468200 -0.91839000 3.40842000

H -6.36503000 0.89812000 2.99491800

**TS^3^A1-^2^D2**

C 4.19630500 -0.19785500 -1.53724300

C 4.99379400 0.89598600 -1.20016100

C 4.39800100 2.03765300 -0.66295600

C 3.01509600 2.04921600 -0.45833900

C 2.82394700 -0.11511400 -1.30802000

C 2.26952100 3.21314900 0.07915400

C 2.86378000 4.43159900 0.42267500

C 2.05728300 5.46438100 0.90104100

H 2.50353000 6.42322100 1.17299700

C 0.68202500 5.26078500 1.02626700

C 0.15989100 4.01875900 0.66871100

H 4.61932800 -1.10563900 -1.96945300

H 6.07344500 0.86791700 -1.36109400

H 2.15664300 -0.93443500 -1.57594700

H 0.01970800 6.04526400 1.39509400

H -0.90755900 3.79907200 0.75080000

N 2.26245900 0.97052200 -0.76863300

N 0.93866000 3.03536200 0.21141100

Ni 0.32268100 1.15949600 -0.23838700

C -0.84718700 -1.82356500 -0.64533100

C -2.08318800 -1.34292700 0.03263500

H -2.09730200 -1.63304500 1.09307100

H -2.14856300 -0.24621300 0.00819800

Cl -0.80596100 1.41092400 -2.18495200

C -3.35166200 -1.88743600 -0.60619300

C -4.65946300 -1.40304500 0.08031700

F -3.33903200 -3.24684300 -0.55007200

F -3.38235000 -1.58473900 -1.95284200

O -5.31250600 -2.22705400 0.70802000

C -4.31406800 0.95475700 -0.76913700

H -3.53927600 0.51011700 -1.39751500

H -5.05459600 1.39396700 -1.45923900

C -3.70389000 2.05444800 0.09484900

H -2.97283900 1.64231900 0.80841800

H -3.17908400 2.77155300 -0.55371400

H -4.46837600 2.60378400 0.66458900

Cl 0.31221000 0.36763200 1.92056100

H 5.00266000 2.90738400 -0.40843600

H 3.93826100 4.57577500 0.31663400

N -4.99656900 -0.09173000 0.00112800

C -6.21301000 0.30911900 0.72168700

H -6.55487700 1.25522700 0.27854300

H -6.98844700 -0.44771300 0.53145400

C -6.00733400 0.46422500 2.22522400

H -6.95207700 0.76897700 2.70420000

H -5.69135500 -0.48963200 2.67268300

H -5.24678900 1.22630400 2.45290000

N 0.19842700 -2.32768100 0.07150100

H 0.22262700 -2.11782500 1.06774100

C 1.28137700 -2.95420100 -0.50465600

O 1.28376200 -3.23732800 -1.70501300

C 2.44840900 -3.22262100 0.38441800

C 3.39900400 -4.15942200 -0.05764700

C 2.67618200 -2.53317100 1.58938300

C 4.53891800 -4.42355600 0.70047100

H 3.22581700 -4.67211000 -1.00526400

C 3.82219900 -2.79619700 2.34288000

H 1.98600800 -1.75830100 1.93064000

C 4.75356900 -3.74324700 1.90501300

H 5.26642800 -5.16012700 0.35036900

H 3.99305400 -2.24828400 3.27286600

H 5.64957600 -3.94571800 2.49711600

H -0.79267100 -1.94953100 -1.72412200

**TS^3^A2-^2^B3**

C 5.33836500 -1.76078800 -0.13318400

C 6.26222500 -0.75320300 -0.41049200

C 5.79720500 0.51986800 -0.73682800

C 4.41766900 0.75547000 -0.76634200

C 3.98029400 -1.45376600 -0.19390200

C 3.82739800 2.07609400 -1.09208900

C 4.58244700 3.19130400 -1.47284600

C 3.92513400 4.38463300 -1.77040800

H 4.49816800 5.26473100 -2.07018400

C 2.53284200 4.43588000 -1.68185600

C 1.84487000 3.28809100 -1.29044800

H 5.65376900 -2.77261200 0.12563400

H 7.33529100 -0.95305100 -0.37555000

H 3.23096200 -2.21882400 0.00447000

H 1.98103600 5.34965700 -1.90892100

H 0.75455000 3.25852200 -1.19698300

N 3.53106200 -0.22861500 -0.49314400

N 2.48171500 2.14424100 -1.00240400

Ni 1.54944100 0.40536800 -0.50845300

C -0.85342500 -1.89503700 -0.03433700

C -2.14291500 -1.14136600 -0.09158100

H -2.25768800 -0.52191900 0.80843500

H -2.18798500 -0.45191500 -0.94373400

C -3.35954800 -2.05114200 -0.14150500

C -4.70789900 -1.30748900 0.08460700

F -3.25775700 -2.99119900 0.83887000

F -3.38421200 -2.77227000 -1.32016500

O -5.27333400 -1.48393500 1.15688100

C -4.58789900 -0.20779000 -2.18654500

H -3.80020900 -0.93743200 -2.38142800

H -5.37260200 -0.39952000 -2.93787100

C -4.04611000 1.20772200 -2.35710100

H -3.26821300 1.44212300 -1.61443700

H -3.60100800 1.30827300 -3.36002400

H -4.84307200 1.96224200 -2.27183800

H 6.50235200 1.31961200 -0.95948600

H 5.66785800 3.13239300 -1.54060700

N -5.17334800 -0.46244100 -0.86675900

C -6.42258700 0.24482400 -0.55074400

H -6.85440400 0.58000100 -1.50468900

H -7.12066300 -0.47891100 -0.10445000

C -6.23141400 1.42423700 0.39667700

H -7.20168600 1.90742000 0.59694800

H -5.81398300 1.08692000 1.35572000

H -5.55432800 2.17828200 -0.02936700

N -0.03759900 -1.64636500 1.02418500

H -0.12889100 -0.71428500 1.43131200

C 1.07491900 -2.39442200 1.33537400

O 1.28797500 -3.48229600 0.78688400

C 2.00220900 -1.82395900 2.35408000

C 2.99084700 -2.67966800 2.87536400

C 1.99966900 -0.47206600 2.75019700

C 3.94891600 -2.20264100 3.76739200

H 2.99812200 -3.72142800 2.55148300

C 2.96573800 0.00368700 3.63970400

H 1.27042900 0.23307300 2.34890400

C 3.94358200 -0.85562500 4.15117800

H 4.70917600 -2.88119500 4.16264200

H 2.95645300 1.05792300 3.92781000

H 4.69982900 -0.47768300 4.84364900

H -0.74046200 -2.86801800 -0.50461100

C -0.22980400 -0.08723800 -2.64550300

C 1.32626300 -1.86887200 -2.31581200

C -1.05517400 -1.01676800 -3.53347400

H 0.43399700 0.49796200 -3.32130000

H -0.87606800 0.64571200 -2.14077000

C 0.46813600 -2.74160600 -3.22871100

H 2.13985000 -1.43529800 -2.93806200

H 1.79241000 -2.51345300 -1.55844800

H -1.57421700 -0.43998800 -4.31491100

H -1.81697600 -1.54811300 -2.93401700

H 1.10391500 -3.45180500 -3.78113300

H -0.25180600 -3.32933900 -2.62394900

N 0.57619900 -0.80137600 -1.65996200

O -0.22949400 -1.96665100 -4.18702200

O 0.07518800 1.14454300 0.70749700

C -0.91877000 1.92214000 0.47018600

O -1.13652300 2.50293900 -0.60344200

C -1.89307400 2.10992700 1.61564300

C -2.97053800 2.99464800 1.45940500

C -1.75494000 1.41184300 2.82613500

C -3.89725600 3.17521200 2.48789500

H -3.06698300 3.53238800 0.51506800

C -2.68617700 1.58435700 3.85306800

H -0.91455100 0.73038000 2.96461500

C -3.75953300 2.46644400 3.68634500

H -4.73493500 3.86395700 2.35281100

H -2.57285200 1.03043800 4.78843000

H -4.48790500 2.60116600 4.49013400

# Supplementary Reference

1. S. Tu, C. Zhang, "Facile Preparation of N -Vinylisobutyramide and N -Vinyl-2-pyrrolidinone," *Organic Process Research & Development* 19 (2015): 2045–2049. DOI: 10.1021/acs.oprd.5b00303.

2. S. Ge, S. I. Arlow, M. G. Mormino, J. F. Hartwig, "Pd-catalyzed α-arylation of trimethylsilyl enolates of α,α-difluoroacetamides," *Journal of the American Chemical Society* 136 (2014): 14401–14404. DOI: 10.1021/ja508590k.

3. O. V. Stanko, Y. V. Rassukana, K. A. Zamulko, V. V. Dyakonenko, S. V. Shishkina, P. P. Onys’ko, "Diastereoselective synthesis of polyfluoroalkylated α-aminophosphonic acid derivatives," *Journal of Fluorine Chemistry* 216 (2018): 47–56. DOI: 10.1016/j.jfluchem.2018.10.001.

4. T. Ferrary, E. David, G. Milanole, T. Besset, P. Jubault, X. Pannecoucke, "A straightforward and highly diastereoselective access to functionalized monofluorinated cyclopropanes via a Michael initiated ring closure reaction," *Organic Letters* 15 (2013): 5598–5601. DOI: 10.1021/ol402837u.

5. A. M. Berman, Johnson Jeffrey S., "Copper-catalyzed electrophilic amination of diorganozinc reagents: 4-phenylmorpholine," *Organic Syntheses* 83 (2006): 31. DOI: 10.15227/orgsyn.083.0031.

6. D. Yang, H. Huang, H. Zhang, L.-M. Yin, M.-P. Song, J.-L. Niu, "Regioselective Intermolecular Hydroamination of Unactivated Alkenes: “Co–H” Enabled Remote Functionalization," *ACS Catalysis* 11 (2021): 6602–6613. DOI: 10.1021/acscatal.1c00625.

7. S. Yotphan, D. Beukeaw, V. Reutrakul, "Synthesis of 2-aminobenzoxazoles via copper-catalyzed electrophilic amination of benzoxazoles with O-benzoyl hydroxylamines," *Tetrahedron* 69 (2013): 6627–6633. DOI: 10.1016/j.tet.2013.05.127.

8. B. Pérez-Saavedra, Á. Velasco-Rubio, E. Rivera-Chao, J. A. Varela, C. Saá, M. Fañanás-Mastral, "Catalytic Lewis Base Additive Enables Selective Copper-Catalyzed Borylative α-C-H Allylation of Alicyclic Amines," *Journal of the American Chemical Society* 144 (2022): 16206–16216. DOI: 10.1021/jacs.2c07969.

9. N. Niljianskul, S. Zhu, S. L. Buchwald, "Enantioselective synthesis of α-aminosilanes by copper-catalyzed hydroamination of vinylsilanes," *Angewandte Chemie International Edition* 54 (2015): 1638–1641. DOI: 10.1002/anie.201410326.

10. G. Liu, M. Zheng, R. Tian, Y. Zhou, "Site-Selective Synthesis of Antitumor C5-Aminated Indoles via Neighboring Aldehyde Group Assisted Catellani Reaction," *Organic Letters* 25 (2023): 9231–9236. DOI: 10.1021/acs.orglett.3c03932.

11. T. D. Svejstrup, A. Ruffoni, F. Juliá, V. M. Aubert, D. Leonori, "Synthesis of Arylamines via Aminium Radicals," *Angewandte Chemie International Edition* 56 (2017): 14948–14952. DOI: 10.1002/anie.201708693.

12. H. Shi, D. J. Babinski, T. Ritter, "Modular C-H functionalization cascade of aryl iodides," *Journal of the American Chemical Society* 137 (2015): 3775–3778. DOI: 10.1021/jacs.5b01082.

13. Q. Gou, G. Liu, Z.-N. Liu, J. Qin, "Pd(II) -Catalyzed Intermolecular Amination of Unactivated C(sp(3) )-H Bonds," *Chemistry - A European Journal* 21 (2015): 15491–15495. DOI: 10.1002/chem.201502375.

14. J. Zhou, Q. Yang, C. S. Lee, J. J. Wang, "Enantio- and Regioselective Construction of 1,4-Diamines via Cascade Hydroamination of Methylene Cyclopropanes," *Angewandte Chemie International Edition* 61 (2022): e202202160. DOI: 10.1002/anie.202202160.

15. S. Chen, P. Wang, H.-G. Cheng, C. Yang, Q. Zhou, "Redox-neutral ortho-C-H amination of pinacol arylborates via palladium(ii)/norbornene catalysis for aniline synthesis," *Chemical Science* 10 (2019): 8384–8389. DOI: 10.1039/C9SC02759A.

16. R. P. Rucker, A. M. Whittaker, H. Dang, G. Lalic, "Synthesis of hindered anilines: copper-catalyzed electrophilic amination of aryl boronic esters," *Angewandte Chemie International Edition* 51 (2012): 3953–3956. DOI: 10.1002/anie.201200480.

17. M. Li, D.-H. Wang, "Copper-Catalyzed 3-Positional Amination of 2-Azulenols with O-Benzoylhydroxylamines," *Organic Letters* 23 (2021): 6638–6641. DOI: 10.1021/acs.orglett.1c02132.

18. Z.-L. Yao, L. Wang, N.-Q. Shao, Y.-L. Guo, D.-H. Wang, "Copper-Catalyzed ortho -Selective Dearomative C–N Coupling of Simple Phenols with *O*-Benzoylhydroxylamines," *ACS Catalysis* 9 (2019): 7343–7349. DOI: 10.1021/acscatal.9b01317.

19. J. Jeon, C. Lee, H. Seo, S. Hong, "NiH-Catalyzed Proximal-Selective Hydroamination of Unactivated Alkenes," *Journal of the American Chemical Society* 142 (2020): 20470–20480. DOI: 10.1021/jacs.0c10333.

20. M. J. Frisch, G. W. Trucks, H. B. Schlegel et al., *Gaussian 16 Rev. C.01*, Wallingford, CT, **2016**.

21. L. Goerigk, S. Grimme, "Efficient and Accurate Double-Hybrid-Meta-GGA Density Functionals-Evaluation with the Extended GMTKN30 Database for General Main Group Thermochemistry, Kinetics, and Noncovalent Interactions," *Journal of Chemical Theory and Computation* 7 (2011): 291–309. DOI: 10.1021/ct100466k.

22. F. Weigend, R. Ahlrichs, "Balanced basis sets of split valence, triple zeta valence and quadruple zeta valence quality for H to Rn: Design and assessment of accuracy," *Physical Chemistry Chemical Physics : PCCP* 7 (2005): 3297–3305. DOI: 10.1039/B508541A.

23. F. Weigend, "Accurate Coulomb-fitting basis sets for H to Rn," *Physical Chemistry Chemical Physics : PCCP* 8 (2006): 1057–1065. DOI: 10.1039/B515623H.

24. A. Hellweg, C. Hättig, S. Höfener, W. Klopper, "Optimized accurate auxiliary basis sets for RI-MP2 and RI-CC2 calculations for the atoms Rb to Rn," *Theoretical Chemistry Accounts* 117 (2007): 587–597. DOI: 10.1007/s00214-007-0250-5.

25. F. Neese, "Software update: The ORCA program system—Version 5.0," *WIREs Computational Molecular Science* 12 (2022): e1606. DOI: 10.1002/wcms.1606.

26. C. Y. Legault, *CYLview20*, Université de Sherbrooke, **2020 (http://www.cylview.org)**.

27. B. Xiong, T. Wang, H. Sun et al., "Nickel-Catalyzed Cross-Electrophile Coupling Reactions for the Synthesis of gem -Difluorovinyl Arenes," *ACS Catalysis* 10 (2020): 13616–13623. DOI: 10.1021/acscatal.0c03993.

# Characterization Spectra for compounds 1-3.

**2,3,4,5,6-pentafluoro-N-vinylbenzamide (1d).**

**
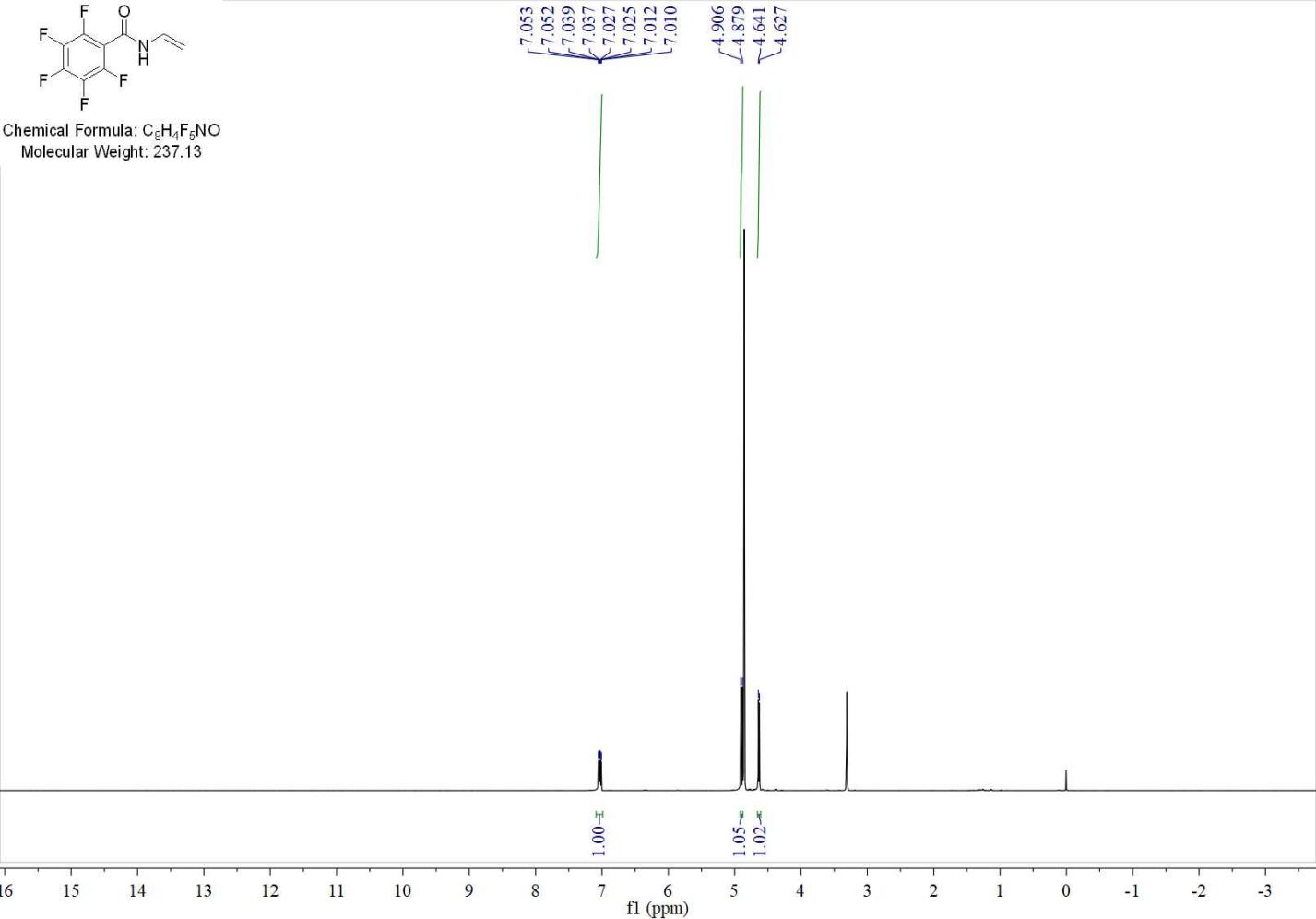

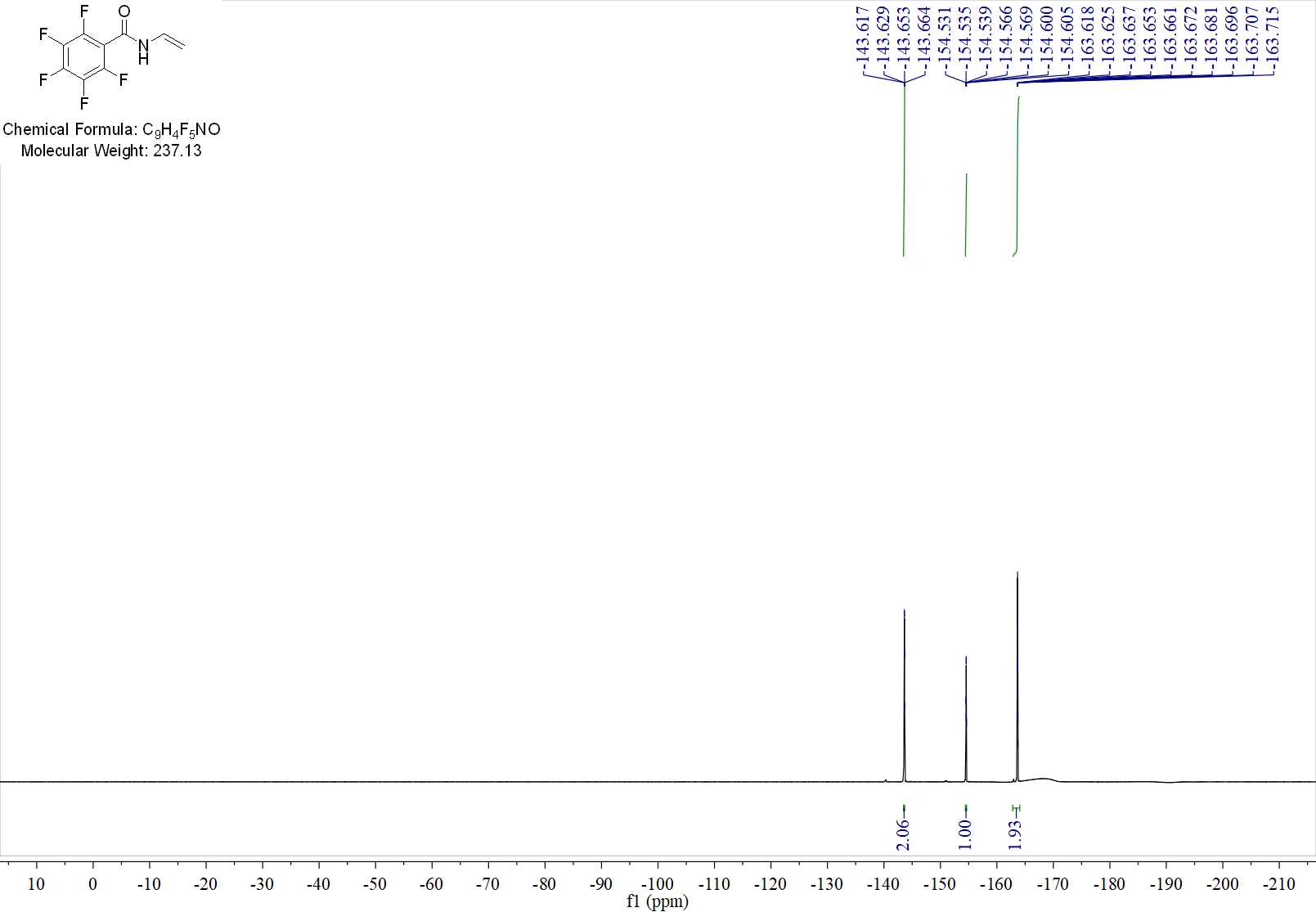
**


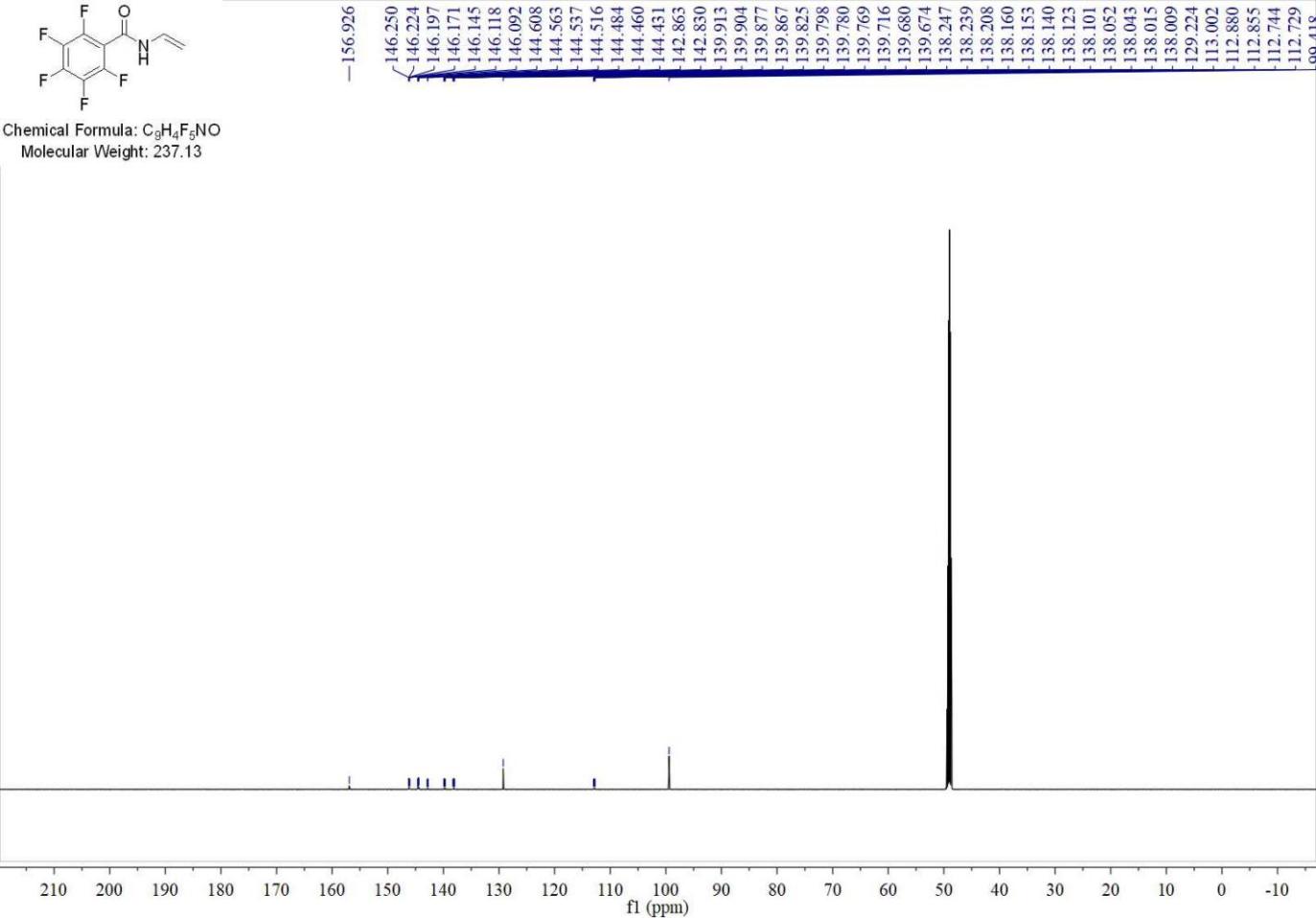


***N*, *N*-dibutyl-2-chloro-2,2-difluoroacetamide (2b).**

**
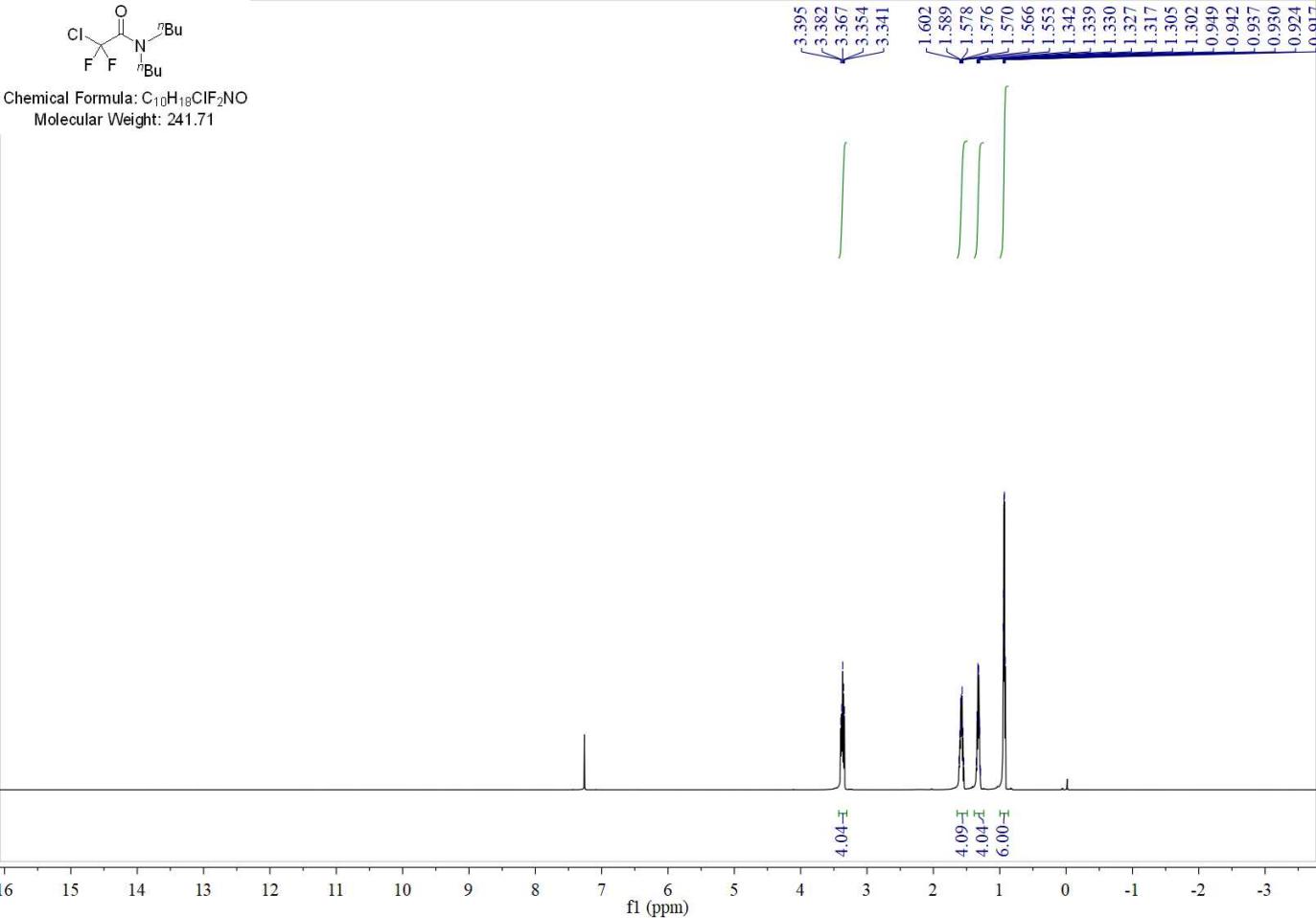
**

**
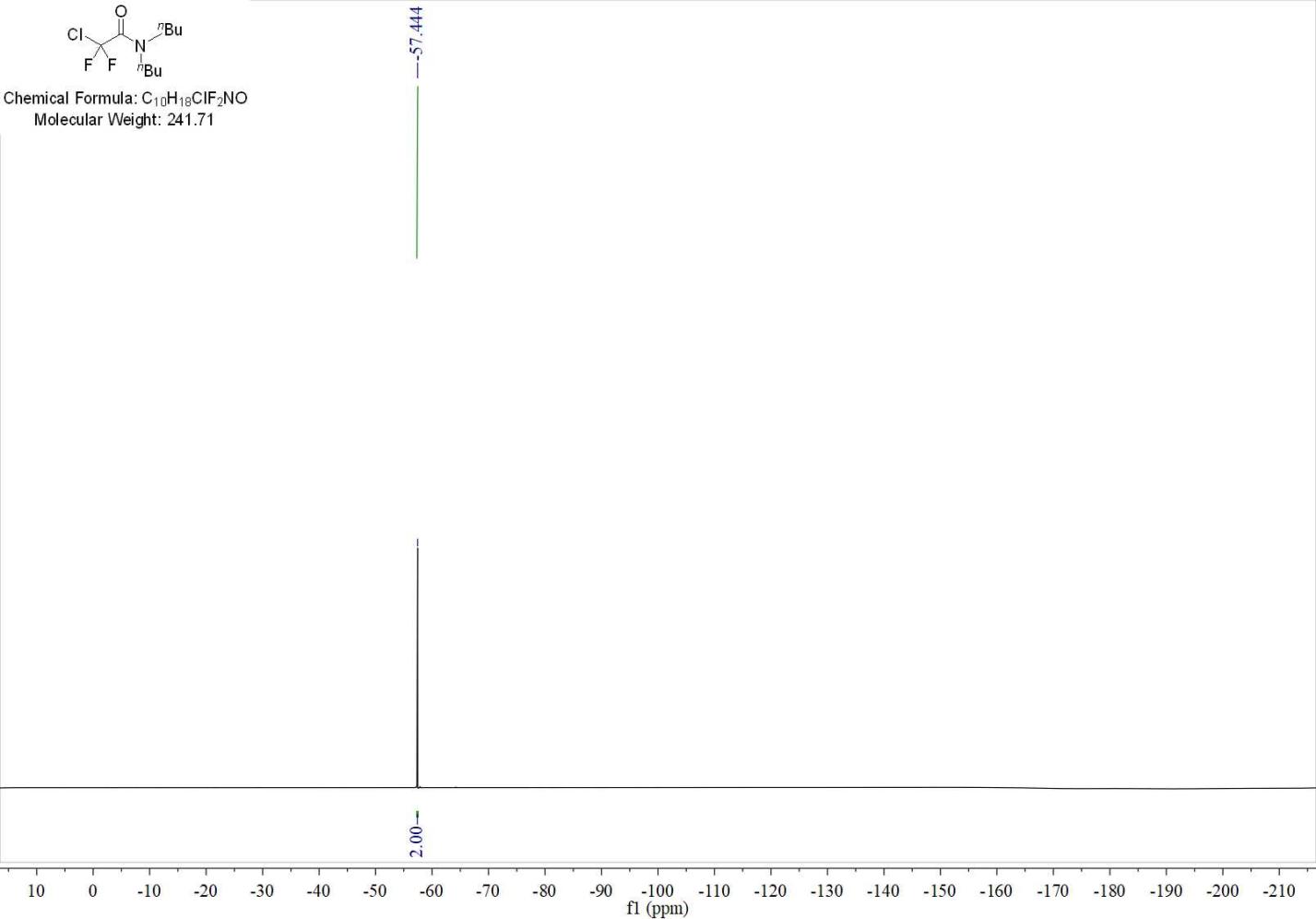
**

**
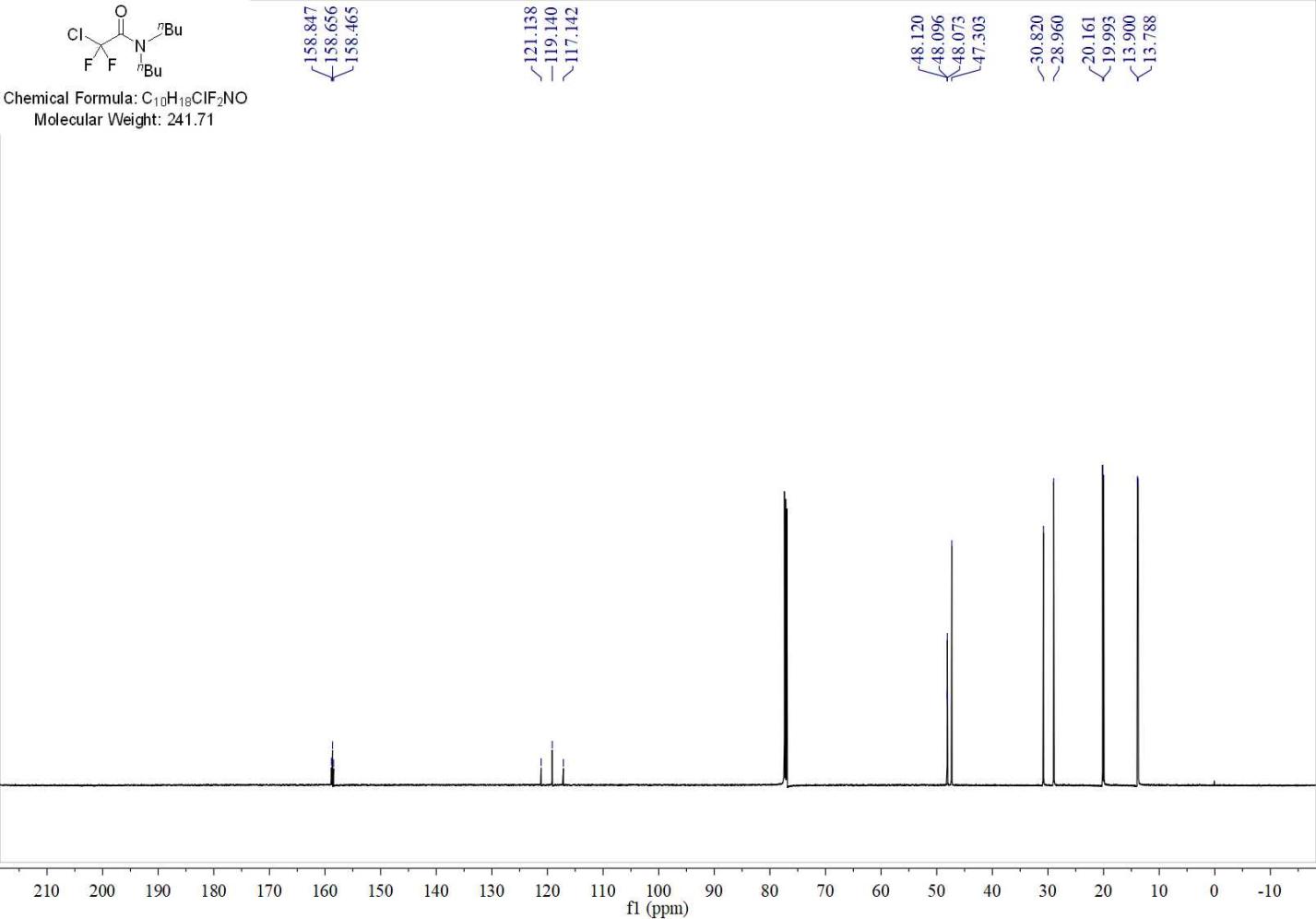
**

**1-(Azetidin-1-yl)-2-chloro-2,2-difluoroethan-1-one (2c).**


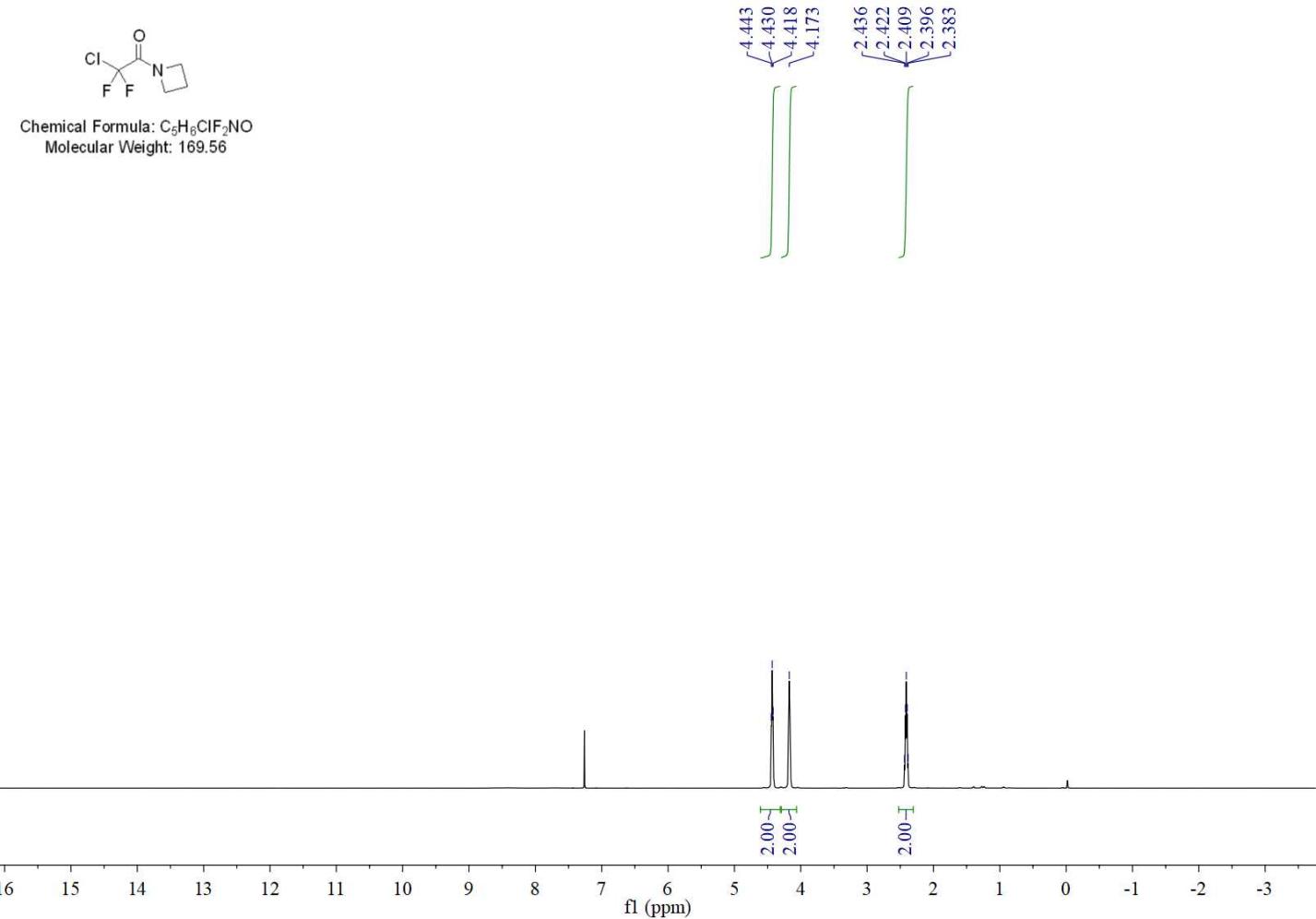

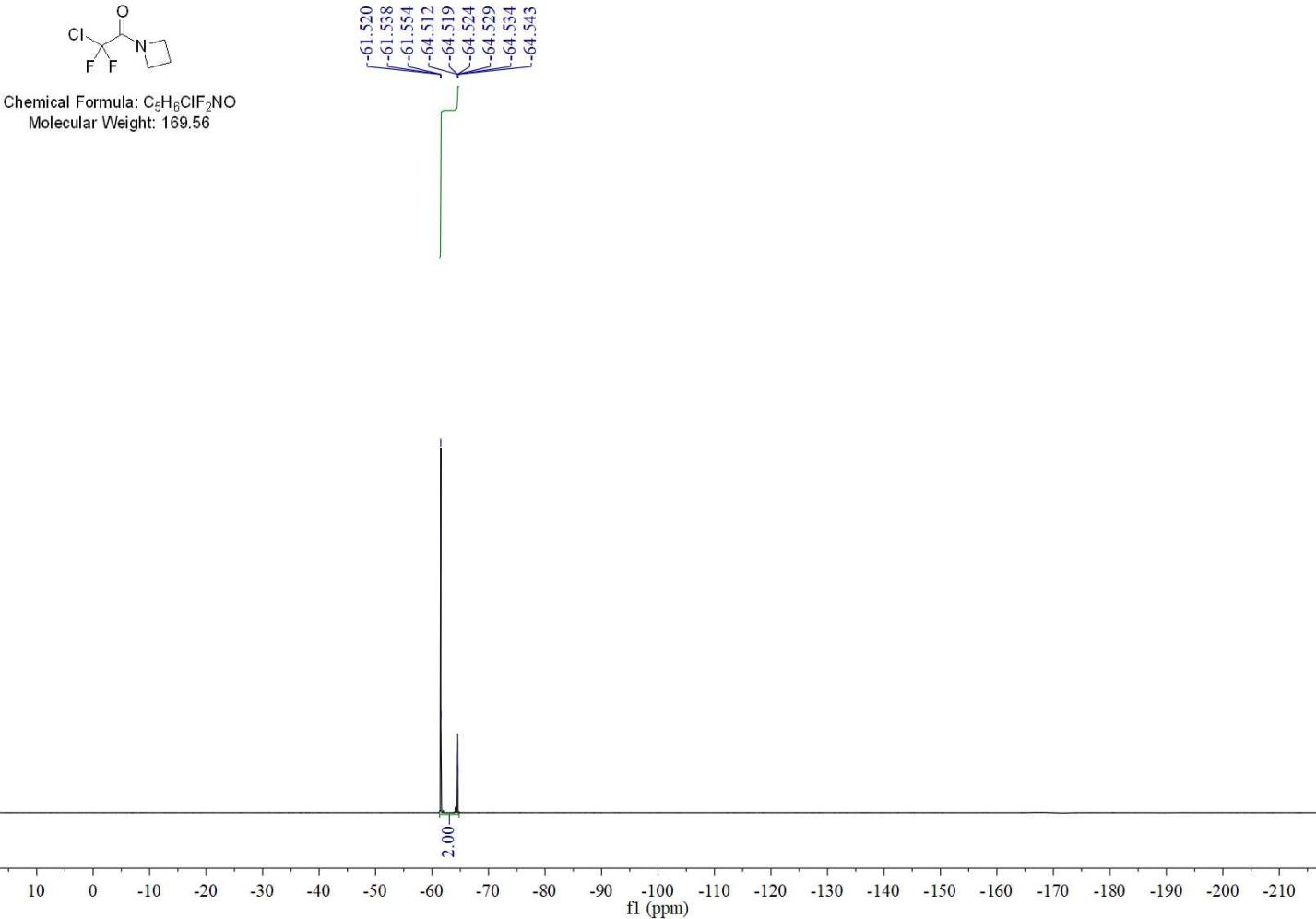


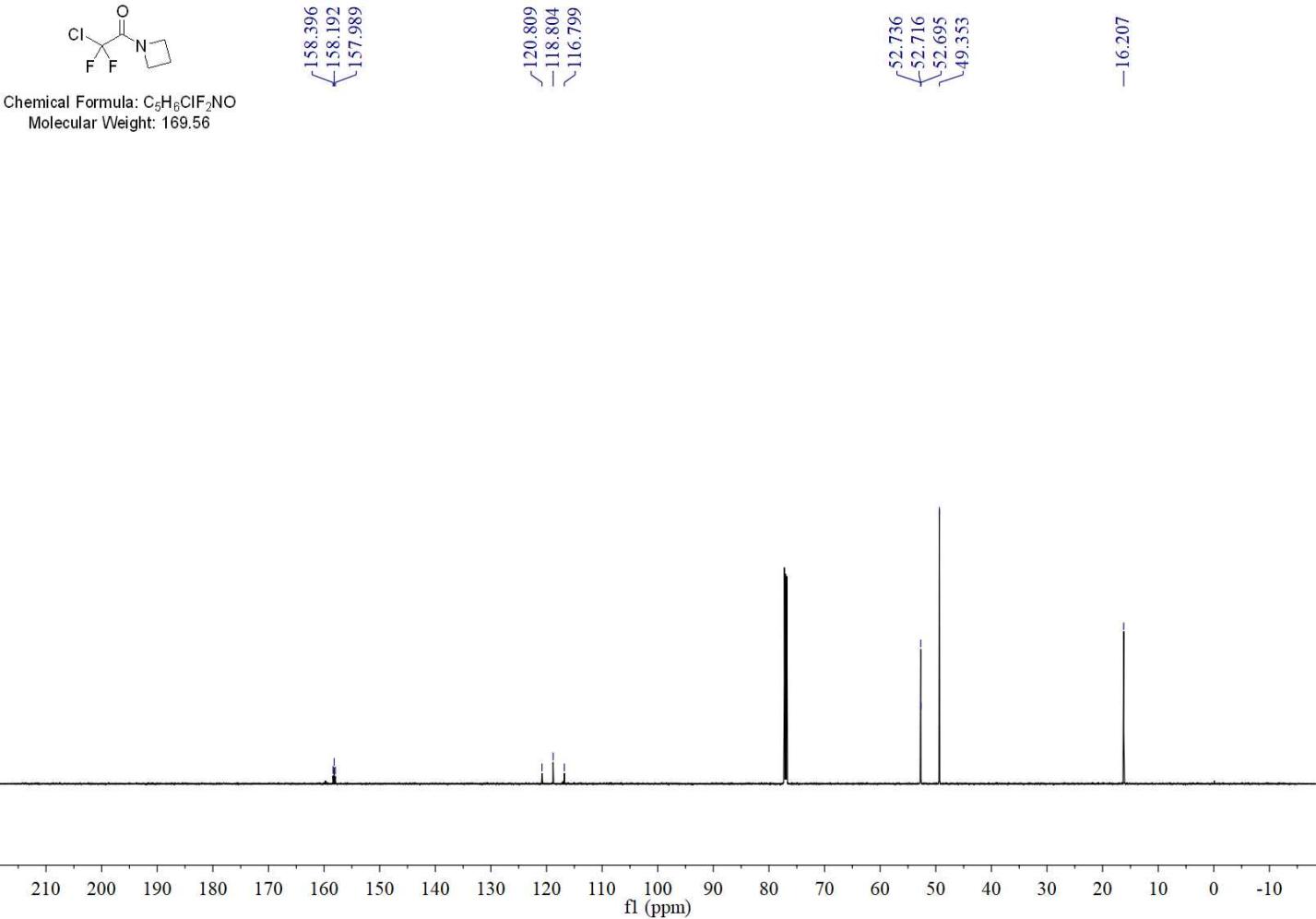


**2-chloro-2,2-difluoro-1-thiomorpholinoethan-1-one (2f).**


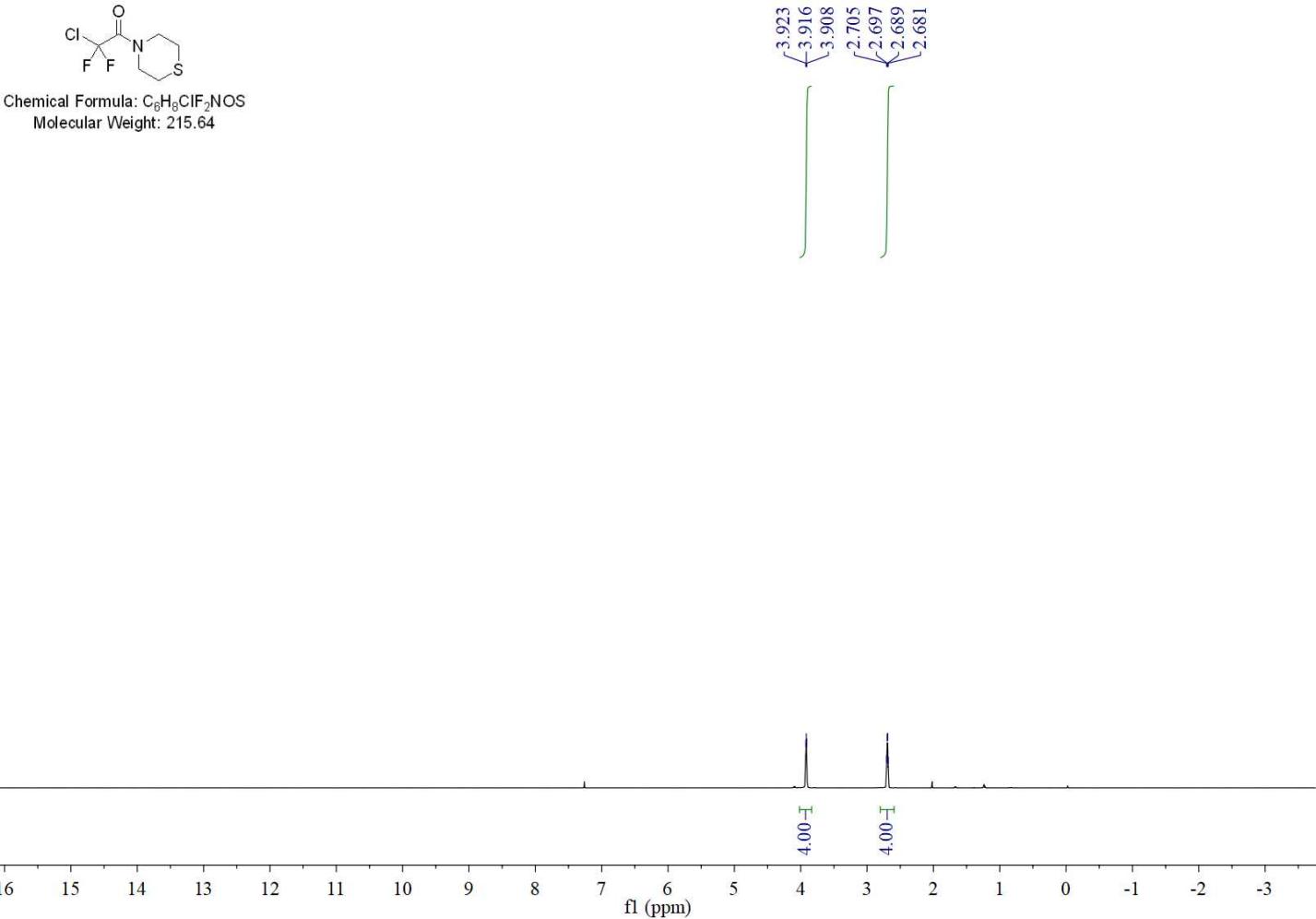

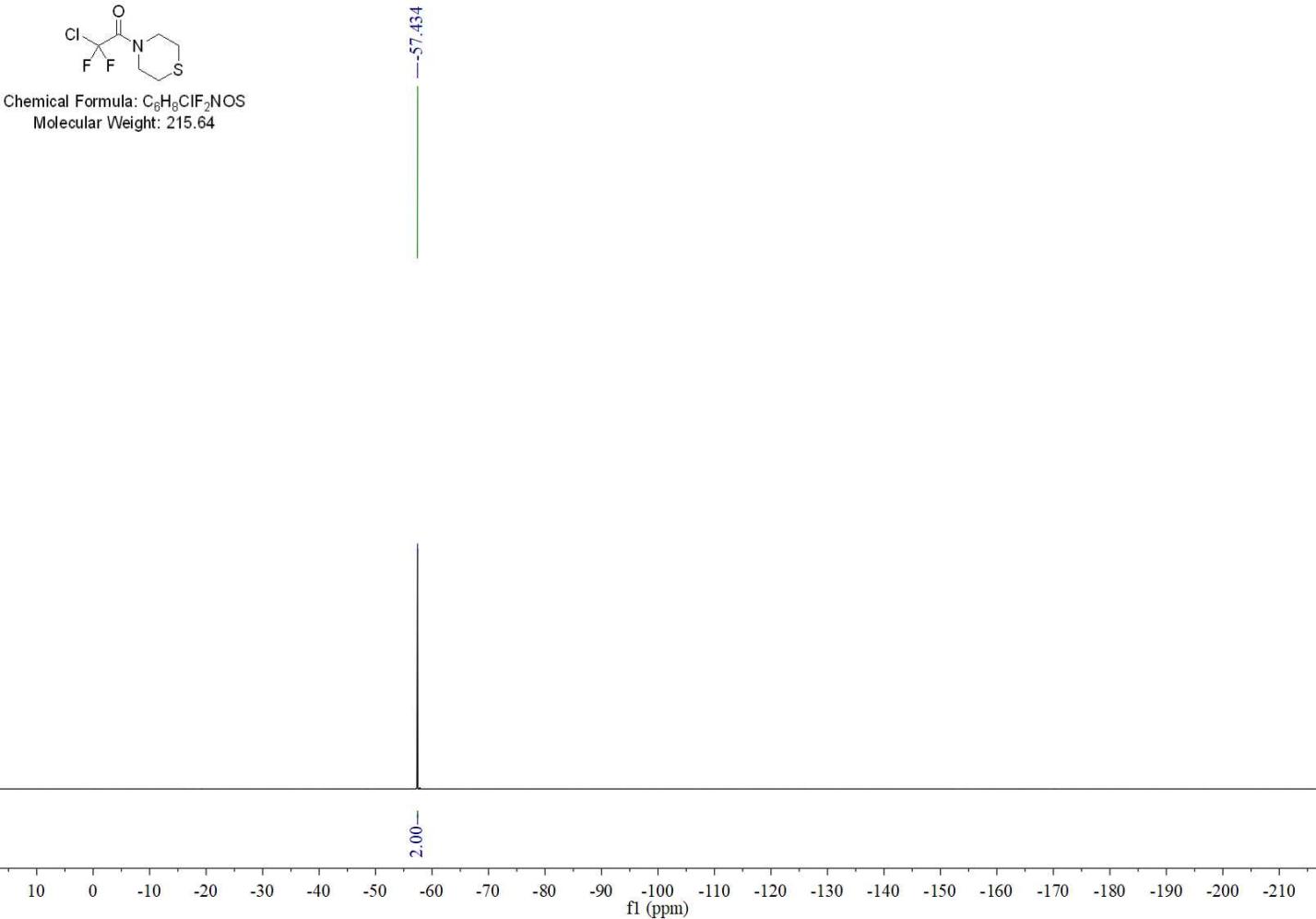

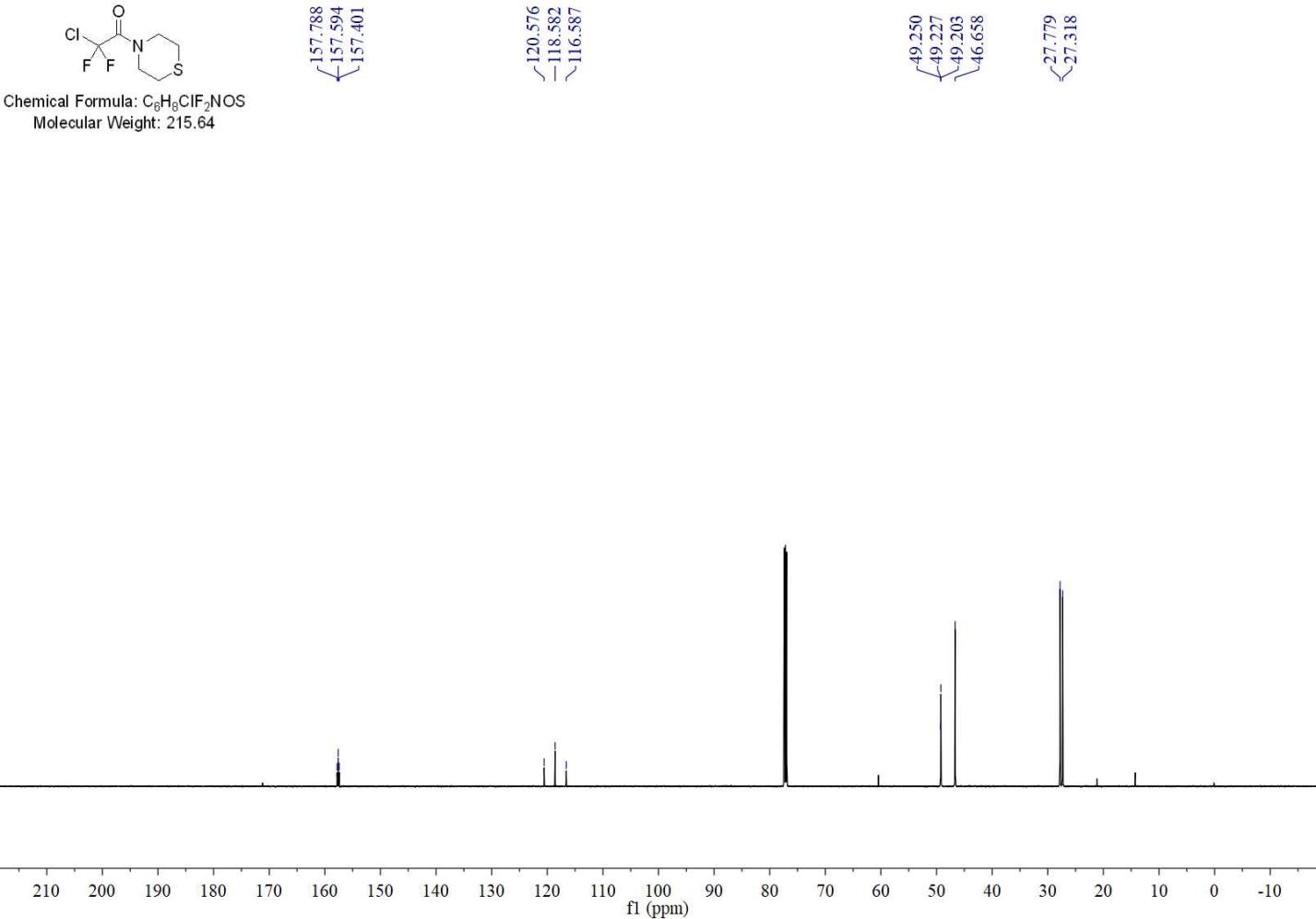


**2-chloro-1-(6,7-dimethoxy-3,4-dihydroisoquinolin-2(1H)-yl)-2,2-difluoroethan-1-one (2g).**

**
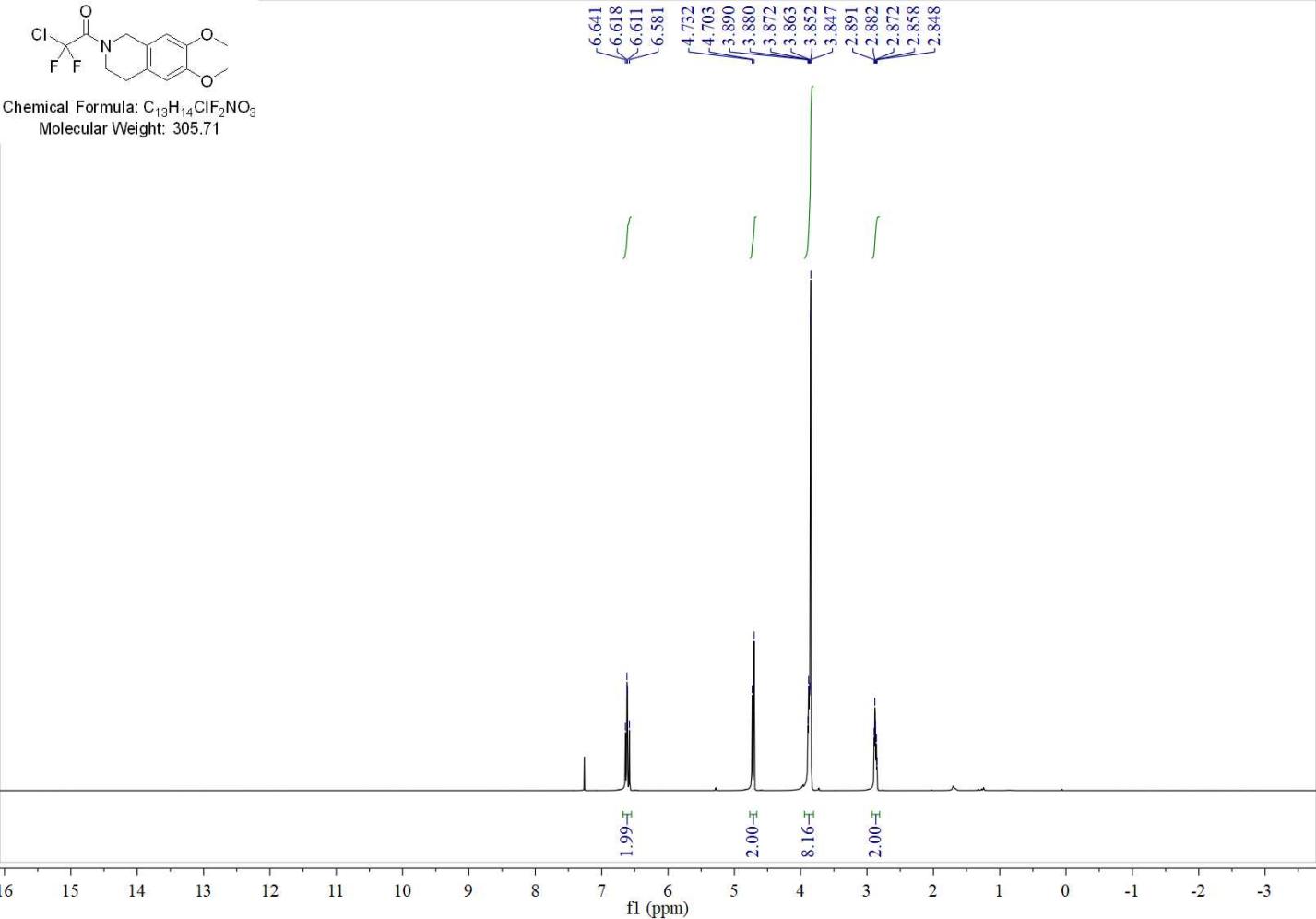

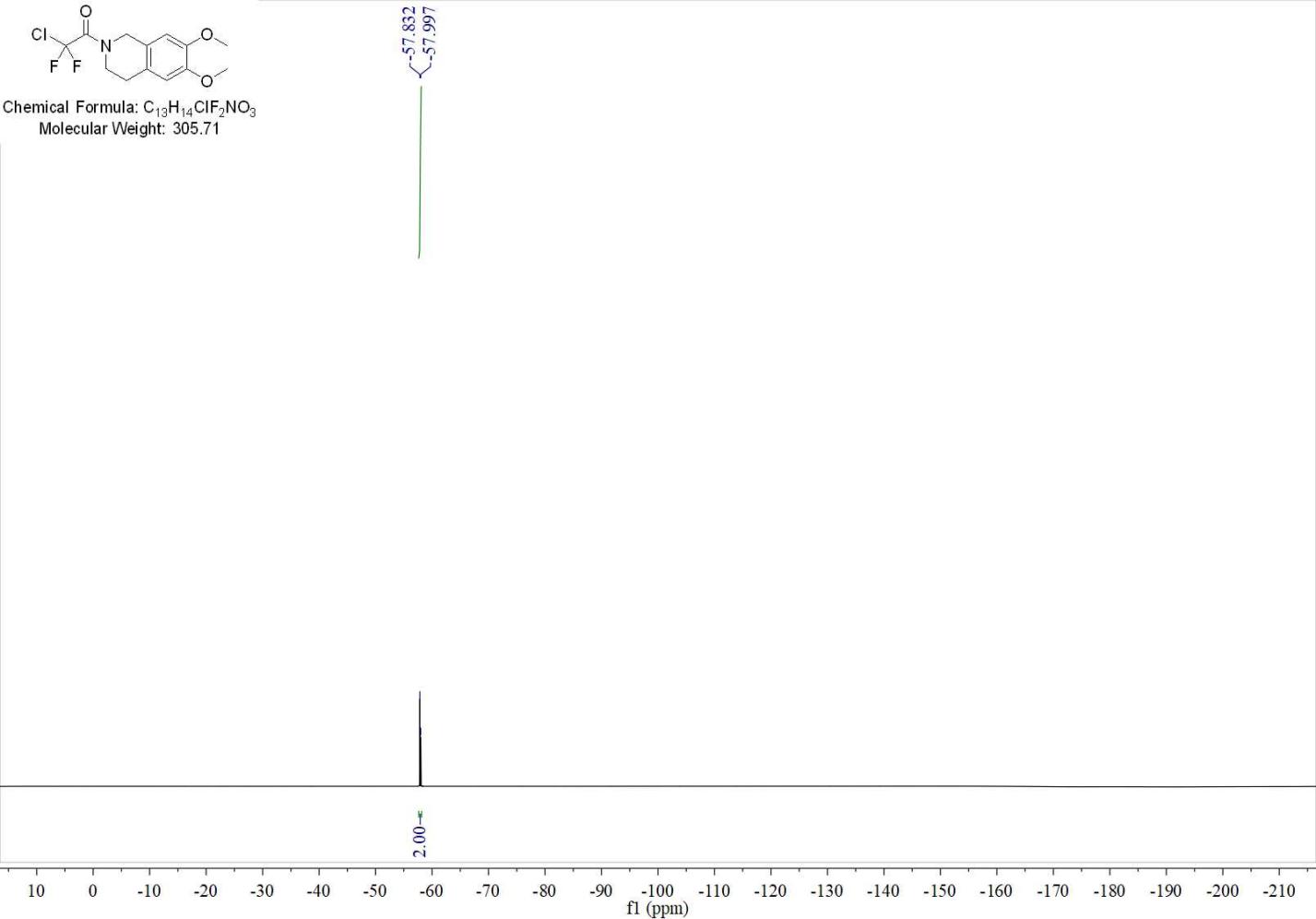

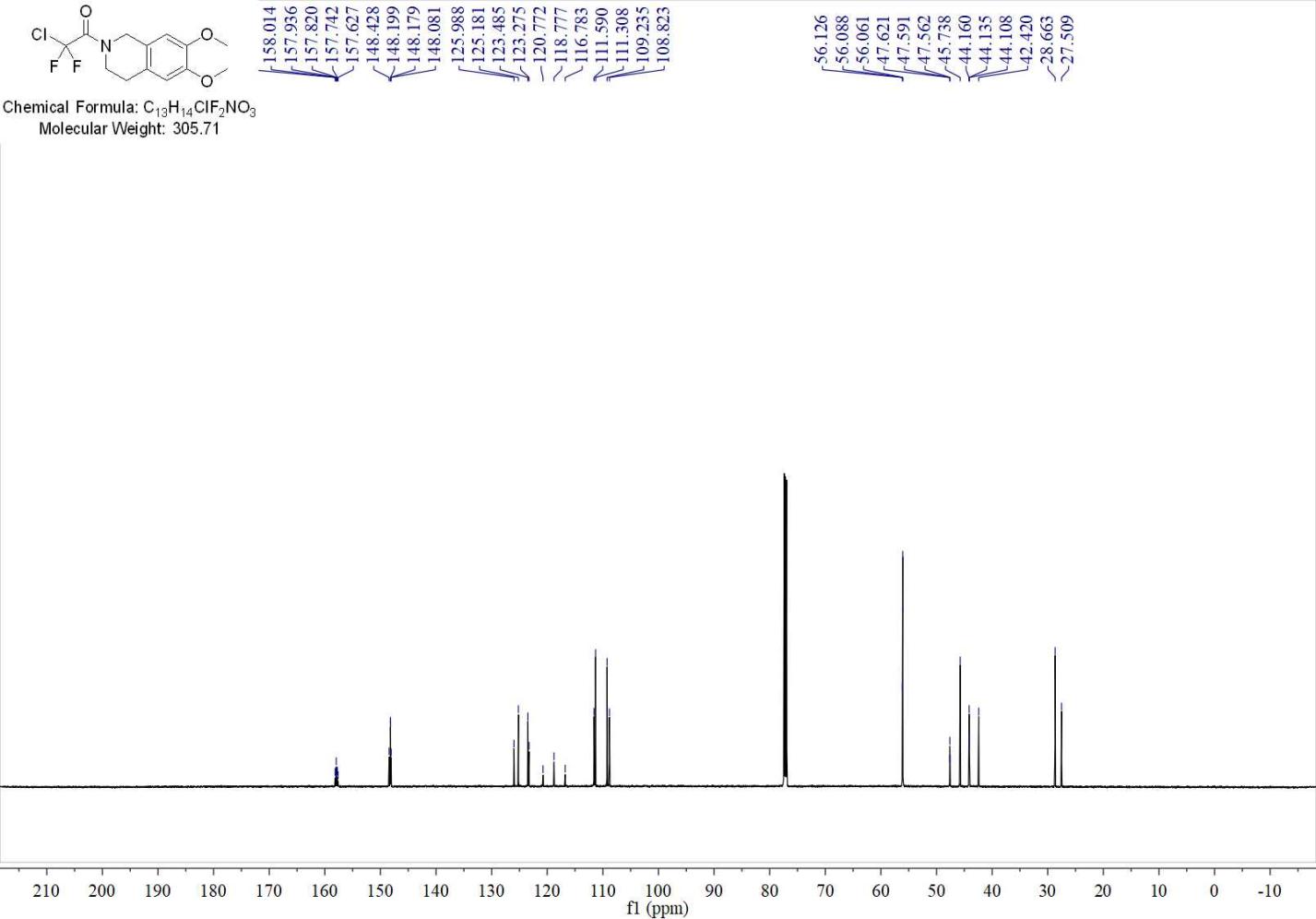
**

**1-(azepan-1-yl)-2-chloro-2,2-difluoroethan-1-one (2h).**


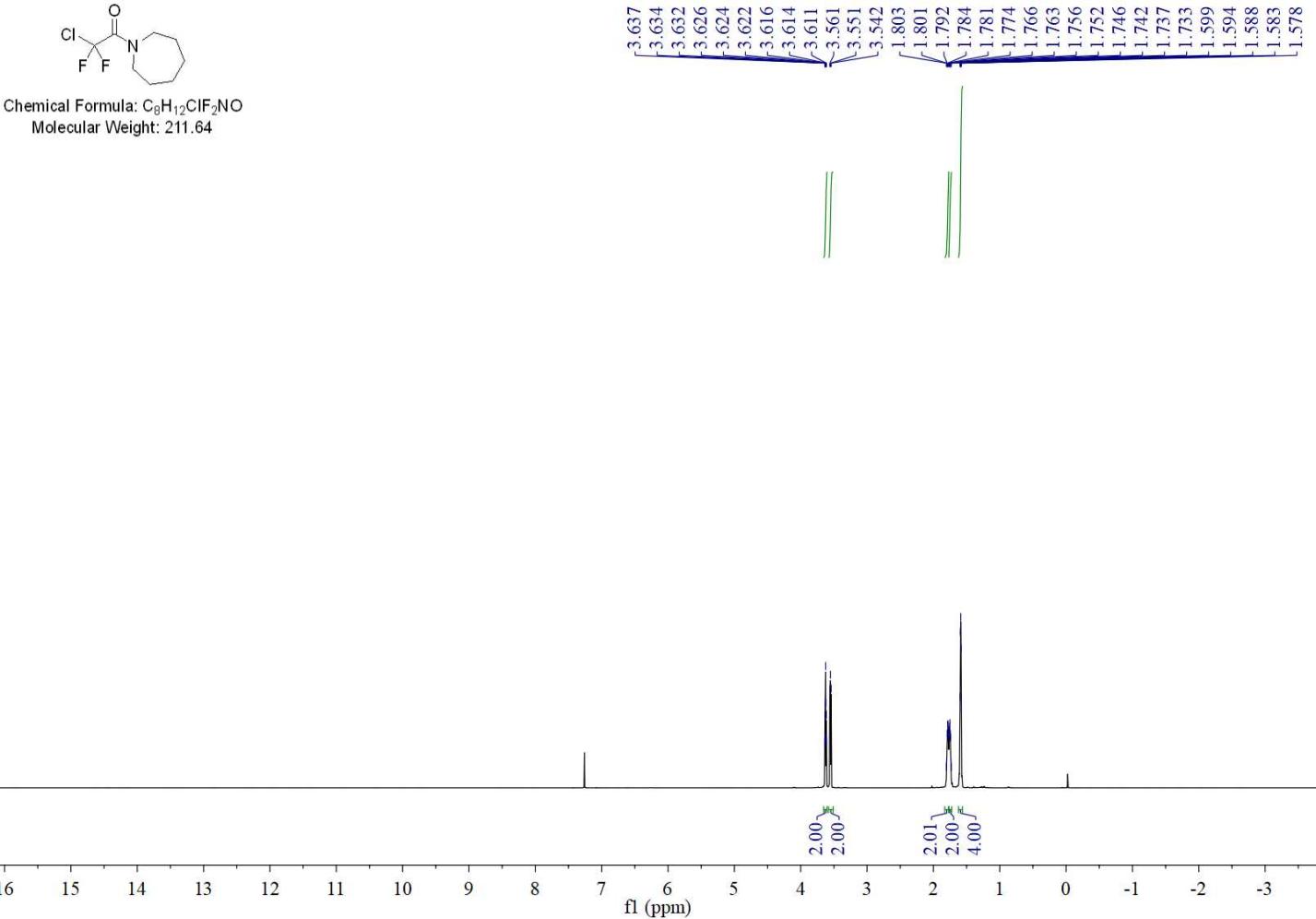

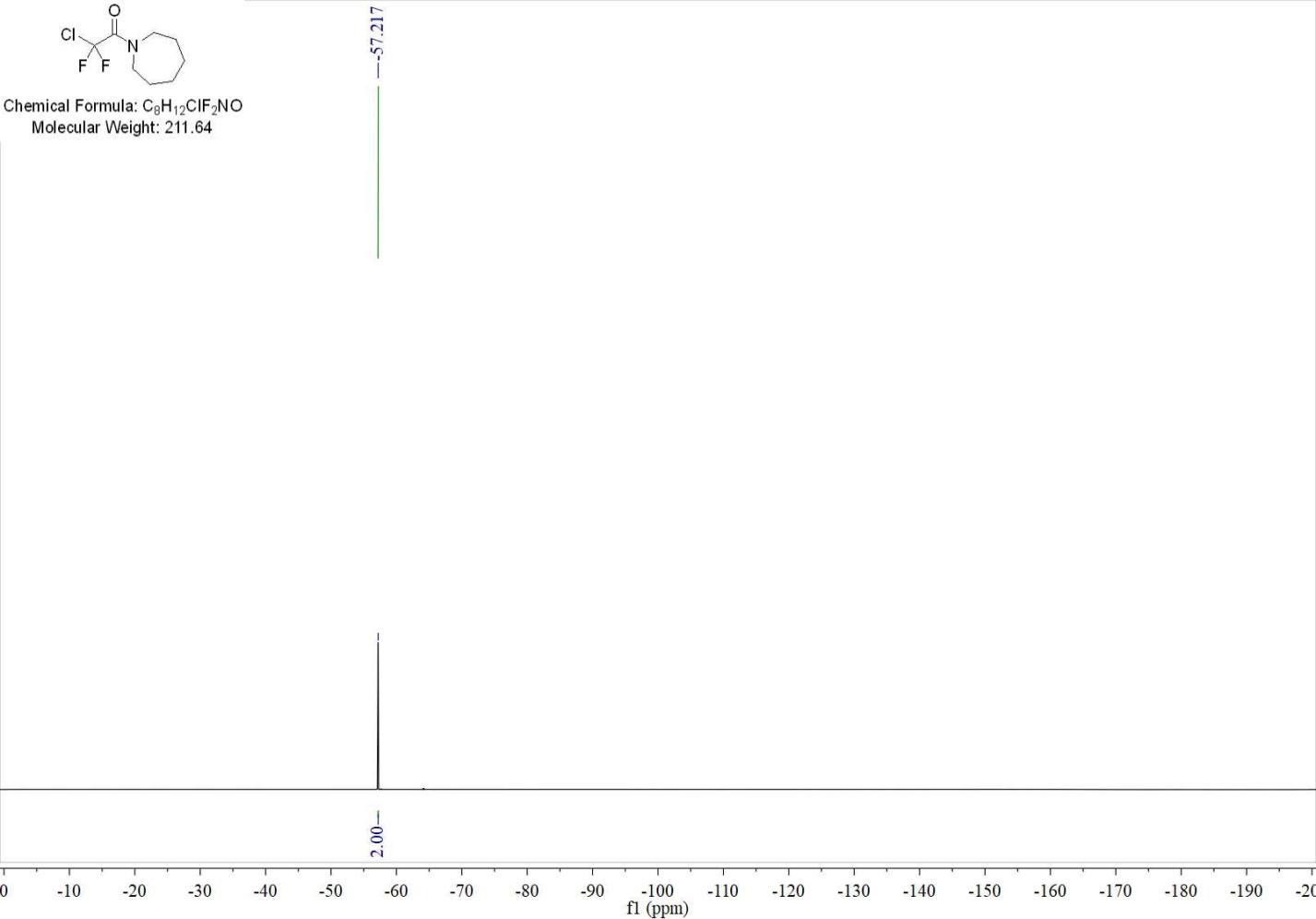


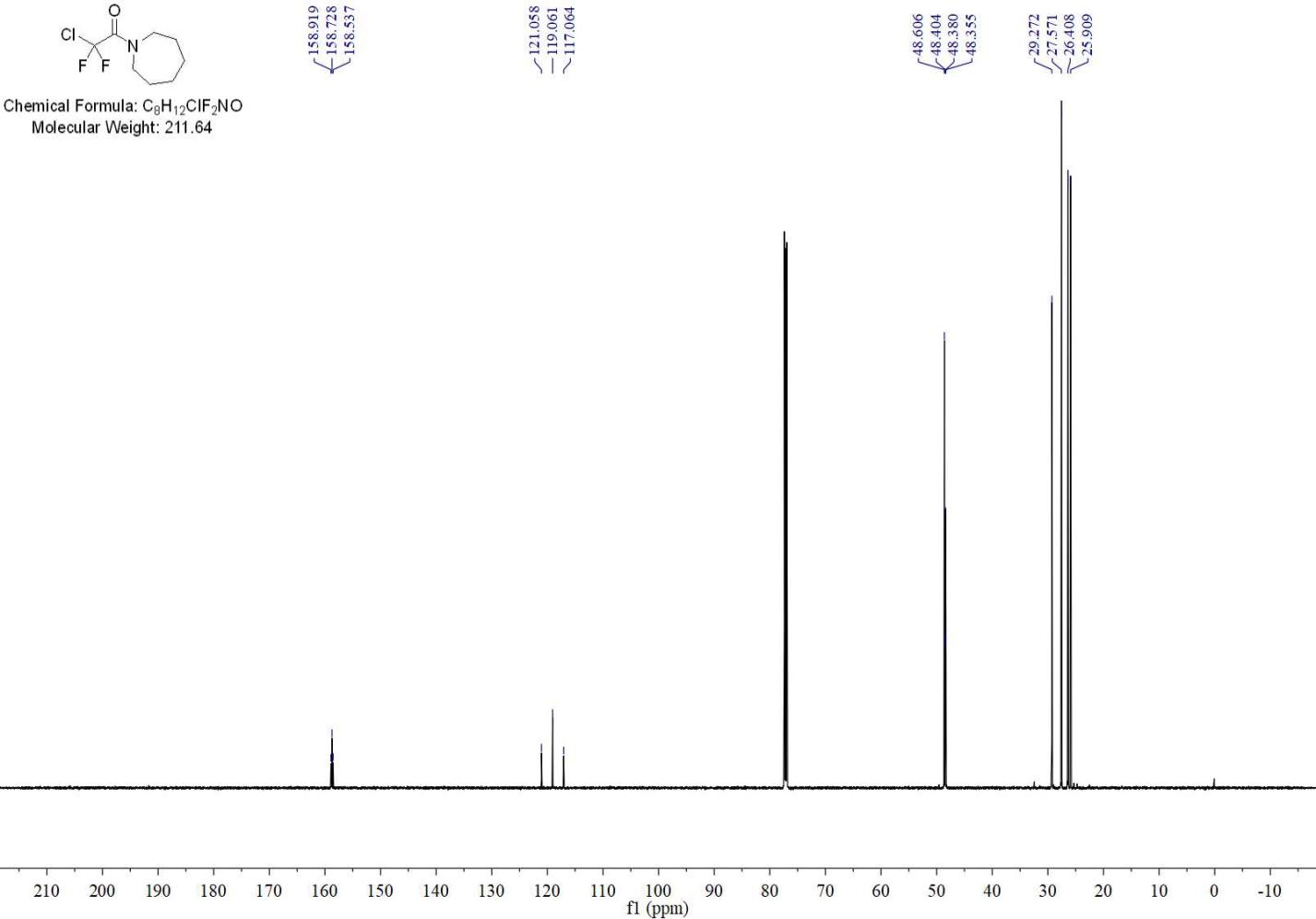


***N*-(*tert*-butyl)-2-chloro-2,2-difluoroacetamide (2i).**

**
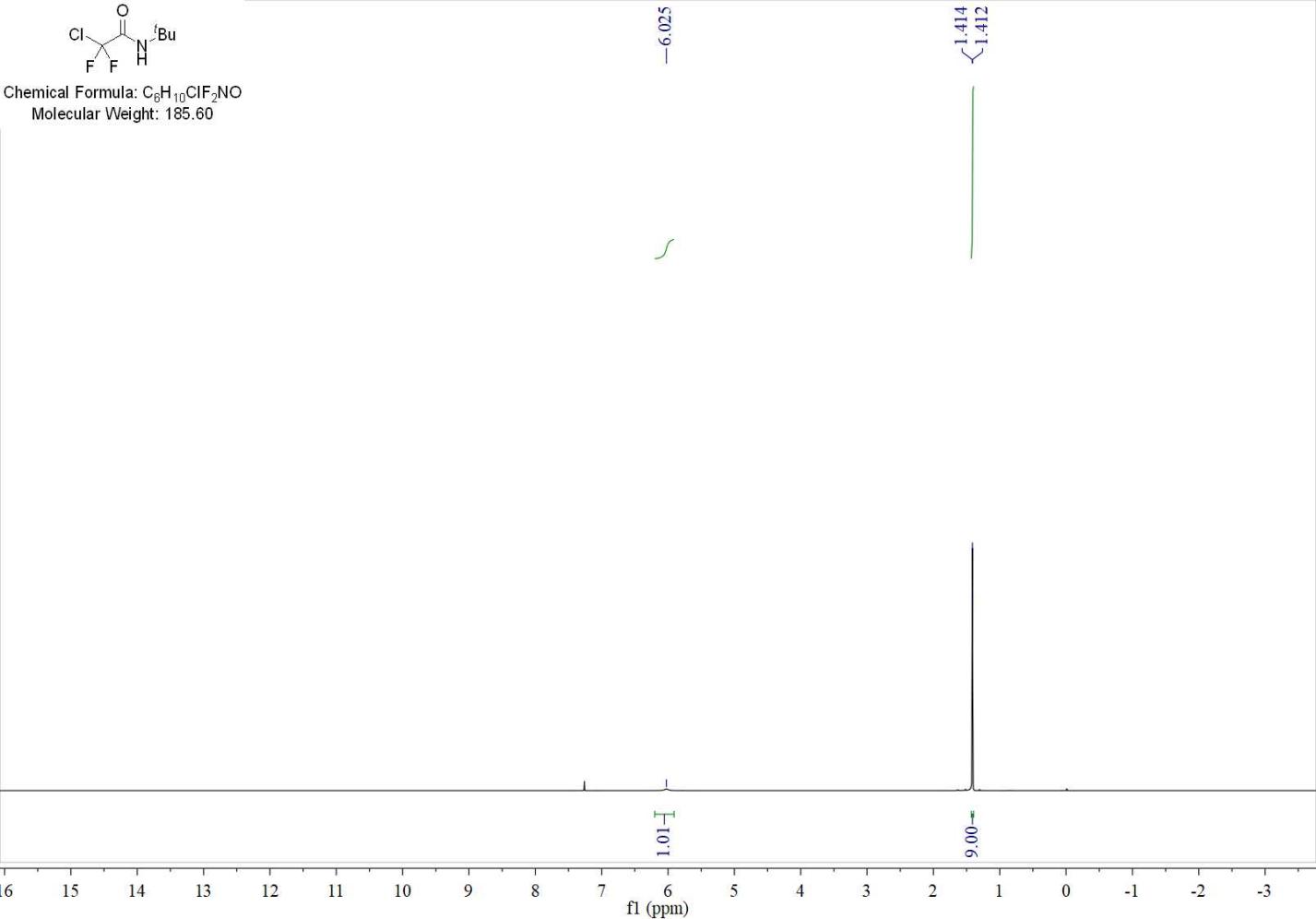
**


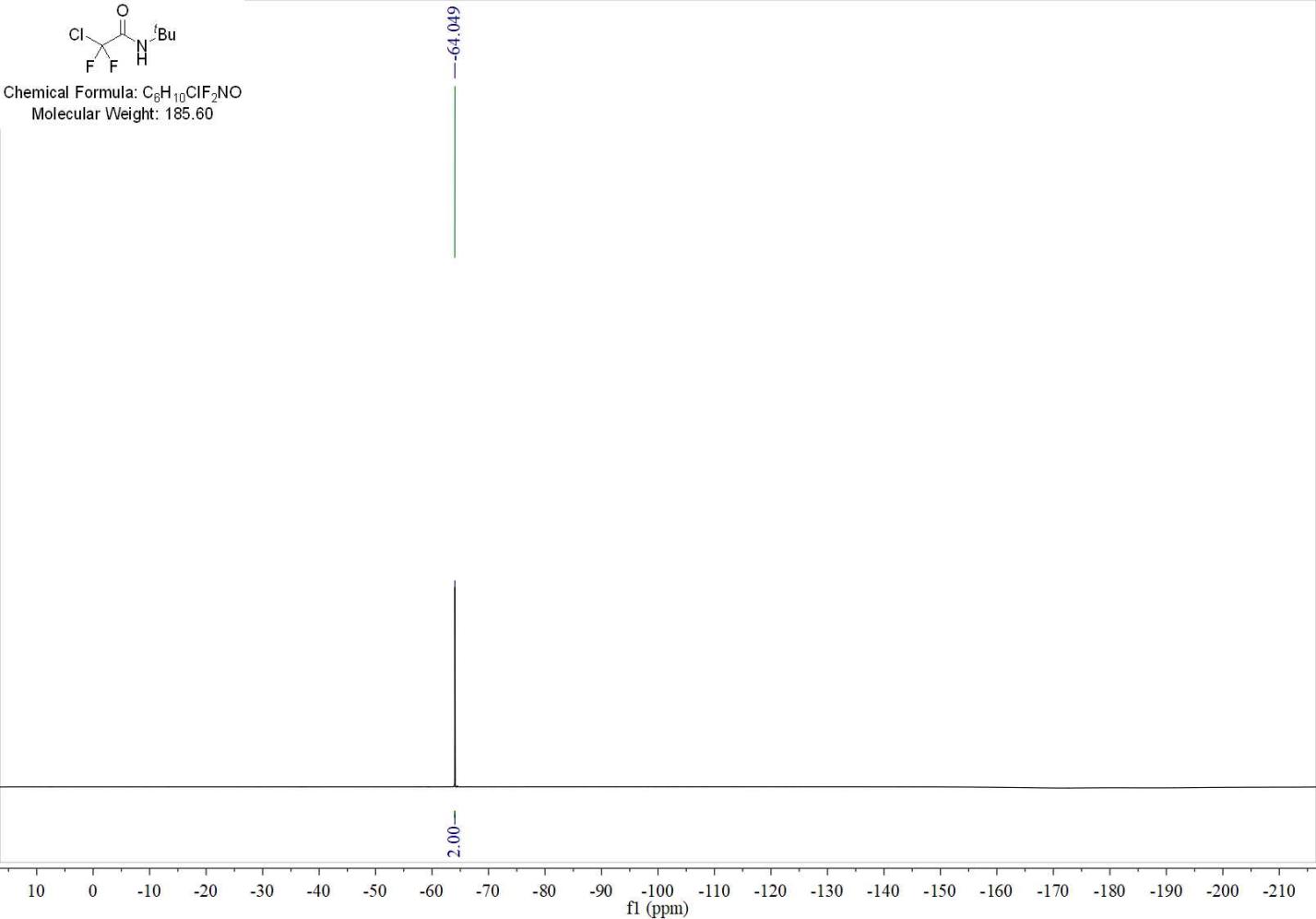


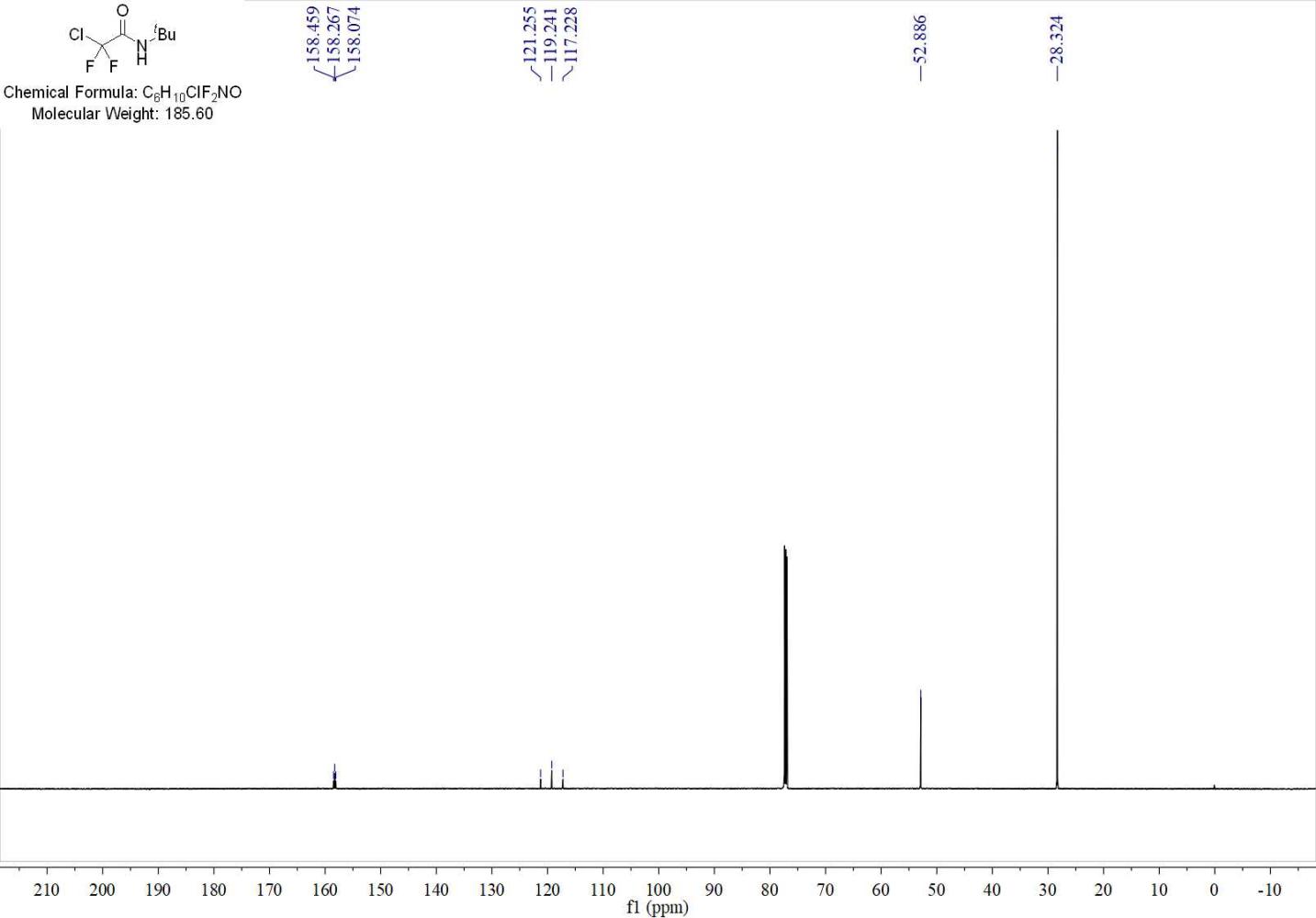


**2-chloro-*N*-cyclohexyl-2,2-difluoroacetamide (2j).**


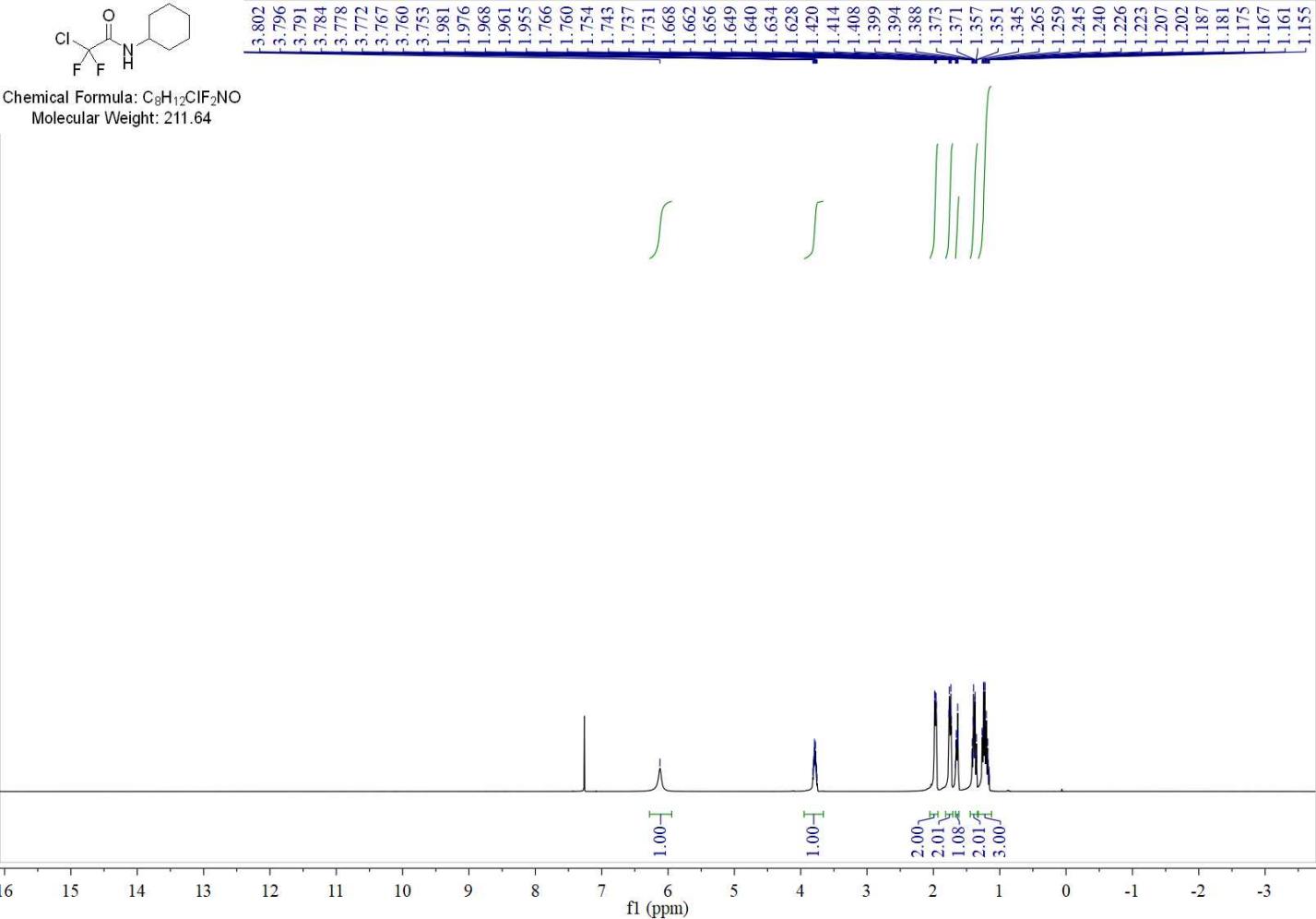


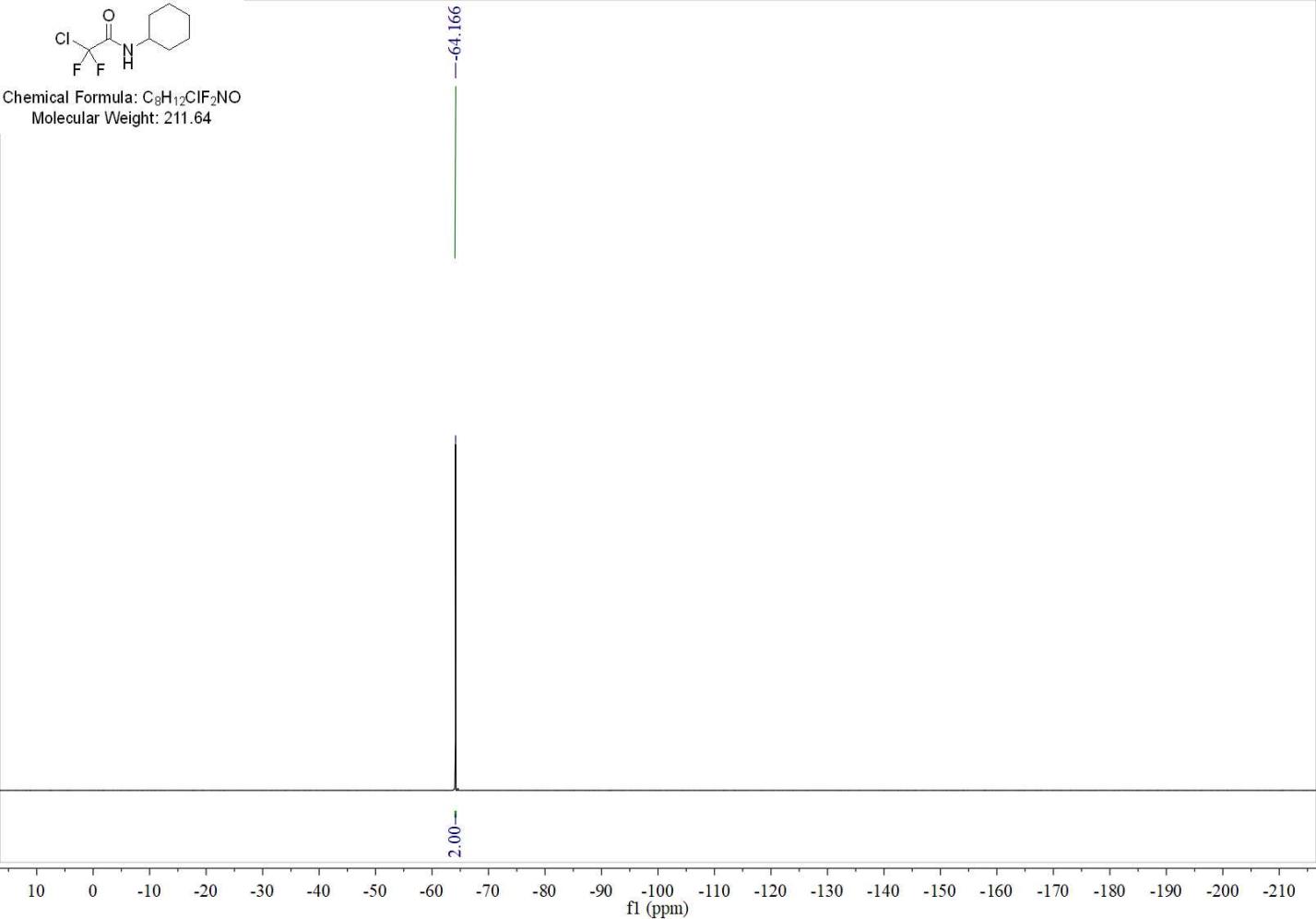

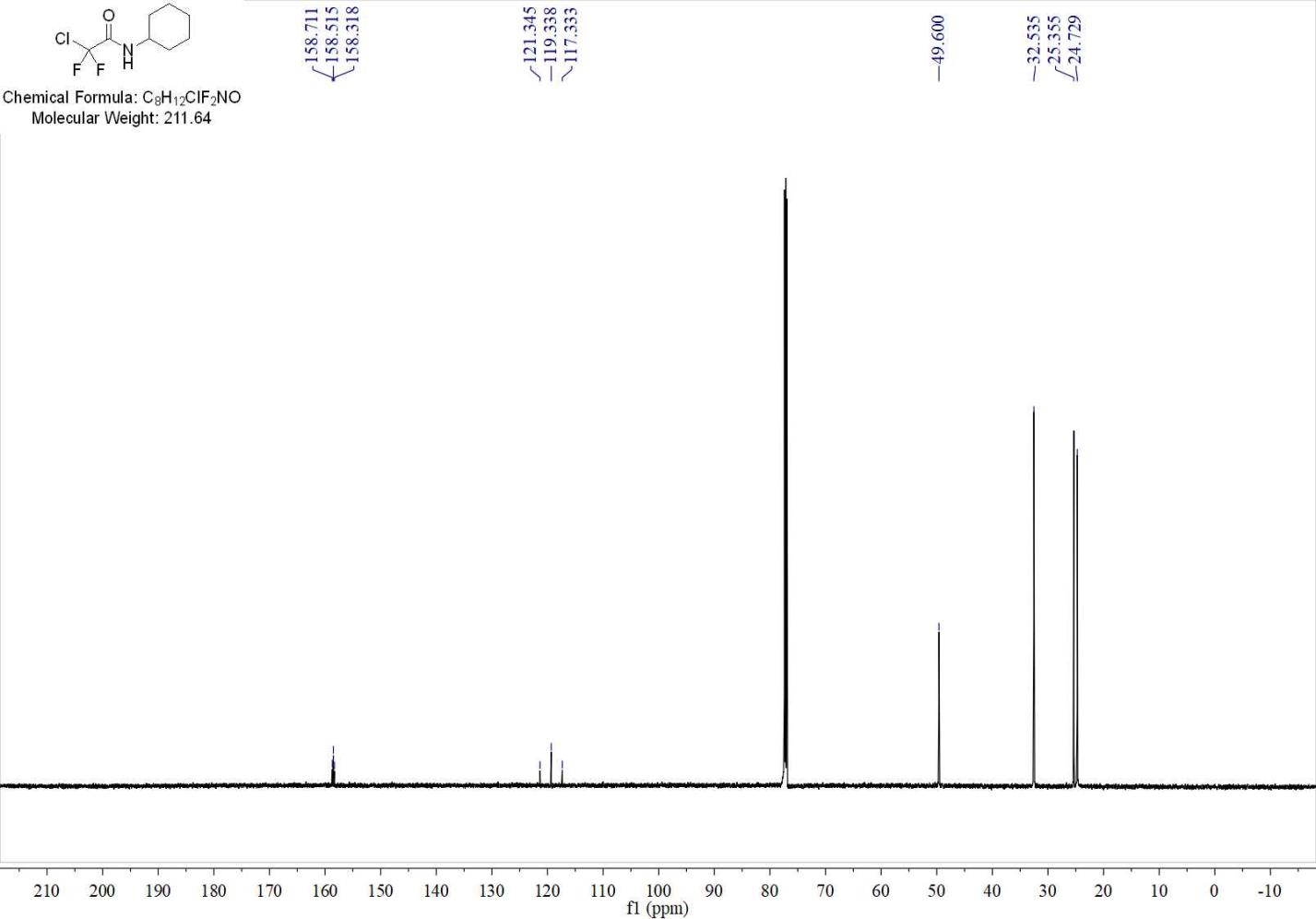


**2-chloro-*N*-(2-cyanoethyl)-2,2-difluoroacetamide (2l).**


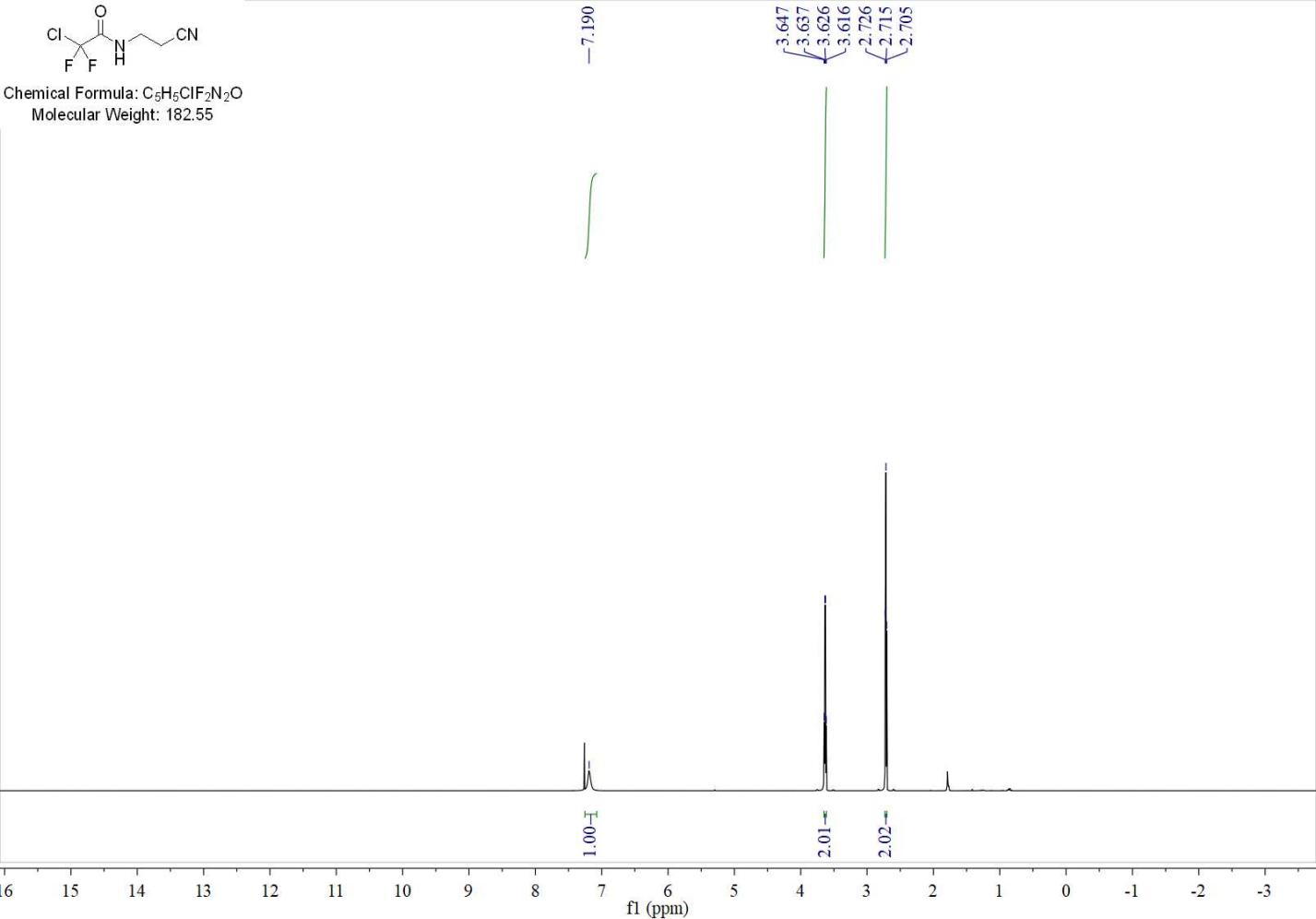

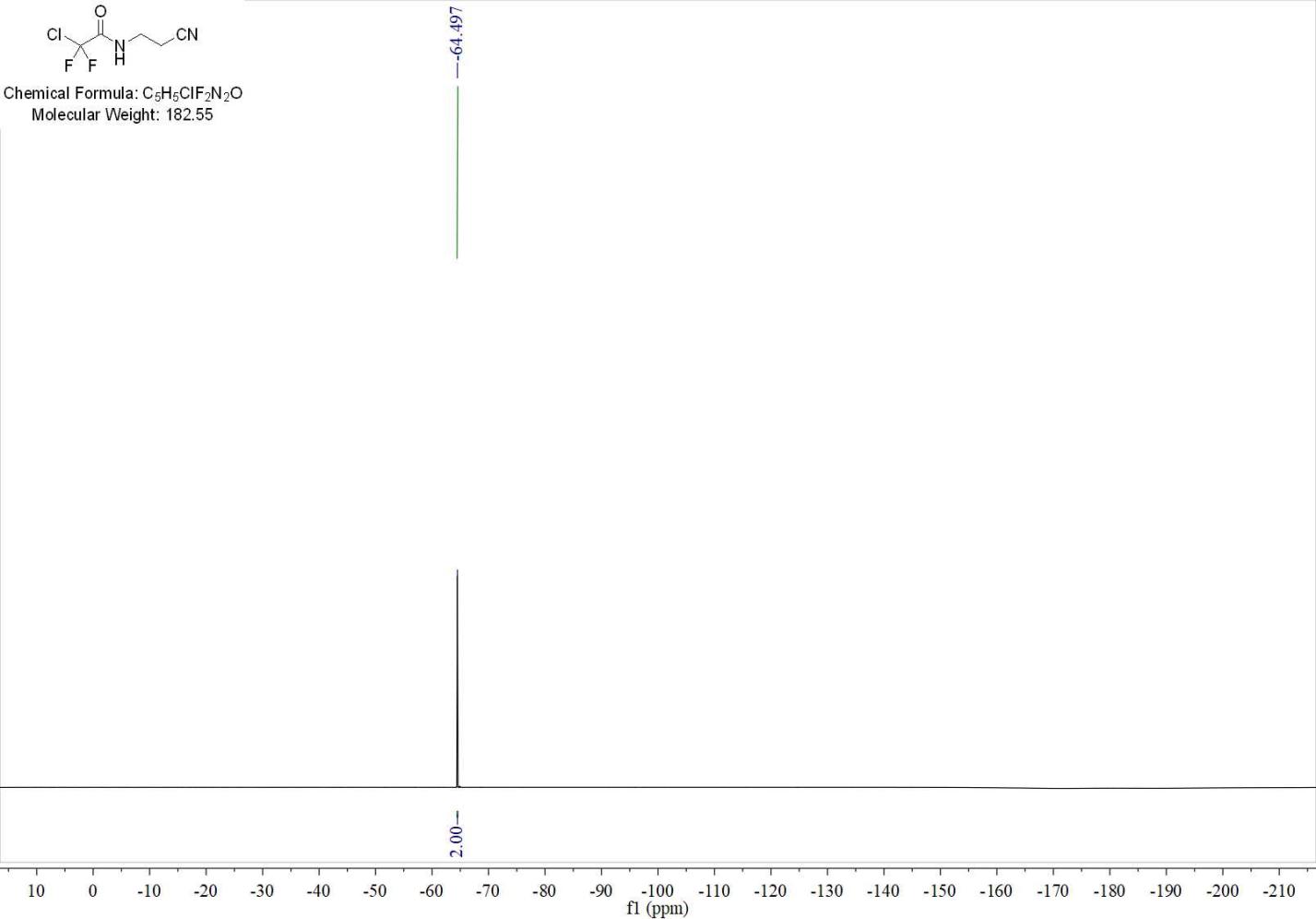


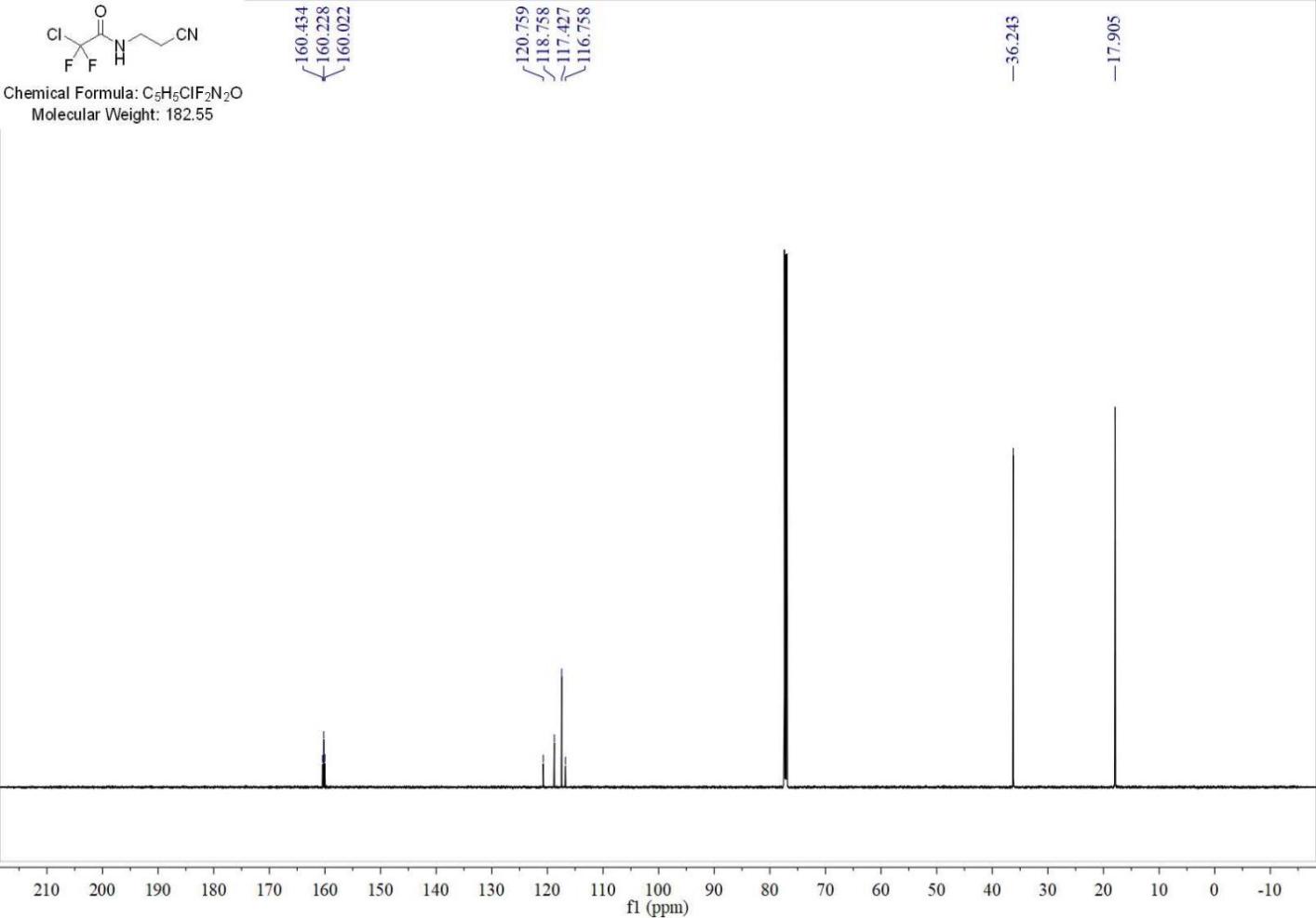


**Methyl 4-(2-chloro-2,2-difluoroacetamido) butanoate (2m).**


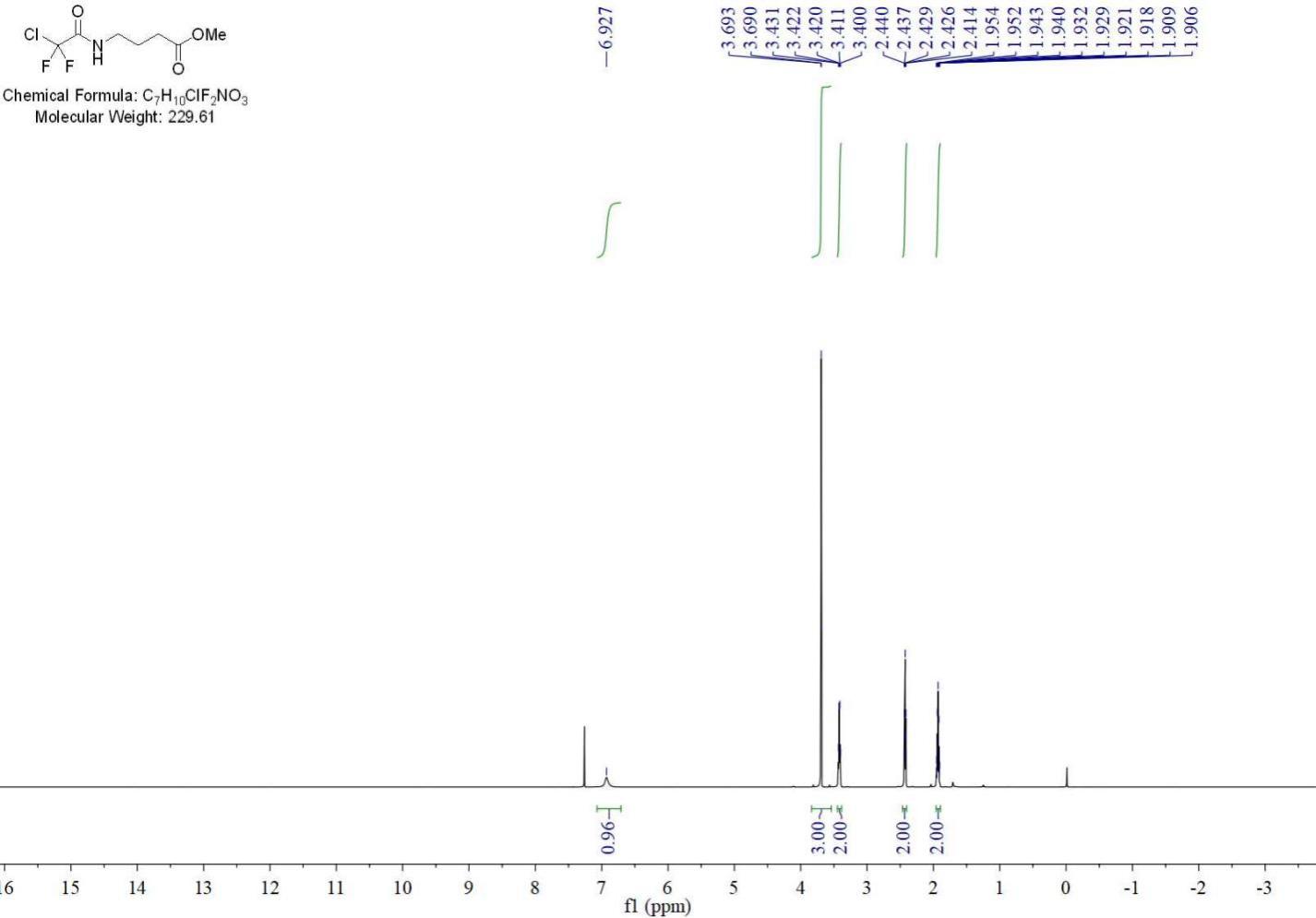

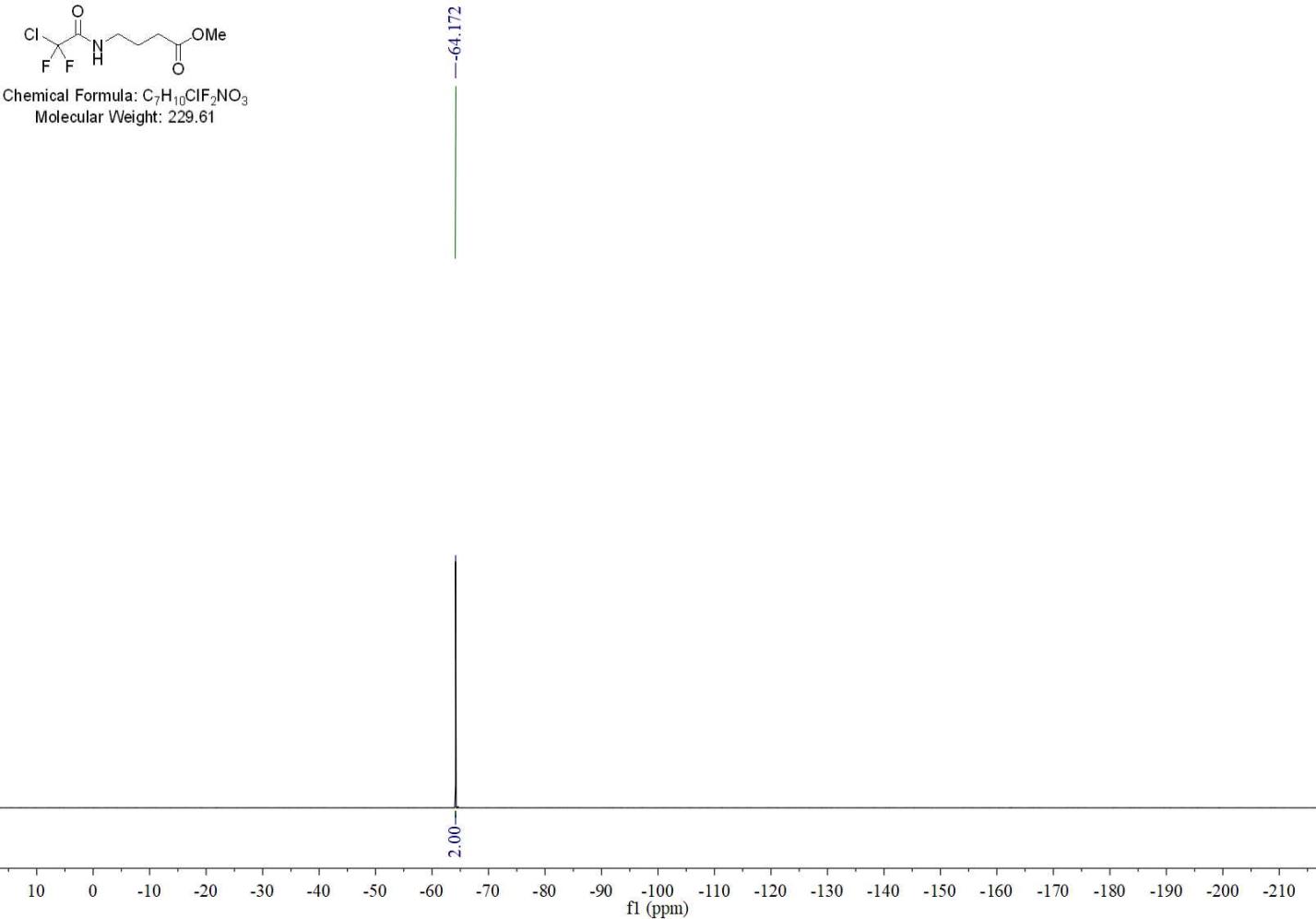


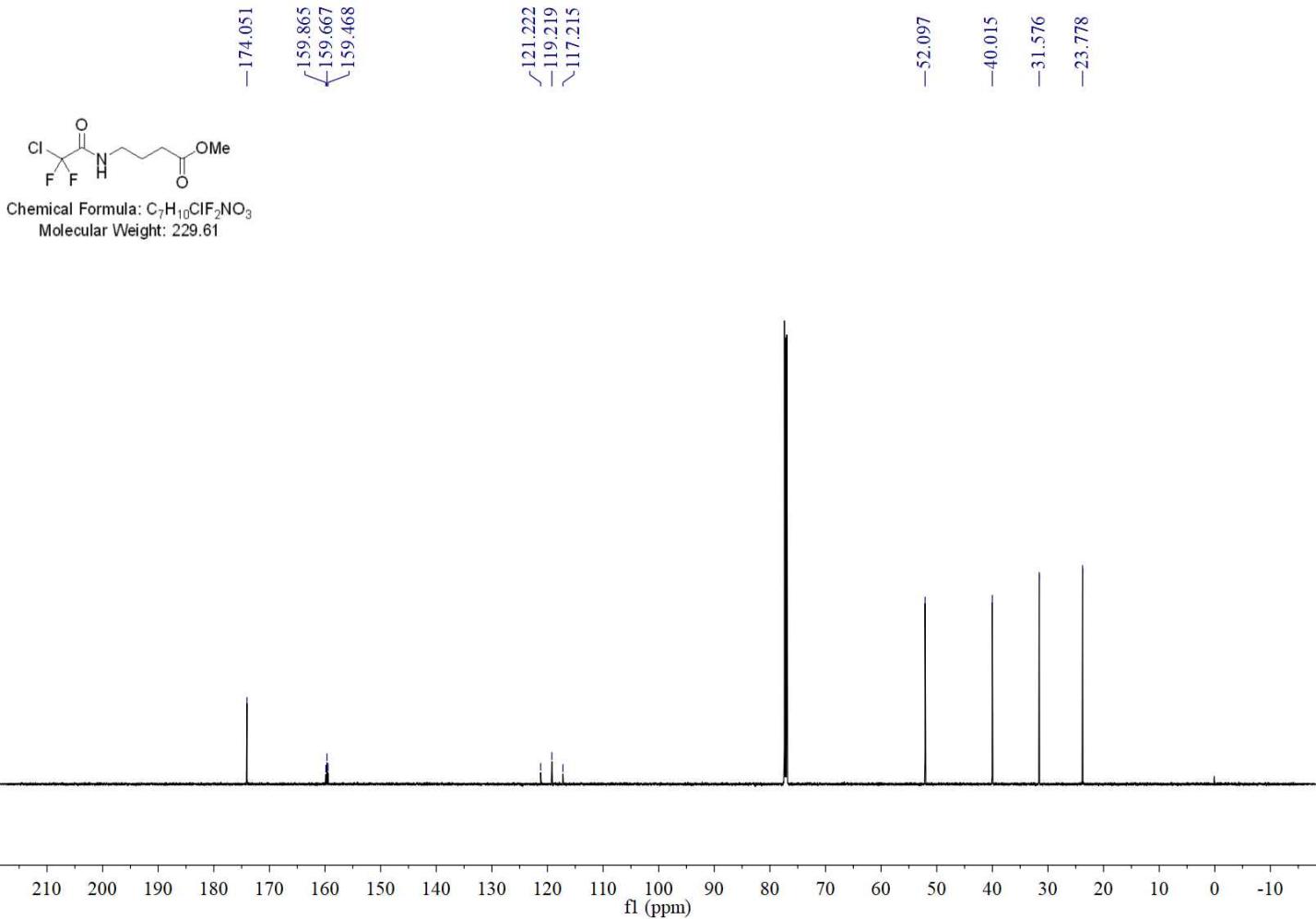


***Tert*-butyl (4-(2-chloro-2,2-difluoroacetamido)butyl)carbamate (2n).**


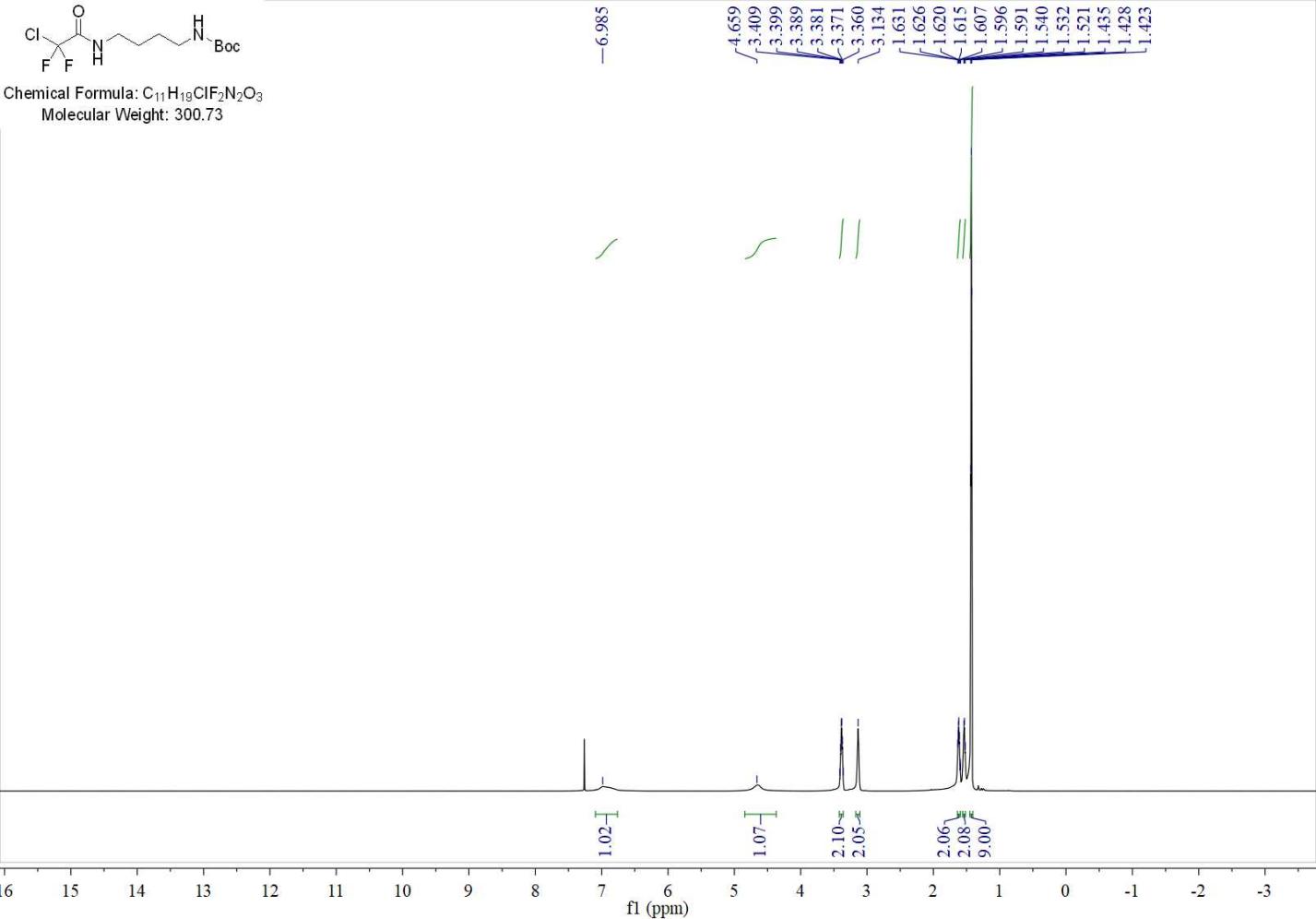

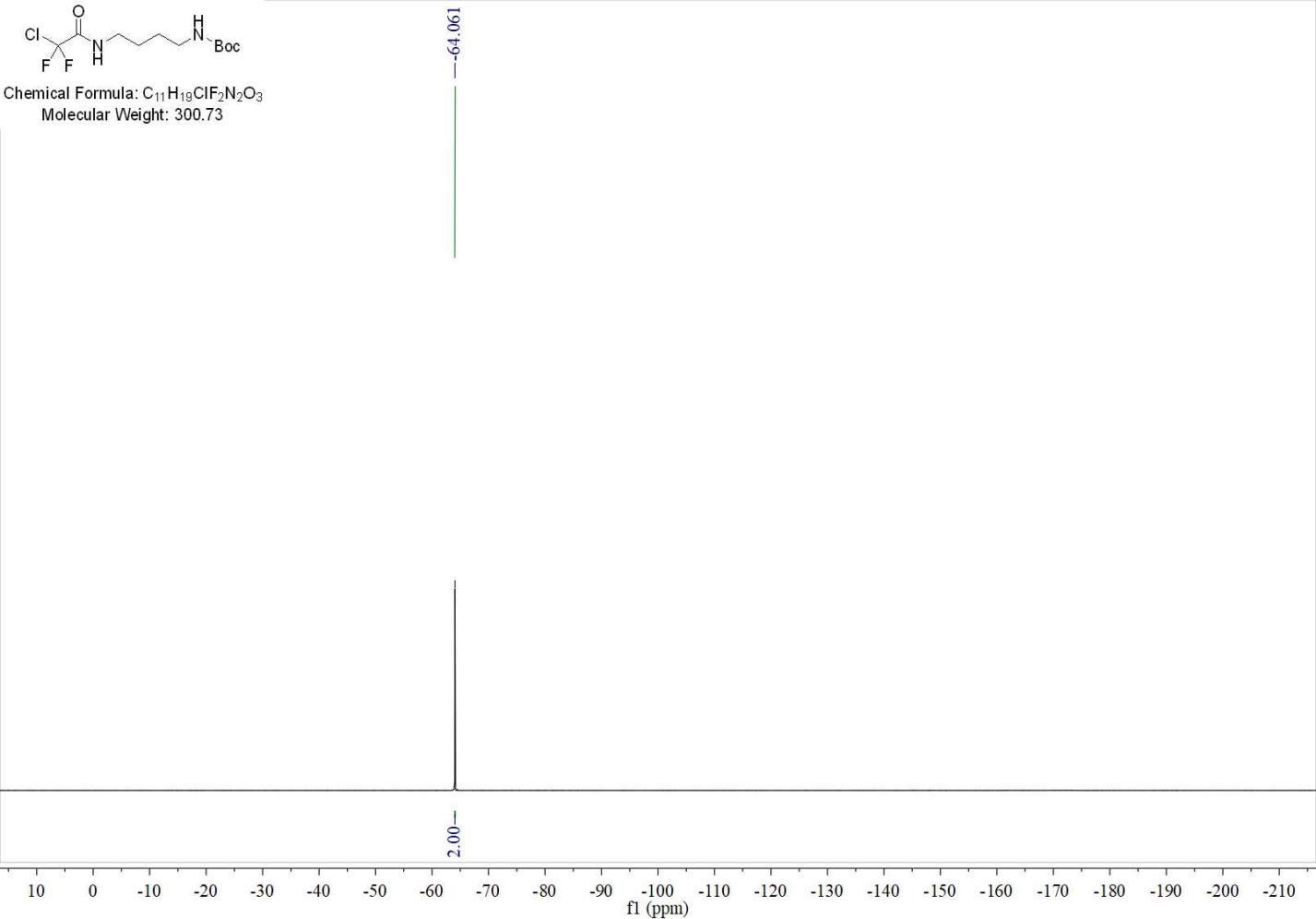

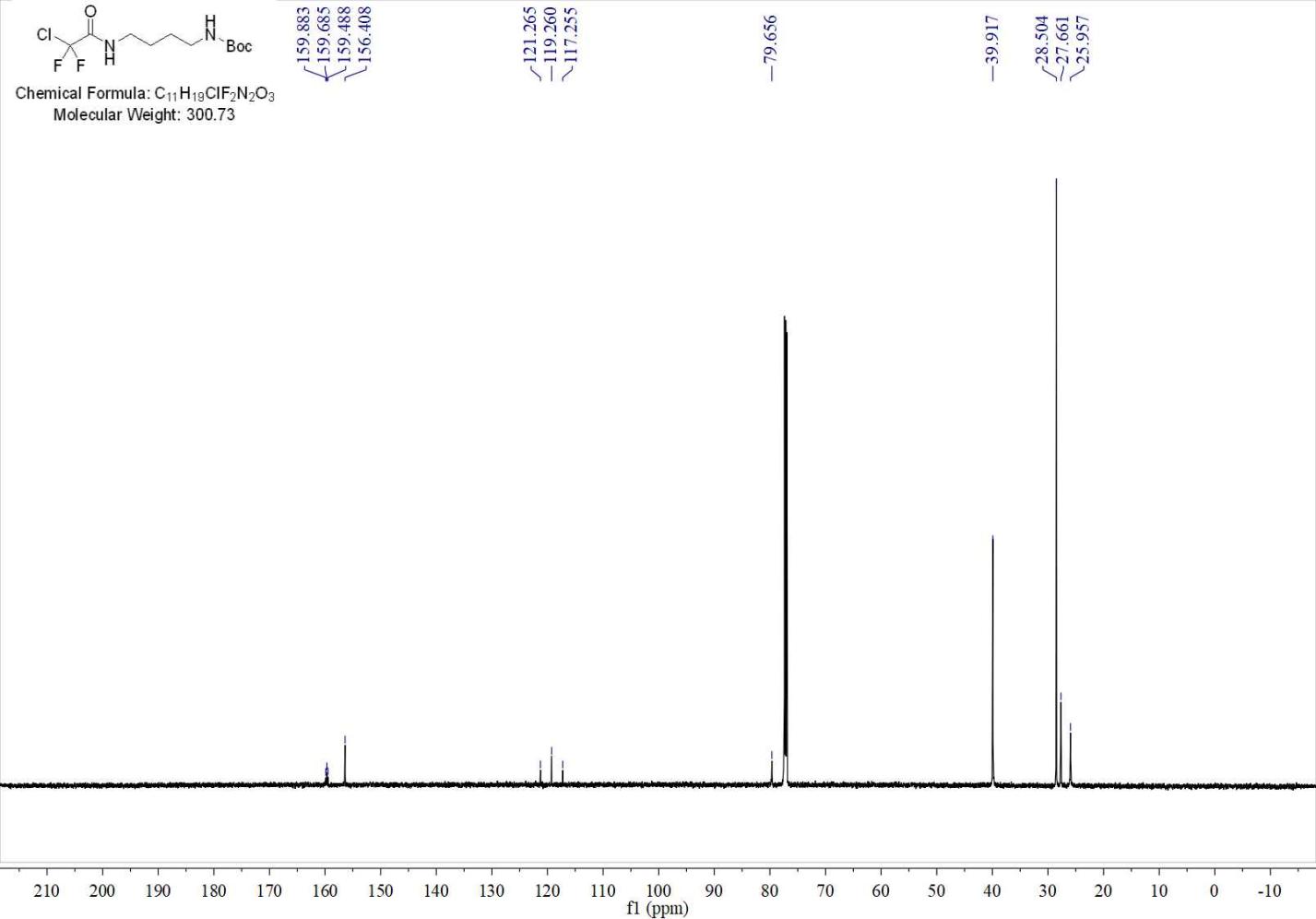


**Methyl *N*^6^-(tert-butoxycarbonyl)-*N*^2^-(2-chloro-2,2-difluoroacetyl)-L-lysinate (2o).**


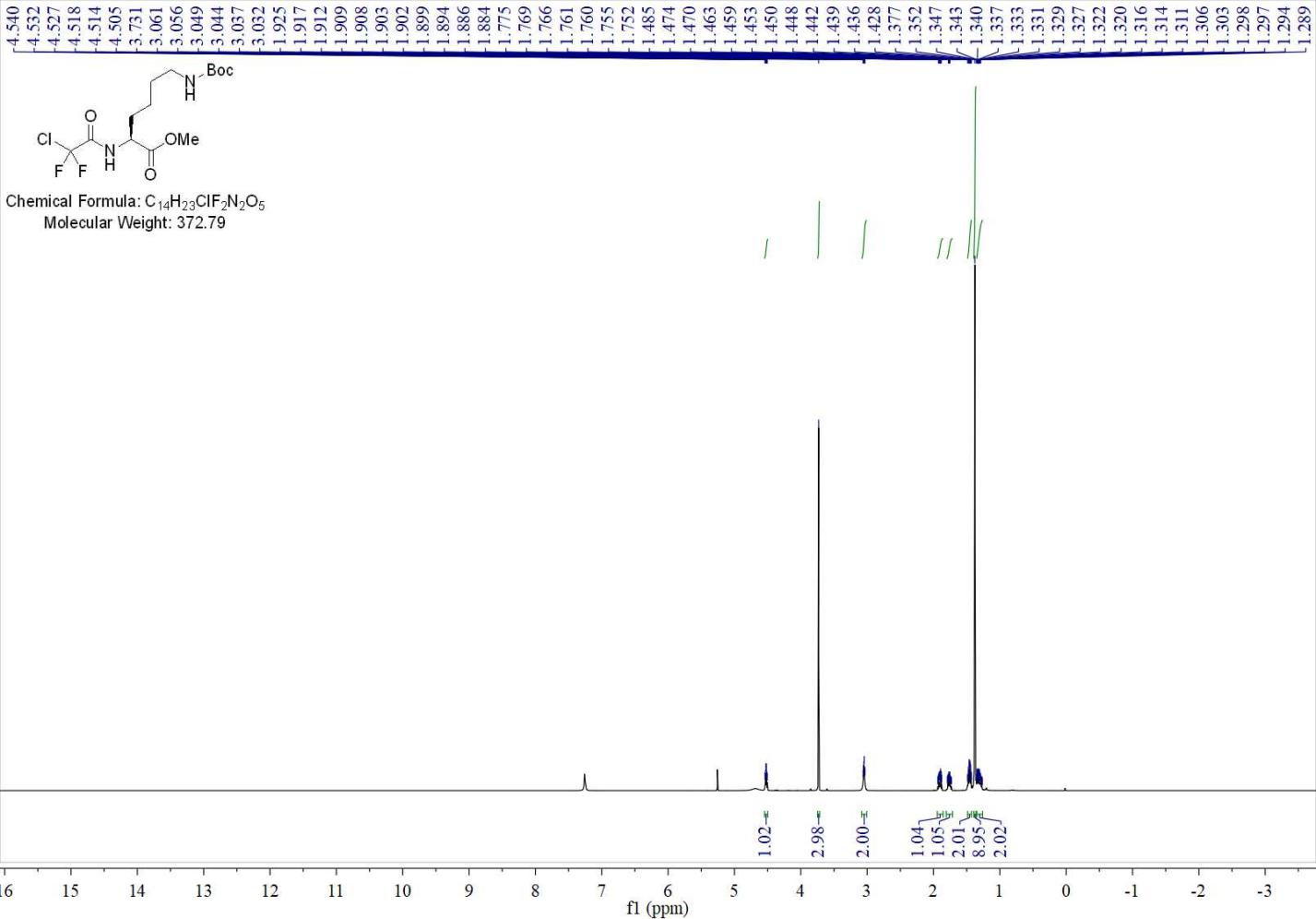

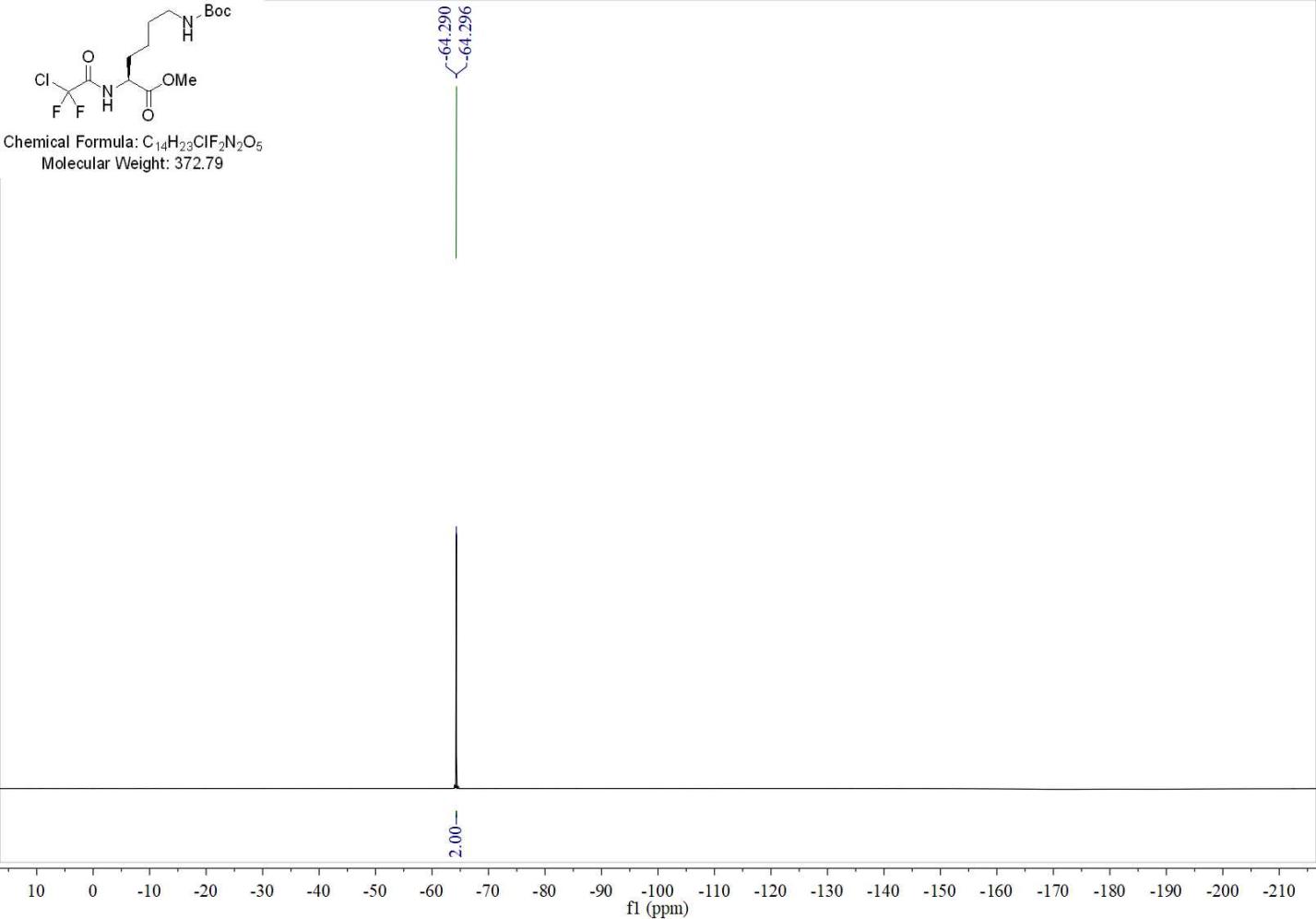

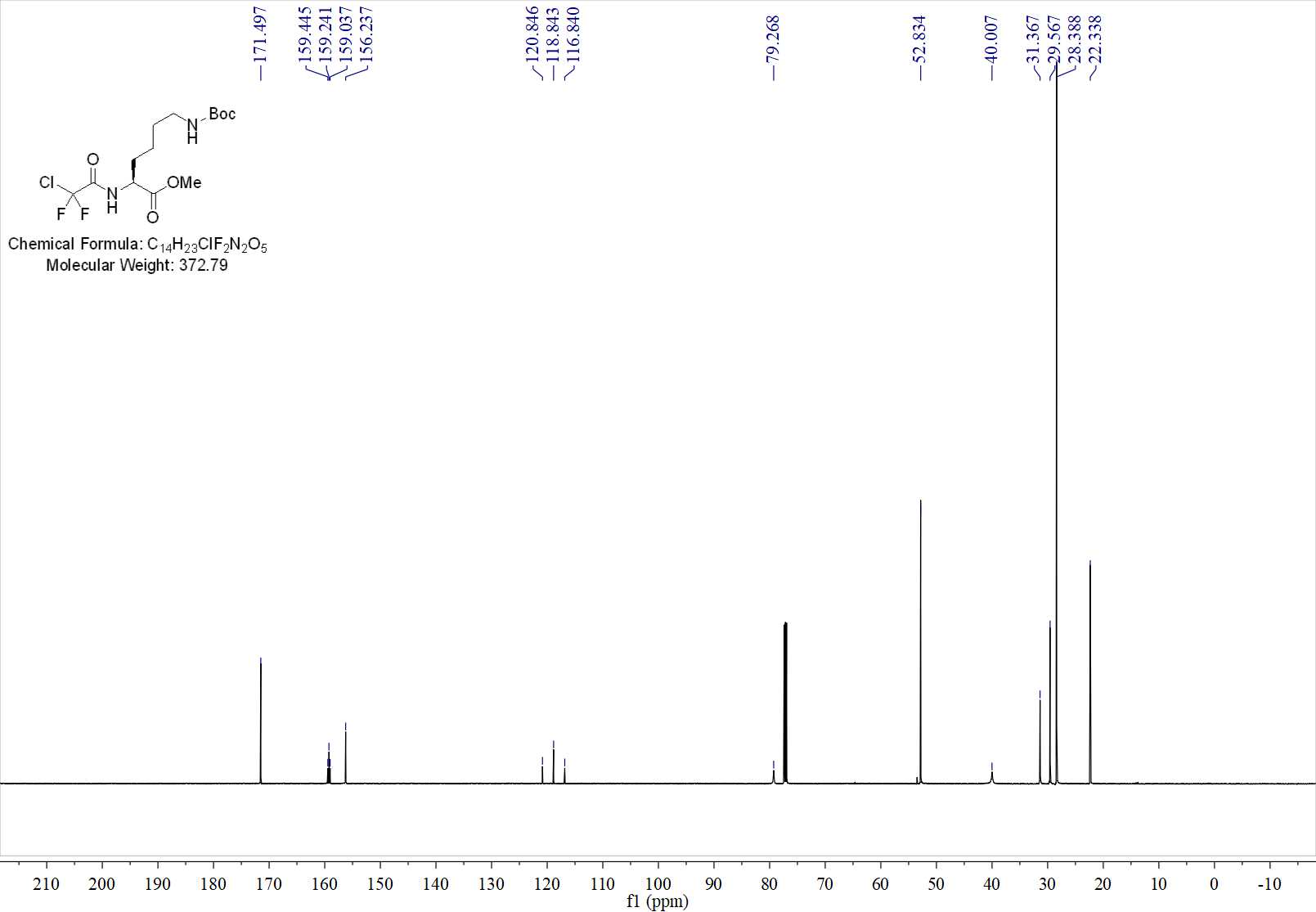


**Methyl (2-chloro-2,2-difluoroacetyl)-L-valinate (2p).**


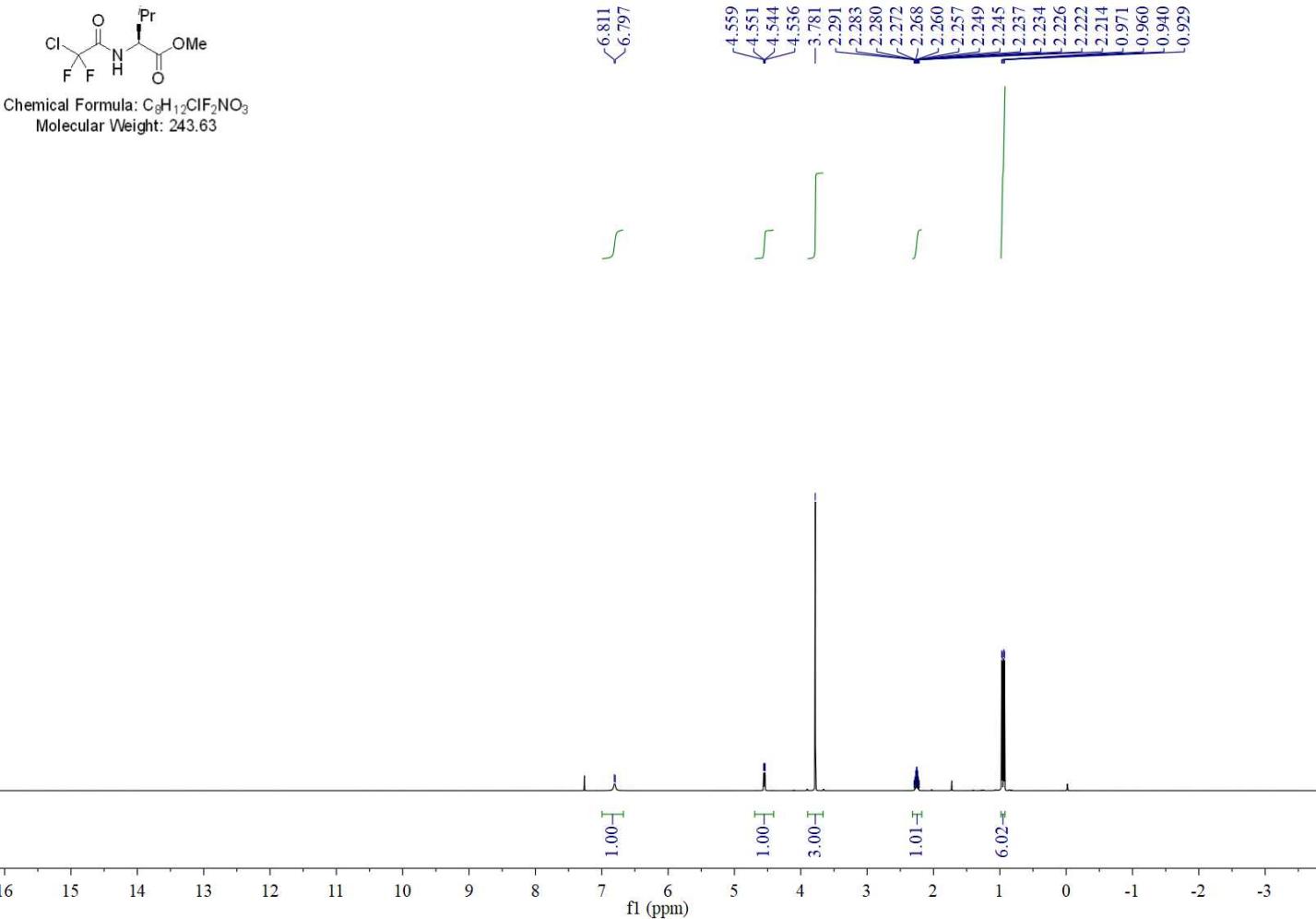

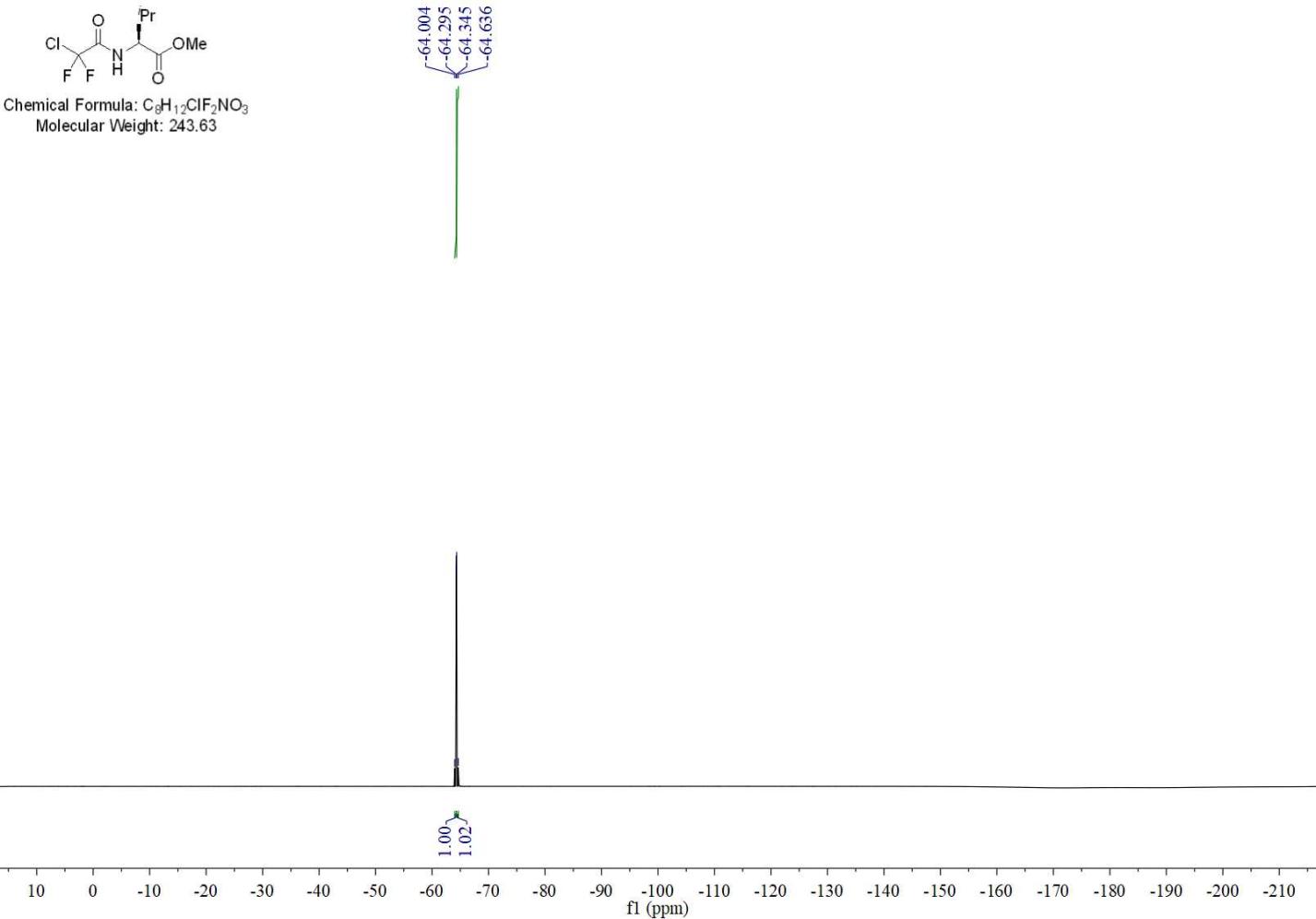


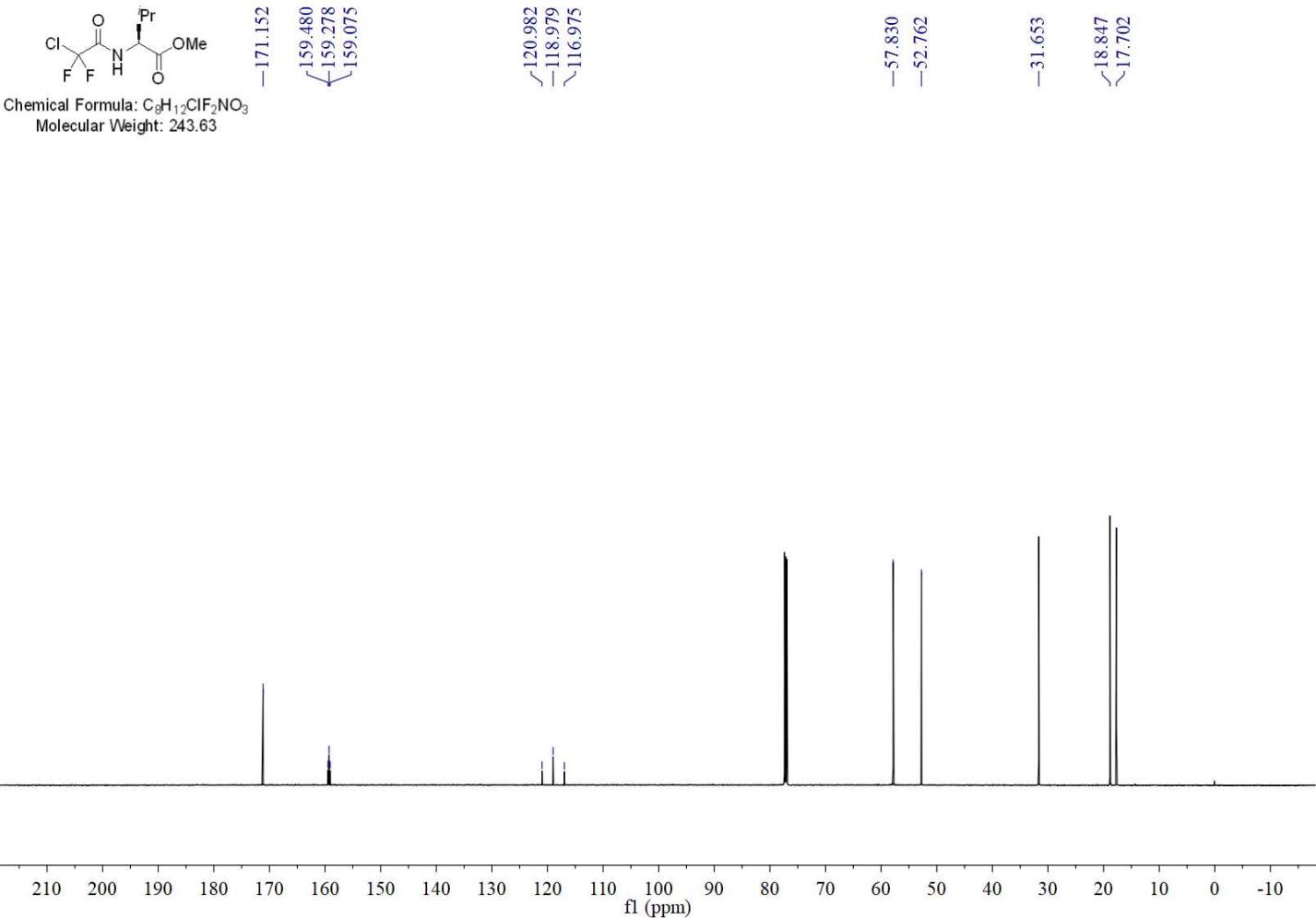


**Methyl (2-chloro-2,2-difluoroacetyl)-L-methioninate (2q).**


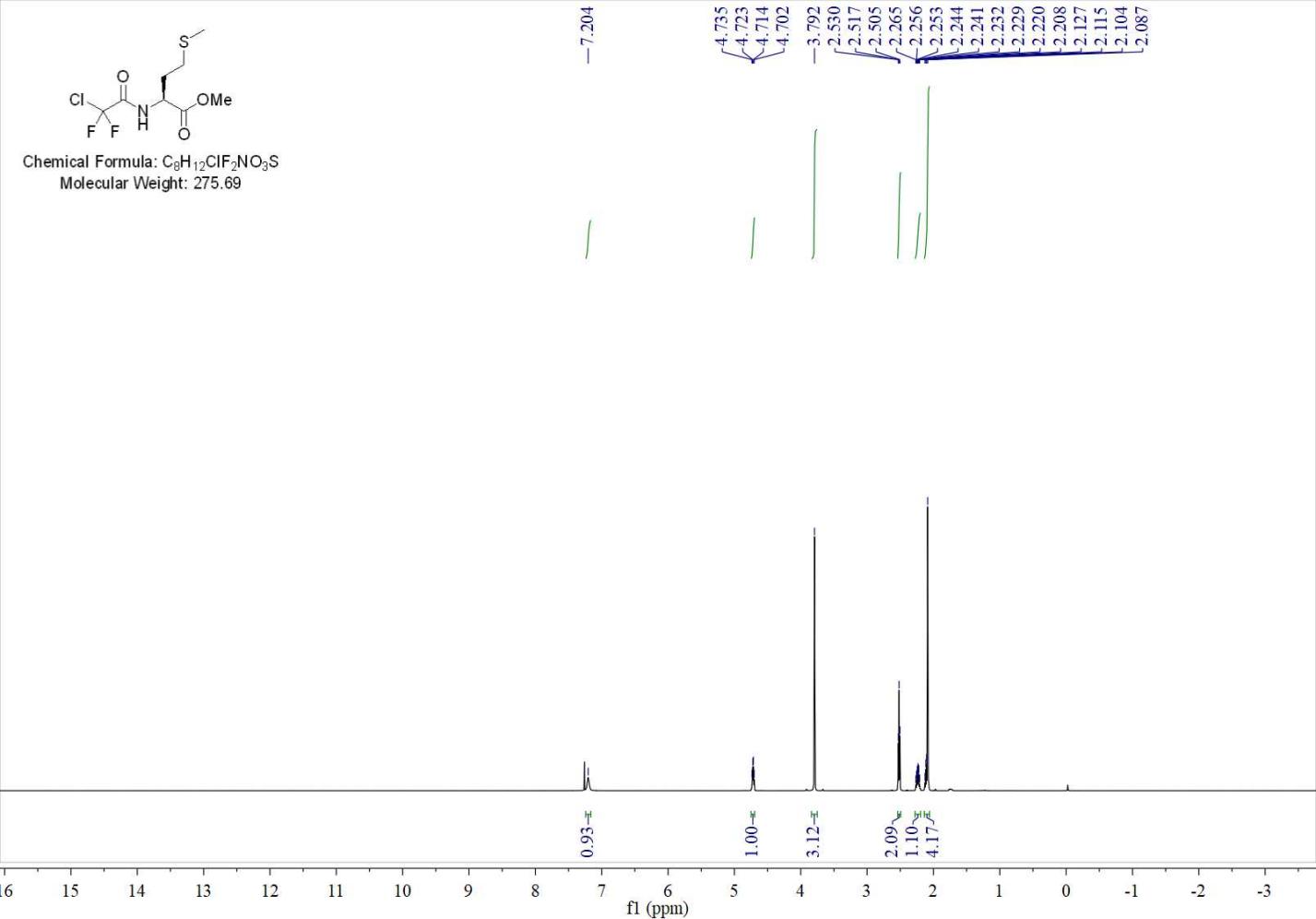

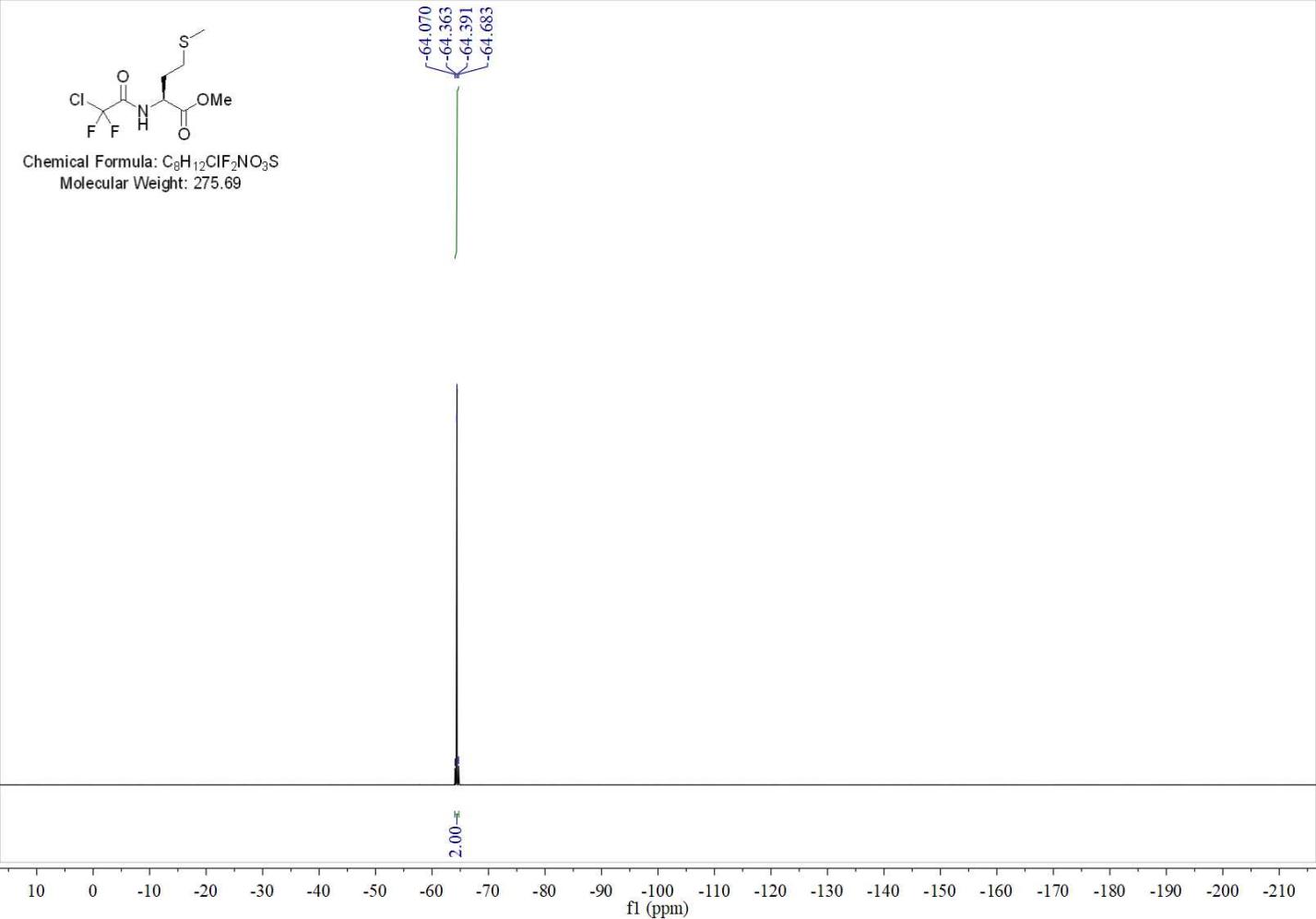

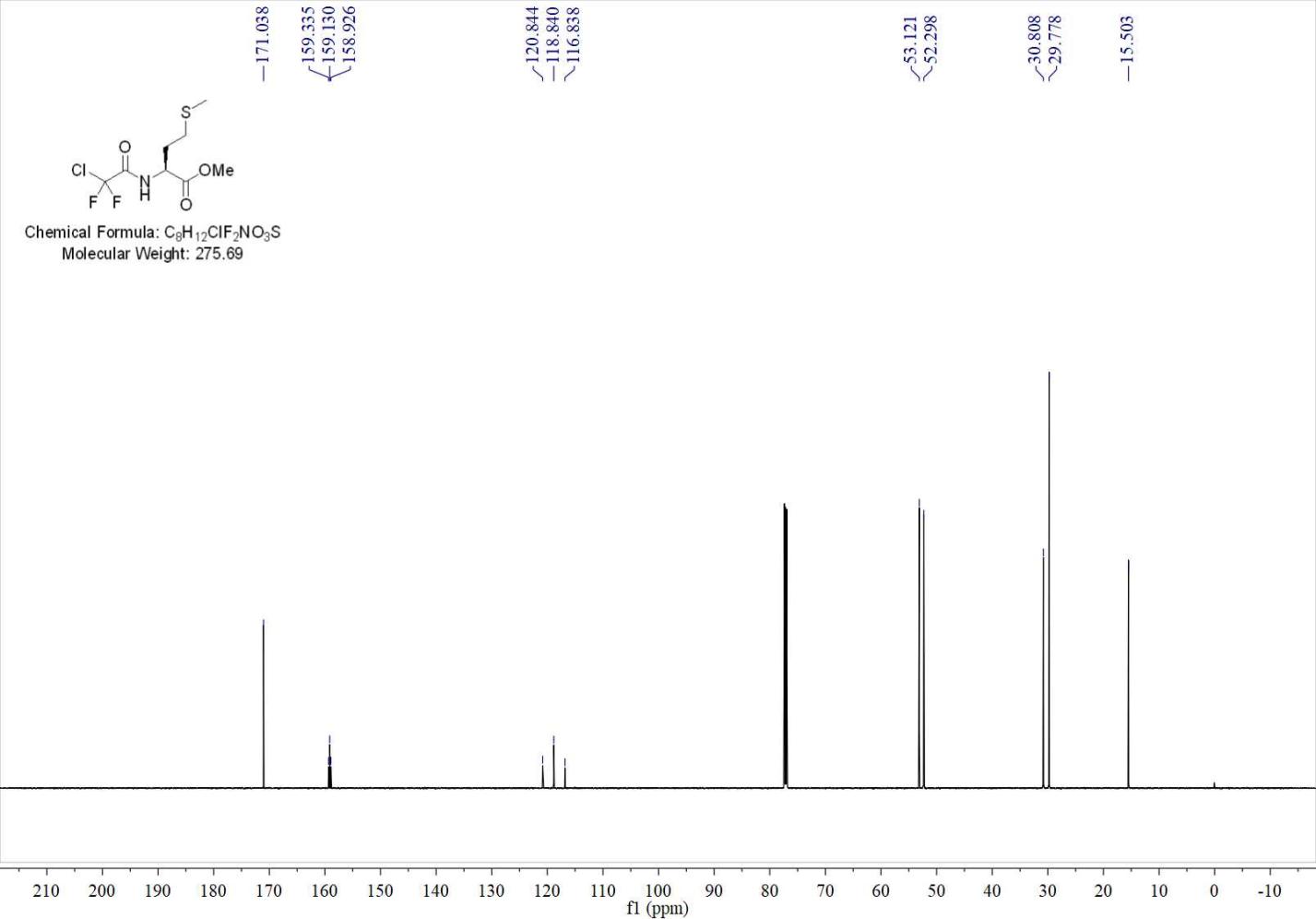


**Methyl (2-chloro-2,2-difluoroacetyl)-L-tryptophanate (2r).**


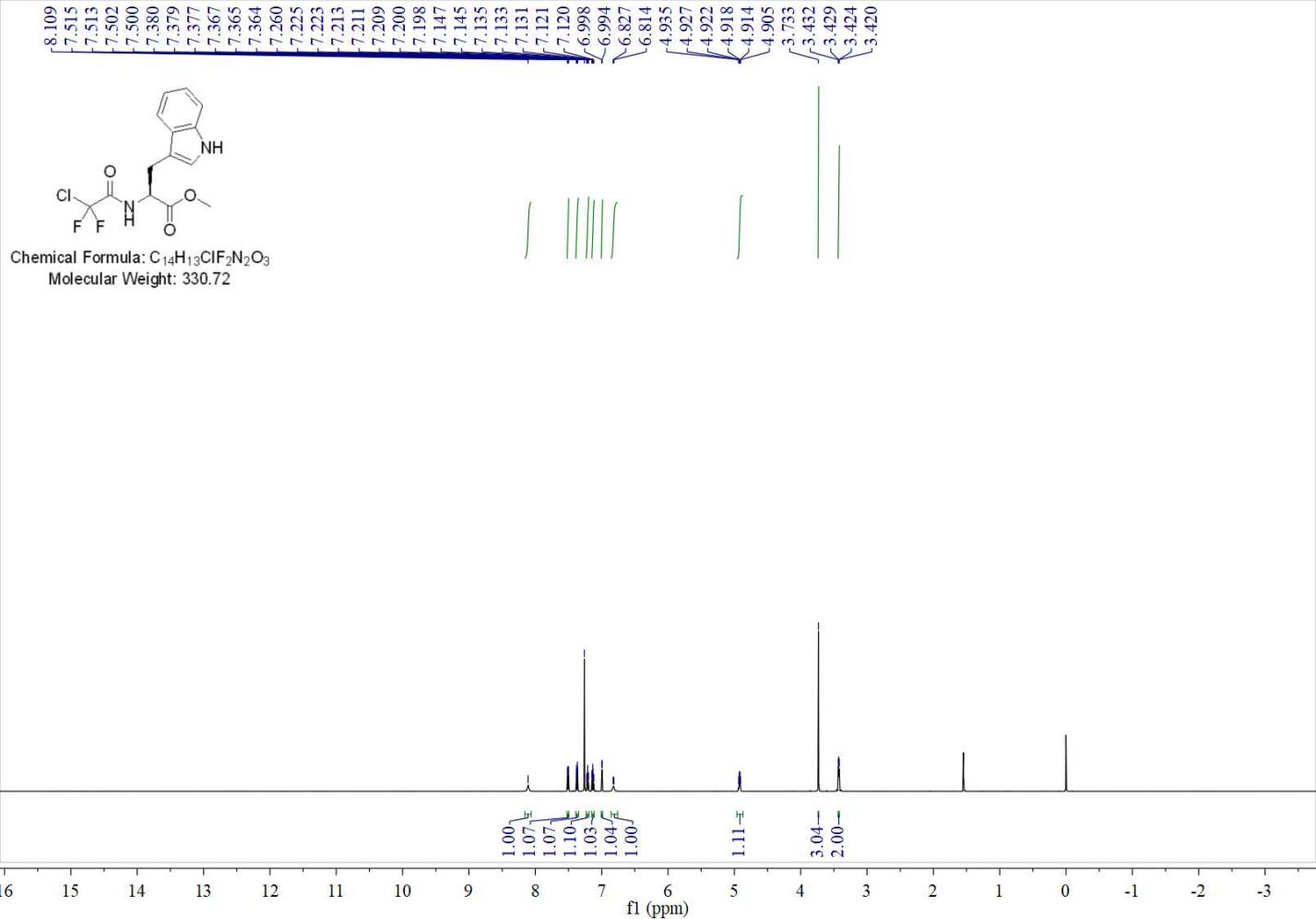

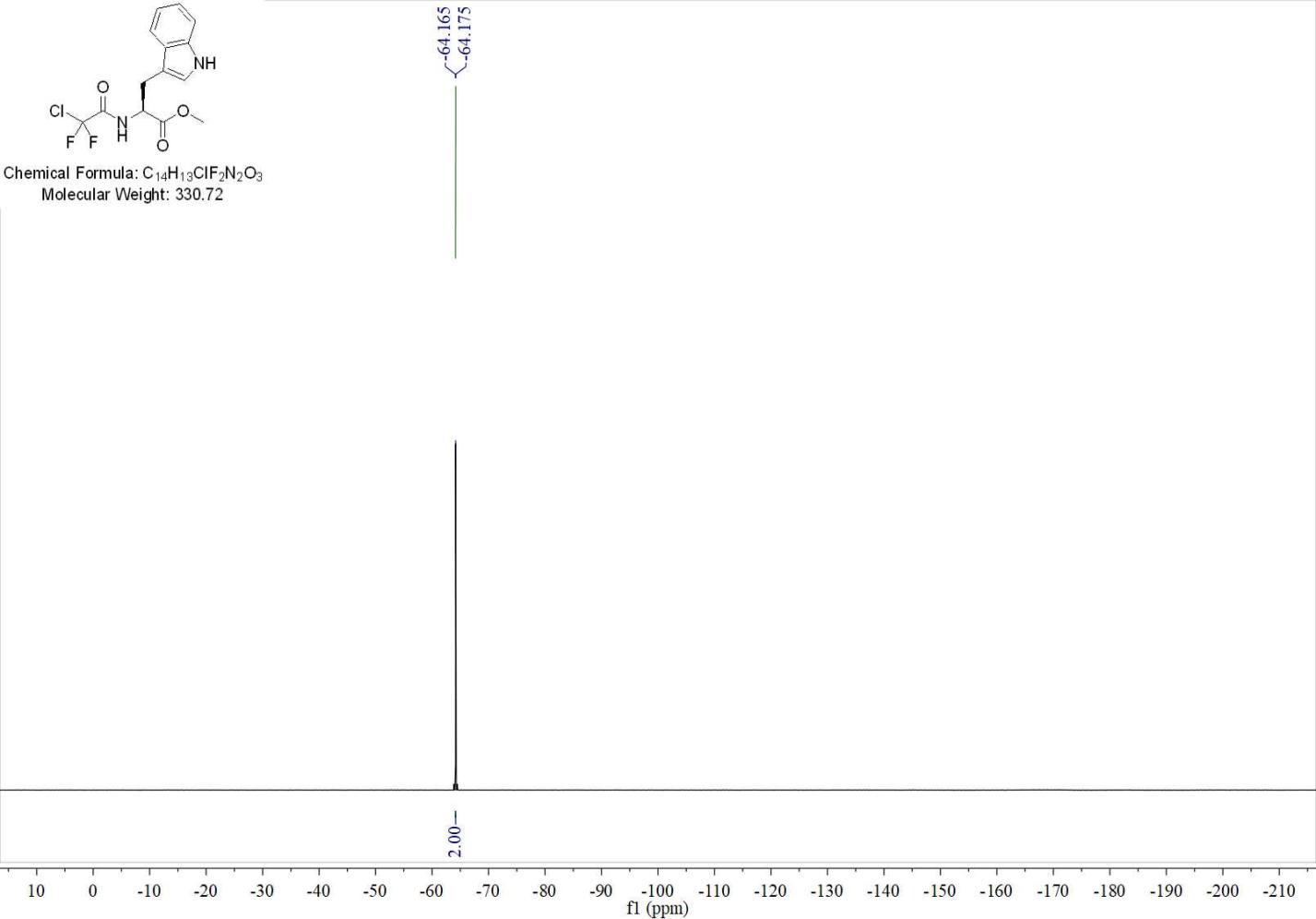

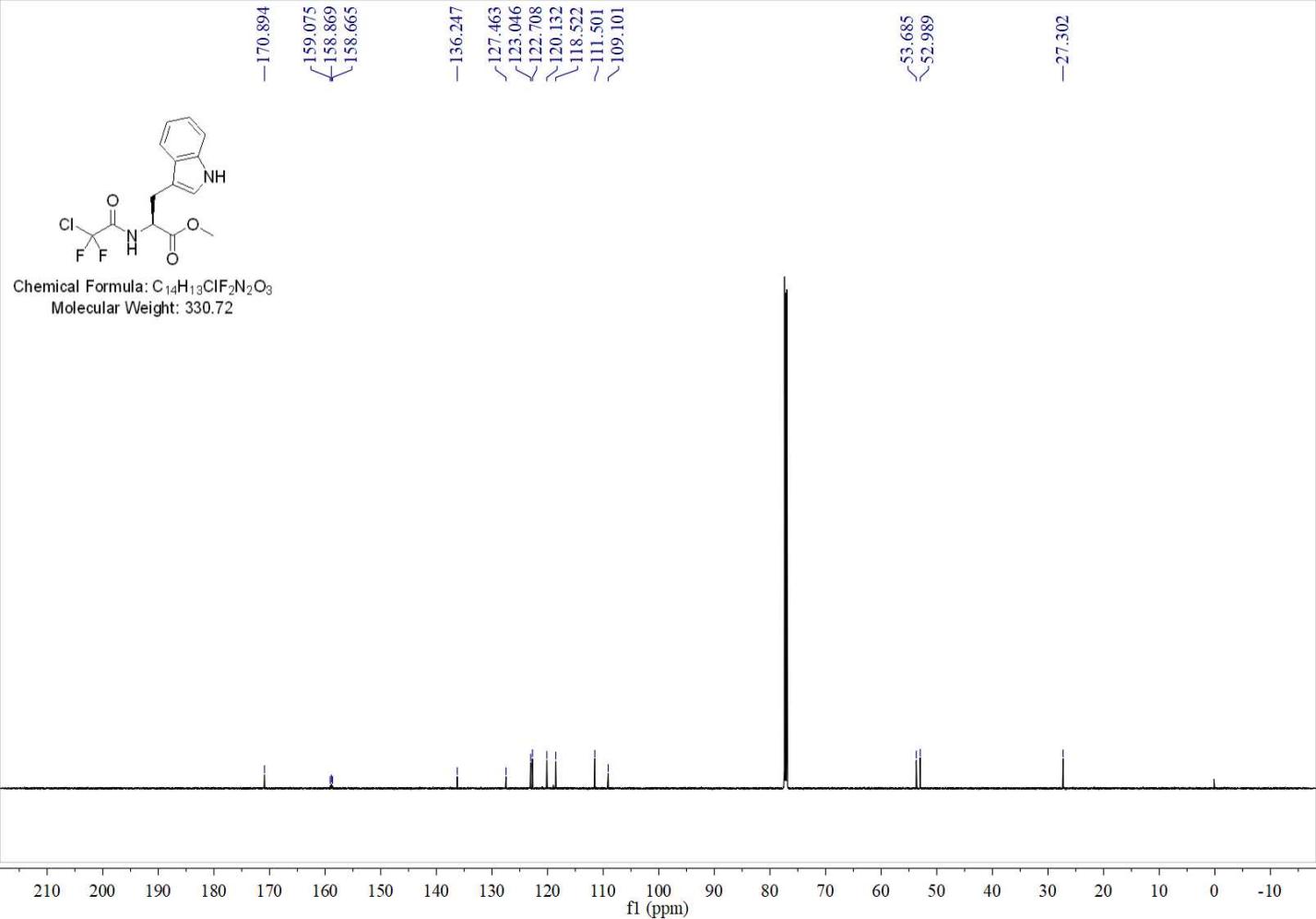


**Methyl 4-(2-chloro-2,2-difluoroacetamido)-3-(4-chlorophenyl) butanoate (2u).**


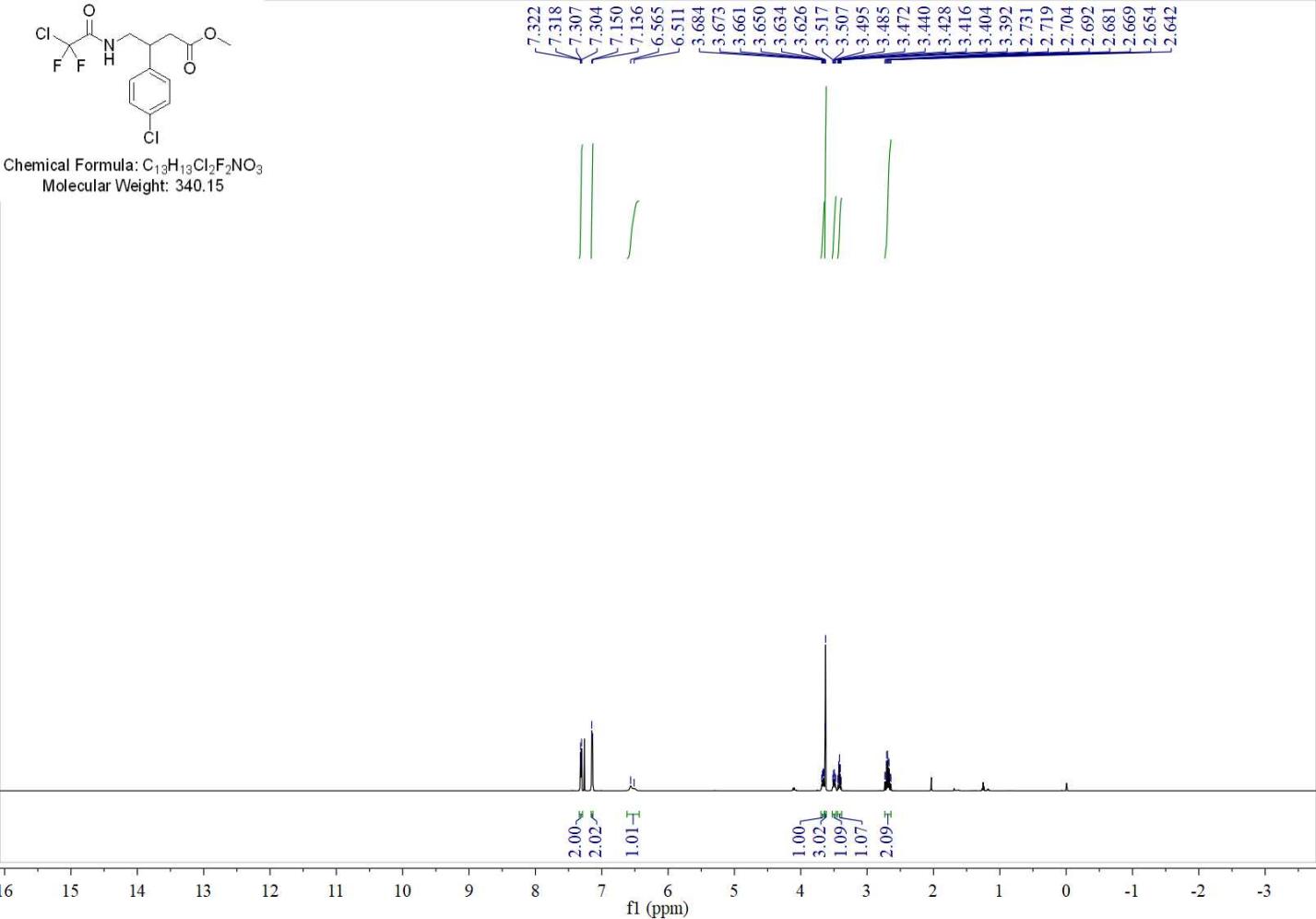

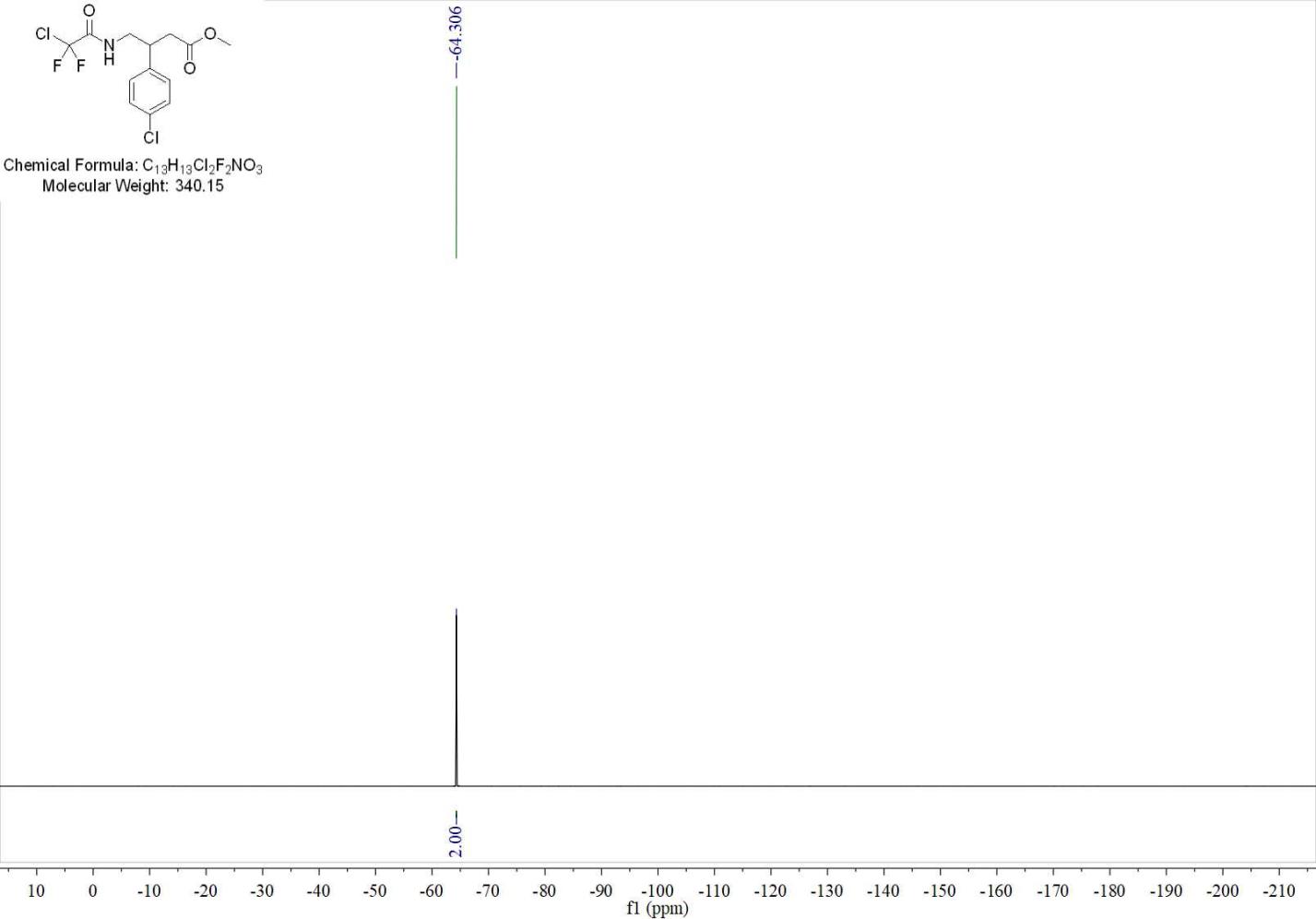

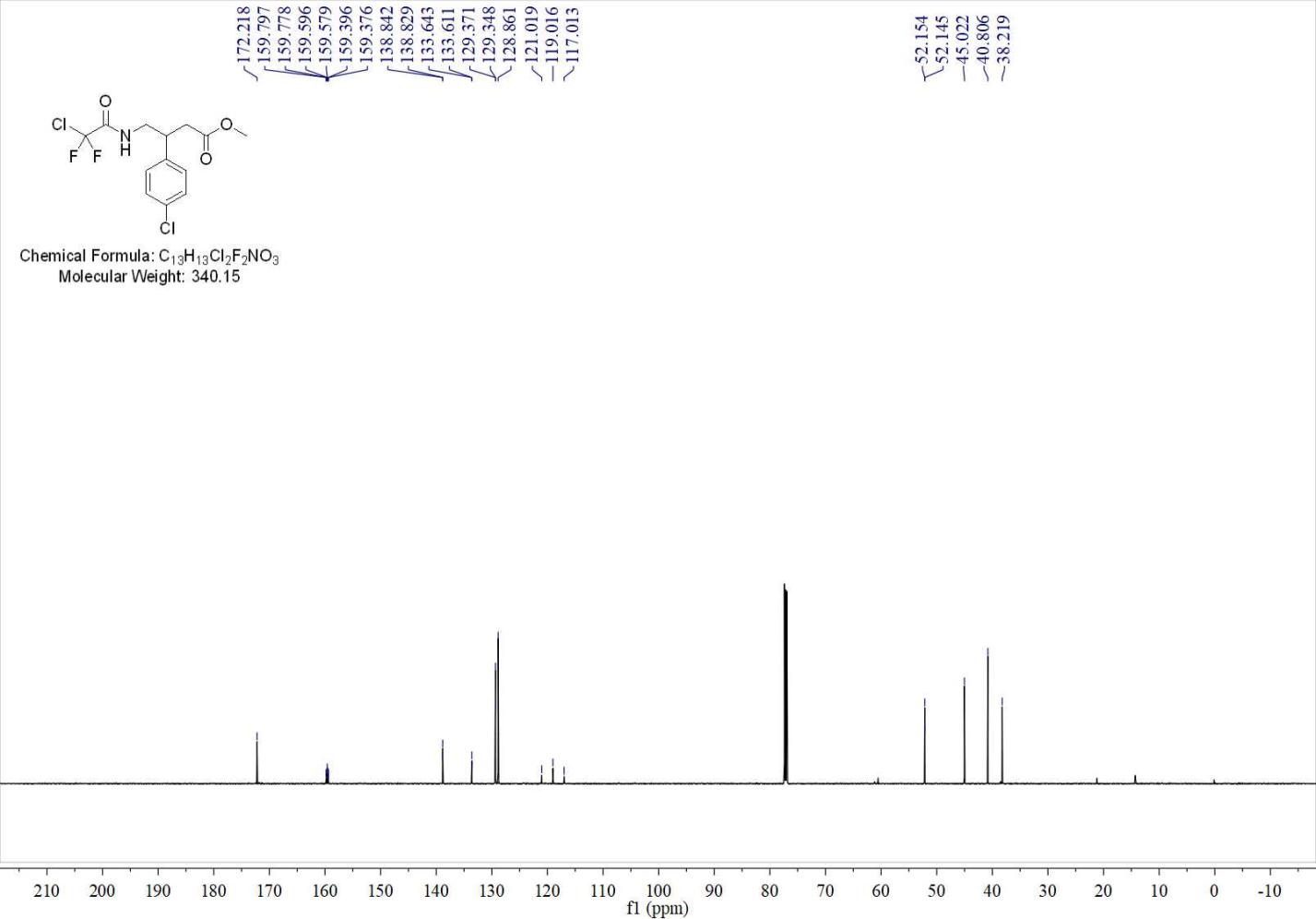


**Methyl (S)-3-((2-chloro-2,2-difluoroacetamido) methyl)-5-methylhexanoate (2v).**


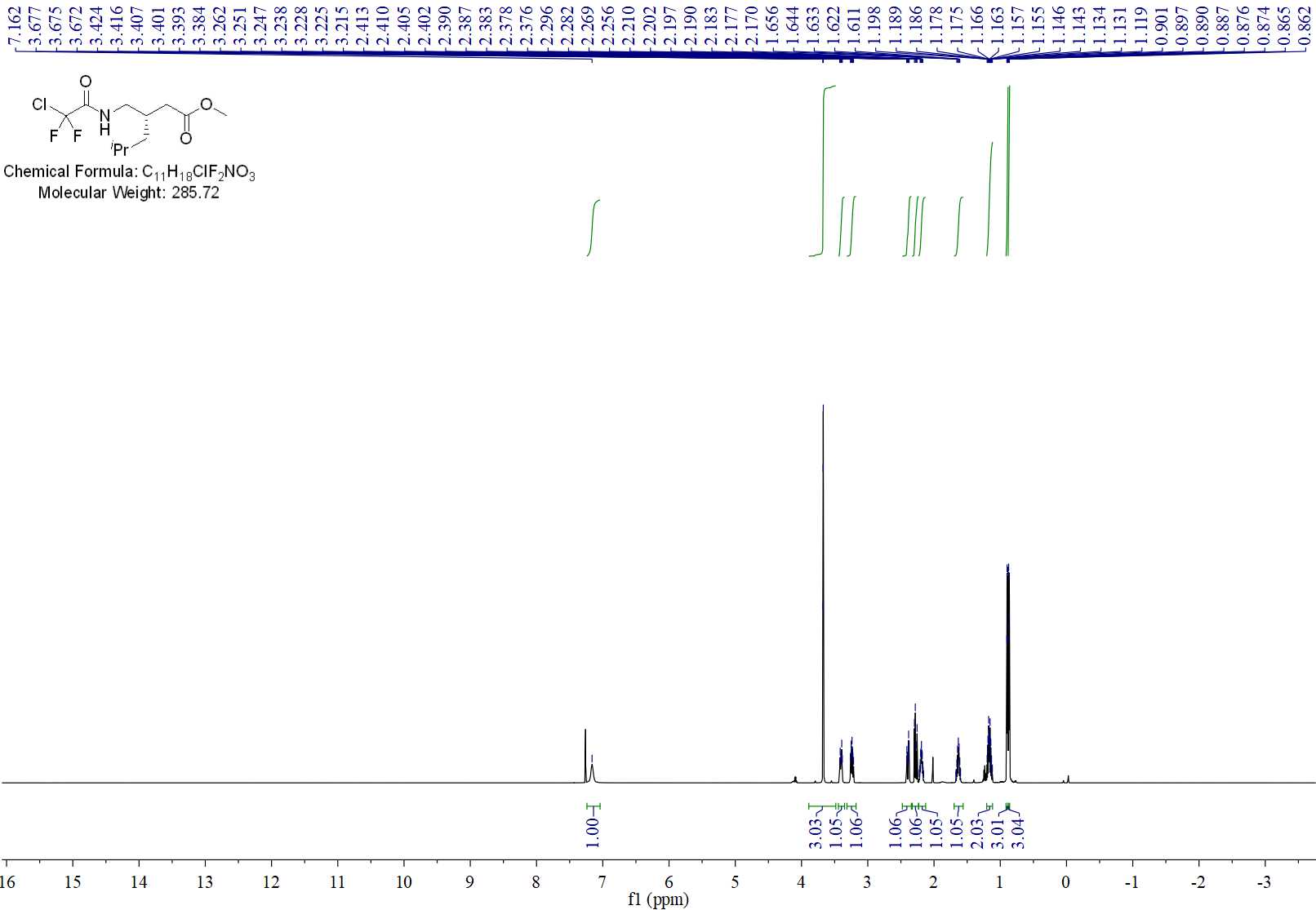

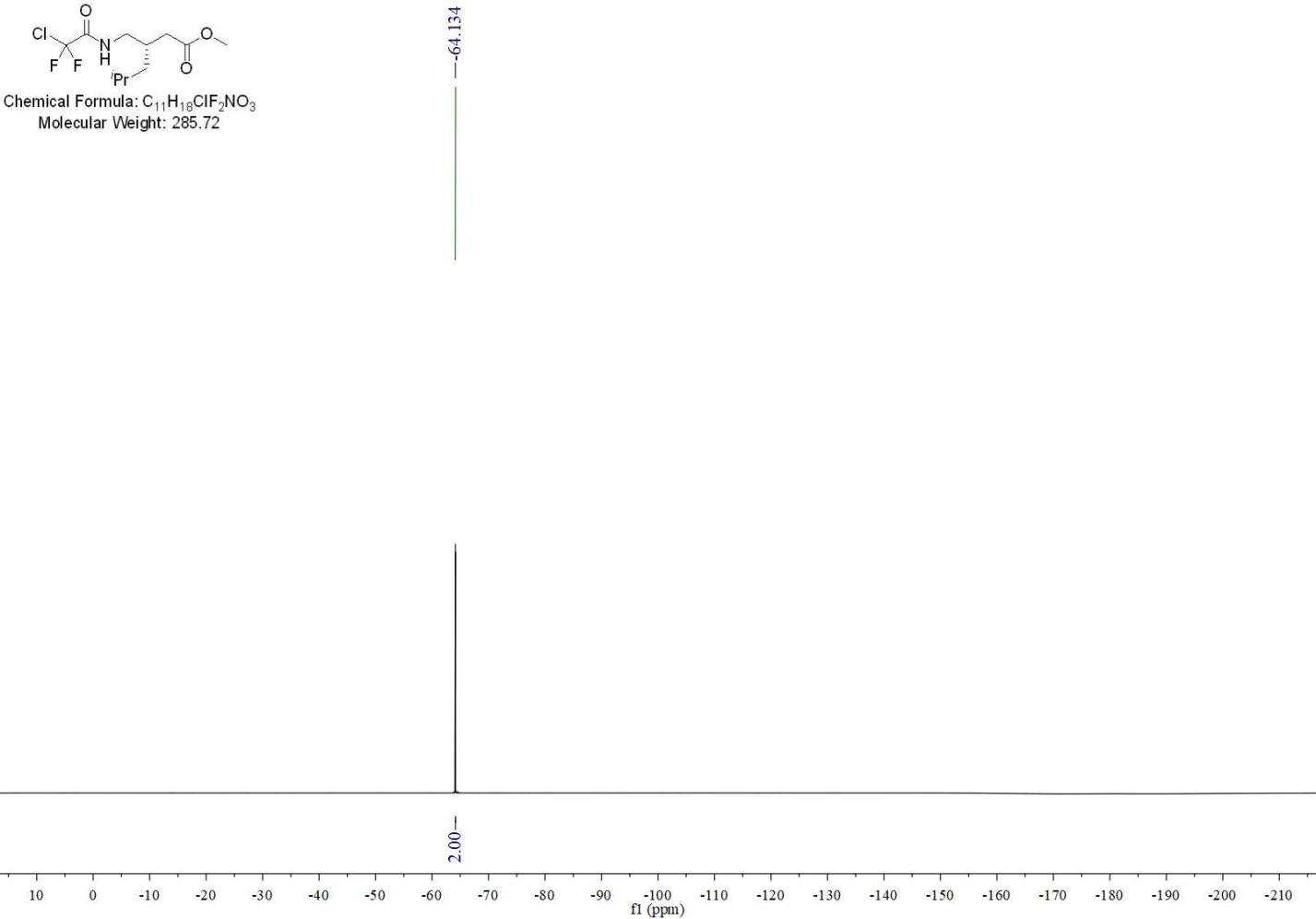

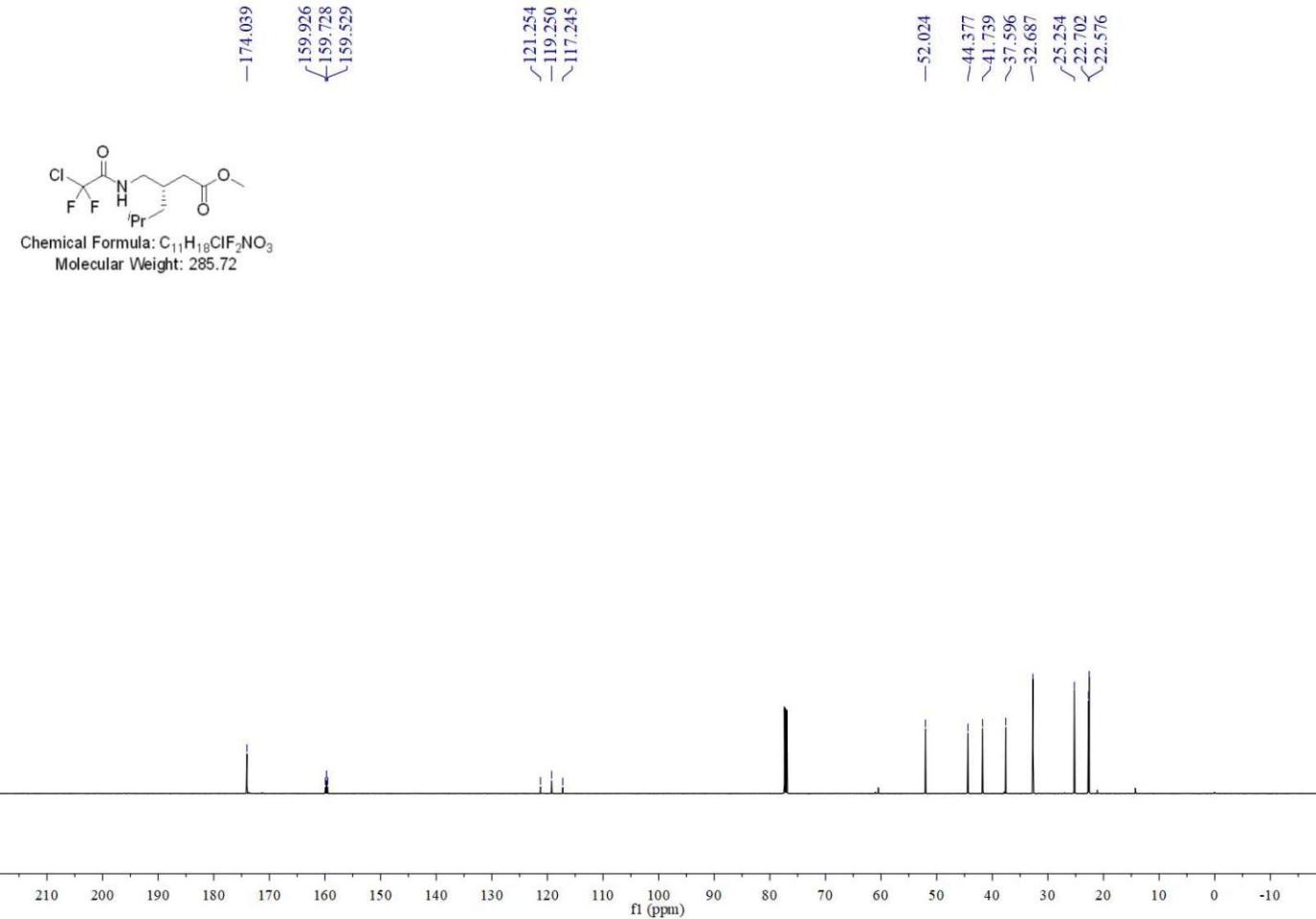


**4-(2-((2,4-dimethylphenyl)thio)phenyl)piperazin-1-yl benzoate (3o).**


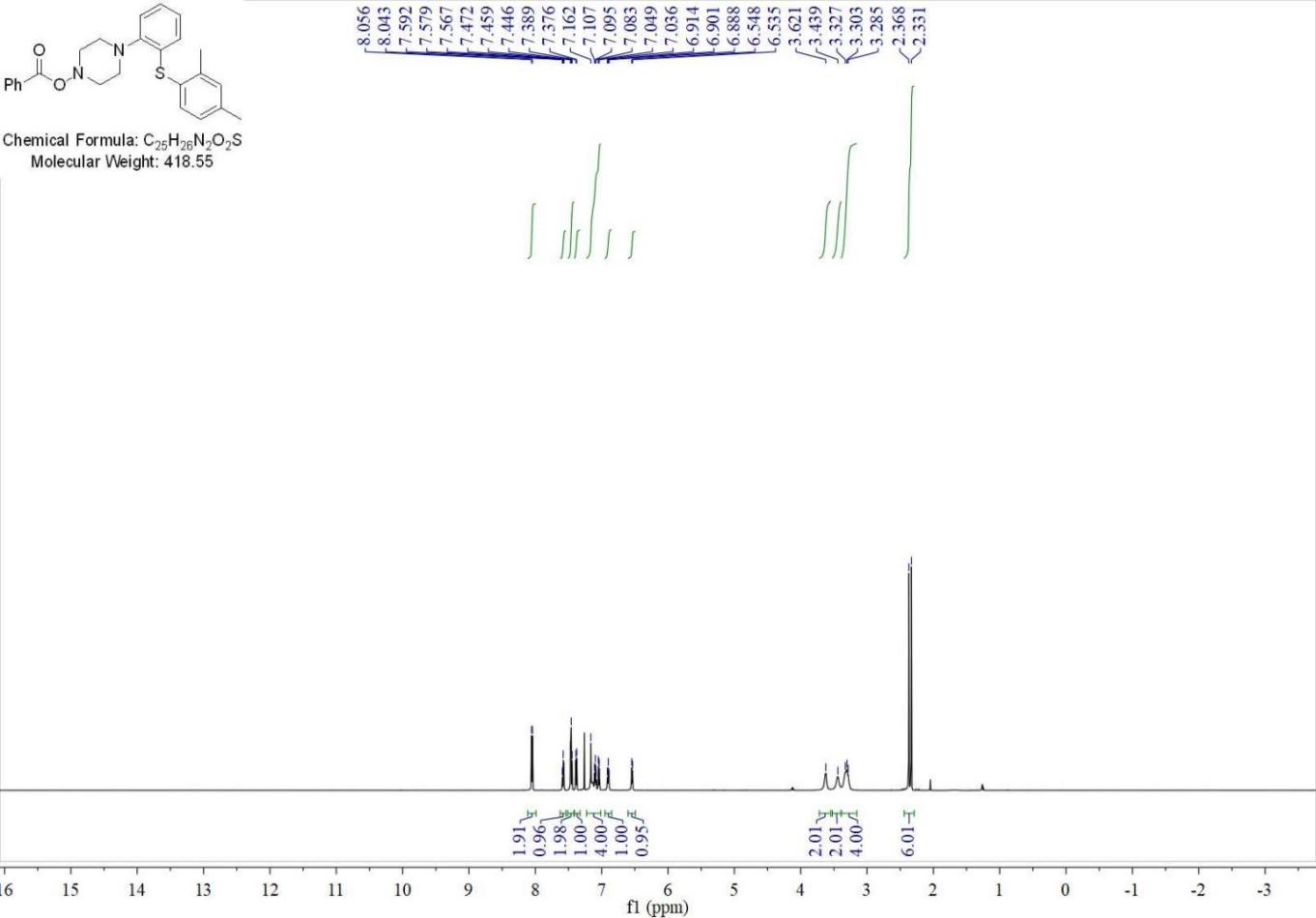

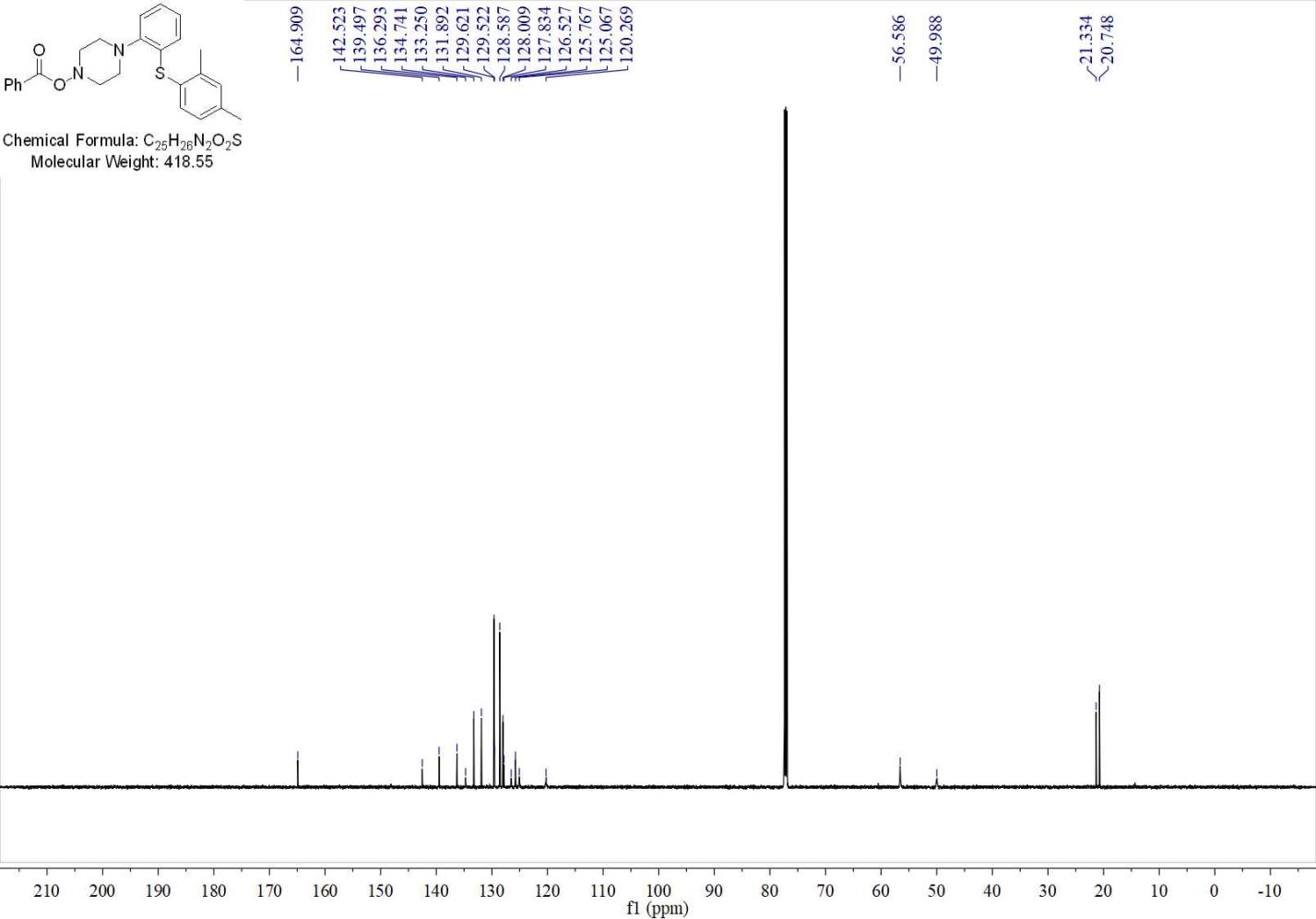


**4-(2-(3-cyano-4-isobutoxyphenyl)-4-methylthiazole-5-carbonyl)piperazin-1-yl benzoate (3s).**


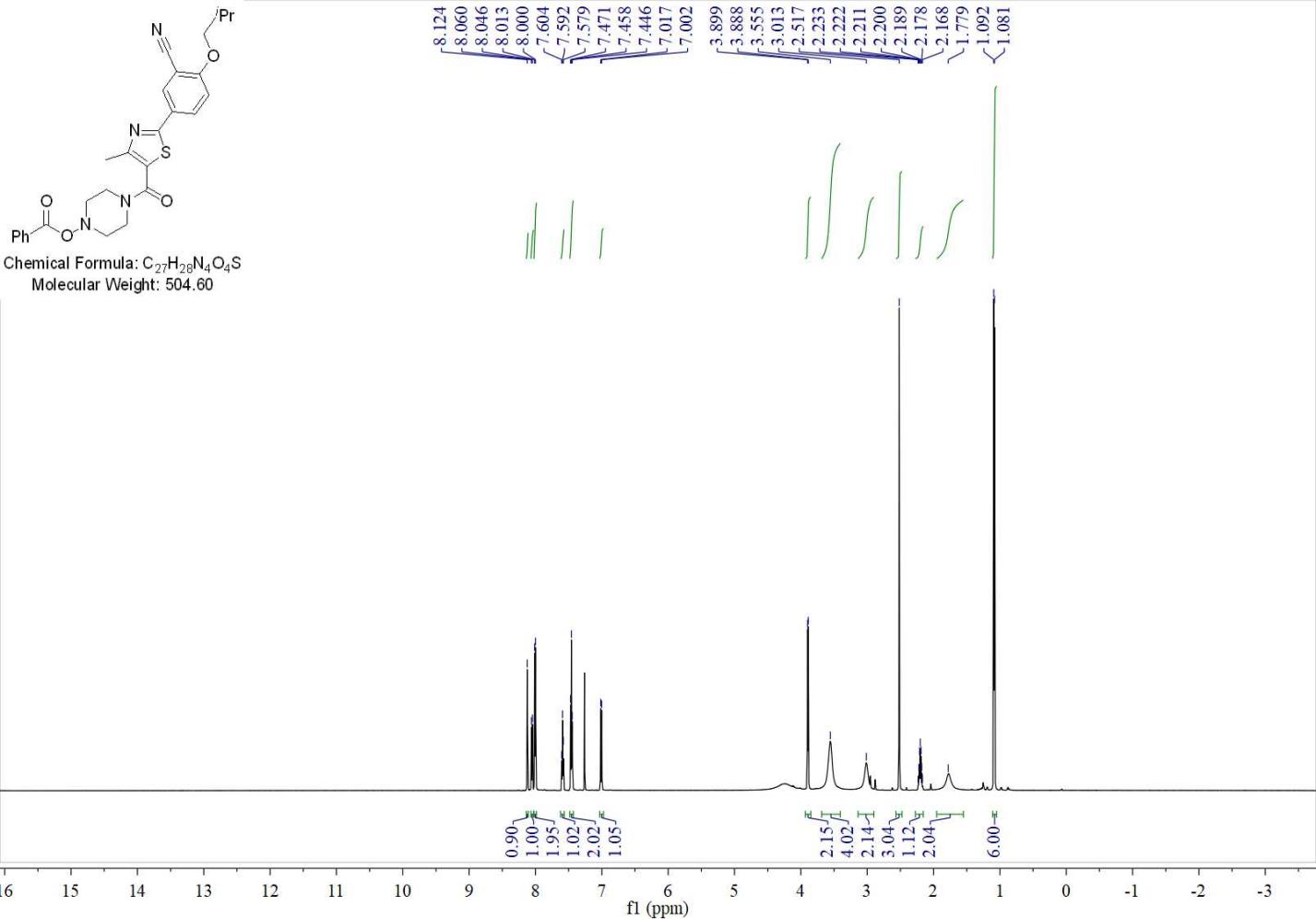

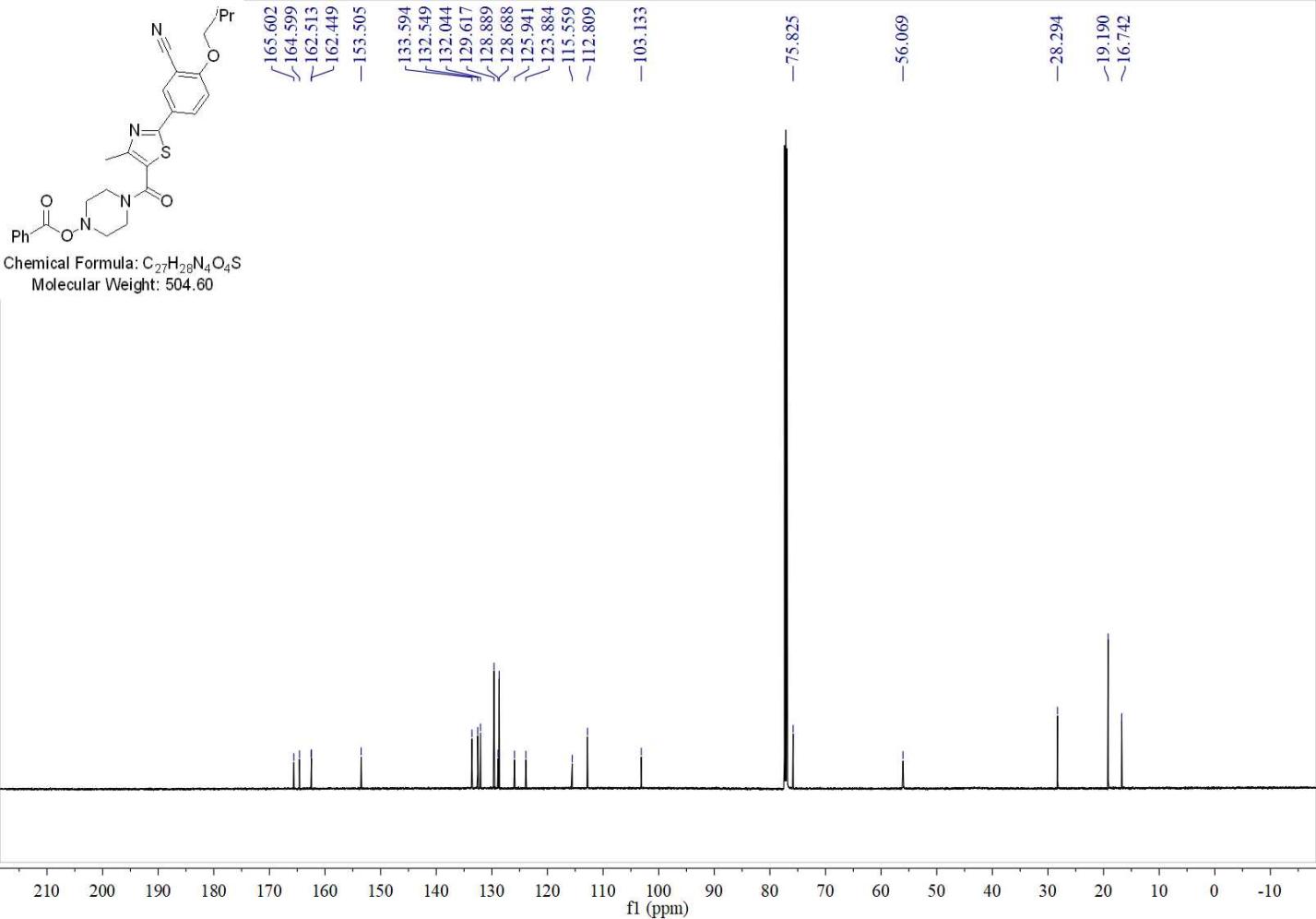


# Characterization Spectra for compounds 4-12, 14 and 16

***N*-(4-(diethylamino)-3,3-difluoro-1-morpholino-4-oxobutyl) benzamide (4a).**

**
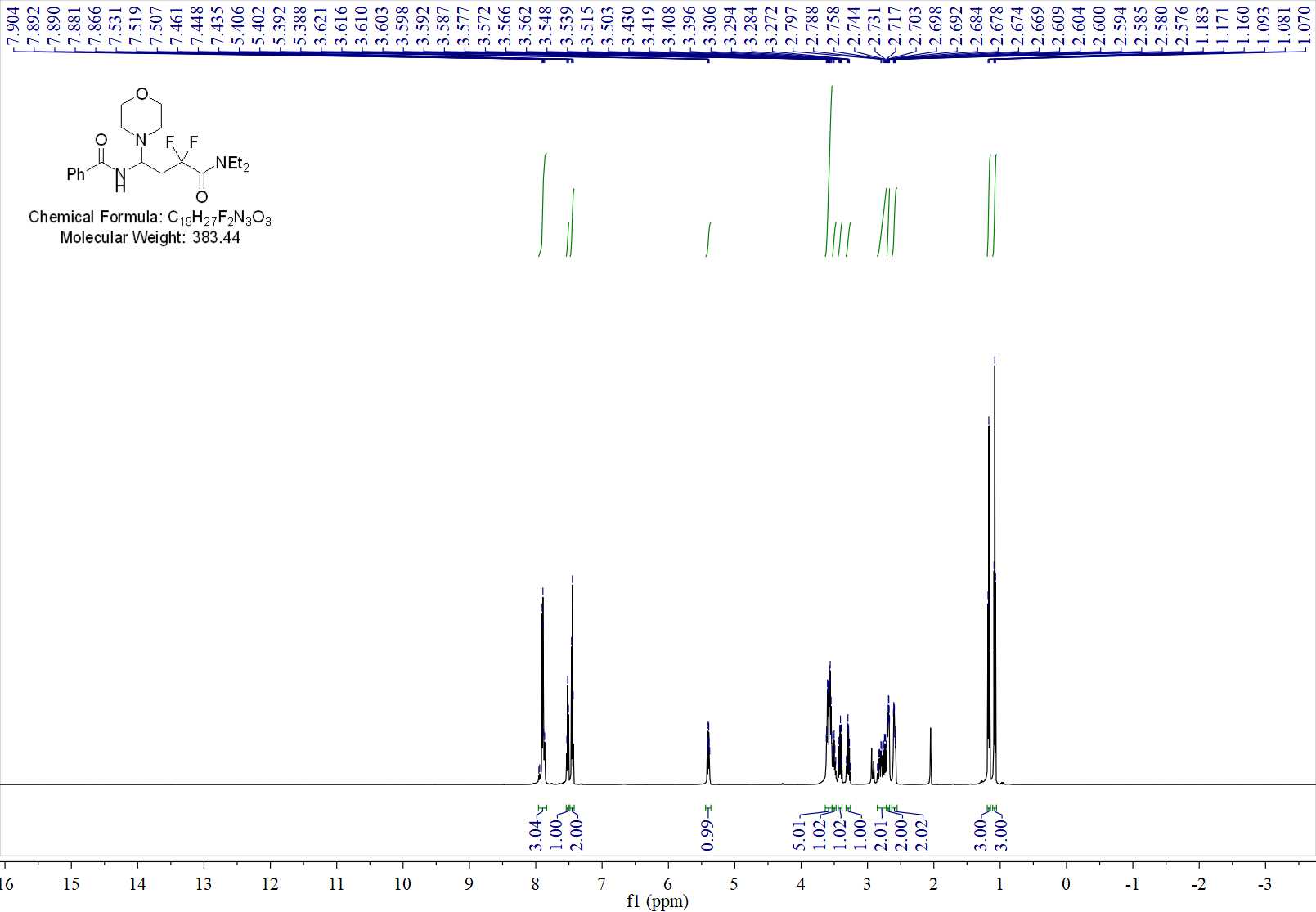

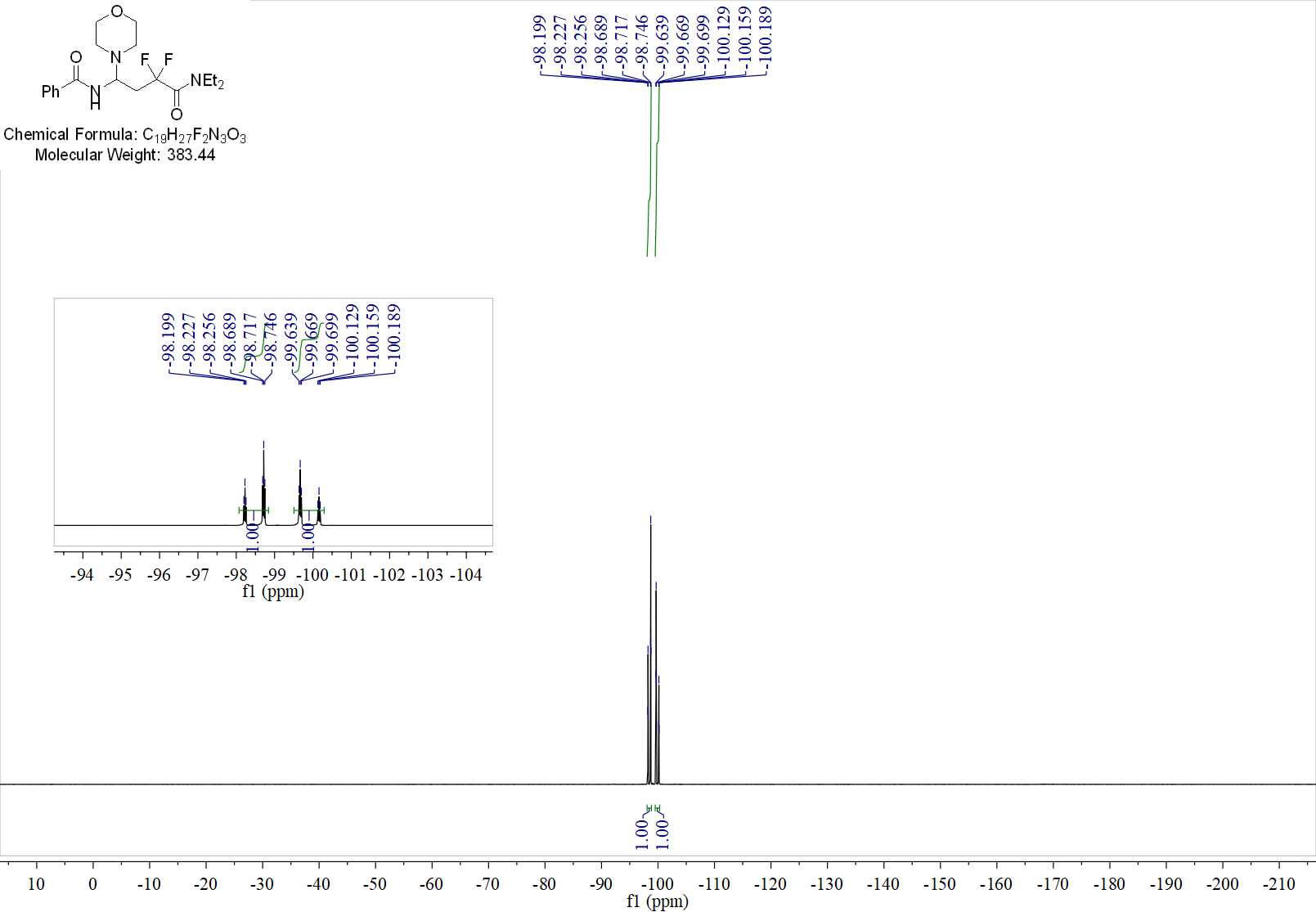

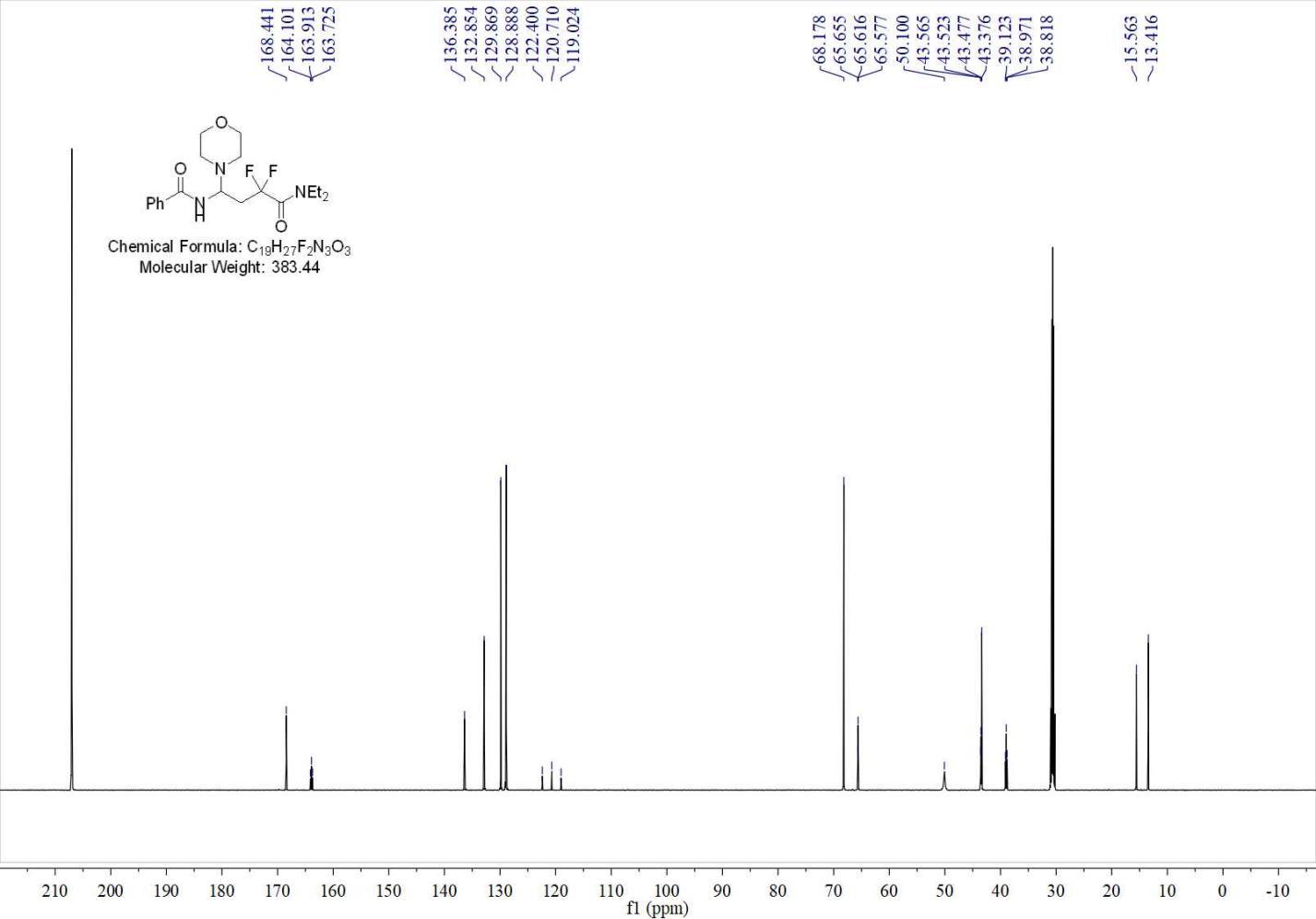
**

***N*-(4-(dibutylamino)-3,3-difluoro-1-morpholino-4-oxobutyl)benzamide (4b).**

**
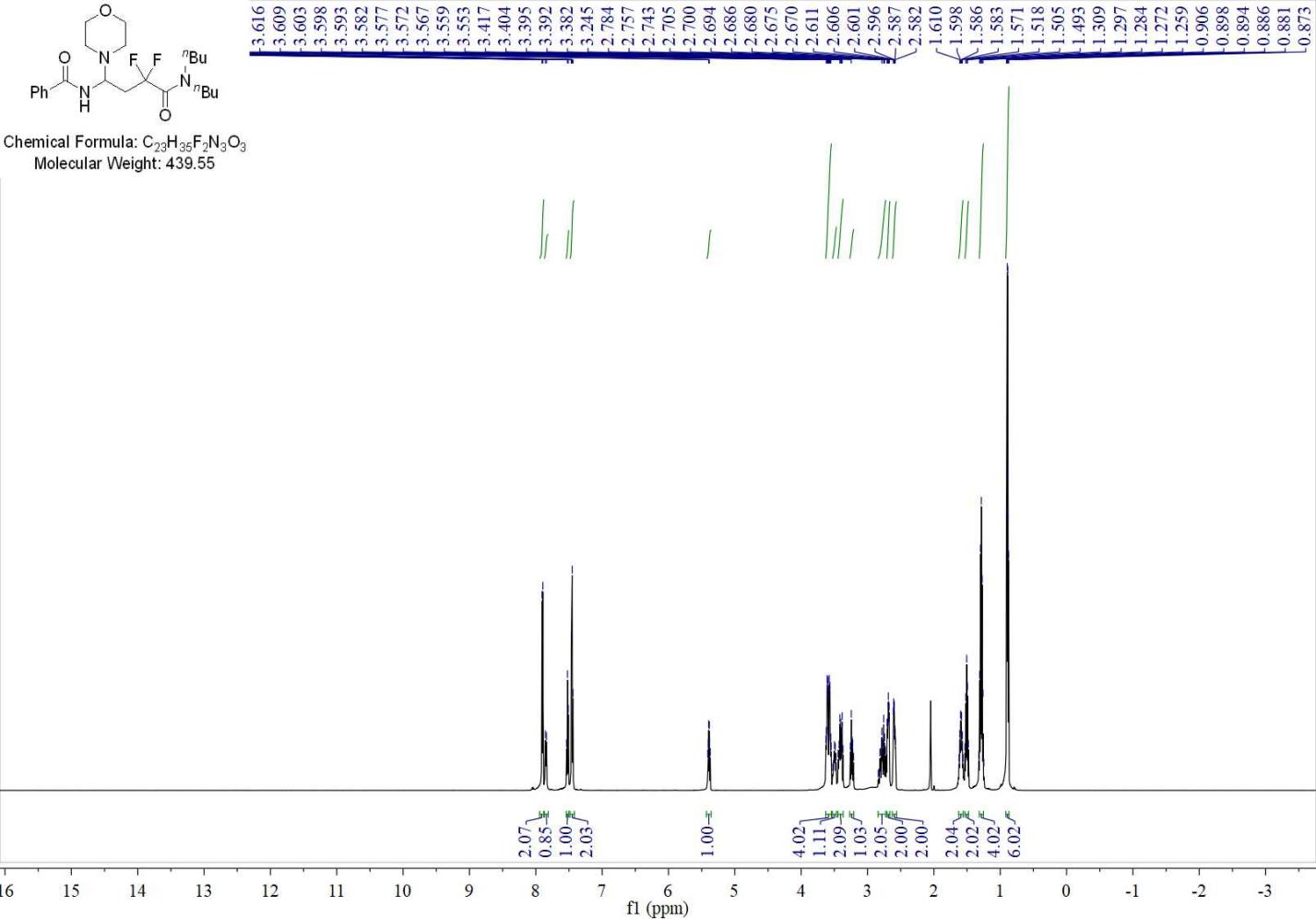

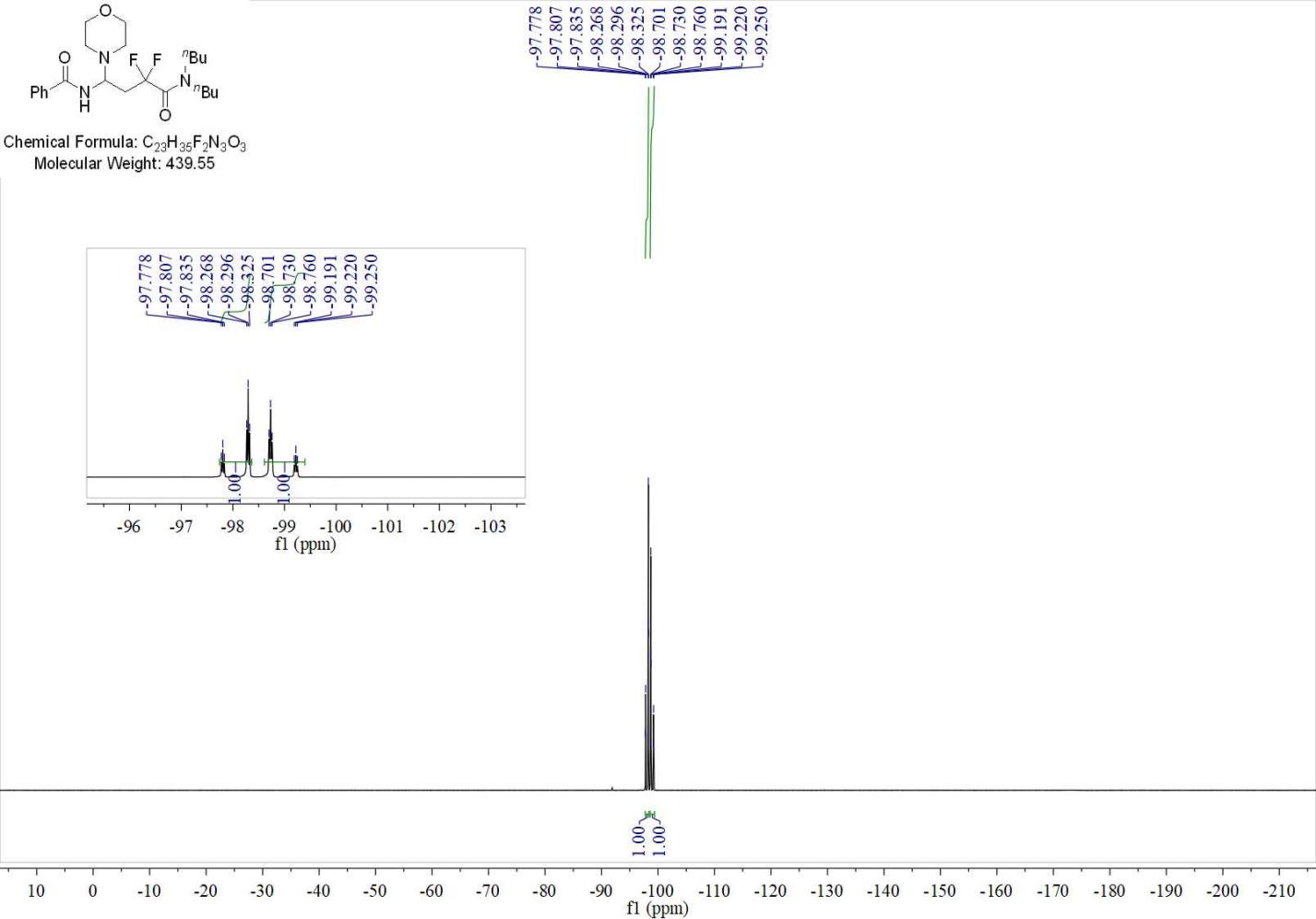

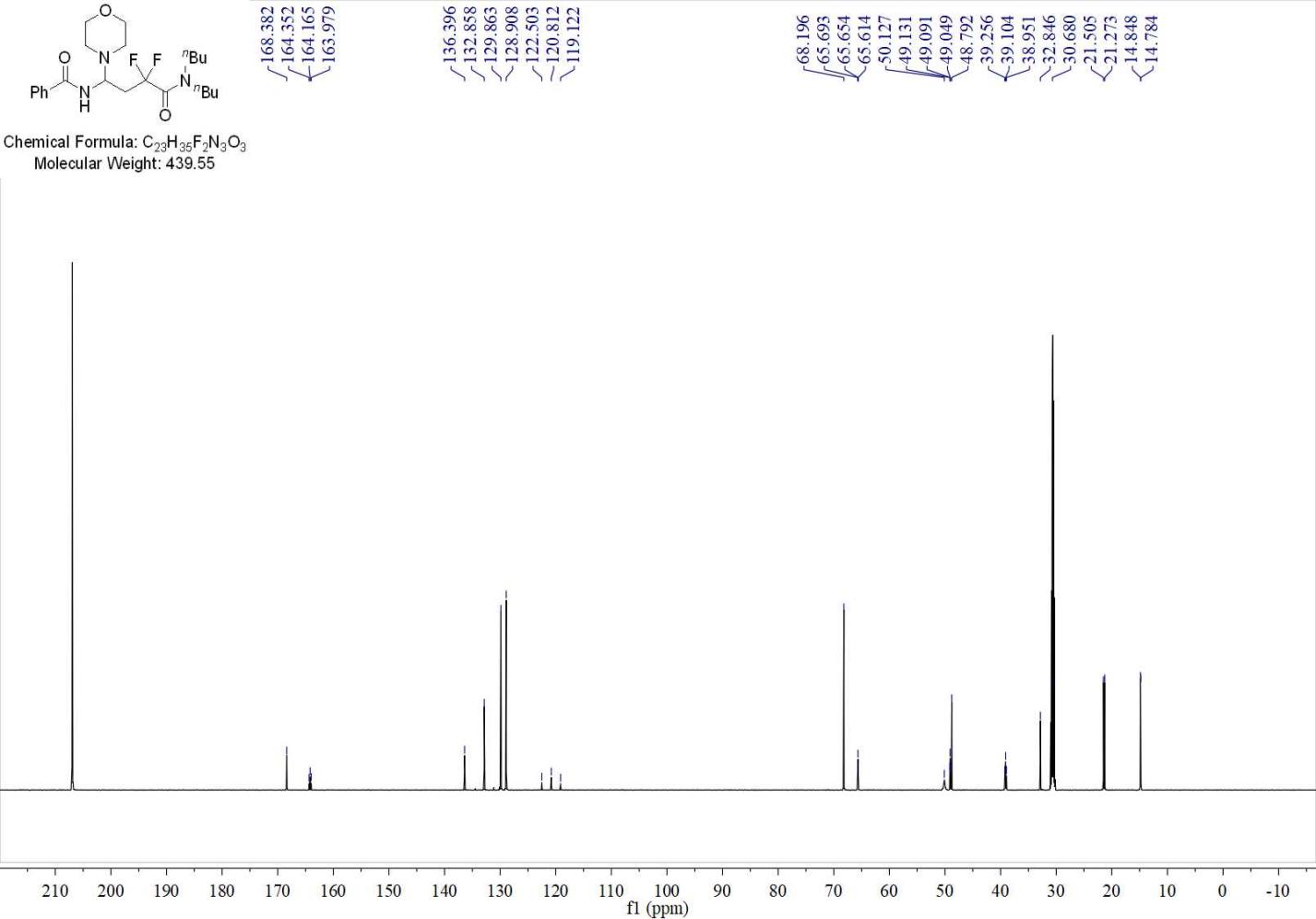
**

***N*-(4-(azetidin-1-yl)-3,3-difluoro-1-morpholino-4-oxobutyl) benzamide (4c).**

**
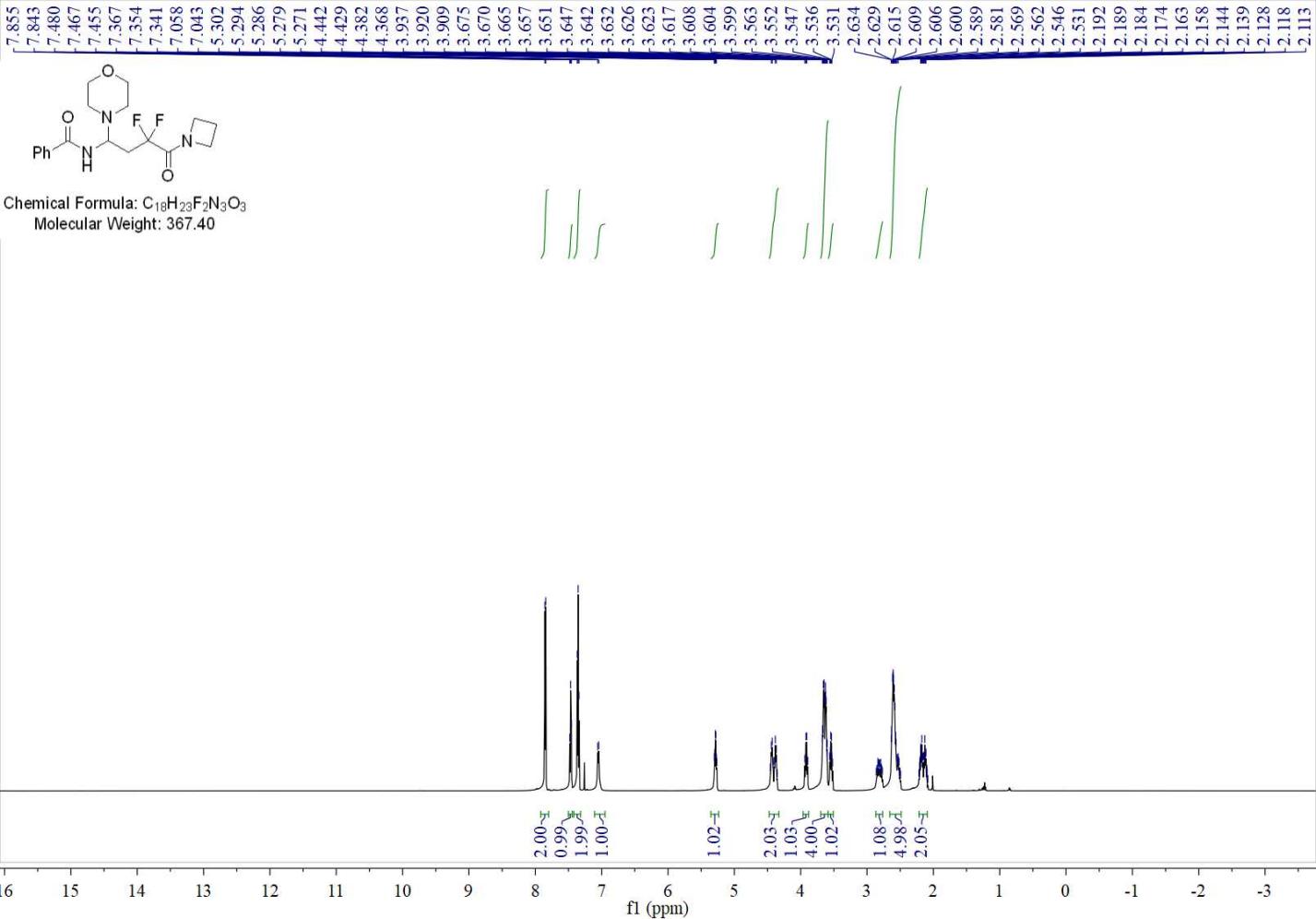

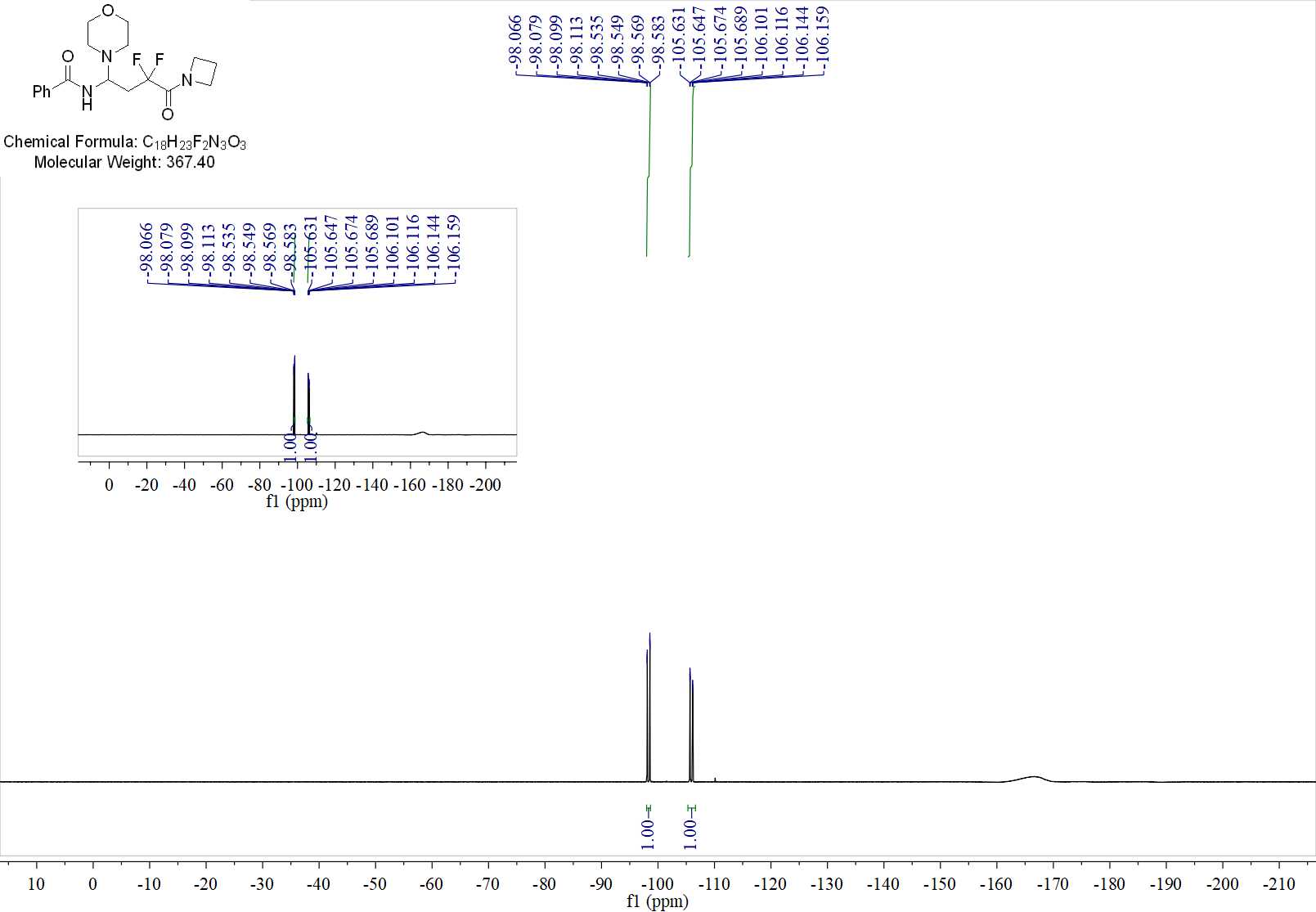

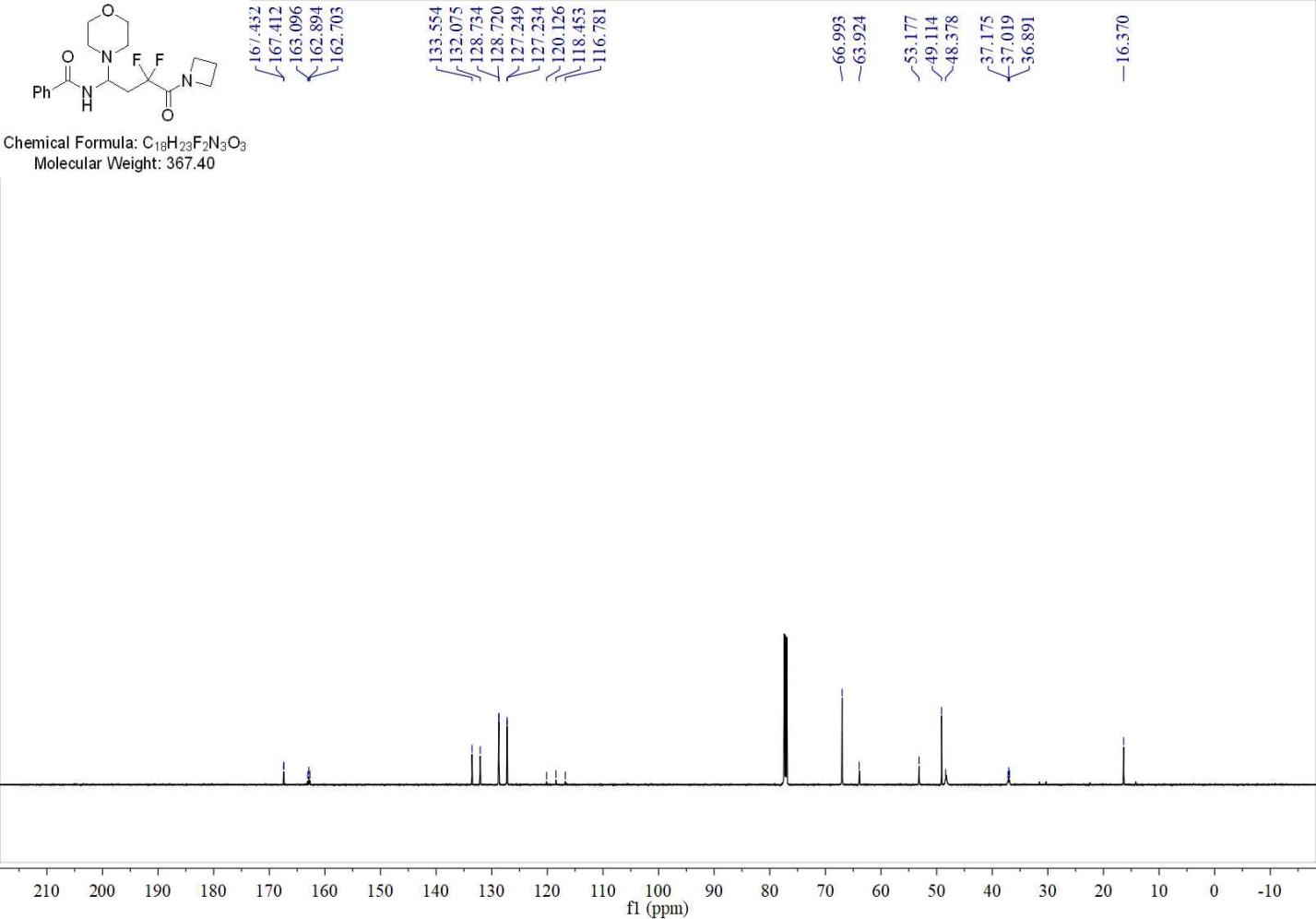
**

***N*-(3,3-difluoro-1-morpholino-4-oxo-4-(pyrrolidin-1-yl) butyl) benzamide (4d).**

**
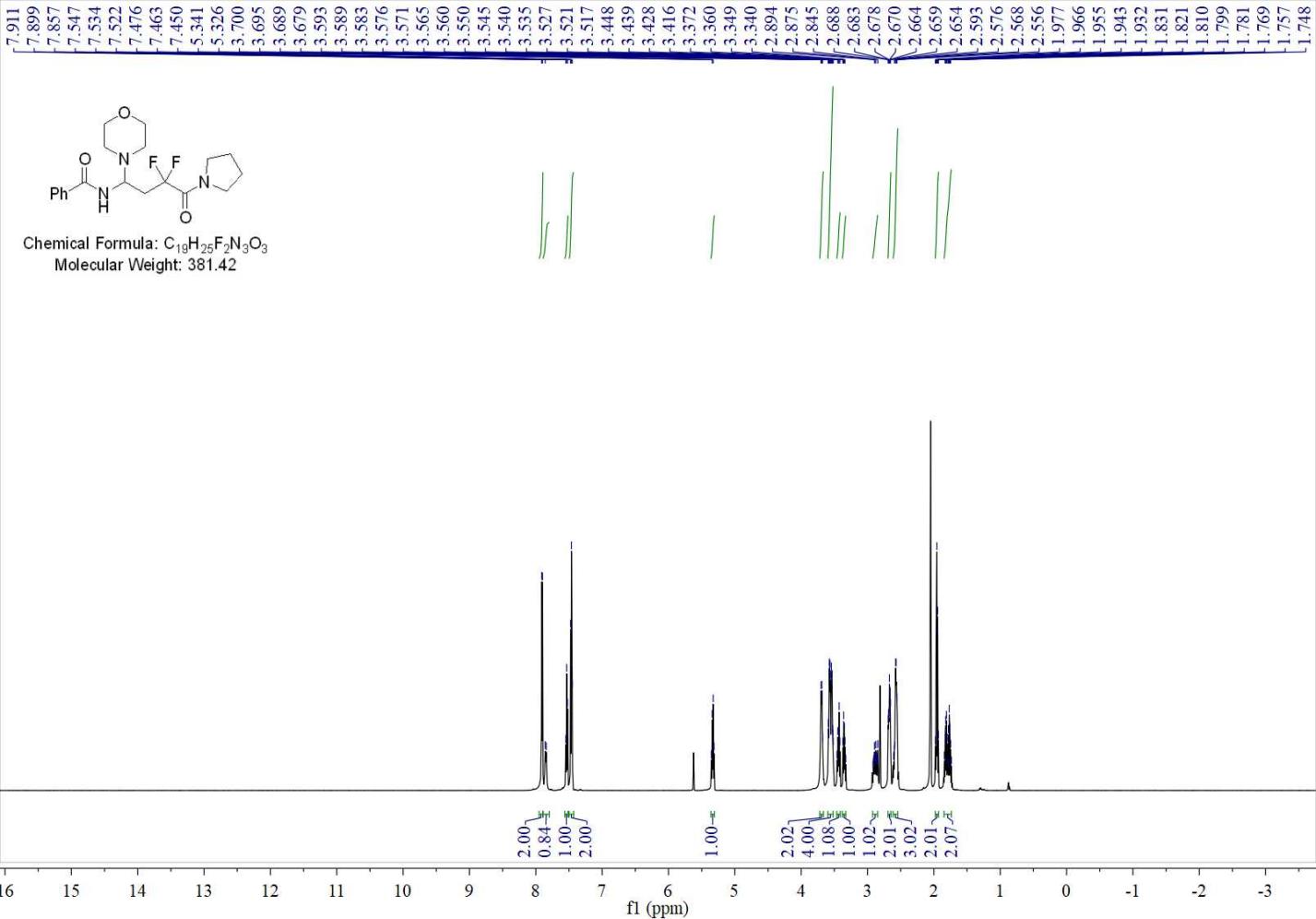

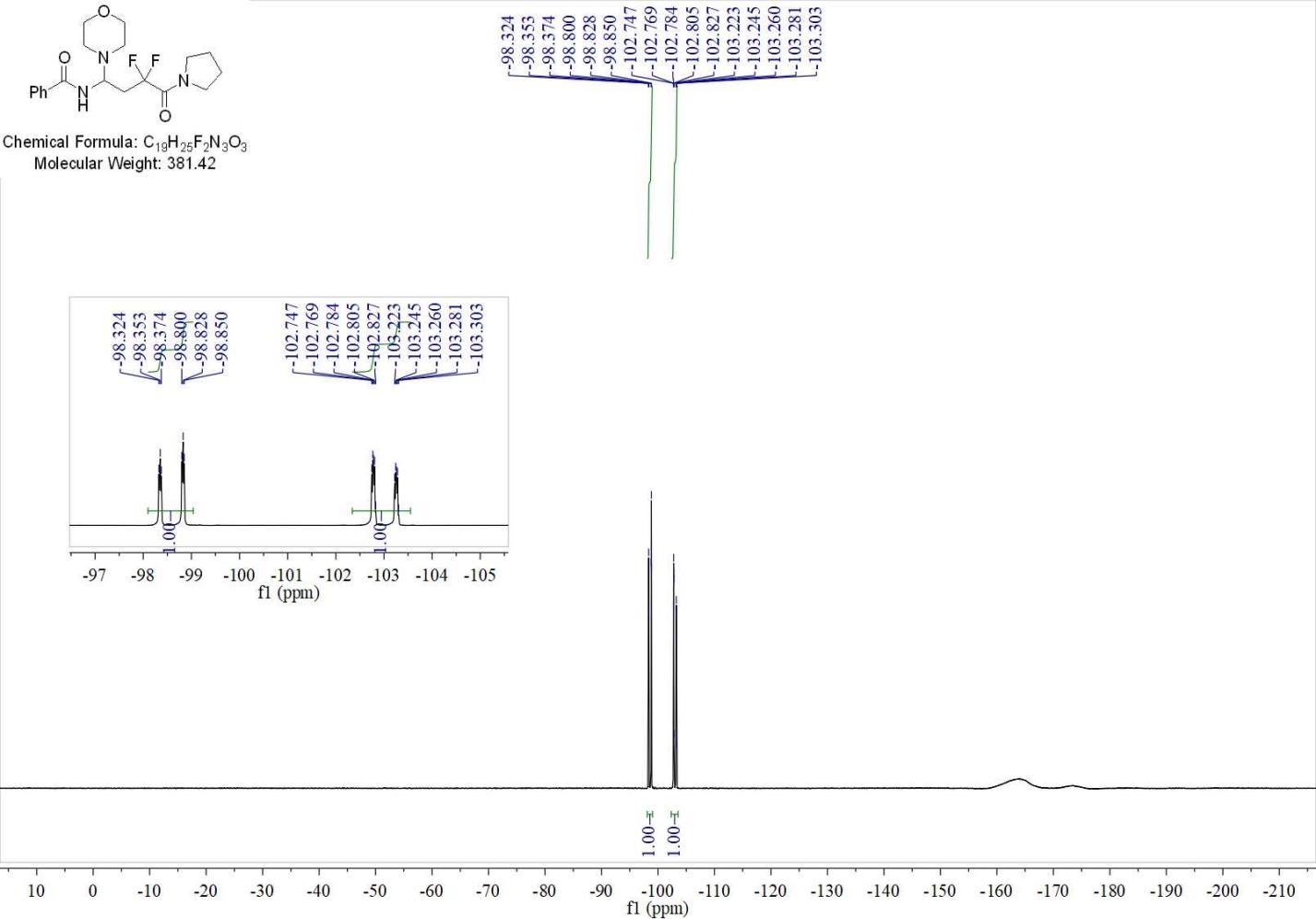

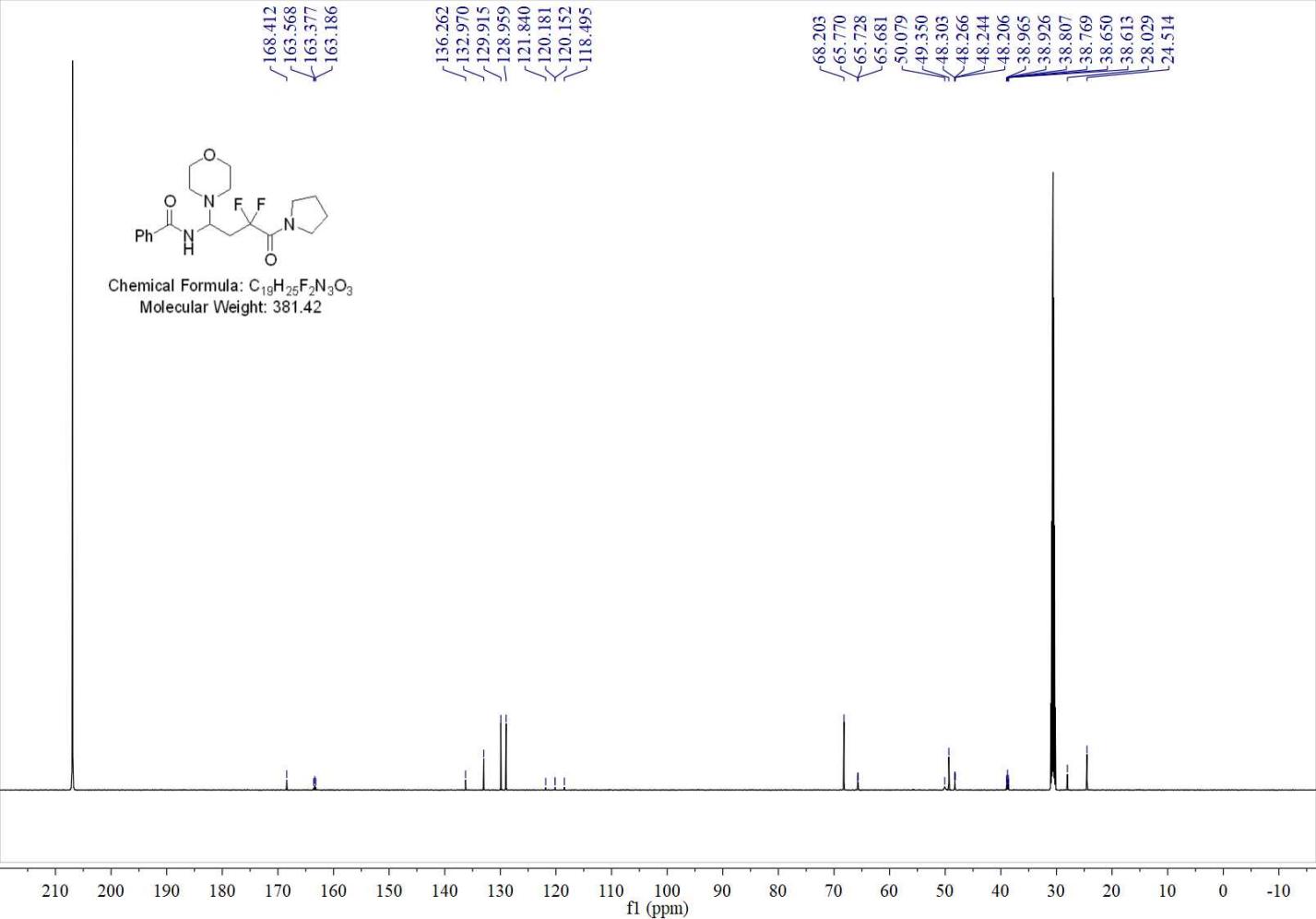
**

***N*-(3,3-difluoro-1,4-dimorpholino-4-oxobutyl)benzamide (4e).**

**
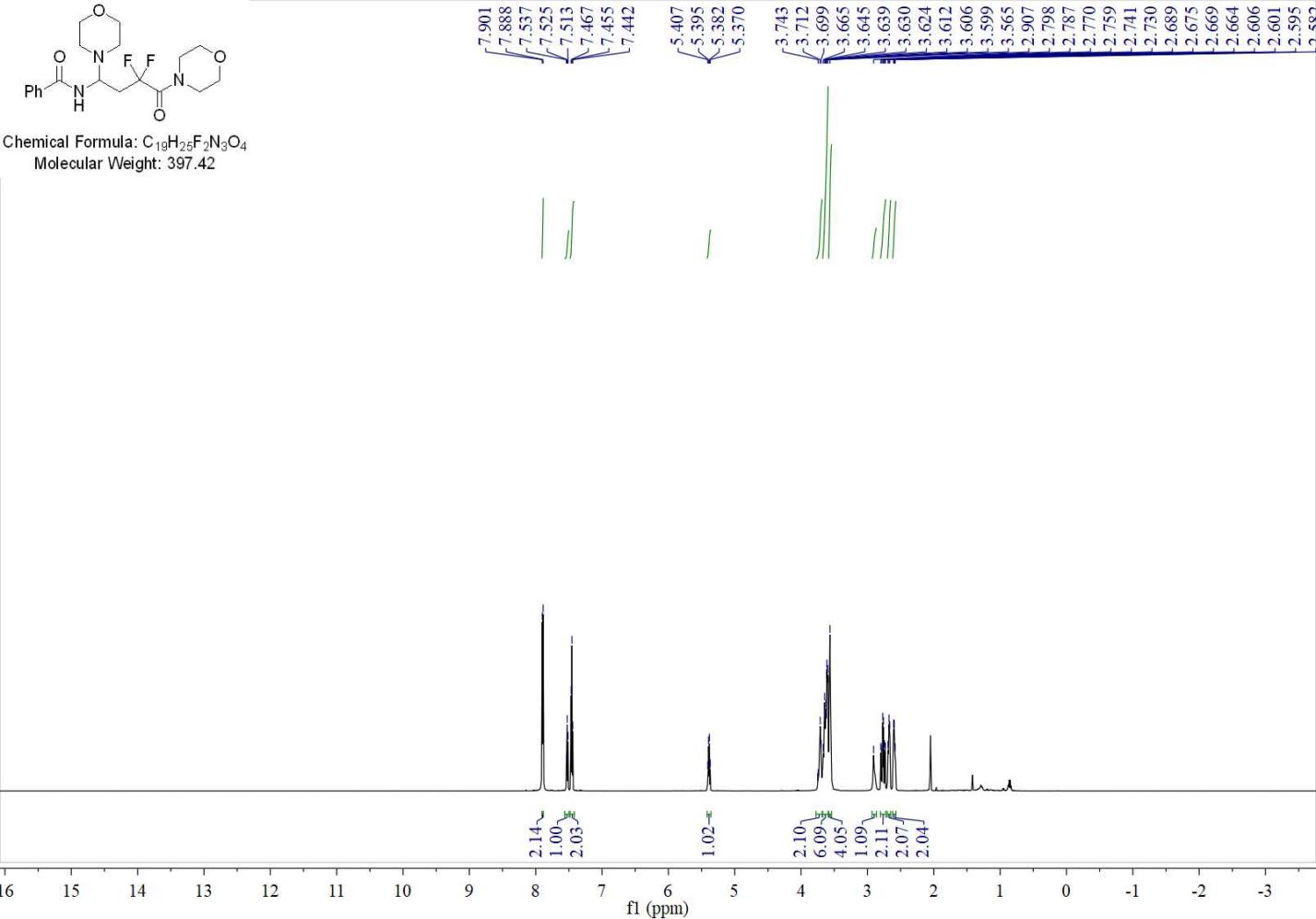

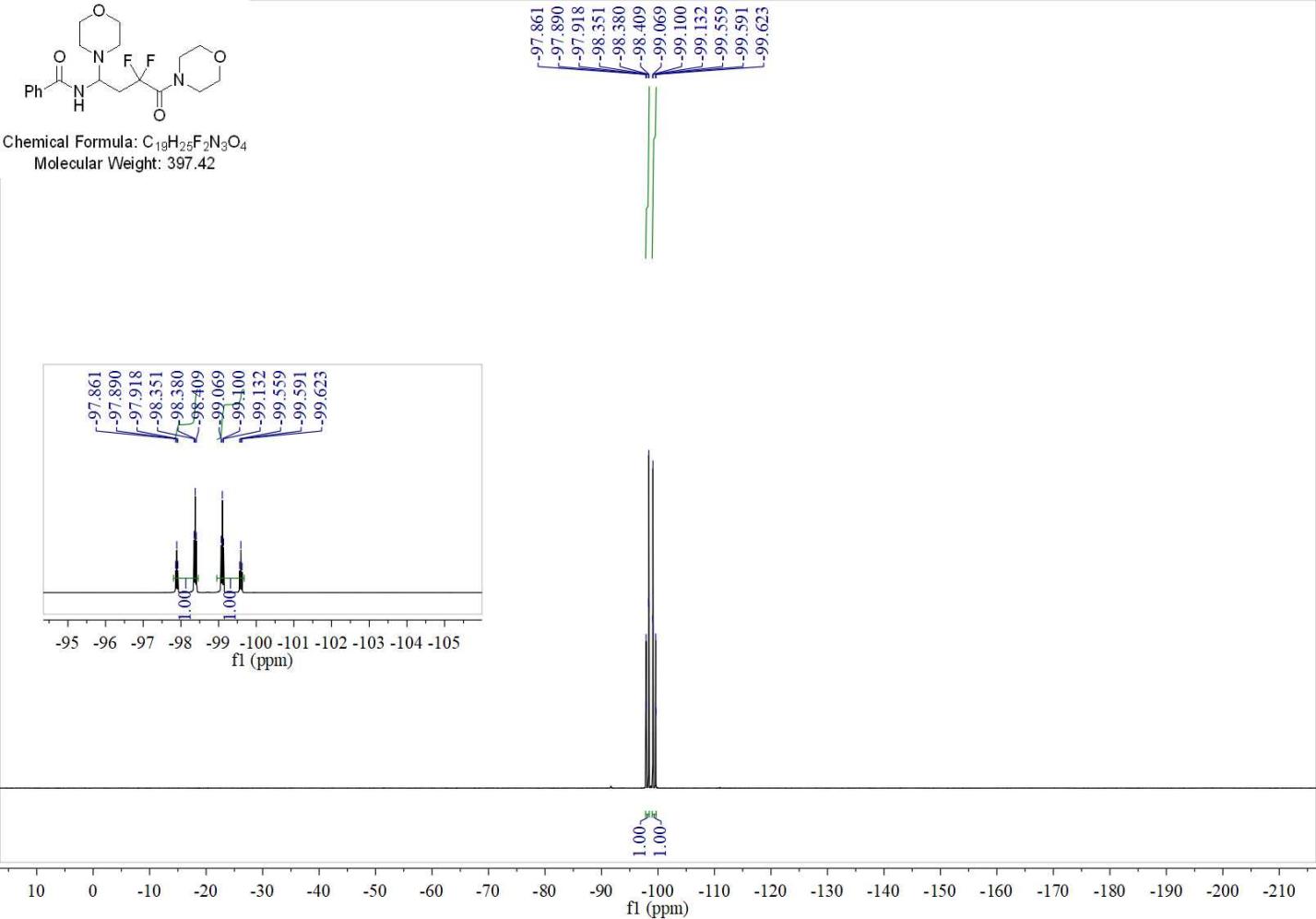

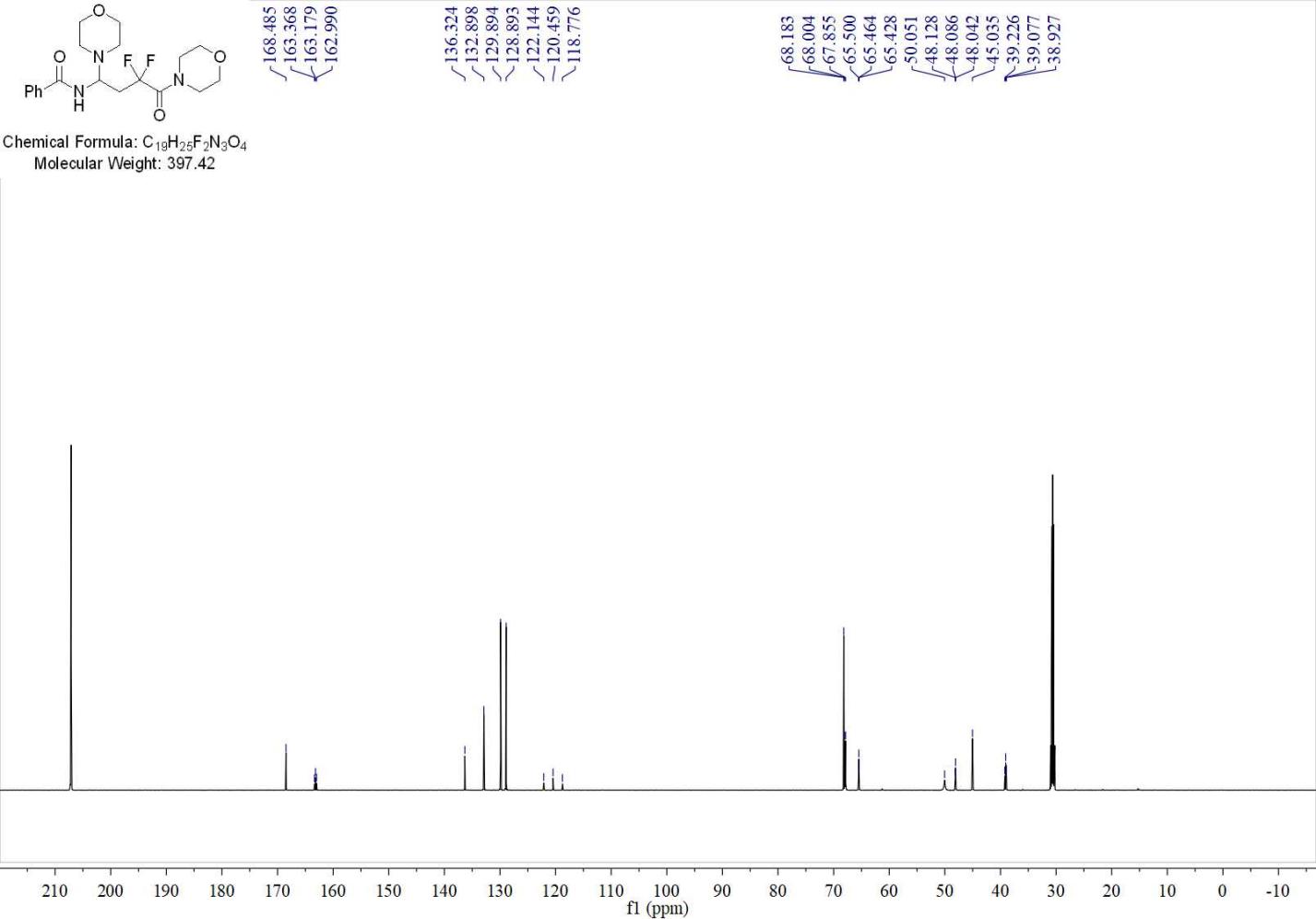
**

***N*-(3,3-difluoro-1-morpholino-4-oxo-4-thiomorpholinobutyl) benzamide (4f).**

**
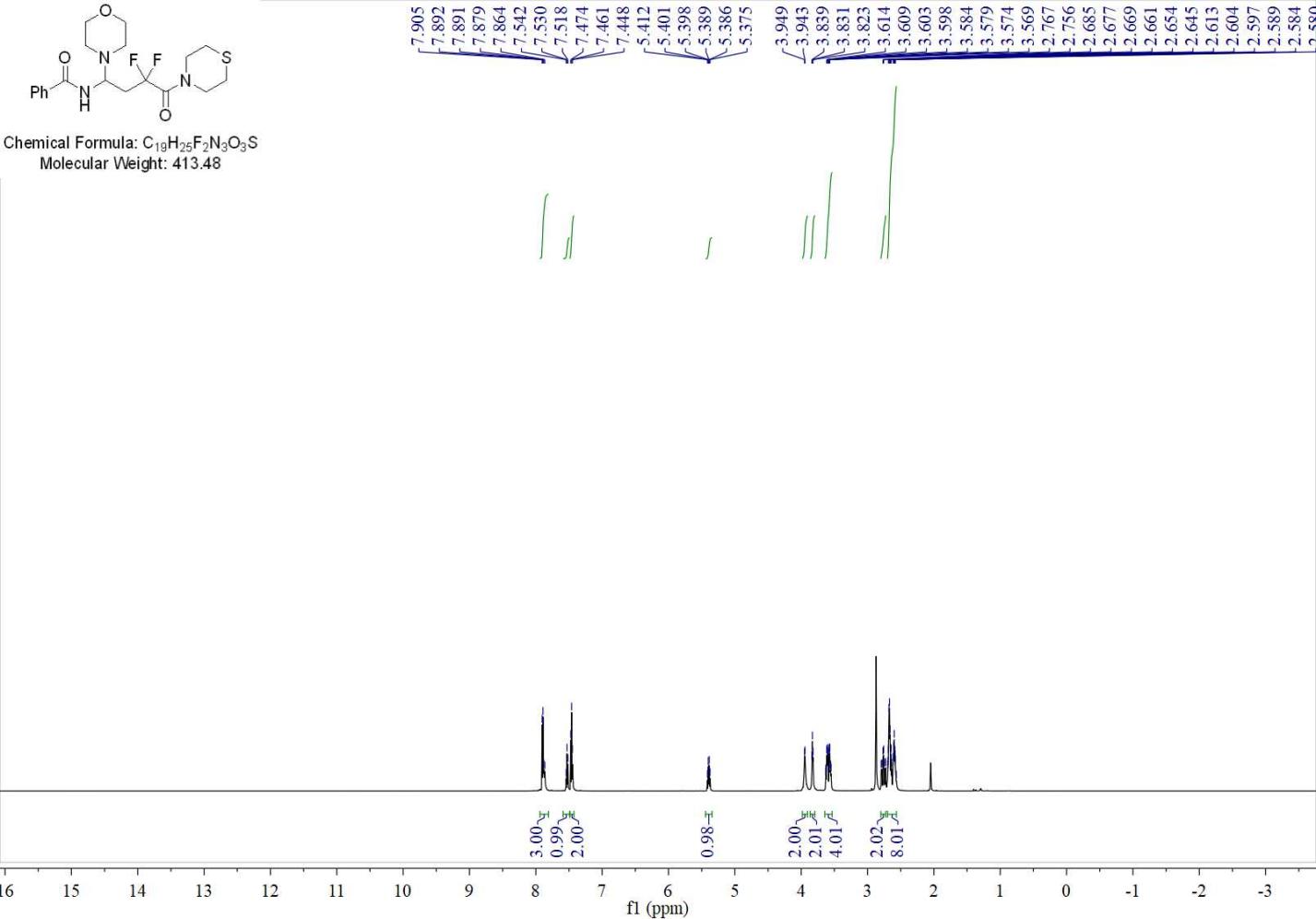

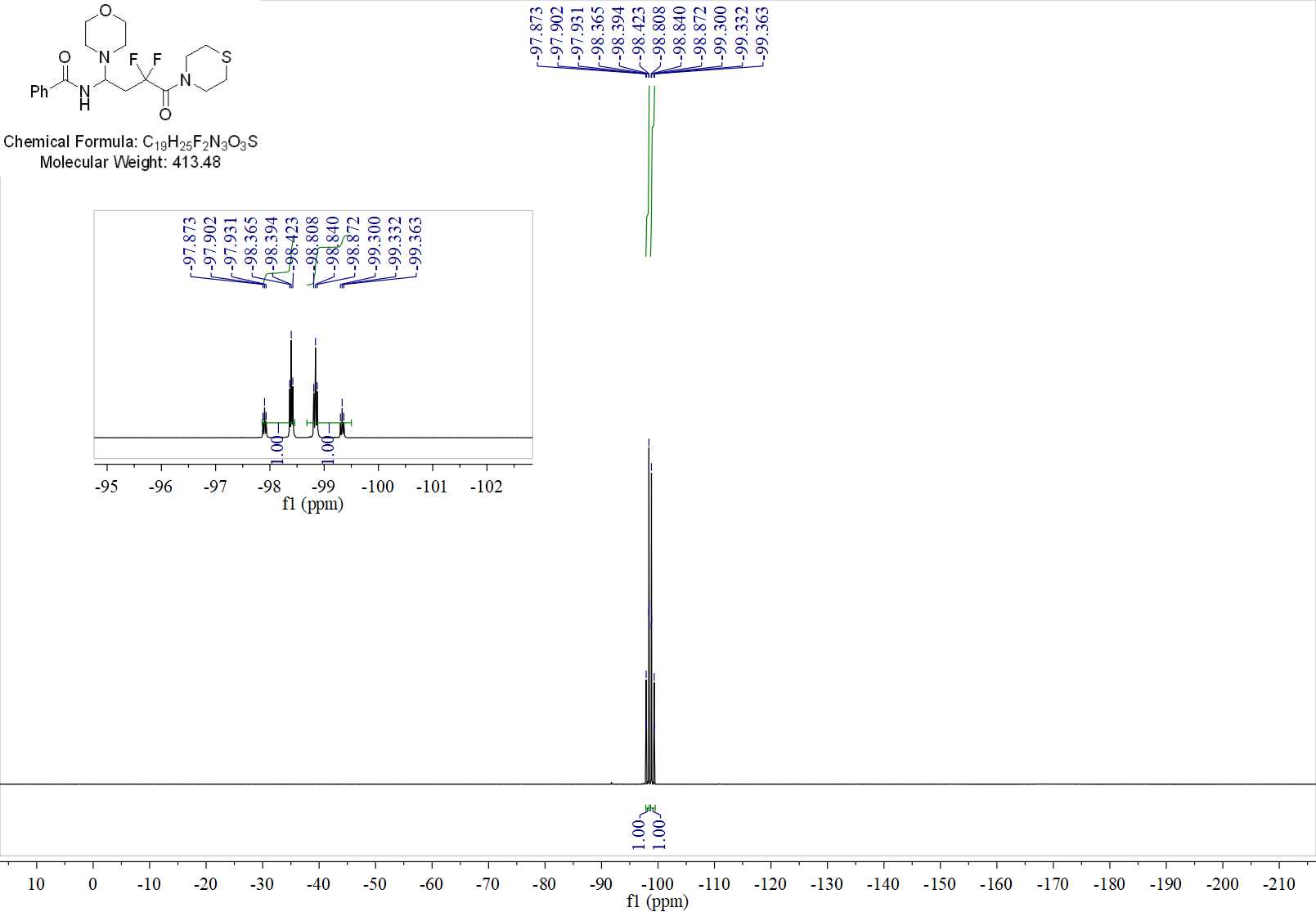
**

**
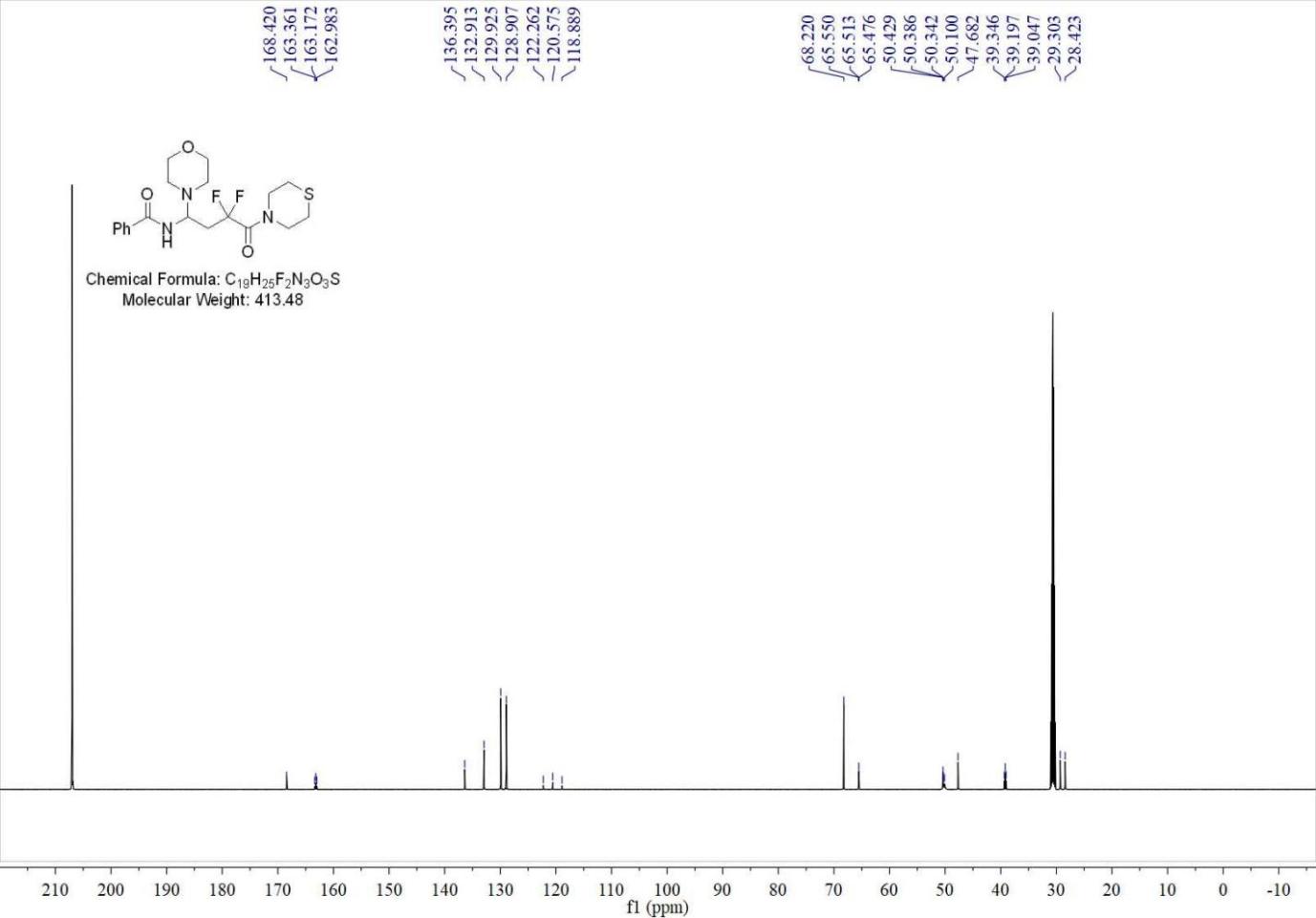
**

***N*-(4-(6,7-dimethoxy-3,4-dihydroisoquinolin-2(1H)-yl)-3,3-difluoro-1-morpholino-4-oxobutyl) benzamide (4g).**

**
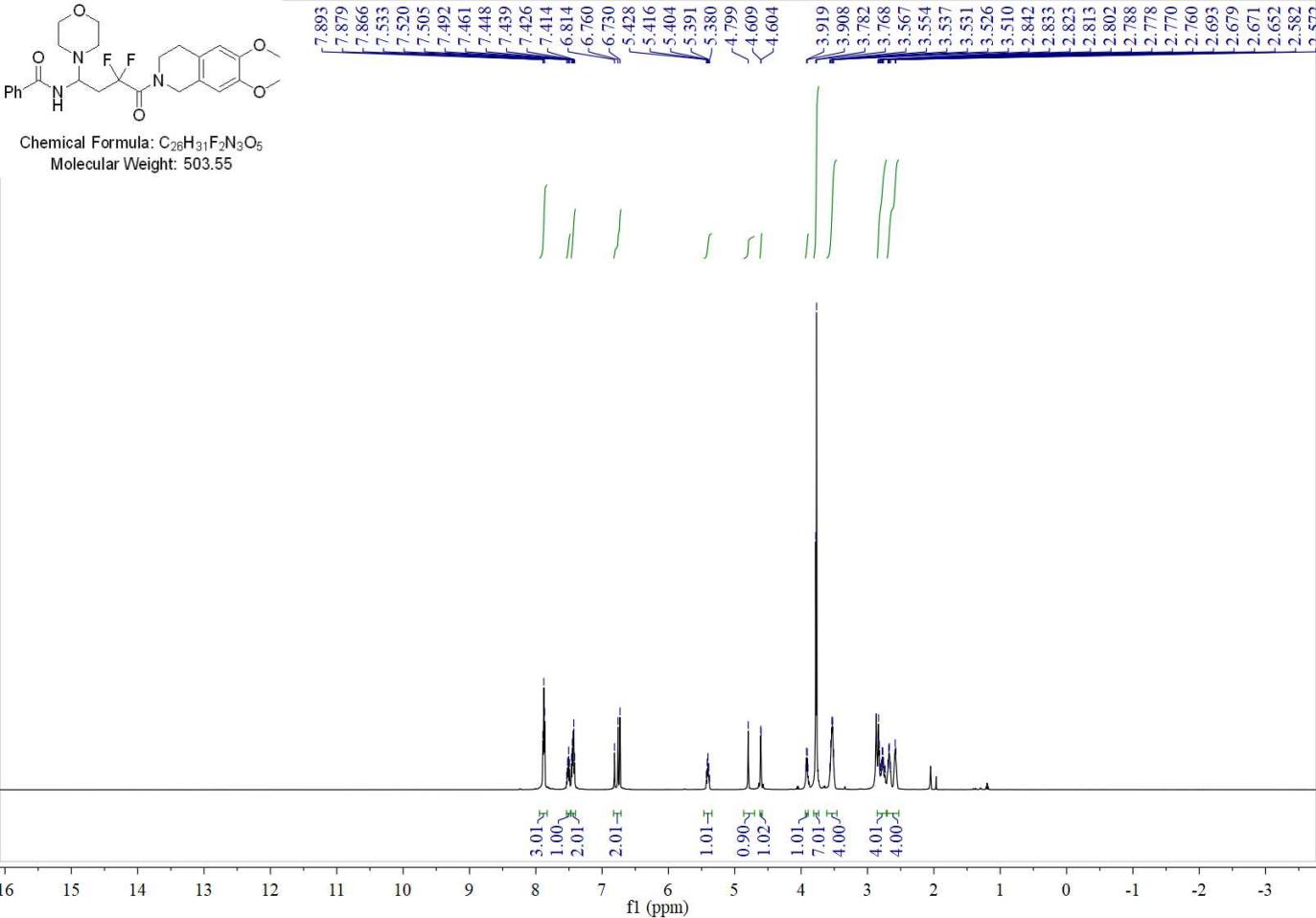

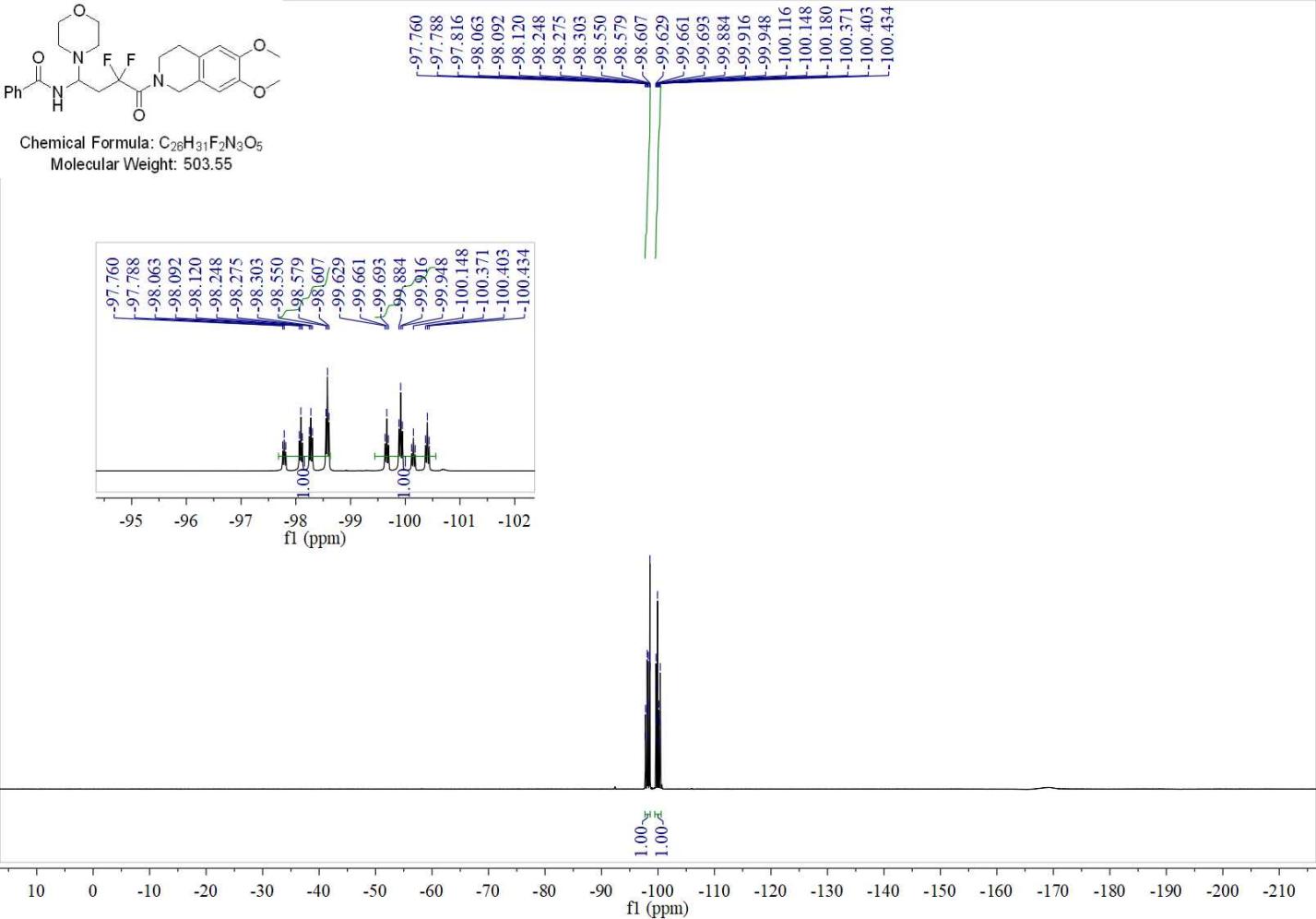

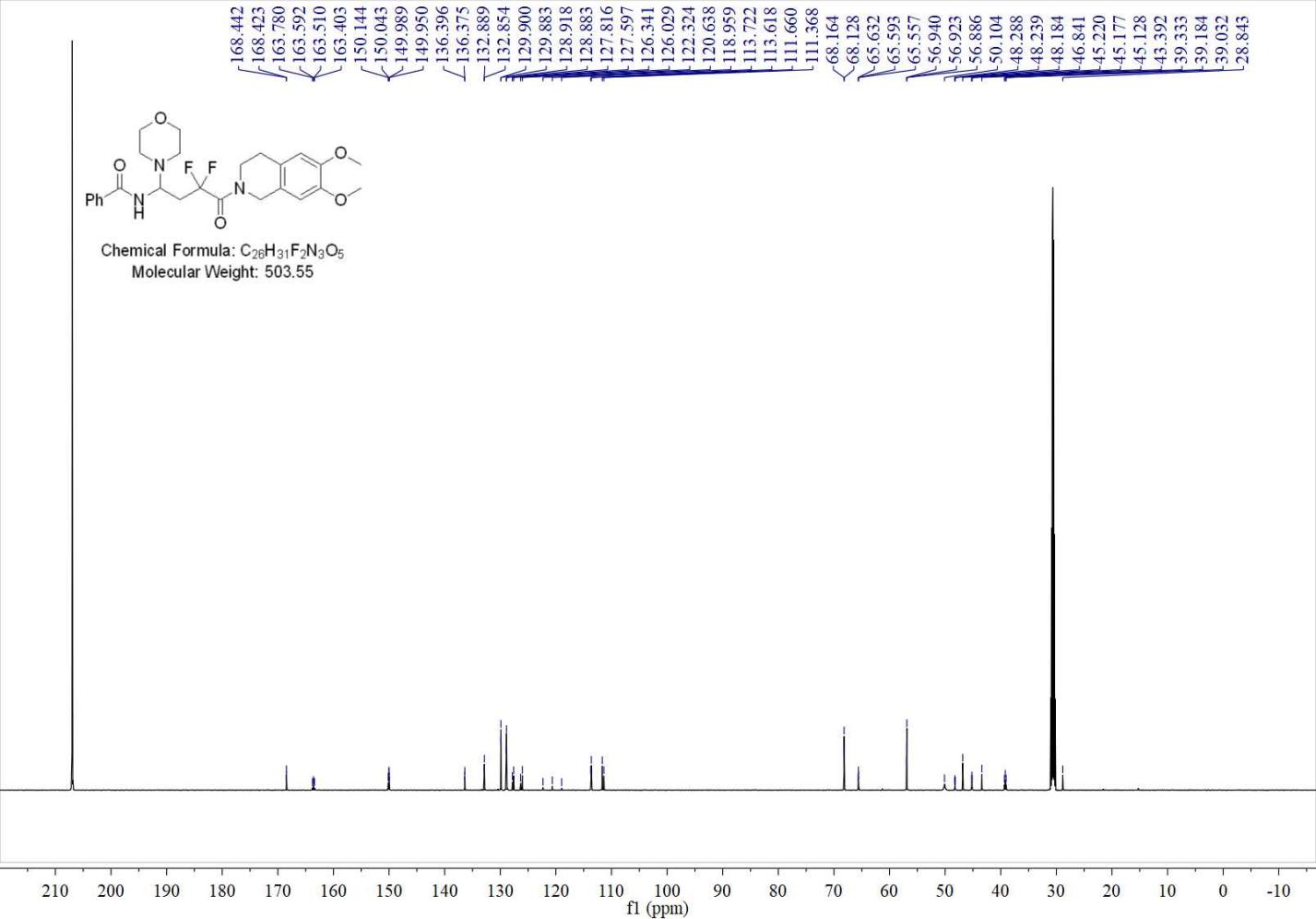
**

***N*-(4-(azepan-1-yl)-3,3-difluoro-1-morpholino-4-oxobutyl)benzamide (4h).**

**
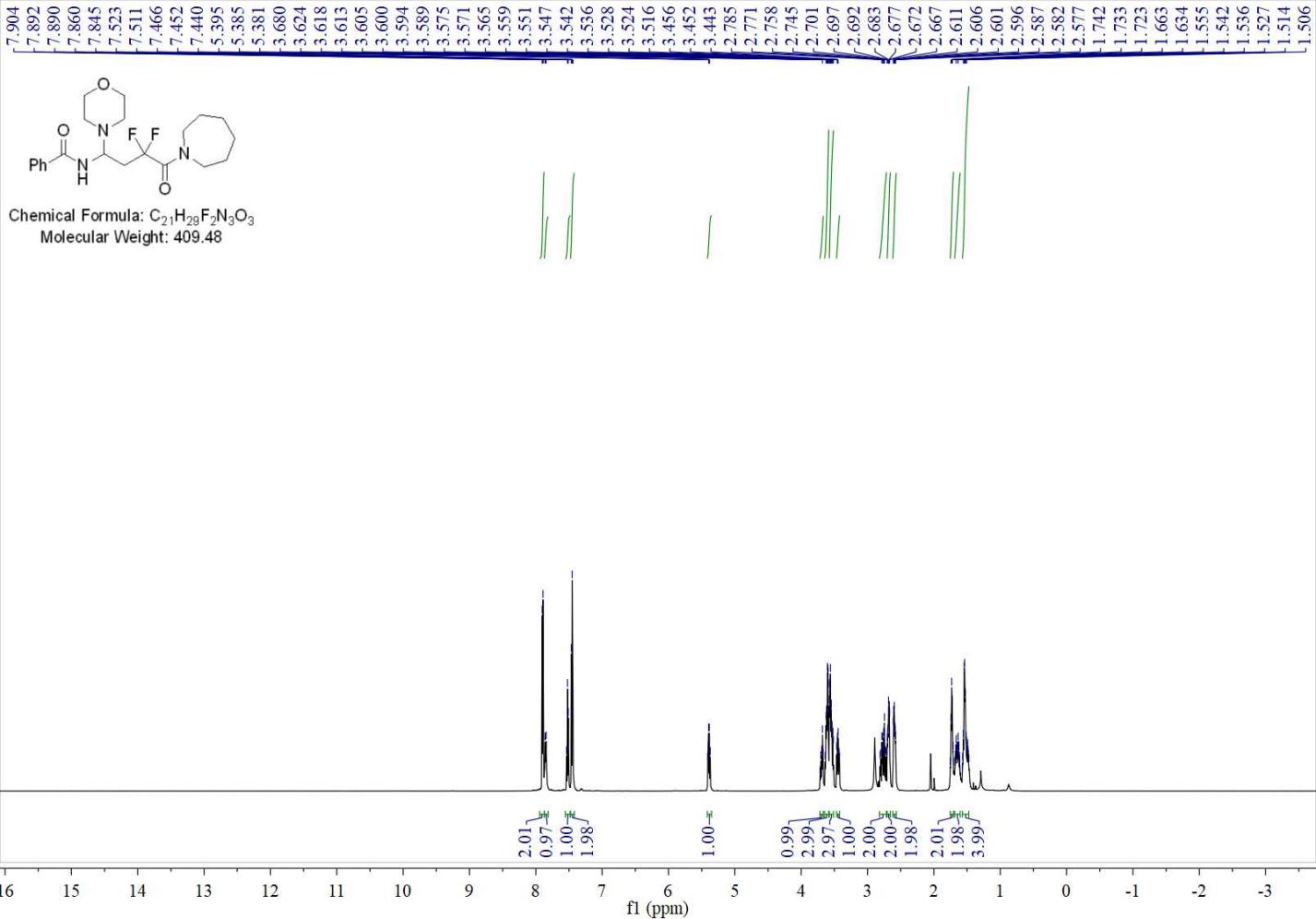

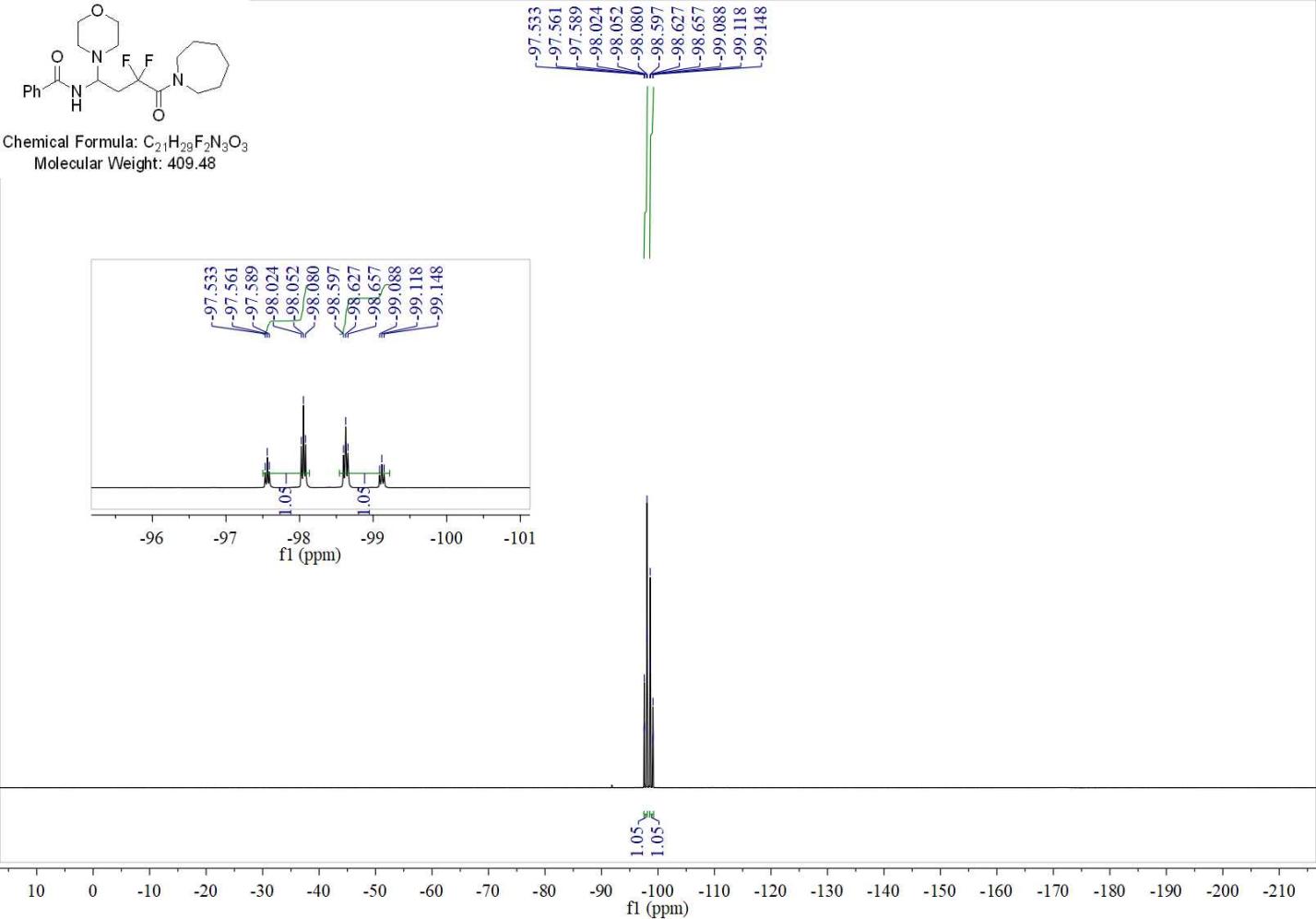

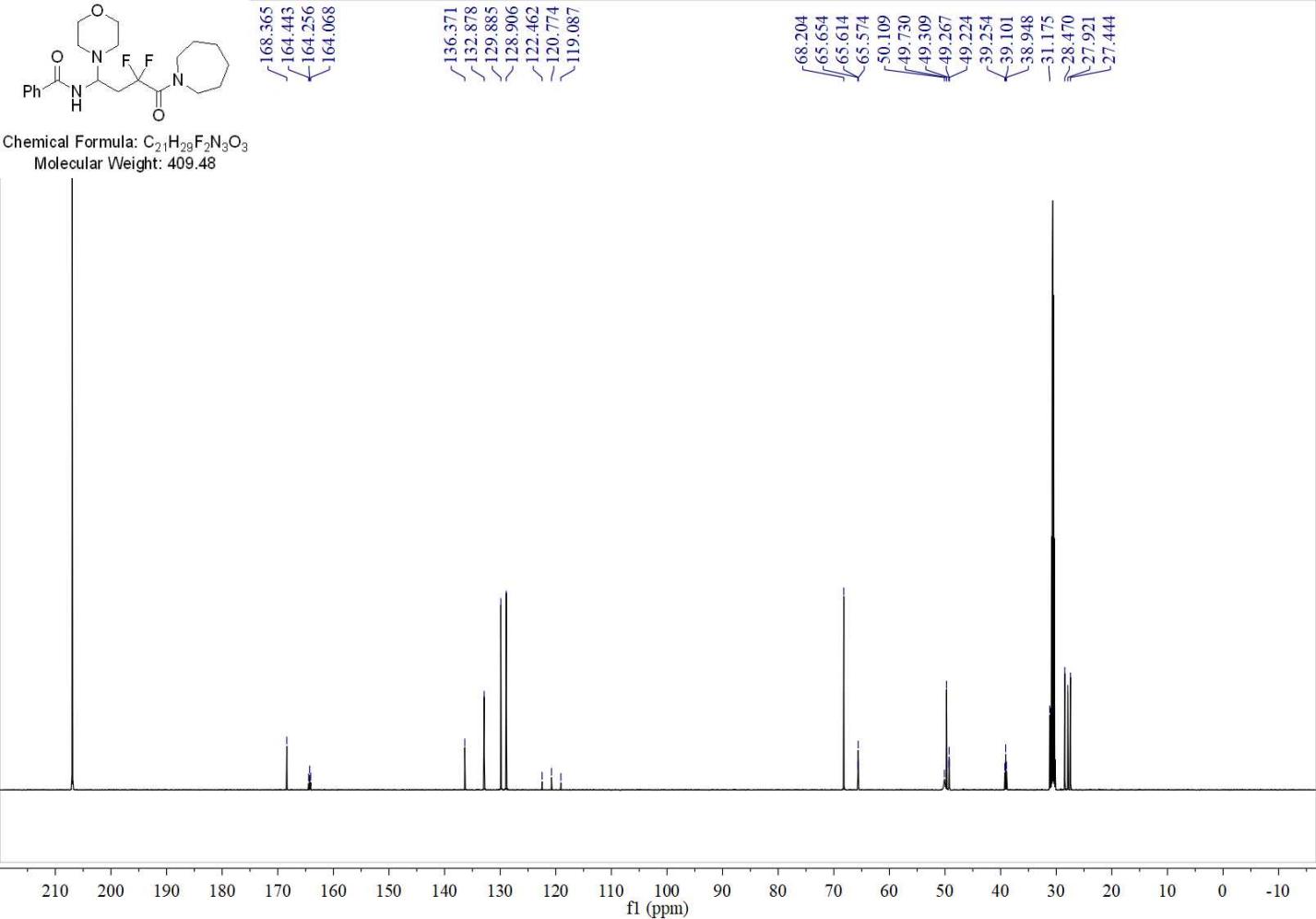
**

***N*-(4-(tert-butylamino)-3,3-difluoro-1-morpholino-4-oxobutyl)benzamide (4i).**

**
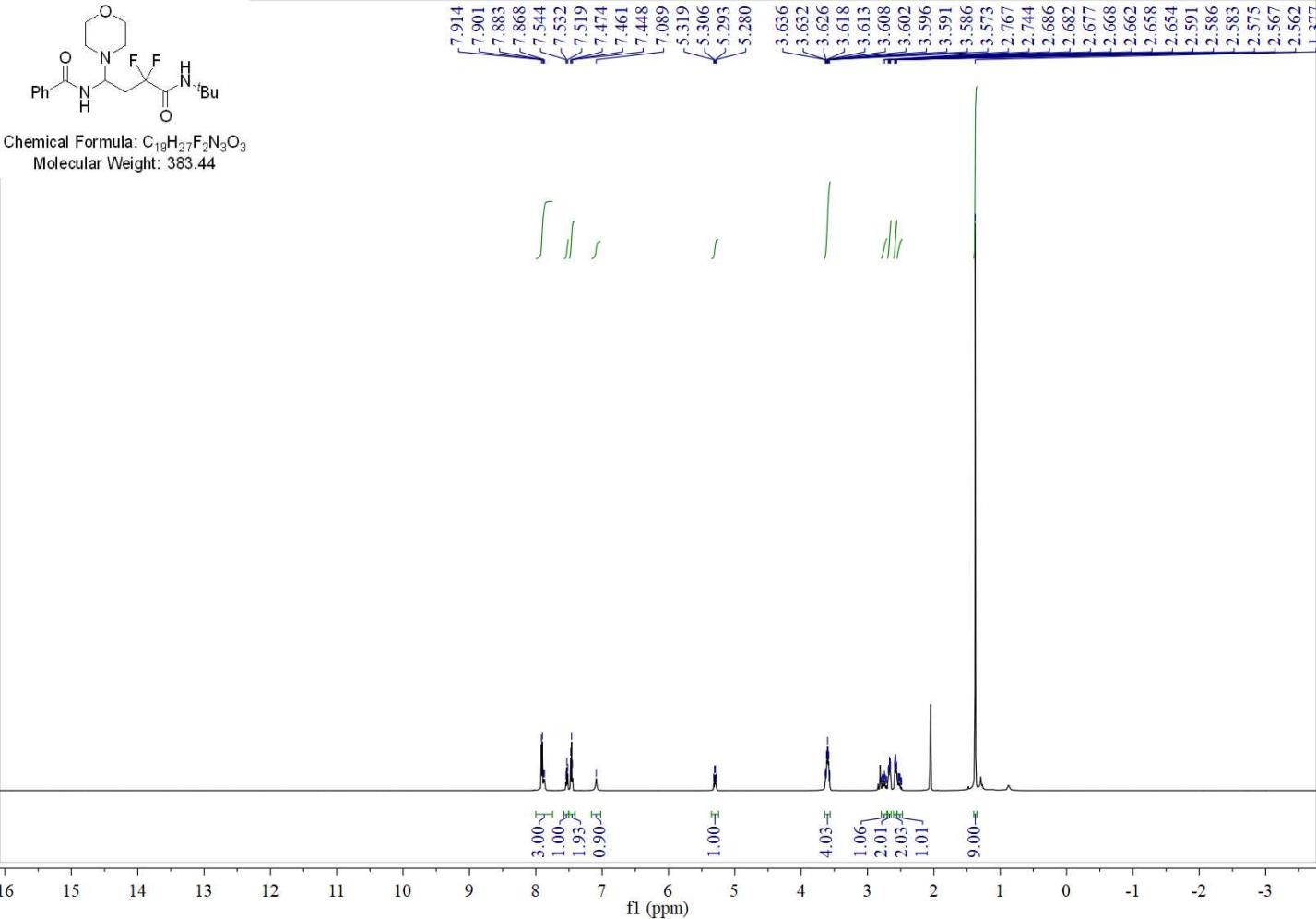

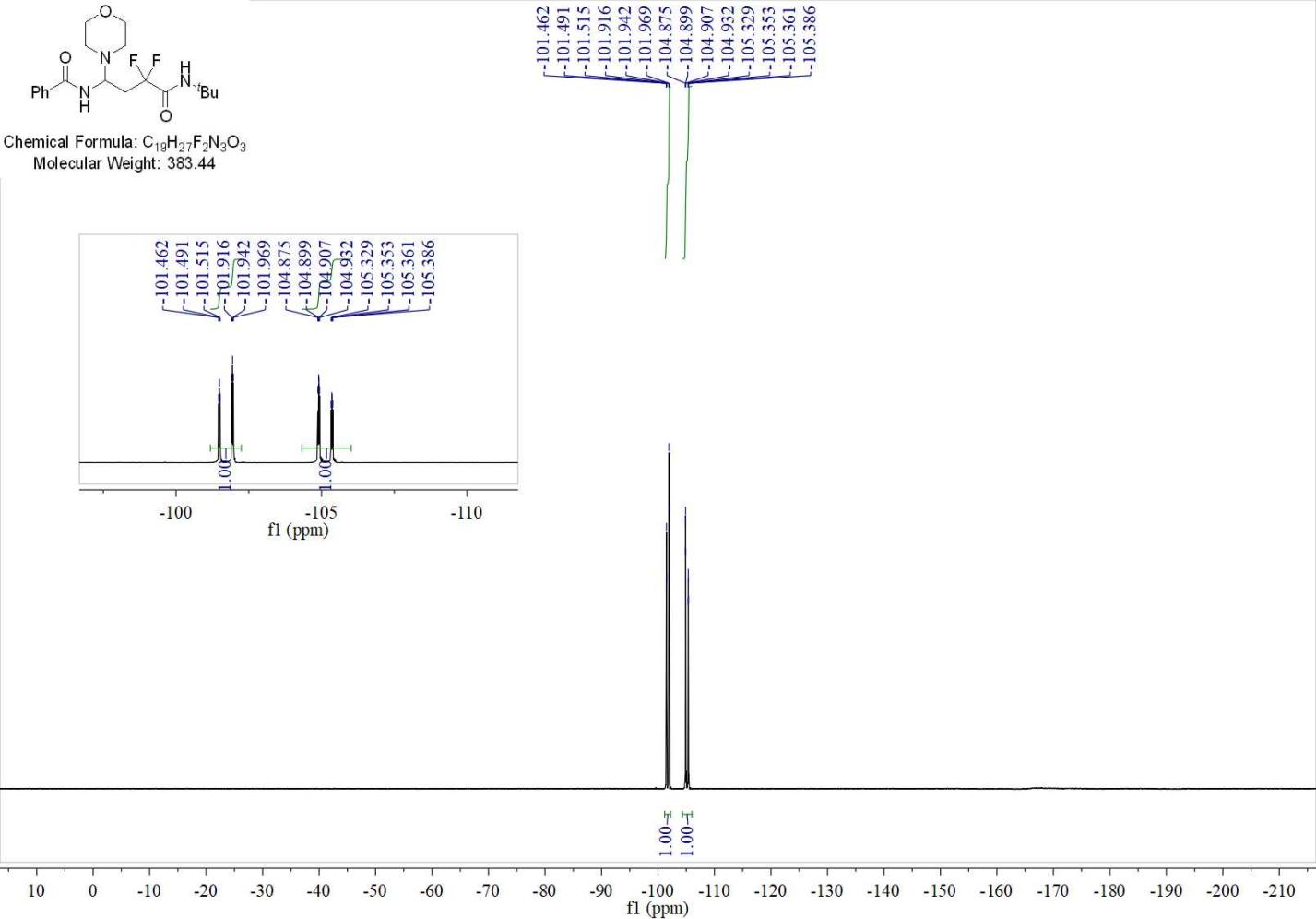

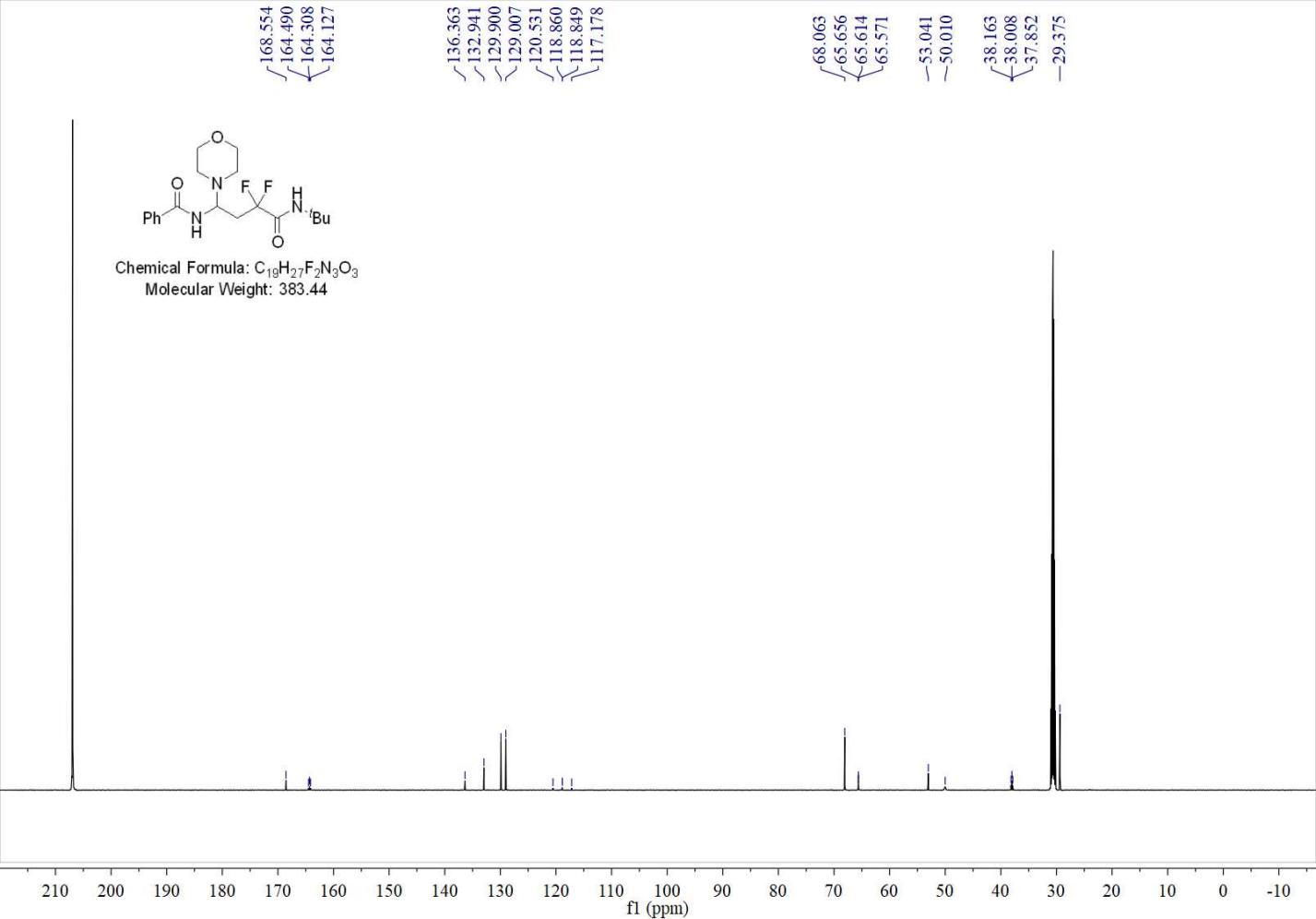
**

***N*-(4-(cyclohexylamino)-3,3-difluoro-1-morpholino-4-oxobutyl)benzamide (4j).**

**
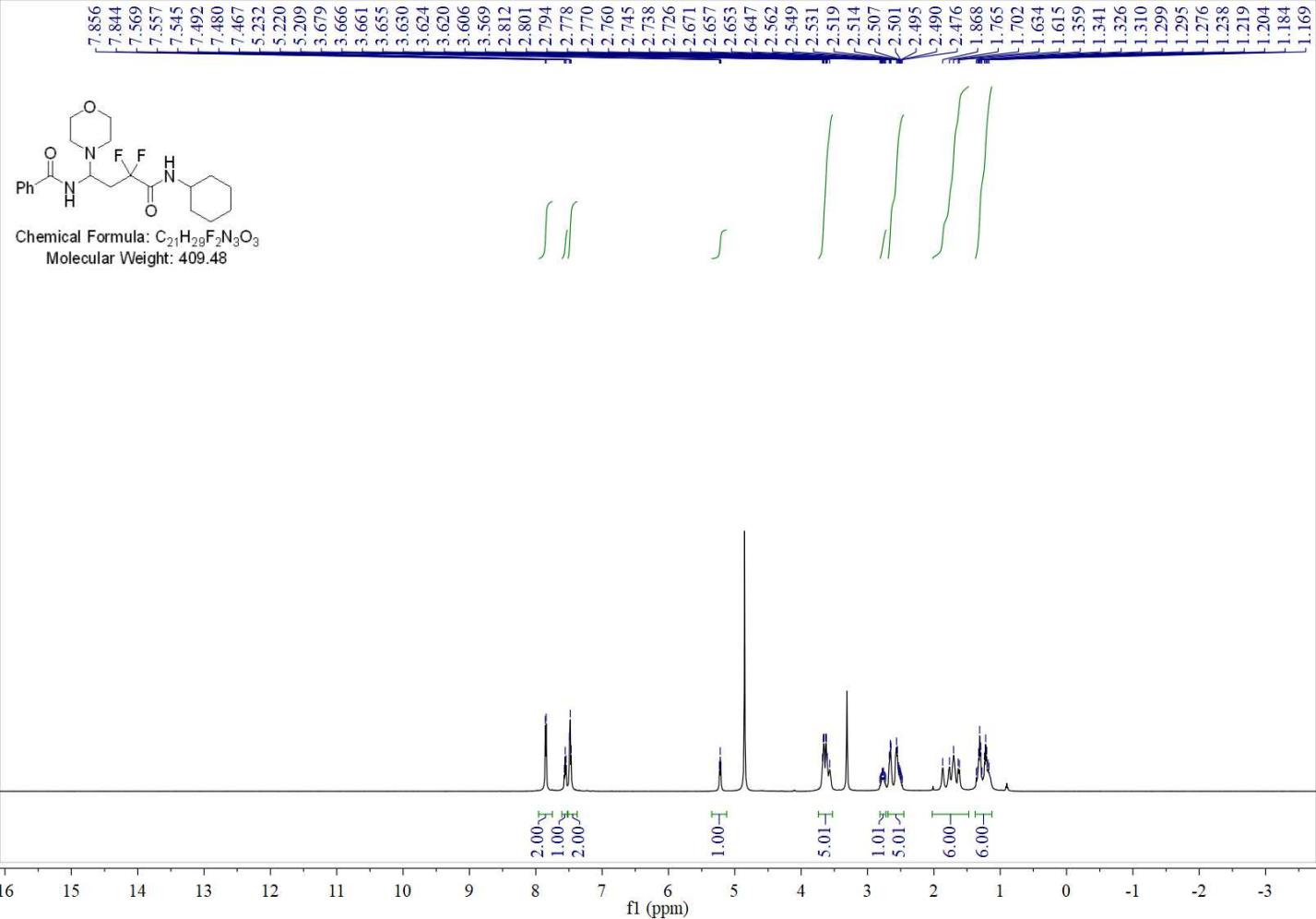

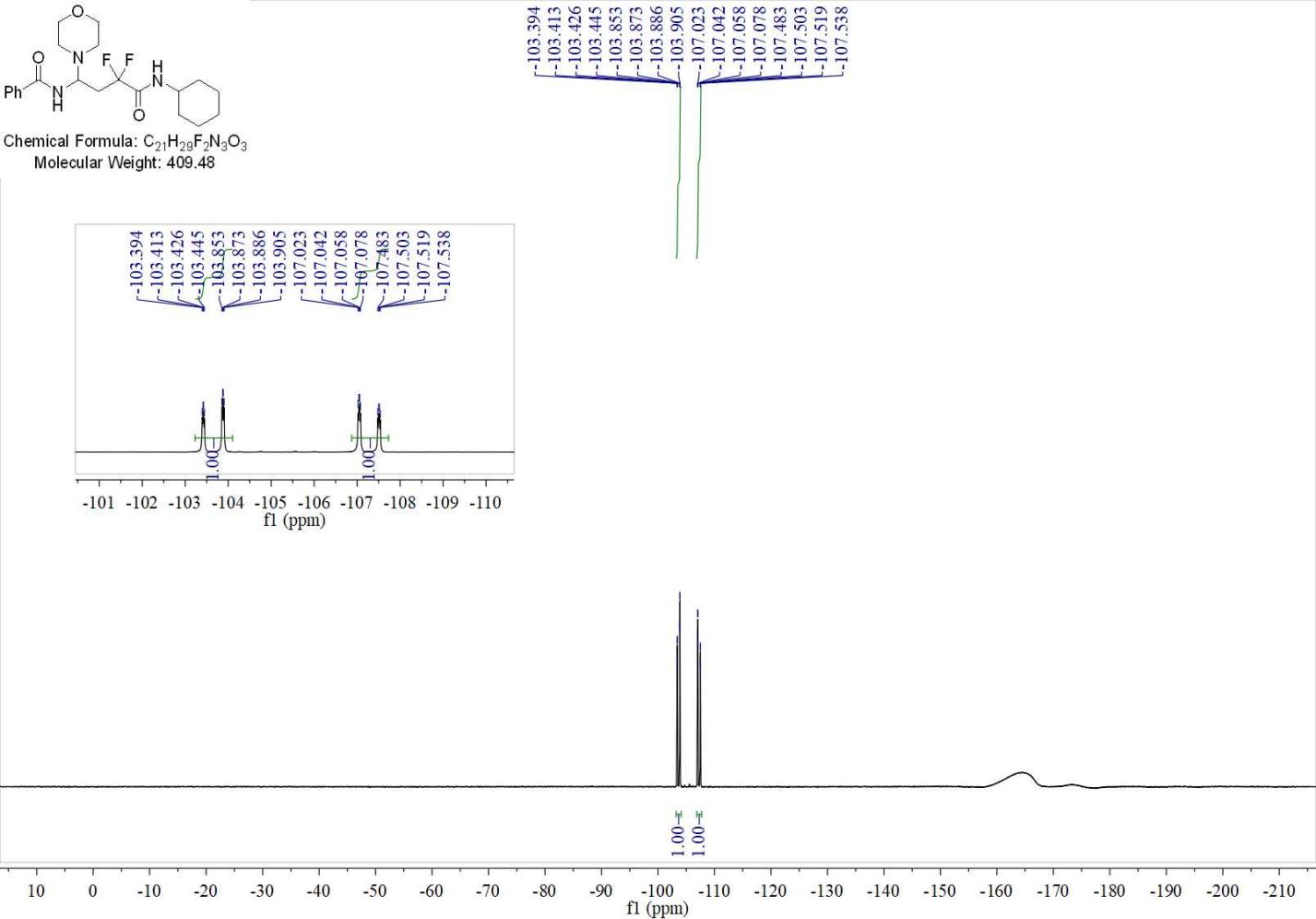

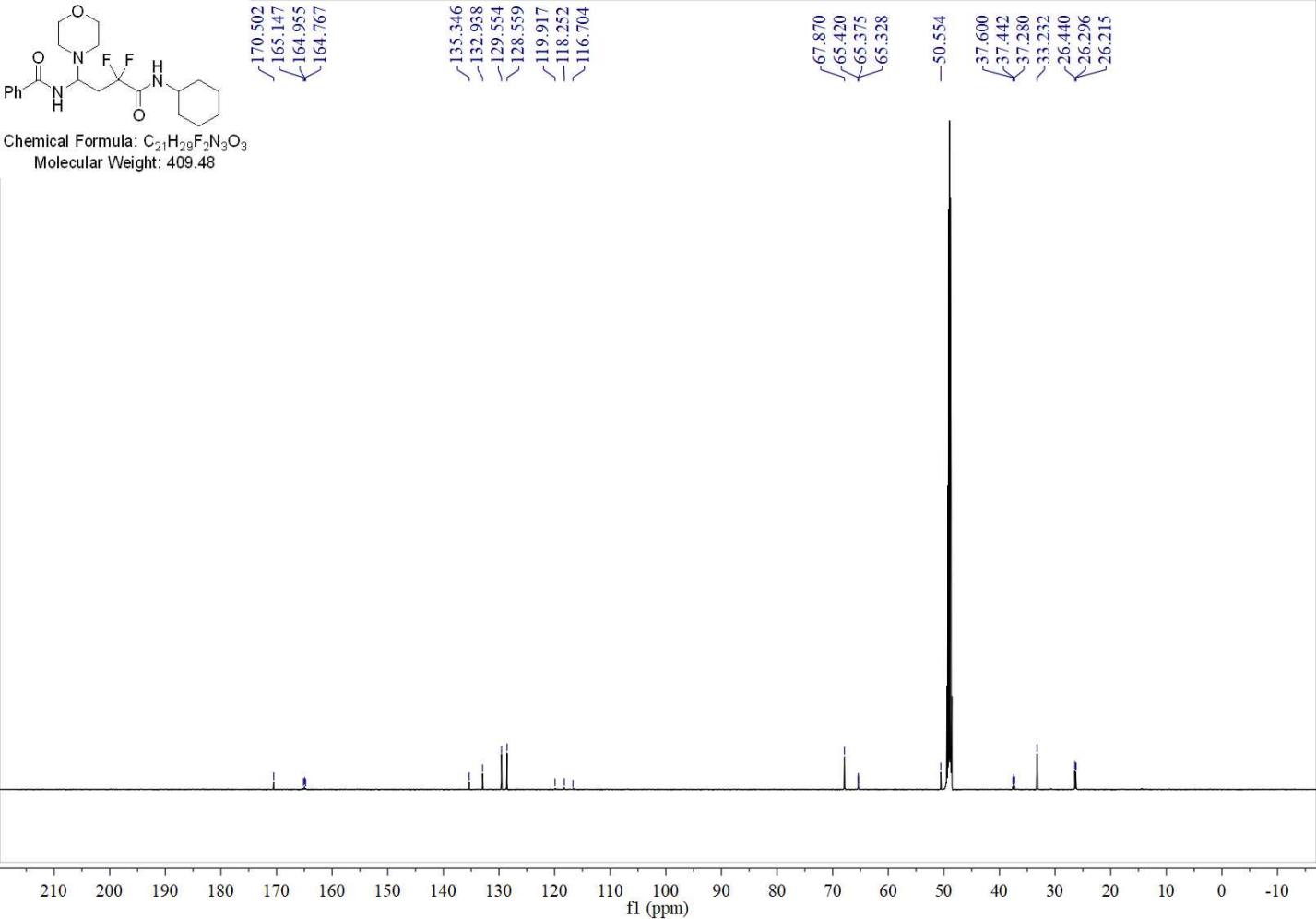
**

***N*-(3,3-difluoro-1-morpholino-4-oxo-4-(((R)-1-phenylethyl)amino)butyl)benzamide (4k).**

**
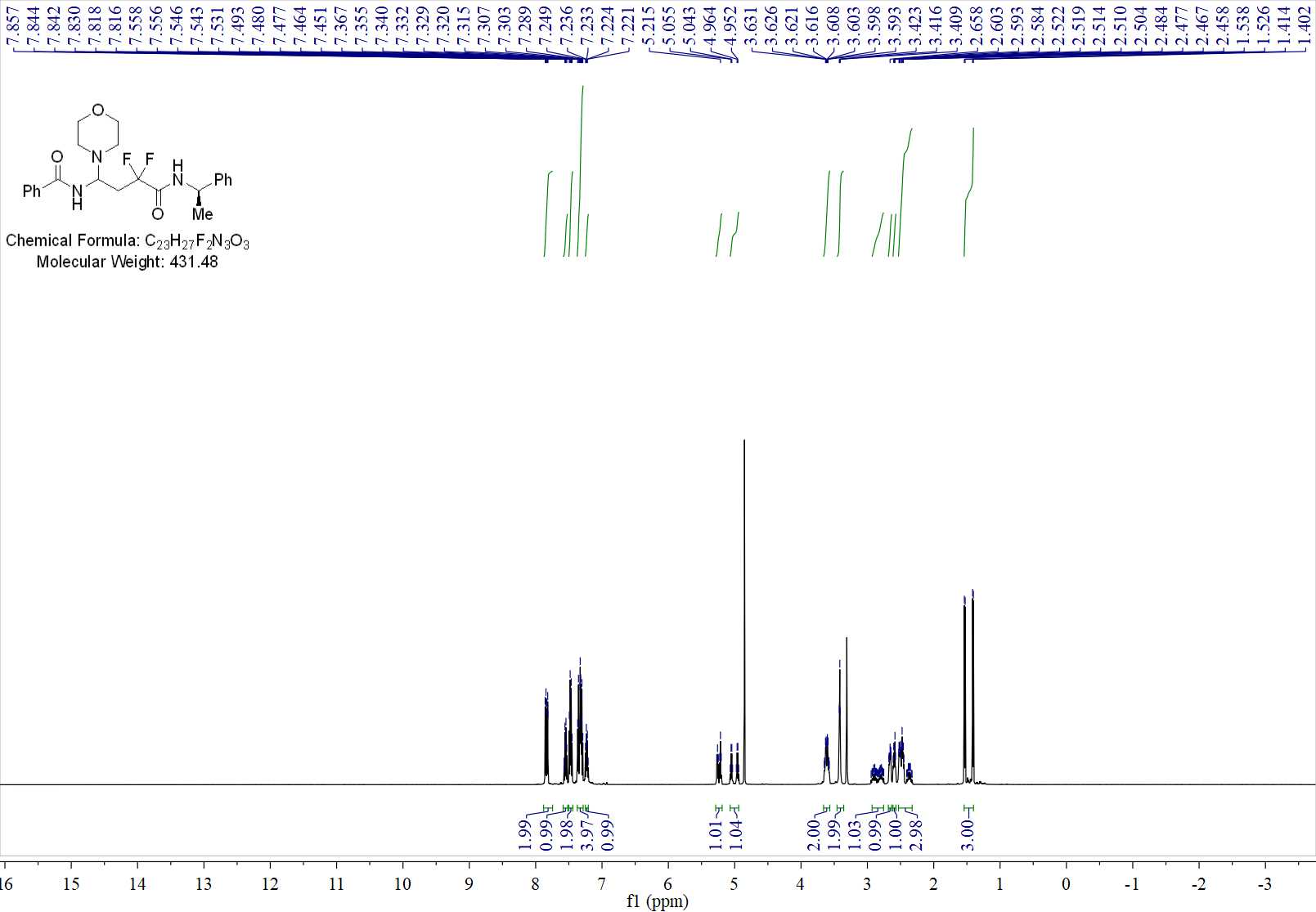

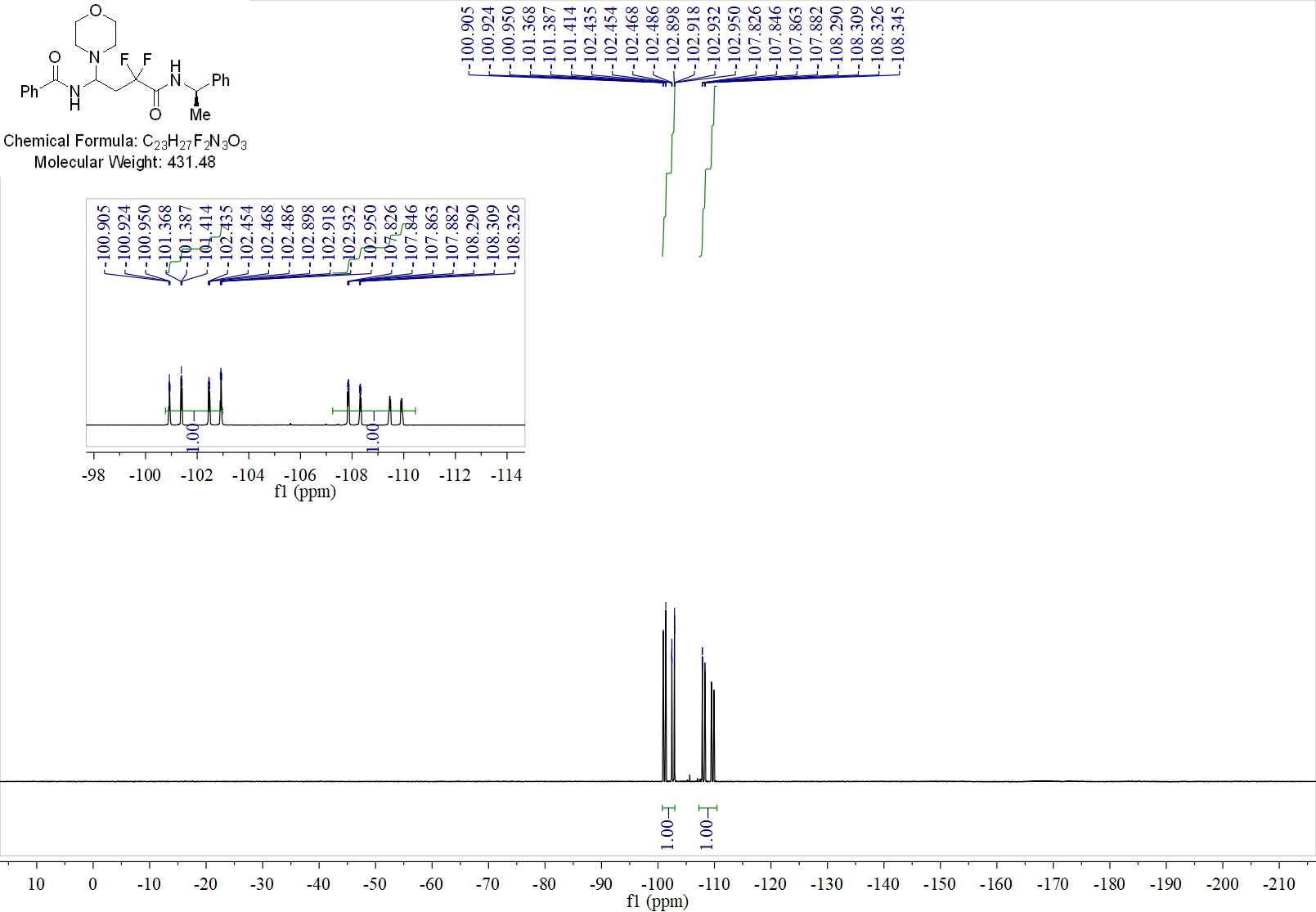

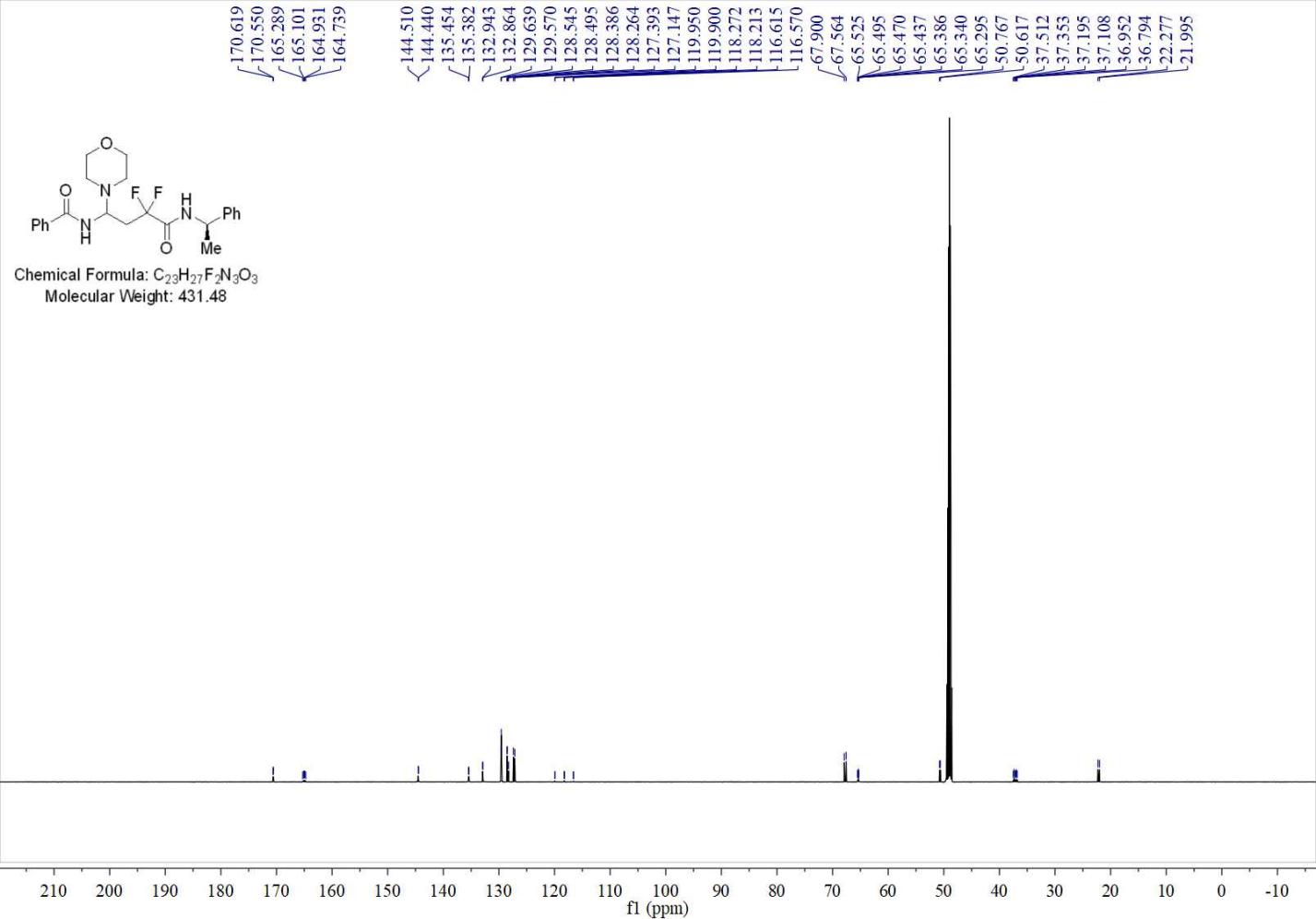
**

***N*-(4-((2-cyanoethyl)amino)-3,3-difluoro-1-morpholino-4-oxobutyl)benzamide (4l).**

**
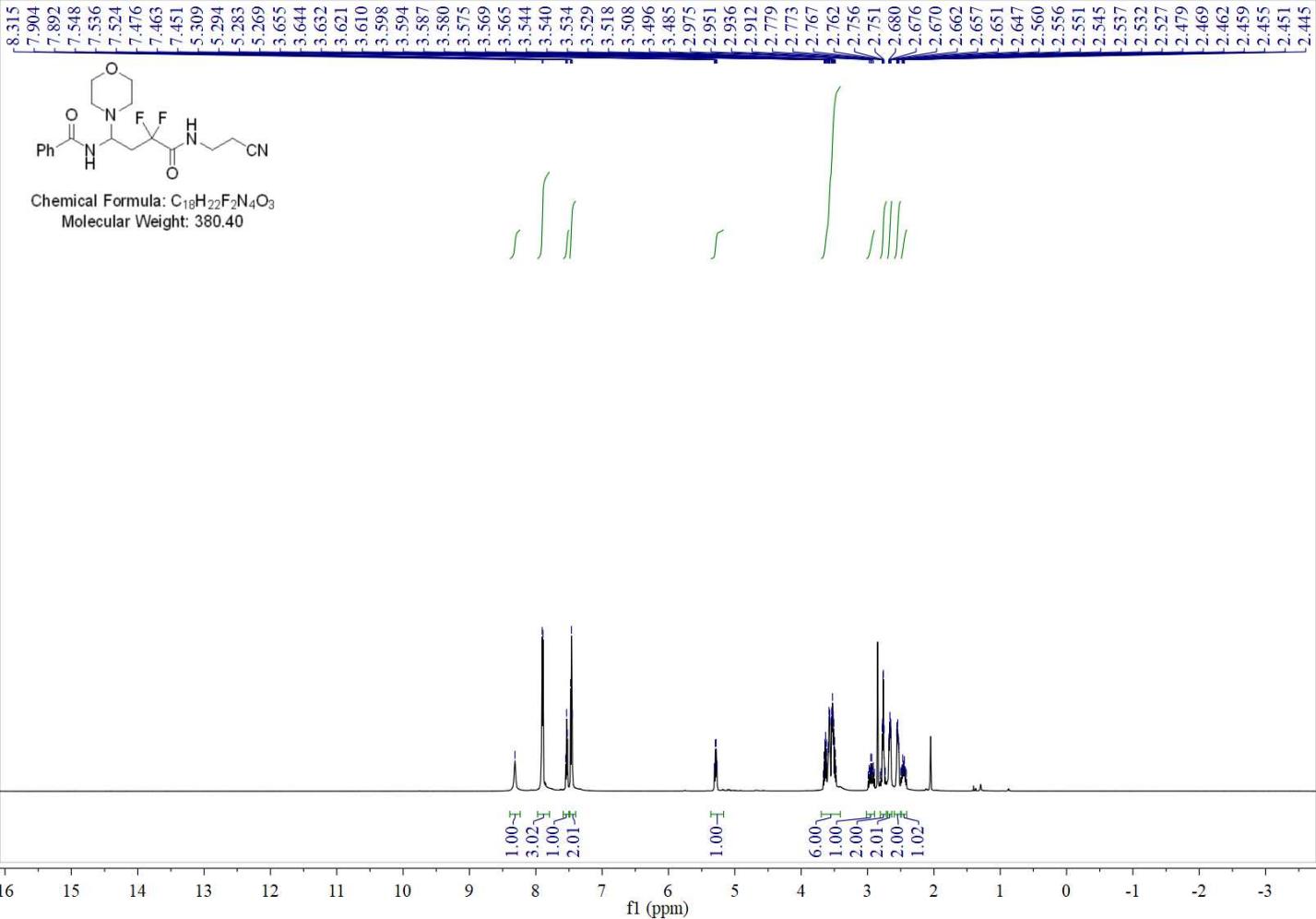

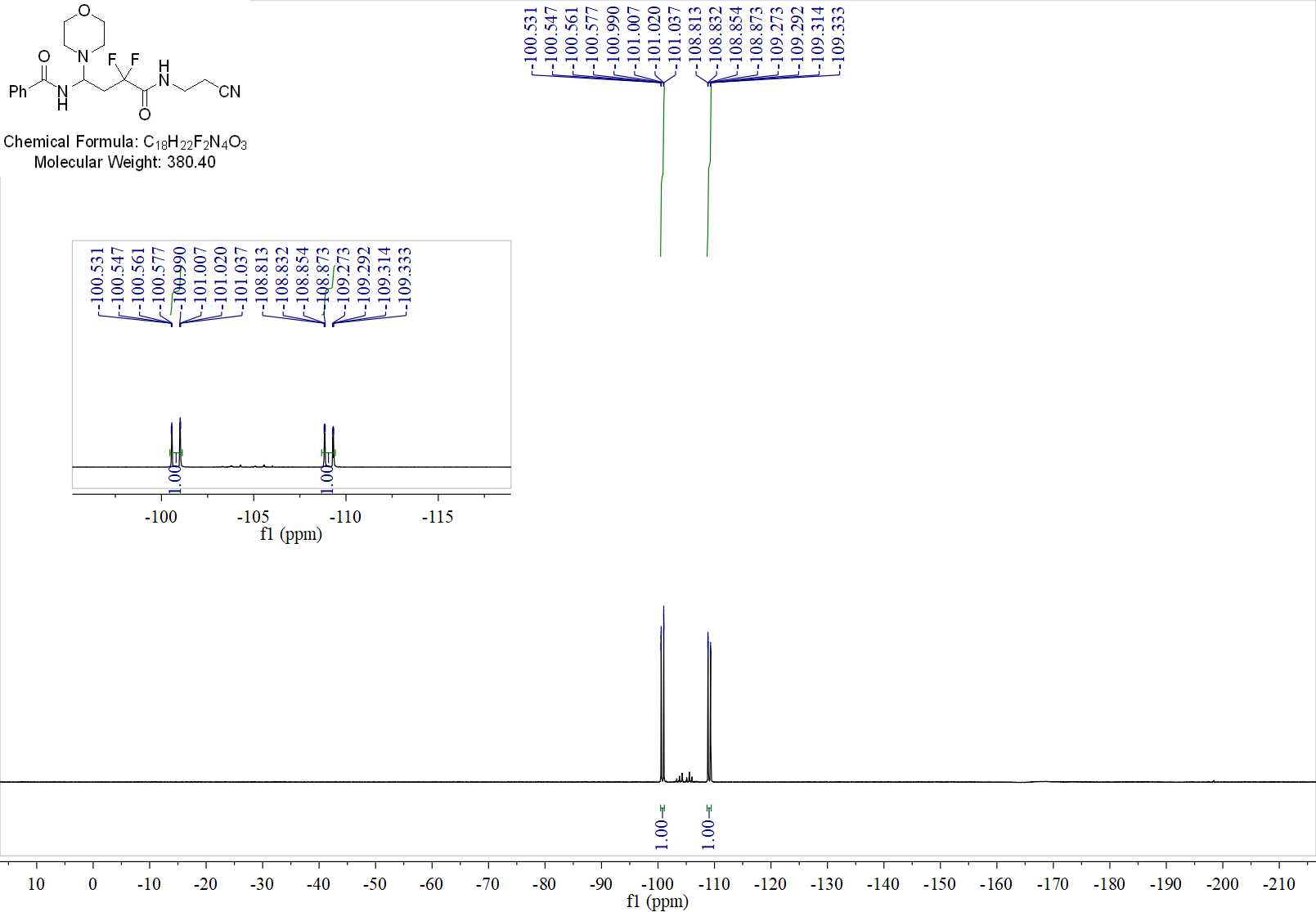

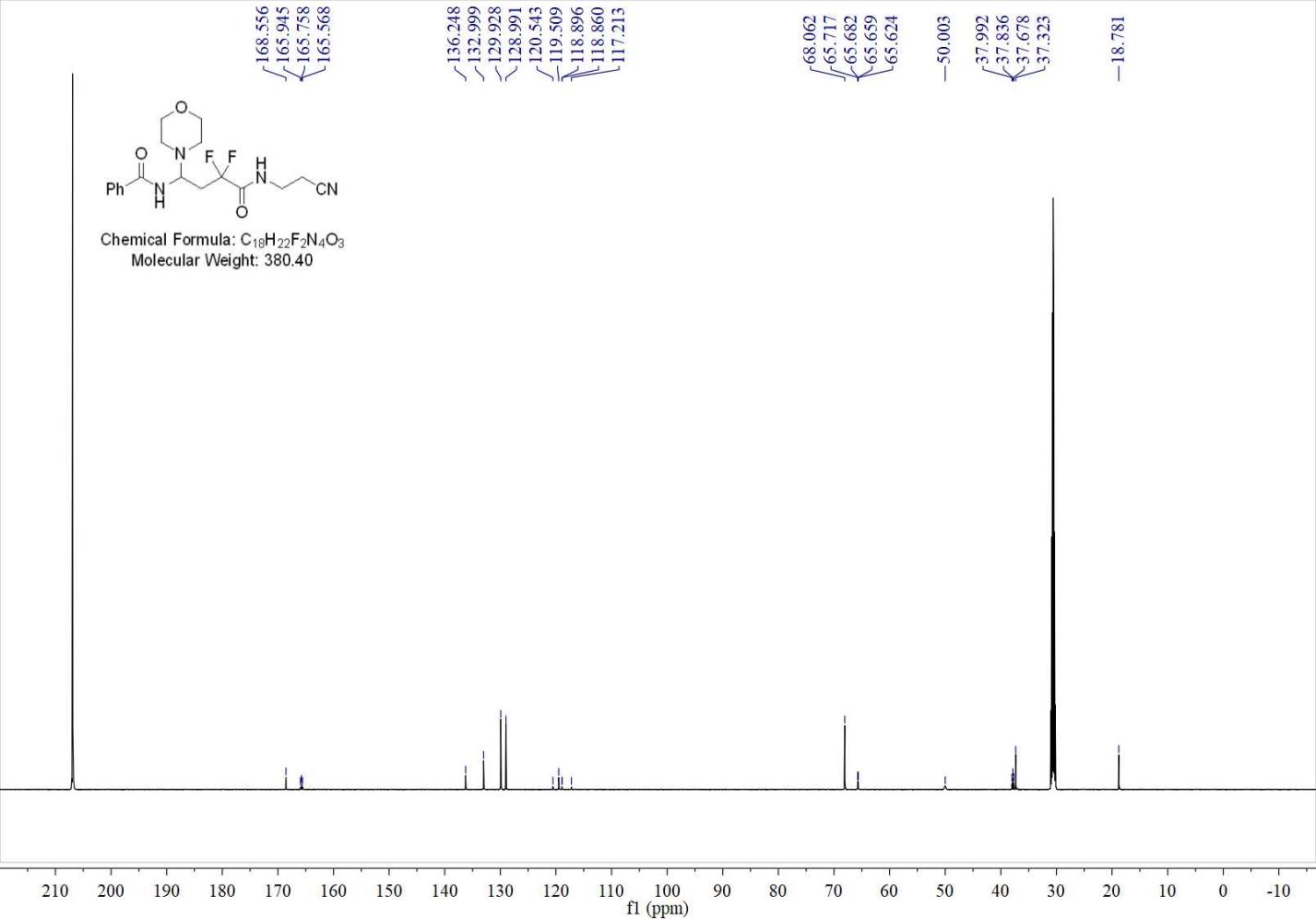
**

**Methyl 4-(4-benzamido-2,2-difluoro-4-morpholinobutanamido)butanoate (4m).**

**
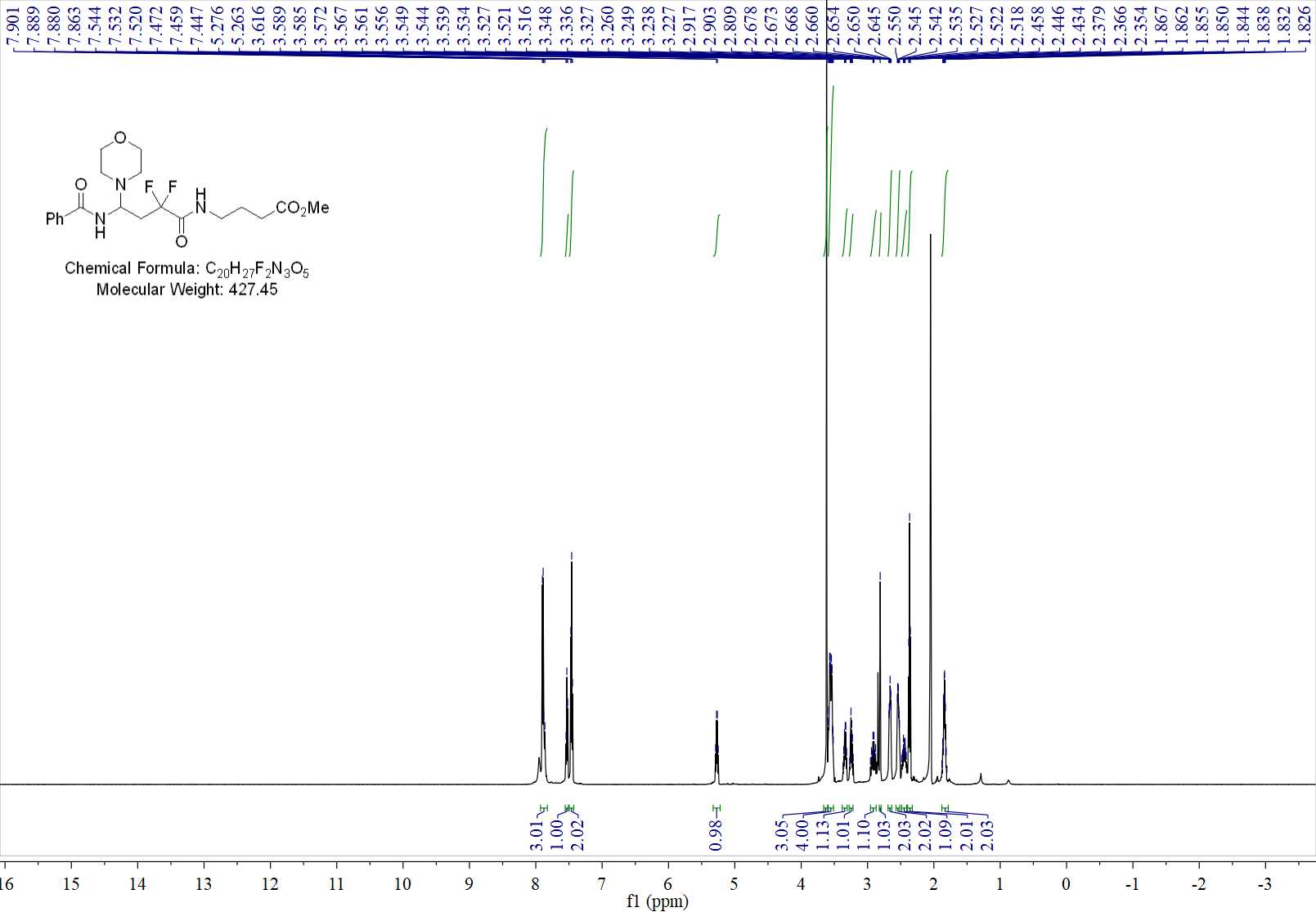

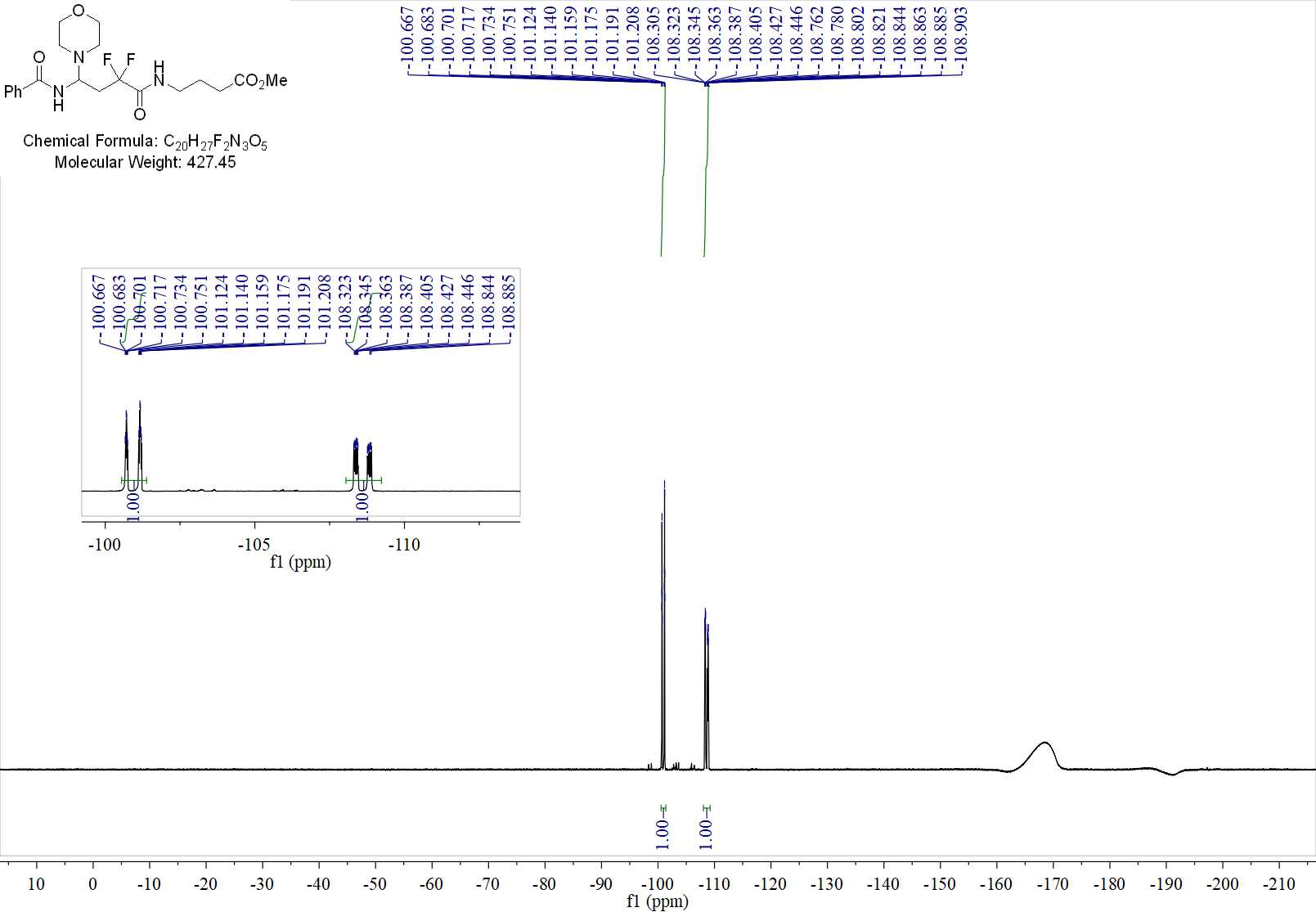

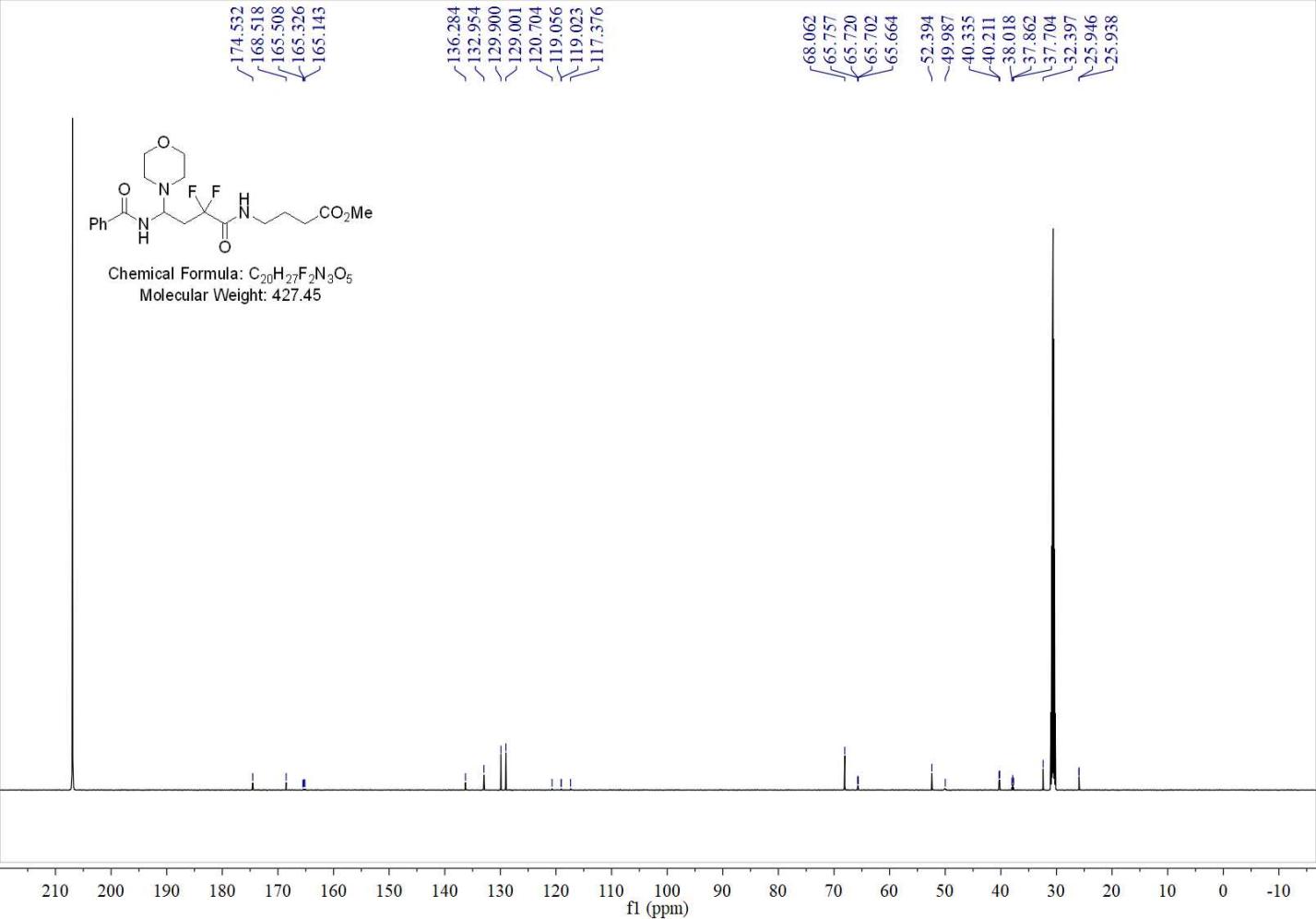
**

***Tert*-butyl (4-(4-benzamido-2,2-difluoro-4-morpholinobutanamido)butyl)carbamate (4n).**

**
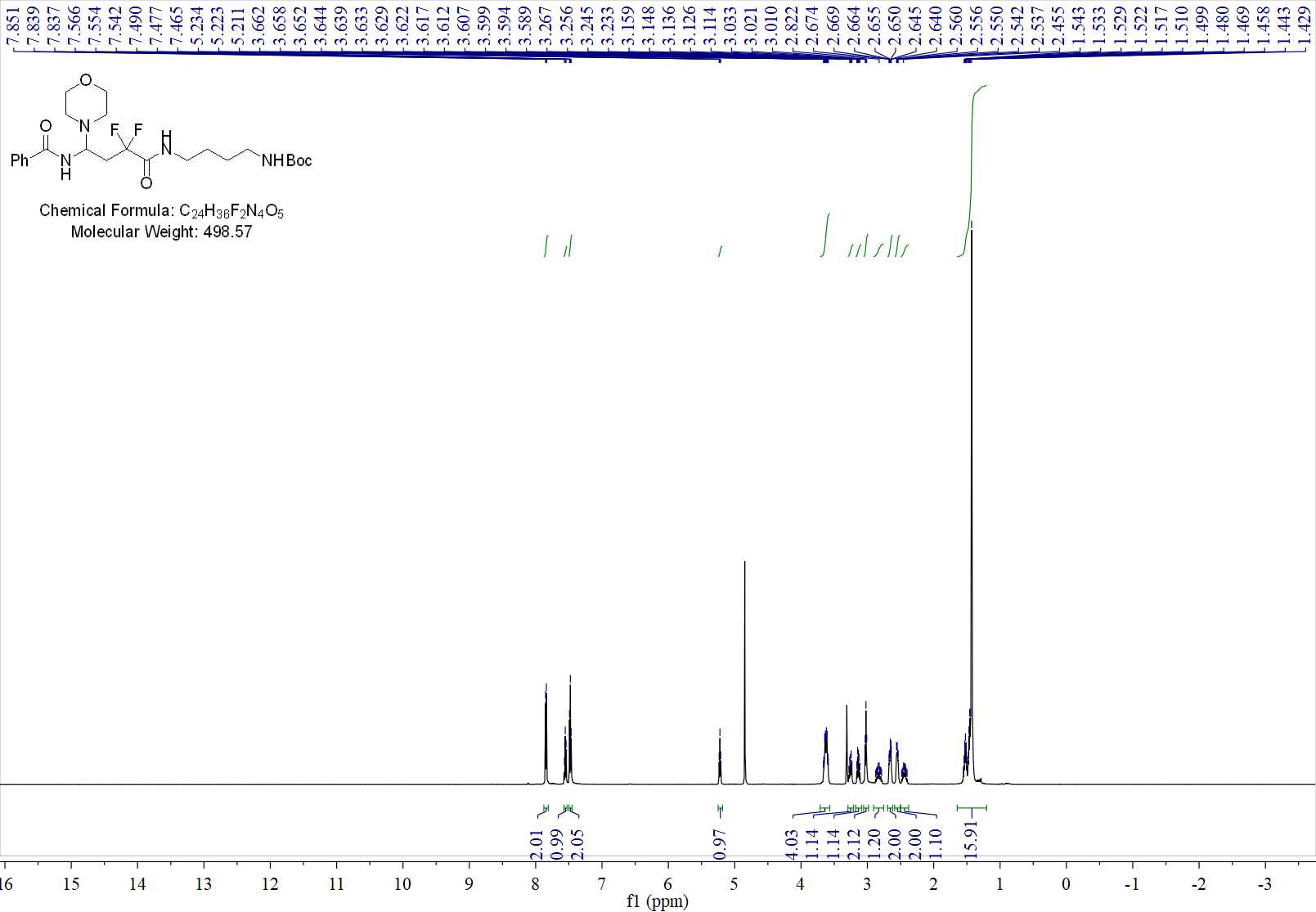

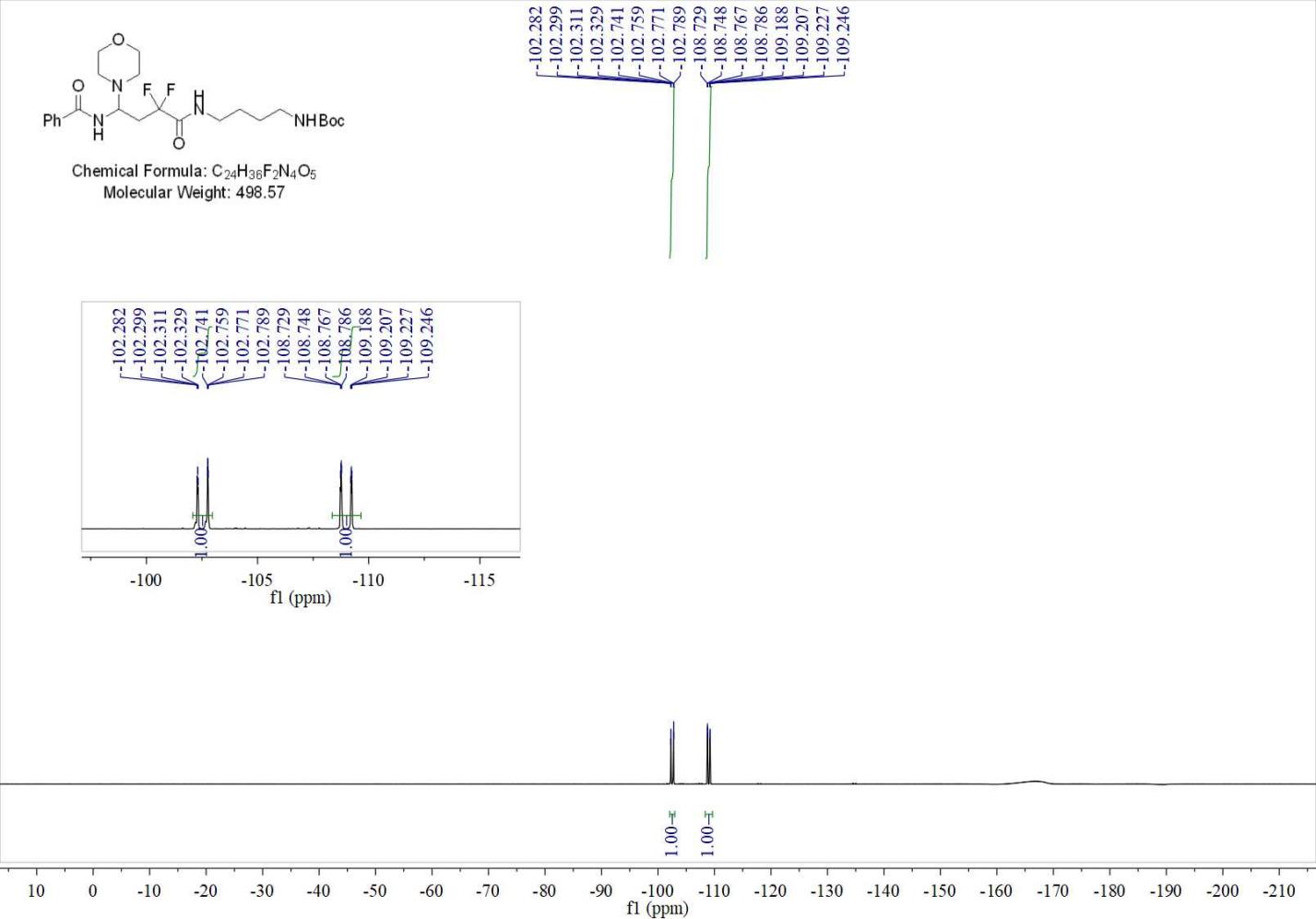
**

**Methyl N2-(4-benzamido-2,2-difluoro-4-morpholinobutanoyl)-N6-(tert-butoxycarbonyl)-L-lysinate (4o).**

**Methyl (4-benzamido-2,2-difluoro-4-morpholinobutanoyl)-L-valinate (4p).**

**Methyl (4-benzamido-2,2-difluoro-4-morpholinobutanoyl)-L-methioninate (4q).**

**Methyl (4-benzamido-2,2-difluoro-4-morpholinobutanoyl)-L-tryptophanate (4r).**

**Ethyl 4-benzamido-2,2-difluoro-4-morpholinobutanoate (4s).**

***N*-(3-fluoro-1,4-dimorpholino-4-oxobutyl)benzamide (4t).**

**Methyl 4-(4-benzamido-2,2-difluoro-4-morpholinobutanamido)-3-(4-chlorophenyl)butanoate (7a).**

**Methyl (3S)-3-((4-benzamido-2,2-difluoro-4-morpholinobutanamido)methyl)-5-methylhexanoate (7b).**

***N*-(4-(diethylamino)-1-((2R,6S)-2,6-dimethylmorpholino)-3,3-difluoro-4-oxobutyl)benzamide (5a).**

***N*-(4-(diethylamino)-3,3-difluoro-4-oxo-1-thiomorpholinobutyl)benzamide (5b).**

***Tert*-butyl 4-(1-benzamido-4-(diethylamino)-3,3-difluoro-4-oxobutyl)piperazine-1-carboxylate (5c).**

***N*-(4-(diethylamino)-3,3-difluoro-4-oxo-1-(4-(pyrimidin-2-yl)piperazin-1-yl)butyl)benzamide (5d).**

***N*-(1-(4-(benzo[d]isothiazol-3-yl)piperazin-1-yl)-4-(diethylamino)-3,3-difluoro-4-oxobutyl)benzamide (5e).**

***N*-(4-(diethylamino)-1-(4,4-difluoropiperidin-1-yl)-3,3-difluoro-4-oxobutyl)benzamide (5f).**

***N*-(4-(diethylamino)-3,3-difluoro-4-oxo-1-(4-phenylpiperidin-1-yl)butyl)benzamide (5g).**

***N*-(1-(4-benzylpiperidin-1-yl)-4-(diethylamino)-3,3-difluoro-4-oxobutyl)benzamide (5h).**

**Ethyl 1-(1-benzamido-4-(diethylamino)-3,3-difluoro-4-oxobutyl)piperidine-4-carboxylate (5i).**

***N*-(4-(diethylamino)-3,3-difluoro-1-(4-(hydroxymethyl)piperidin-1-yl)-4-oxobutyl)benzamide (5j).**

***N*-(4-(diethylamino)-3,3-difluoro-1-(4-(hydroxydiphenylmethyl)piperidin-1-yl)-4-oxobutyl)benzamide (5k).**

***N*-(4-(diethylamino)-3,3-difluoro-1-(4-hydroxypiperidin-1-yl)-4-oxobutyl)benzamide (5l).**

***Tert*-butyl (1-(1-benzamido-4-(diethylamino)-3,3-difluoro-4-oxobutyl)piperidin-4-yl)carbamate (5m).**

***N*-(1-(4-(8-chloro-5,6-dihydro-11H-benzo[5,6]cyclohepta[1,2-b]pyridin-11-ylidene)piperidin-1-yl)-4-(diethylamino)-3,3-difluoro-4-oxobutyl)benzamide (8a).**

***N*-(1-(4-(3-((benzo[d][1,3]dioxol-5-yloxy)methyl)-4-fluorophenyl)piperidin-1-yl)-4-(diethylamino)-3,3-difluoro-4-oxobutyl)benzamide (8b).**

***N*-(4-(diethylamino)-1-(4-(2-((2,4-dimethylphenyl)thio)phenyl)piperazin-1-yl)-3,3-difluoro-4-oxobutyl)benzamide (8c).**

***N*-(1-(4-(8-chlorodibenzo[b,f][1,4]oxazepin-11-yl)piperazin-1-yl)-4-(diethylamino)-3,3-difluoro-4-oxobutyl)benzamide (8d).**

***N*-(1-(4-(2-(3-cyano-4-isobutoxyphenyl)-4-methylthiazole-5-carbonyl)piperazin-1-yl)-4-(diethylamino)-3,3-difluoro-4-oxobutyl)benzamide (8e).**

***N*-(4-(diethylamino)-3,3-difluoro-1-morpholino-4-oxobutyl)-4-methoxybenzamide (6a).**

***N*-(4-(diethylamino)-3,3-difluoro-1-morpholino-4-oxobutyl)-4-(trifluoromethyl)benzamide (6b).**

***N*-(4-(diethylamino)-3,3-difluoro-1-morpholino-4-oxobutyl)-2,3,4,5,6-pentafluorobenzamide (6c).**

***Tert*-butyl (4-(diethylamino)-3,3-difluoro-1-morpholino-4-oxobutyl)carbamate (6d).**

***N*-(3,3-difluoro-4-(2-hydroxyethoxy)butyl)benzamide (9).**

***N*-(3,3-difluoro-1-morpholino-4-oxooctyl)benzamide (10).**

**(Z)-*N*-(3-fluoro-1,4-dimorpholino-4-oxobut-2-en-1-yl)benzamide (11a).**

**Ethyl (Z)-4-benzamido-2-fluoro-4-morpholinobut-2-enoate (11b).**

**Ethyl (E)-benzoyl(3-morpholino-3-oxoprop-1-en-1-yl)carbamate (12).**

**3-(3,4-dihydronaphthalen-1-yl)-N,N-diethyl-2,2-difluoropropanamide (14).**

***N,N*-diethyl-2,2-difluoro-4,4-diphenylbutanamide (16).**
